# Women’s views about current and future management of Ductal Carcinoma in Situ (DCIS): A mixed-methods study

**DOI:** 10.1371/journal.pone.0288972

**Published:** 2023-07-21

**Authors:** Brooke Nickel, Kirsten McCaffery, Jesse Jansen, Alexandra Barratt, Nehmat Houssami, Christobel Saunders, Andrew Spillane, Claudia Rutherford, Kirsty Stuart, Geraldine Robertson, Ann Dixon, Jolyn Hersch

**Affiliations:** 1 Faculty of Medicine and Health, Wiser Healthcare, Sydney School of Public Health, The University of Sydney, Sydney, NSW, Australia; 2 Faculty of Medicine and Health, Sydney Health Literacy Lab, Sydney School of Public Health, The University of Sydney, Sydney, NSW, Australia; 3 Department of Family Medicine, Care and Public Health Research Institute (CAPHRI), Maastricht University, Maastricht, The Netherlands; 4 The Daffodil Centre, The University of Sydney, a Joint Venture with Cancer Council NSW, Sydney, NSW, Australia; 5 Department of Surgery, Melbourne Medical School, University of Melbourne, Melbourne, VIC, Australia; 6 Northern Clinical School, The University of Sydney, St Leonards, NSW, Australia; 7 Mater Hospital, Wollstonecraft, NSW, Australia; 8 Royal North Shore Hospital, St Leonards, NSW, Australia; 9 Faculty of Science, School of Psychology, The University of Sydney, Sydney, NSW, Australia; 10 Faculty of Medicine and Health, Sydney Nursing School, The University of Sydney, Sydney, NSW, Australia; 11 Department of Radiation Oncology, Crown Princess Mary Cancer Centre, Westmead, NSW, Australia; 12 Westmead Breast Cancer Institute, Westmead, NSW, Australia; 13 Westmead Clinical School, The University of Sydney, Sydney, NSW, Australia; 14 Breast Cancer Network Australia, Camberwell, VIC, Australia; 15 Faculty of Science, Sydney Neuropsychology Clinic, School of Psychology, The University of Sydney, Sydney, NSW, Australia; Local Health Authority Caserta: Azienda Sanitaria Locale Caserta, ITALY

## Abstract

Management of low-risk ductal carcinoma in situ (DCIS) is controversial, with clinical trials currently assessing the safety of active monitoring amidst concern about overtreatment. Little is known about general community views regarding DCIS and its management. We aimed to explore women’s understanding and views about low-risk DCIS and current and potential future management options. This mixed-method study involved qualitative focus groups and brief quantitative questionnaires. Participants were screening-aged (50–74 years) women, with diverse socioeconomic backgrounds and no personal history of breast cancer/DCIS, recruited from across metropolitan Sydney, Australia. Sessions incorporated an informative presentation interspersed with group discussions which were audio-recorded, transcribed and analysed thematically. Fifty-six women took part in six age-stratified focus groups. Prior awareness of DCIS was limited, however women developed reasonable understanding of DCIS and the relevant issues. Overall, women expressed substantial support for active monitoring being offered as a management approach for low-risk DCIS, and many were interested in participating in a hypothetical clinical trial. Although some women expressed concern that current management may sometimes represent overtreatment, there were mixed views about personally accepting monitoring. Women noted a number of important questions and considerations that would factor into their decision making. Our findings about women’s perceptions of active monitoring for DCIS are timely while results of ongoing clinical trials of monitoring are awaited, and may inform clinicians and investigators designing future, similar trials. Exploration of offering well-informed patients the choice of non-surgical management of low-risk DCIS, even outside a clinical trial setting, may be warranted.

## Introduction

Ductal carcinoma in situ (DCIS) is a non-invasive breast malignancy. DCIS differs from invasive cancer as the malignant cells are fully contained within the basement membrane of the breast ducts and have not spread into other breast tissue. DCIS however is heterogenous, ranging from indolent low-grade lesions to high-grade lesions with a higher risk of progression to invasive breast cancer. Across all grades of DCIS, estimates of rates of progression to invasive cancer vary from 14% to 53% after up to 31 years follow-up [1].

Before widespread breast screening, DCIS was rarely diagnosed. The incidence of DCIS has markedly increased since implementation of organised screening, and DCIS represents approximately 20% of screen-detected malignant lesions [2]. There are now concerns that screening has led to overdiagnosis and overtreatment of low-risk (small volume, low to intermediate grade) DCIS [3,4]. Yet, the majority of patients with DCIS of all grades are offered treatment, including surgery, radiotherapy and endocrine therapies which are similar to treatment for invasive cancer [5], importantly noting that chemotherapy is not administered in DCIS cases. These treatments may not improve overall survival which is already excellent, particularly for low-grade DCIS, and may cause detriments to health and quality of life including physical (e.g. cardiotoxicity, pain and fatigue) and psychological harm (e.g. negative effect on body image) [6–8]. To address harm from overtreatment of low-risk DCIS, active monitoring has been proposed as an alternative management strategy [9–11], and several ongoing clinical trials are assessing the outcomes of monitoring [12–14]. These trials compare standard treatment for low-risk DCIS to active monitoring (every 6 or 12 months) to determine whether women diagnosed with low-risk DCIS can safely avoid surgery, at least until and *if* progression occurs. Results from these trials are expected in a few years’ time, although all have faced recruitment challenges [15]. A similar trial proposed for Australia and New Zealand [16] did not gain funding to proceed.

DCIS is challenging to explain to patients [17], which often leaves patients confused about the meaning of their diagnosis [18], with exaggerated risk perceptions and anxiety [19]. Furthermore, our previous work [20] highlighted that healthcare professionals involved in managing DCIS generally feel uncomfortable recommending active monitoring as an option for low-risk DCIS. However, little is known about what women in the general community know about DCIS, and how they view the options and controversies around optimal management. Given that DCIS is primarily identified through breast screening, we aimed to qualitatively and quantitatively explore among women of screening age their understanding and views about DCIS, and current and potential future management options.

## Materials and methods

### Study design

We conducted a mixed-method study involving qualitative focus groups and brief quantitative questionnaires. Our study built on qualitative work done in preparation for the LORIS DCIS monitoring trial [21], and was conducted with a larger Australian sample several years later in a context where the state of the science in relation to the management of DCIS had moved forward substantially and three international clinical trials, including the LORIS trial, had commenced [12–14]. Brief written questionnaires were administered at the start and end of each focus group. The study was approved by the University of Sydney Human Research Ethics Committee (2018/060). Participants gave written informed consent prior to participating.

### Participant recruitment

We recruited a community sample of women across metropolitan Sydney, Australia. Women were aged 50–74 years, the target age range for population-based breast screening in Australia. An independent research recruitment organisation (Taverner Research) used random landline and location known mobile samples from Sydney to contact potential participants by phone. Trained interviewers introduced the study as a ‘focus group about women’s health’ and used a series of questions to assess eligibility, excluding women with a personal history of breast cancer or DCIS and those not fluent in English as interpreting services were unavailable for this study. Interested participants were then mailed/emailed the Participant Information Statement and booked into a focus group time. To gain a diverse range of perspectives, quotas were used to ensure inclusion of participants with and without post-secondary school education, across three age groups (50–59, 60–69 and 70–74 years). Participants received a $100 retail gift voucher as compensation for their time and any costs incurred.

We held six focus groups (two per age group) in May 2018 at two Sydney locations which were convenient to attend and captured different socio-economic regions. We recruited 10 participants per group to optimise group dynamics. This number of participants enabled us to reach thematic data saturation, as indicated by data redundancy i.e., when participants no longer raised original themes [22,23].

### Focus group presentation and discussion

Details of the development of the focus group presentation are outlined in S1 Appendix, and the presentation slides are provided in S2 Appendix. Box 1 outlines the presentation topics and key discussion questions. The focus groups were moderated by two female public health researchers trained in qualitative methods, with experience conducting focus groups.

**Box 1. DCIS focus group presentation topics and key discussion questions**

**Table:** 

Presentation content	Corresponding questions for discussion
**1. Breast cancer screening** • What breast screening is and how it is done	1a. Have you had a mammogram before?
2. **What is DCIS?** • What DCIS is • How DCIS differs from invasive cancer • What happens after DCIS is diagnosed	2a. Have you ever heard of DCIS before? 2b. Do you feel you understand what I’ve said so far? Is anything unclear or confusing about it? Any questions? 2c. Have you ever heard the idea that some people are diagnosed through screening, and have treatment, for something that may not have ever become life-threatening? 2d. What are your thoughts and feelings about DCIS, from what I’ve said so far?
**3. How is DCIS treated?** • Breast conserving surgery + radiotherapy • Mastectomy • Common side effects of each treatment	3a. What are your thoughts and feelings about that information about treatment for DCIS? 3b. Hypothetically, if you had just been diagnosed with DCIS and told about these standard treatment options, how do you think you might feel?
**4. Could some women be spared treatment?** • Treating DCIS as women get older^a^ • Efforts to identify low-risk DCIS • DCIS Grade and its relevance	4a. What are your thoughts and feelings about DCIS in older women in particular?^a^
5. **Clinical trials** • What a clinical trial is • The ongoing international clinical trials of monitoring in low-risk DCIS	5a. What are your thoughts and feelings about these clinical trials? 5b. Imagine if you had been diagnosed with low-risk DCIS and invited to be in a trial like this, how do you think you would feel about whether to join the research?

^a^Brief additional content for only the two focus groups with women aged 70–74 years.

### Data collection and analysis

Focus group discussions were audio-recorded and transcribed verbatim. Transcripts were thematically analysed to identify recurring themes and data patterns [24], using NVivo software. Two researchers (BN and JH) independently reviewed notes taken during the focus groups and one researcher (BN) read all of the transcripts. Analysis initially took an inductive approach where there were no set or pre-determined hypotheses to ensure the findings were grounded in participant responses, and data were analysed based on the explicit meaning of what participants said (i.e. semantic level [24]). Using constant comparison [22,25], the two researchers (BN and JH) discussed similarities and differences in the data and developed the coding framework. Coding was performed by one researcher (BN), with JH checking a sub-sample of the data to ensure coding was consistent with the framework. The final coding was then examined by two researchers (BN and AD) and discussed with JH to identify overarching themes and concepts.

Questionnaire 1 (start of session) included demographics, breast cancer worry (*not worried at all*, *a bit worried*, *quite worried*, *very worried*), breast screening history (*never been screened*, *once*, *twice*, *three or more times)* and future intentions (*definitely will*, *likely*, *unsure*, *not likely*, *definitely will not*). Questionnaire 2 (end of session) assessed conceptual understanding of DCIS using a free-text item (defining DCIS) and four true/false questions adapted from previous research [26], hypothetical intentions towards joining a DCIS monitoring trial (multiple choice) together with reasons (free text), and shared decision making preferences [27]. Free-text responses for women’s definitions of DCIS were scored as 0, 1, or 2 according to how many of the following two points they indicated correctly: (i) that DCIS represents a change within cells confined to the breast milk ducts, and (ii) the uncertain potential for DCIS to progress to invasive cancer. Responses regarding interest in joining a DCIS clinical trial were summarised descriptively, and free-text responses about the corresponding reasons were incorporated into the thematic analysis.

## Results

Fifty-six women from diverse socioeconomic backgrounds took part in six focus groups (Table 1).

**Table 1:** Participant characteristics (n = 56).

Characteristic	Frequency n (%)
**Age (years)** 50–59 60–69 70–74	19 (33.9) 18 (32.1) 19 (33.9)
**Education** University undergraduate degree or above Diploma, certificate or apprenticeship Higher school certificate or equivalent School certificate or intermediate certificate (or equivalent) No school or other qualifications	17 (30.4) 16 (28.6) 7 (12.5) 15 (26.8) 1 (1.8)
**Marital Status** Married/living with partner Divorced/separated Widowed Single	37 (66.1) 8 (14.3) 4 (7.1) 7 (12.5)
**Employment** Full time Part time Retired Other	8 (14.3) 13 (23.2) 28 (50.0) 7 (12.5)
**Place of birth** Australia Other	33 (58.9) 23 (41.1)
**Self-rated general health** Excellent Very good Good Fair Poor	12 (21.4) 16 (28.6) 19 (33.9) 8 (14.3) 1 (1.8)
**Breast cancer worry**^a^ Not worried at all A bit worried Quite worried Very worried	27 (48.2) 25 (44.6) 4 (7.1) 0 (0.0)
**Previous breast screening history** Never been screened Once Twice Three or more times	6 (10.7) 6 (10.7) 8 (14.3) 36 (64.3)
**Future breast screening intention** Definitely will Likely Unsure Not likely Definitely will not	40 (71.4) 4 (7.1) 7 (12.5) 3 (5.4) 2 (3.6)

^a^A validated single item that measures level of worry about developing breast cancer, using four response categories ranging from *not worried at all to very worried*.

Thematic analysis identified four main themes and six sub-themes. Participant quotes are presented throughout to illustrate common and diverse responses. Differences observed between age groups are outlined where relevant.

### 1. Catch it early

There was little to no previous awareness of DCIS. Only two women had heard the term before, but neither had really understood what it was.

Following the presentation about what DCIS is and its link to screening, initial reactions were overall very positive towards early detection. Women expressed the view that DCIS, like cancer generally, needs to be ‘caught early’ and acted upon quickly.

*“I think it’s good that screening will actually find it… You can be screened for it and find it and act upon it”* (FG2 50–59)

Some women further discussed that the initial information made them want to be more proactive with regular screening, on the basis that finding DCIS would allow one to prevent breast cancer.

*“…it’s the thought of having breast cancer but now it gives you more hope that if you have this… it’s very important and all that it doesn’t have to be cancer but it can be prevented*.*”* (FG1 50–59)

### 2. Treatment provides a ‘cure’… with consequences

Reponses towards surgical treatments (lumpectomy and mastectomy) for DCIS varied. Some women suggested they would feel more optimistic if they were diagnosed with DCIS compared to invasive breast cancer, and more confident that surgery would be effective.

“…*if you have the DCIS there are treatments*, *you know*, *and I think that… it gives you some hope… If there’s some sort of treatment*, *there’s hope*.*”* (FG3 70–74)

Other women however, had very negative responses to surgical treatment for DCIS in terms of risks and impact on quality of life. Often, they indicated some familiarity with these treatments from experiences of family, friends or acquaintances who had undergone them.

*“Oh*, *it can be quite devastating*. *It changes your quality of life… being detected*, *having an operation*, *all the possibilities that could go wrong*.*”* (FG4 60–69)

Of the women who felt more positive about surgery, most suggested lumpectomy was their preferred treatment as it was far less invasive than mastectomy.

*“I’m a bit nervous about something as drastic as a mastectomy*. *So lumpectomy sounds a bit more like the way I’d go”* (FG3 70–74)

A few women however, expressed preference for more invasive treatment based on their previous experiences and wanting to ensure the best outcome.

*“I’d go for the full mastectomy*. *Only knowing what I know*. *Because that’s… my daughter was diagnosed and once they did the mastectomy they found a rogue cell*.*”* (FG6 70–74)

One of the older women’s focus groups was also more positive about mastectomy, with a couple of women sharing stories of friends and acquaintances who had undergone mastectomy and reconstruction, with positive outcomes.

*“…there’s a lady in our swimming group who has had a double mastectomy and she’s had the reconstruction and… they look great*.*”* (FG3 70–74)

In terms of radiotherapy, women again discussed negative side-effects they’d encountered in people they knew.

#### 2a. Treatment decision making is complex

A common sentiment was that with DCIS, compared to invasive breast cancer, there was more time to get second opinions and consider the options. Many women thought they would seek out several opinions from medical professionals and compare notes.

*“I guess the advantage of the DCIS diagnosis though*, *you have got time to actually to consider*, *explore a couple of other alternatives*, *specialists or whatever*, *and get a few opinions before you went straight into surgery … it’s not like a life-threatening thing tomorrow*.*”* (FG5 60–69)

Overall, women discussed wanting information on surgical and non-surgical options presented to them so they could make a decision. Although, there was a slight divide between those whose approach would be to just accept their medical professional’s recommendations and those who would take on their clinician’s advice but additionally search for their own information to make a final decision themselves. Underlying this was uniform acknowledgement that decision making around treating DCIS is very personal and ultimately the patient’s choice to make, but could be influenced by medical professionals.

*“…certainly the medical person with whom you interact that gives you the first information can make or break where you go from there*.*”* (FG5 60–69)

Factors that women identified as influential in their hypothetical decision making for DCIS treatment were their relationship with providers (general practitioner and/or specialist), support from family and friends, personal or family history of DCIS or breast cancer, access to information and whether one is proactive in terms of sourcing and digesting information. Further influences included one’s financial situation, co-morbidities, and psychosocial factors including anxiety.

### 3. Is it safe to avoid surgery for low-risk DCIS?

#### 3a. Is surgery really needed?

A few women spontaneously brought up the idea of not having immediate surgical treatment early on during their focus group, before it was discussed or prompted by the moderator. This occurred particularly in the 70–74 year groups and related to the possibility that DCIS may not progress or may take many years to do so.

*“I mean if it could take years to develop and if you have regular screening rather than rushing into surgery… I think [not having surgery] would be a good option*.*”* (FG3 70–74)

#### 3b. A decision to be made

When specifically presented with information on low-risk DCIS and the idea that some women could avoid immediate surgery, women initially discussed wanting further clarification on potential risks of active monitoring. In particular they wanted to know statistics associated with adverse outcomes and survival. After additional information about the evidence base and explaining the uncertainty around it, a number of women remained focused on whether doctors could predict reliably whether the DCIS would spread.

*“I’d want to know*, *what chance there is that maybe you made the wrong decision and you should have just opted for the surgery*? *Like what risk is there with going down that path*?*”* (FG1 50–59)

Women also noted that DCIS could take years to possibly turn into invasive cancer, and some seemed to want to take a cautious approach and think about their options. The idea that it might be sensible to ‘watch and wait’ for future treatment options to become available was raised a few times.

*“The way I look at treatment too*, *the way science is progressing*, *if I was diagnosed with DCIS I would tend to watch it*, *with the doctor’s advice because you never know what science will come up with in the future*. *And it might be a much better way to treat it*. *And less invasive*.*”* (FG3 70–74)

Age was also specifically discussed in the 70–74 year groups in relation to their preference for the possibility of avoiding surgery. Women believed that if they were older (than they currently were), then maybe they would not worry as much about it.

*I think if I was diagnosed and I’m in my 80’s*, *maybe*, *I don’t think I would want to have any treatment*. *Hopefully it would just stay as it is and not affect me for the rest of my life*. (FG6 70–74)

### 4. Willingness to accept active monitoring and whether to join a clinical trial

Generally, women viewed active monitoring as a reasonable potential management option for low-risk DCIS, and there was substantial interest in joining a hypothetical clinical trial assessing that approach. In considering such a trial, women wanted clarification around how often they would be monitored and whether they could still get surgery later, if needed. The idea of monitoring regularly and picking up any changes in a timely manner, to check if they then required surgery, was important.

*“if it becomes aggressive… and it’s still monitored as far as regular mammograms maybe even twice a year… and that it turns into breast cancer*, *you then still have that option of the treatment at a very early stage of breast cancer*, *don’t you*?*”* (FG3 70–74)

However, some women also saw active monitoring (and therefore participation in such a trial) as a risk.

*“I think you have to be pretty brave to go into a trial*, *quite honestly*.. *because there’s still a risk… if survival was your biggest criteria*, *which I think for most of us it is… you probably want to be treated immediately*. *To get rid of the cancer*.*”* (FG6 70–74)

#### 4a. Reasons to join a trial

Generally, most women felt that monitoring provides a good safety net for those that receive no treatment and was a valid alternative to consider. It was viewed as the least traumatic option, avoiding potential side-effects, and one that simultaneously contributes to medical advances in the field.

*“Way I see it the odds are in your favour*. *Because if you can avoid the surgery*, *which not everybody can recuperate that well… in the meantime you’re helping science to come up with an easier cure for our kids*.*”* (FG2, 50–59)

Women felt that monitoring in a trial minimised risk and was the ‘best of both worlds’ as they would be offered treatment if clinically indicated and could withdraw at any time if they were unhappy or changed their mind.

*“But while you’re still being monitored*, *if your condition becomes worse… then they know about it*. *Something can be done*. *Sounds like the best of all worlds really*.*”* (FG6 70–74)

Women emphasised the importance of regular surveillance. A few women who were a bit unsure said they would want more frequent scans to feel comfortable with this option.

“*I just feel 12 months [between mammograms] might be a bit too long*.*”* (FG6 70–74)

Some older women discussed being more comfortable with the idea of monitoring at their age, due to their life expectancy and changed social roles.

*“Maybe not when I was younger*, *when you have dependent children… I wouldn’t be taking any chances at all*. *But at my age I think that would be fine*.*”* (FG3 70–74)

#### 4b. Reasons to decline a trial

The principal reason women had reservations about joining a trial was because they objected to being randomised. Often women said they did not want to necessarily have surgery and therefore did not want to be randomised into the treatment arm.

*“I think the bit that I was struggling with is if*, *if [monitoring] is the path I wanted to go then that decision’s been taken away from me and forcing me to have actually invasive treatment…”* (FG2 50–59)

Some women however, discussed declining the trial because they would want to have surgery as soon as possible and not risk being randomised to monitoring. These women discussed wanting to be proactive about their health, feeling a sense of responsibility towards their family, and the potential psychological burden of always wondering whether the cancer cells are progressing.

*“But you’ve got the psychological worry the whole time… at the back of your mind there’s this little person on your shoulder going*, *you’ve got cancer cells in there*.*”* (FG5 60–69)

#### 4c. Many deciding factors however, you don’t really know until it’s real

Multiple factors would influence women’s preferences for active monitoring and decisions about joining a clinical trial. Table 2 summarised factors discussed during the focus groups and/or noted in the final questionnaire. Uniformly women voiced that as this was a hypothetical decision, they could not accurately predict how they would feel and behave in that situation.

**Table 2:** Factors that would influence women’s willingness to accept active monitoring for DCIS, and joining a clinical trial.

Factors	Explanation	Supporting quotes
Perceptions about widespread access to monitoring outside of a trial setting	Women believed that all medical professionals would discuss this option and that they would make a choice with them in deciding about how appropriate monitoring would be for the individual patient	*“This is a low-grade and it’s not terminal*, *I don’t think I would go for it*. *I think I would prefer to just go and get monitored individually through a general practitioner*.*”* (FG3 70–74) *“If you were diagnosed with a low-grade DCIS*, *you could then make your choice*, *because I’m sure that your treating doctor would follow those sorts of guidelines and do regular testing to determine whether the grade of your cancer increased or not and then you could make the decision based on that information*.*”* (FG5 60–69)
Trial design, process and information	• Randomisation–not being able to choose was unappealing to many • Women would need to feel well supported and confident that they were being carefully monitored • Ongoing impartial information about the trial would be valued	*“It’s just… in the trial you don’t have the choice*. *You don’t have an option*. *You just go into either treatment or active monitoring*.*”* (FG1 50–59)*“And all strict check-ups*. *You know*, *you’ve got to be really diligent with that and have the support*.*”* (FG4 60–69)*“…information is just absolutely important*. *And the person has to feel that… they’ve been given as much information as possible*. • *And unbiased*. • *Unbiased*. • *So you’re not being pushed into a trial because they need a certain number of people to do it*.*”* (FG4 60–69)
Personal factors	• Physical factors e.g. age, co-morbidities • Psychological factors e.g. anxiety • Lifestyle factors e.g. location (urban vs rural)	*“…because I have a heart condition*, *it might dissuade me not to have the treatment ‘cause of that*, *rather than having radiotherapy because of the heart*. *So I think health for me definitely would be a factor”* (FG4 60–69) *“I think it’s a lot to do with your own mental strength as well” (FG3 70–74)* *“Only thing that might be an influencing factor is where you actually live*. *Living in Sydney or a major metropolitan area*, *access to all those essential type services are literally at your fingertips*. *But if you start getting out to the more remote areas*, *it might change your mind… and actually opt for the treatment because*, *you know*, *it’s done with*, *whatever*. *Not traipsing backwards and forwards at potentially vast distances*.*”* (FG1 50–59)
Support	• Family, friends, medical professionals	*“… it would depend on the support you get from your family*, *or husband and whether they’re encouraging you to do what you want to do*, *not pushing their point… (FG3 70–74)*
Clinician recommendations	• Trust in medical professionals to influence their decision making	*“If doctor think*, *you know*, *I need to do the trial I think I would go for it*.*”* (FG1 50–59) *“…if the doctor tells you it might never [progress]*, *you’d go for the trial*. *I would*.*”* (FG6 70–74)

### 5. Final questionnaire

Participants largely understood the key concepts presented about DCIS (Table 3), with a mean score of 2.97 out of a possible 4 marks. The mean score for free-text definitions of DCIS was 1.13 out of a possible 2 marks (Table 4).

**Table 3:** Participant responses to questions assessing concepts surrounding DCIS (n = 56).

Knowledge item	Frequency n (%)
All DCIS will eventually cause illness and death if it is not found and treated True False (correct answer) Unsure	6 (10.7) 42 (75) 8 (14.3)
When screening finds DCIS, doctors can reliably predict whether it will ever cause harm True False (correct answer) Unsure	11 (19.6) 39 (69.6) 6 (10.7)
Even DCIS that may not cause any health problems is likely to be treated True (correct answer) False Unsure	47 (83.9) 5 (8.9) 4 (7.1)
Screening leads some women with harmless DCIS to get treatment they do not need^a^ True (correct answer) False Unsure	36 (64.3) 13 (23.2) 6 (10.7)

^**a**^Data missing for one participant.

**Table 4:** Scores for participant free-text definitions of DCIS, from written questionnaire completed at the conclusion of the focus group session (n = 56).

Marks (out of 2)	Example definitions	Frequency n (%)
2 marks	*“Abnormal cells contained in a milk duct that could stay as they are or could turn into invasive cancer at a later date*.*”* (FG1, age 59)	16 (28.6)
1 mark	*“It is the presence of cancerous cells within the duct/s of the breast*.*”* (FG2, age 50) *“A type of breast condition that could or could not become cancer*.*”* (FG5, age 69)	31 (55.3)
0 marks	*“Pre cancer contained in the breast*.*”* (FG3, age 71)	9 (16.1)

The majority of participants indicated that they would be willing to join a clinical trial (53.6%) and want to take an active role in this decision (89.3%) (Table 5).

**Table 5:** Responses to hypothetical scenario about joining a clinical trial, from written questionnaire completed at the conclusion of the focus group session^a^.

Response	Frequency n (%)
Join Do not join Impossible to say	30 (53.6) 13 (23.2) 12 (21.4)
I prefer to make the decision myself about whether to join the clinical trial I prefer to make the final decision about whether to join the clinical trial myself after seriously considering my doctor’s opinion I prefer that my doctor and I share equally in deciding whether I join the clinical trial I prefer my doctor to make the final decision about whether I join the clinical trial after seriously considering my opinion I prefer to leave the decision about whether I join the clinical trial to my doctor^b^	12 (21.4) 27 (48.2) 11 (19.6) 2 (3.6) 0 (0.0)

^a^Questions not answered by all 56 participants.

^b^All response options adapted from the Control Preferences Scale [27].

## Discussion

This mixed-method study in a sample of Australian women of breast screening age found that community awareness of DCIS remains very limited, despite women having high levels of screening participation. Study participants developed reasonable understanding of DCIS and the issues surrounding its management, as demonstrated in both the focus group discussions and the final questionnaire. Overall, women expressed substantial support for active monitoring being offered as a management approach for low-risk DCIS, although there were mixed views about being personally willing to accept active monitoring. While discussions did demonstrate overarching support for the ongoing trials of monitoring for low-risk DCIS, and potential willingness to participate if hypothetically invited, women noted a number of important questions and factors they would consider in their decision making process.

Our findings are the first to specifically highlight the limited awareness of DCIS in Australia, and studies in similar cohorts in other countries with population-based screening programs have also demonstrated limited awareness and knowledge about DCIS [21,28,29]. As DCIS is much more common today than in the past, due to breast cancer screening, it is surprising that there continues to be minimal communication to women about the condition. Our study demonstrated the same highly positive attitudes and widespread enthusiasm towards cancer screening and early detection as previously documented [30,31]. It has also been documented that there is a strong bias toward more invasive treatments in cancer [32]. Together, these views make the idea of monitoring as a management option for DCIS quite counter-intuitive. However, as evident in our study the option of active monitoring can be discussed and may be preferred by many or at least some women who view it as a deliberate or positive action [33]. Women understood and were generally accepting of active monitoring being an option for low-risk DCIS, in particular due to the low-risk nature of the condition and the ongoing monitoring to pick up any changes. Parallel views have been found in recent studies on DCIS and low-risk thyroid cancer where active monitoring was being discussed as a potential management option [15,34]. Similarly, there is evidence of increasing support for and use of active surveillance as management for low-risk prostate cancer [35,36].

Participants acknowledged that they were making a hypothetical decision in relation to joining or not joining a clinical trial, but raised various personal, lifestyle, and clinical factors that influenced their thinking. This further demonstrates the complexity of such trials and the importance of appropriate information provision [37]. Recruitment into clinical trials has been slower than expected [15], and although our study found overarching willingness to consider a DCIS monitoring trial it provides further insight into the complex decision making process for women and the range of factors that may be considered. Interestingly, women in our study believed that clinicians would guide them in considering whether or not active monitoring was an acceptable management option for low-risk DCIS. Our previous work however, demonstrates that clinicians involved in managing DCIS in Australia and New Zealand generally felt uncomfortable recommending active monitoring outside an ethically approved clinical trial and viewed it as outside of current standard care [20]. As it will still be some years before the relevant results are published and new evidence potentially incorporated into clinical guidelines, our study suggests that in the meantime discussions around less invasive management options, particularly for older women, would be welcomed by the community.

### Strengths and limitations

This is the first study to investigate and report in-depth the views expressed by a sizeable sample of screening-aged women about the current management of DCIS and their views about active monitoring for low-risk DCIS. We recruited a diverse sample of women varying in educational background. However, it is important to note that excluding non-English speaking women means our findings may not reflect the views of such women. The presentation was evidence-based, developed by a multidisciplinary expert team including a consumer representative, and rigorously pilot-tested. The main limitation of the study is that women were considering a hypothetical diagnosis and giving immediate responses to complex new information. To avoid overloading participants with information, we did not present numeric estimates of the risk of missing invasive cancer on biopsy or the chance of progression of untreated DCIS, although we did raise these important issues. Adding further complexity to the information presented might have influenced women’s views but would have limited our capacity to address the key research questions. Conducting face-to-face focus groups allowed for clarification of the topic through both queries to the facilitator and discussion amongst the participants [38]. Furthermore, comprehension was assessed in the final questionnaire, which indicated that most participants gained a reasonable understanding of key concepts. We acknowledge that invitation to a DCIS monitoring trial is an unlikely prospect for women in Australia in the near future. Nonetheless, it was appropriate to frame our discussions about monitoring by situating it within a trial scenario because that is the only context where clinicians would currently be comfortable offering it [20].

## Conclusions

We found that public awareness of DCIS remains very low, but women developed reasonable understanding of DCIS following a short informational presentation. There was substantial support for offering the option of de-escalated treatment for low-risk DCIS, especially among older women. The perceptions of women in this focus group study continue to be timely while clinicians and women await the results of clinical trials, and are informative for investigators designing future, similar trials. If clinical trials are to generate much-needed high-quality evidence supporting the safety of new management approaches, effective communication with participants is essential. Further, our findings suggest that exploration of offering well-informed patients the choice of non-surgical treatment of low-risk DCIS, even outside the setting of clinical trials, may already be warranted. Findings from this study provide new evidence and support existing evidence on preferences and factors relevant for decision making around management for low-risk DCIS to help guide clinicians in this process.

## Supporting information

S1 Appendix Further details on the focus group presentation and discussion. (PDF)

S2 Appendix Presentation slides. (PDF)

S3 Appendix Focus group transcripts. (PDF)

S4 Appendix Questionnaire data. (PDF)
