# Supplementary material for: Women’s views about current and future management of Ductal Carcinoma in Situ (DCIS): A mixed-methods study
Source: PLoS One. 2023 Jul 21;18(7):e0288972. doi: 10.1371/journal.pone.0288972 (PMC10361483; doi:10.1371/journal.pone.0288972)
Supplement: S1 Appendix — (PDF) [file pone.0288972.s001.pdf]

S1: Further details on the focus group presentation and discussion

Each focus group session comprised consent, introductions, a brief written questionnaire (sociodemographics), a detailed live PowerPoint presentation interspersed with periods of discussion, and a final brief written questionnaire. Sessions lasted approximately two hours and were facilitated by JH with assistance from BN, who also took notes. The presentation was developed and reviewed by a multidisciplinary team of public health and medical researchers, breast clinicians, psychologists, and a consumer representative. One additional slide about "Treating DCIS as women get older", developed with the team expert on ageing and decision making, was included in the presentation for the women aged 70-74 years. We elicited feedback on the draft slides from three independent experts not involved in the project: a breast physician, surgical oncologist, and radiation oncologist. We held three pilot focus groups with women across our target age range, including an initial convenience sample plus women from the broader community. The final presentation included plain language, pictures and infographics including simple pictograms and graphs appropriate for diverse numeracy levels [1]. We presented a consumer-friendly explanation about DCIS and its diagnosis and treatment based on the latest evidence, and outlined the clinical trials on active monitoring for low-risk DCIS [2-5].

At the beginning of each focus group the moderators highlighted that they wanted to hear women's honest thoughts and opinions, and that we did not expect the group to come to any consensus. Moderators positioned themselves as neutral throughout and avoided characterising the views expressed by participants as 'right' or 'wrong'. Preferences for and against joining a clinical trial were discussed which allowed women to articulate their individual points of view and reflect on the views expressed by others [6]. Throughout the presentation, we encouraged participants to request clarification or ask questions to help them understand the material.

References:

1. Hawley ST, Zikmund-Fisher B, Ubel P, Jancovic A, Lucas T, Fagerlin A. The impact of the format of graphical presentation on health-related knowledge and treatment choices. *Patient Education and Counseling*. 2008;73(3):448-455.
2. Elshof LE, Tryfonidis K, Slaets L, et al. Feasibility of a prospective, randomised, open-label, international multicentre, phase III, non-inferiority trial to assess the safety of active surveillance for low risk ductal carcinoma in situ - The LORD study. *European Journal of Cancer*. 09 Jul 2015;51(12):1497-1510. doi:http://dx.doi.org/10.1016/j.ejca.2015.05.008
3. Hwang ES, Hyslop T, Lynch T, et al. The COMET (Comparison of Operative versus Monitoring and Endocrine Therapy) trial: a phase III randomised controlled clinical trial for low-risk ductal carcinoma in situ (DCIS). *BMJ open*. Mar 12 2019;9(3):e026797. doi:10.1136/bmjopen-2018-026797
4. Francis A, Thomas J, Fallowfield L, et al. Addressing overtreatment of screen detected DCIS; the LORIS trial. *European journal of cancer*. Nov 2015;51(16):2296-303. doi:10.1016/j.ejca.2015.07.017

5. Sagara Y, Mallory MA, Wong S, et al. Survival Benefit of Breast Surgery for Low-Grade Ductal Carcinoma In Situ: A Population-Based Cohort Study. *JAMA surgery*. Jun 3 2015;doi:10.1001/jamasurg.2015.0876
