# Supplementary material for: Women’s views about current and future management of Ductal Carcinoma in Situ (DCIS): A mixed-methods study
Source: PLoS One. 2023 Jul 21;18(7):e0288972. doi: 10.1371/journal.pone.0288972 (PMC10361483; doi:10.1371/journal.pone.0288972)
Supplement: S2 Appendix — (PDF) [file pone.0288972.s002.pdf]

Community Views about Management of DCIS*

*Ductal Carcinoma In Situ

The University of Sydney

Introduction

- Welcome and thanks
- Who we are
- Aims of the session
  - What we are going to do
  - Recording of discussions
  - Set-up / house-keeping
- Any questions from you

Complete Questionnaire 1

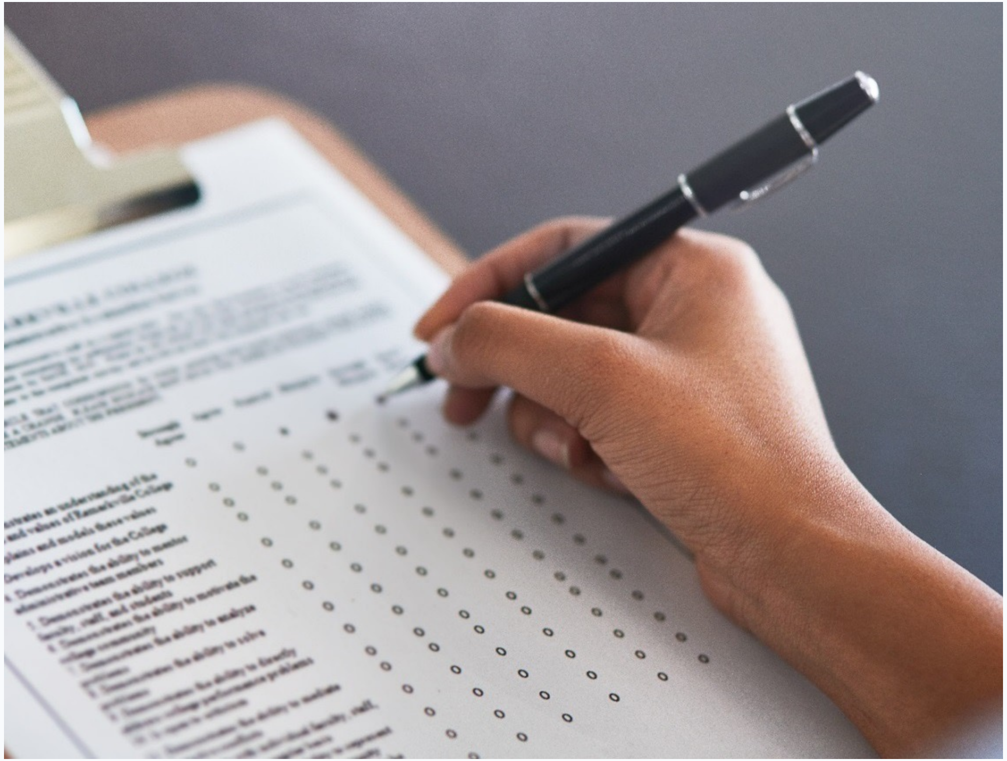

Community Views about Management of DCIS*

*Ductal Carcinoma In Situ

THE UNIVERSITY OF SYDNEY

What is breast cancer screening?

- Breast screening looks for early signs of breast cancer

Screening is for women without any breast symptoms (*e.g.* lump, pain, nipple discharge)

- Aim: to find women who have cancer so they're given treatment early, in the hope of a better outcome

- Main goal: to lower the number of women who die of breast cancer

How is breast screening done?

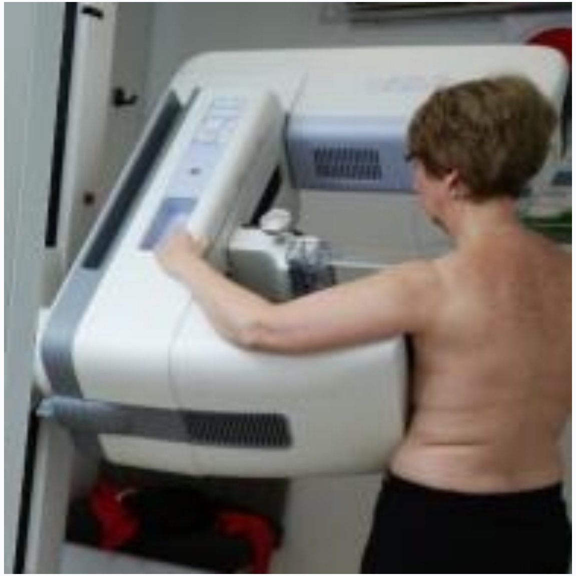

- A **mammogram** uses X-rays to make images of the breasts
- If the mammogram result looks abnormal, the woman may then have a **biopsy** to check what is going on
- This means taking a small sample of cells from the breast with a needle, to look at under a microscope

Ductal Carcinoma In Situ (DCIS)

- DCIS is a condition that can affect cells in the breast
- DCIS usually doesn’t cause symptoms and can’t be felt, so it’s generally only found through screening
- Before screening, DCIS was hardly ever diagnosed
- Now we have screening, DCIS is diagnosed more often than ever before

Number of women diagnosed by screening

Out of 1000 women who have screening for 25 years (starting when they are 50 years old)

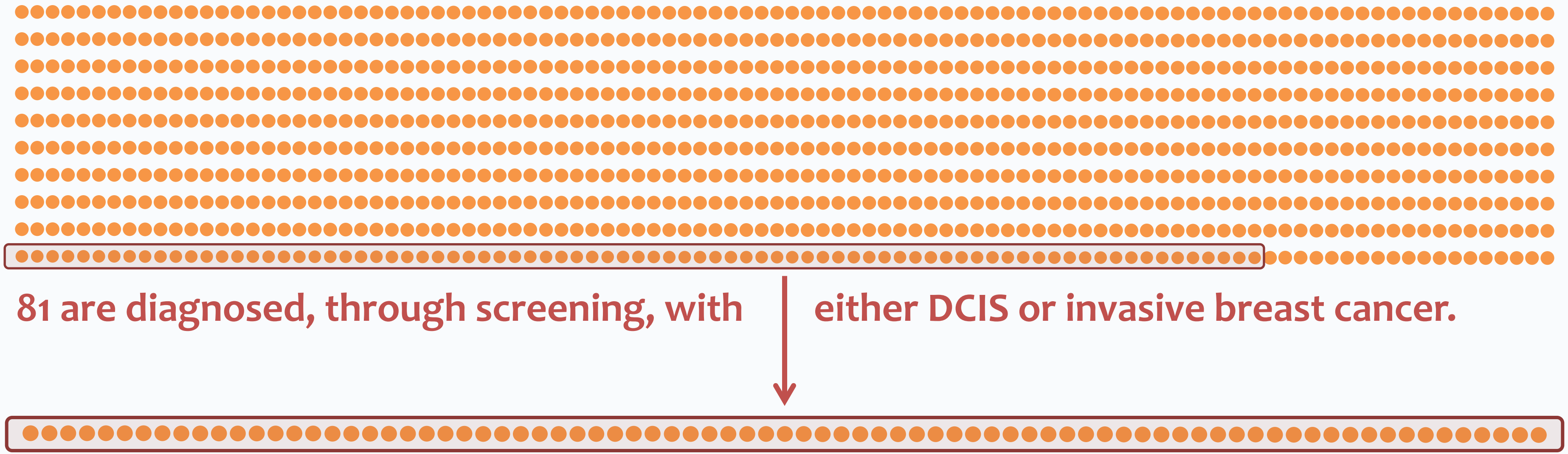

No. diagnosed with DCIS vs. invasive cancer

Out of 1000 women who have screening for 25 years (starting when they are 50 years old)

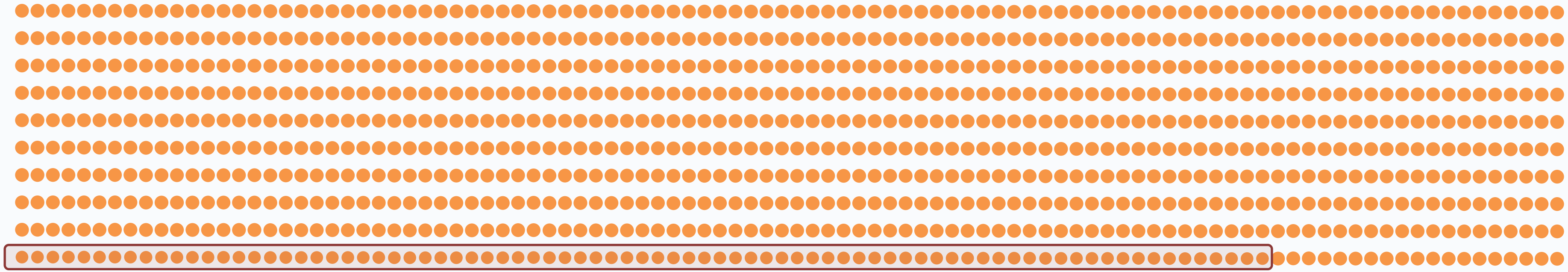

81 are diagnosed through screening.

Of these,

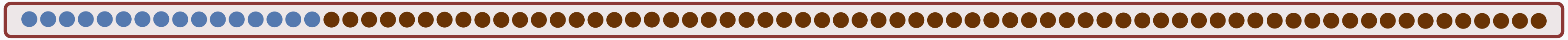

16 are diagnosed with DCIS and 65 are diagnosed with invasive breast cancer.

What is DCIS?

- **Ductal**: in milk duct (tube that carries milk to nipple)
- **Carcinoma**: cells show abnormal features similar to cancer
- **In Situ**: contained in the original place (has not spread outside lining of duct)

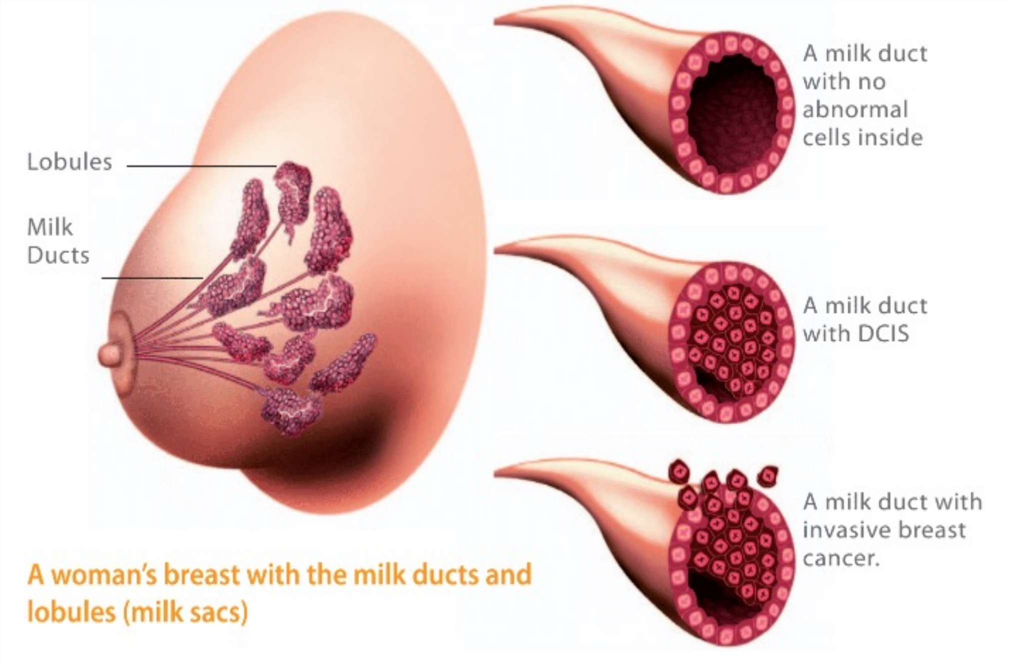

How DCIS differs from ‘invasive’ cancer

DCIS	Invasive breast cancer
Cells are inside milk duct and have not spread to breast	Cells have spread from milk duct to breast
Cells do not spread to other parts of body	Cells may spread from breast to other parts of body
Not life-threatening by itself, *but might develop into invasive breast cancer later on*	Potential to become life-threatening, if not treated

DCIS may be described in a variety of ways

- ‘abnormal changes in cells in the milk ducts’
- ‘cells that look like breast cancer… confined to the ducts’
- ‘some of the cells… have started to become cancer cells’
- ‘very early form of breast cancer’
- ‘Stage Zero breast cancer’
- ‘**non-invasive** breast cancer’
- ‘**pre-invasive** breast cancer’

What happens after DCIS is diagnosed?

- DCIS itself does not cause health problems
- But DCIS has the potential to turn into invasive cancer
- On the other hand, some cases of DCIS might never turn into invasive cancer (even without treatment)
- **Right now, doctors cannot tell for sure who will develop invasive cancer (and when) and who will not**
- So generally everyone is recommended some treatment

Time for discussion

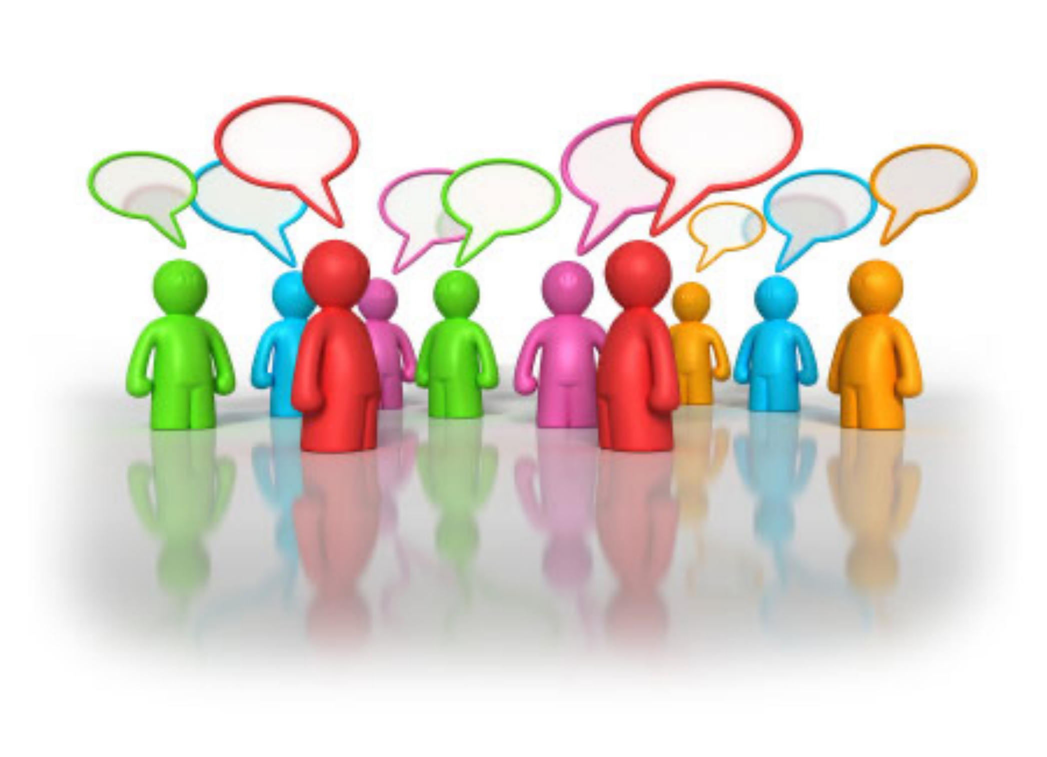

How is DCIS treated?

- Goal: to prevent the abnormal cells from turning into invasive breast cancer

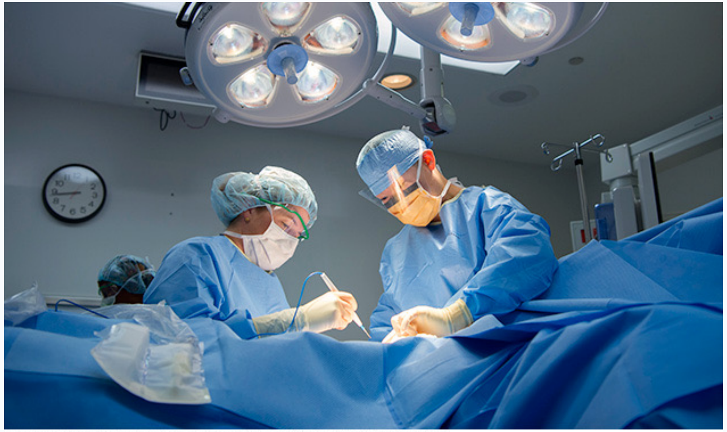

© MAYO FOUNDATION FOR MEDICAL EDUCATION AND RESEARCH. ALL RIGHTS RESERVED.

- Nearly all **DCIS patients have surgery**, which may be combined with another type of treatment
- Each patient's treatment depends on features of
  - the DCIS – *e.g.* size, grade (level of activity of cells)
  - the woman – *e.g.* age, overall health, preferences

1. Breast conserving surgery + *radiotherapy*

- DCIS is commonly treated by **breast conserving surgery** followed by radiotherapy
- Surgery **removes the area of DCIS** and some healthy tissue around it

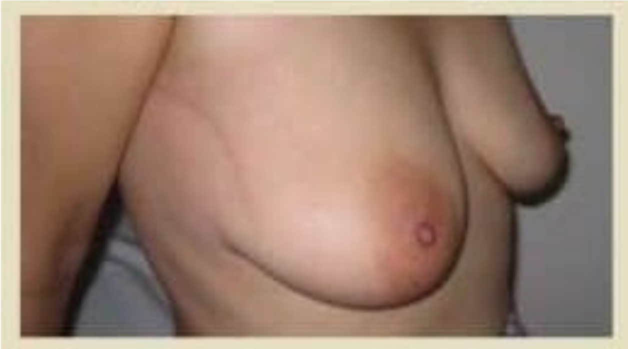

- May also be called lumpectomy or wide local excision
- Common side effects: pain, discomfort, numbness, bruising or swelling around the wound

1. *Breast conserving surgery + radiotherapy*

- After surgery, **radiotherapy** (radiation therapy) is given, usually daily for 3-6 weeks
- Radiotherapy uses X-rays to destroy any abnormal cells left in the breast
- Common side effects: tiredness, skin becoming red / dry / darker
- Radiation may slightly increase long-term chance of heart problems

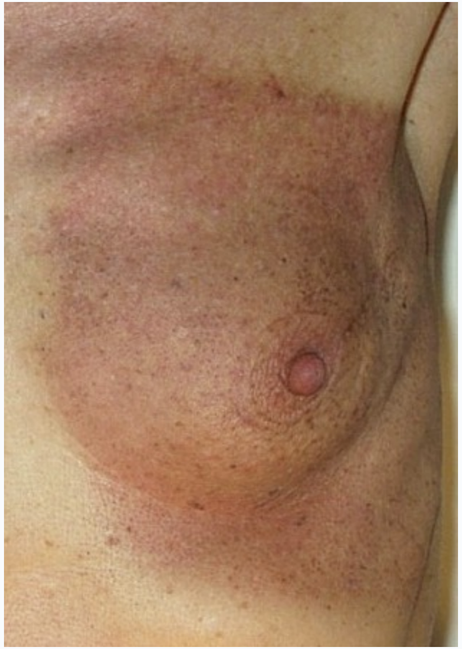

2. Mastectomy (removing whole breast)

- A **mastectomy** is surgery to **remove the whole breast**
- Bigger operation with higher chance of complications and longer recovery time
- Extra side effects, *e.g.* stiffness in arm or shoulder

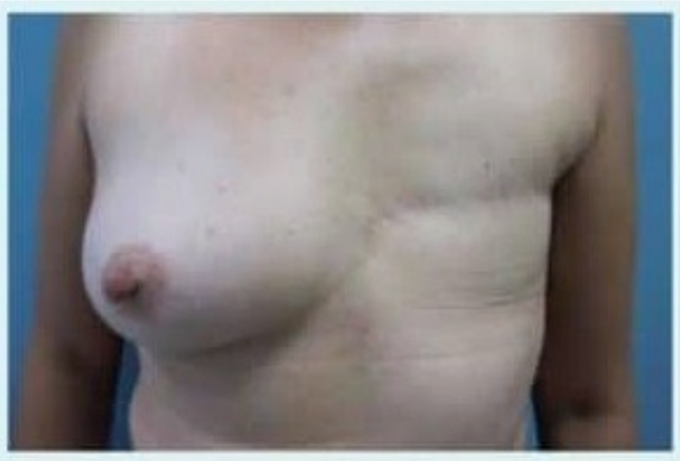

- Affects body shape and body image
- Women may have the option of breast reconstruction

Time for discussion

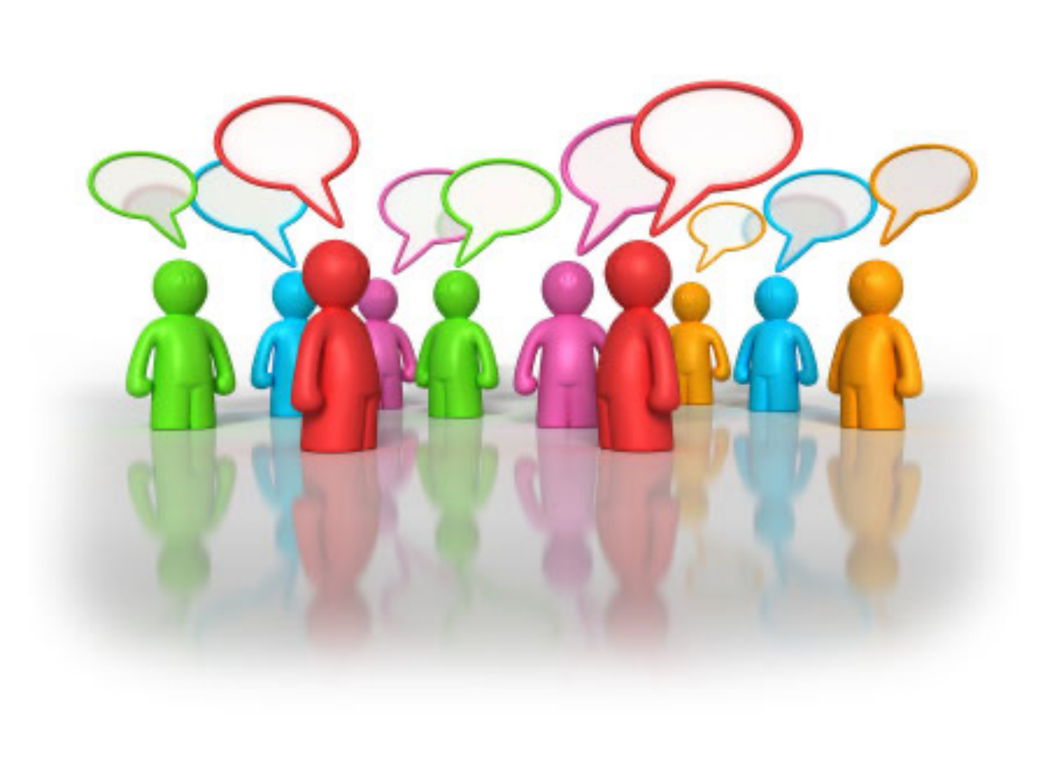

Could some women be spared treatment?

- Treatment for DCIS is based on current scientific knowledge and wanting to do what’s best for patients
- Many patients experience side effects from treatment
  - Effects on bodies and effects on feelings
  - Short-term and long-term effects

Treating DCIS as women get older

- The older women get, the more likely they are to have other conditions affecting their health
- DCIS may stay as it is or change slowly over many years. So in older women, DCIS may not affect their health in their remaining lifetime.
- Older women may get more treatment side effects
  - e.g. complications related to surgery
  - e.g. heart problems related to radiotherapy

Could some women be spared treatment?

- Treatment for DCIS is based on current scientific knowledge and wanting to do what’s best for patients
- Many patients experience side effects from treatment
  - Effects on bodies and effects on feelings
  - Short-term and long-term effects
- **Experts are working on ways to identify ‘lower risk’ patients who could avoid treatment and still do well**
- An important clue to identify lower risk DCIS is **grade**

Grade: how fast the DCIS cells may grow

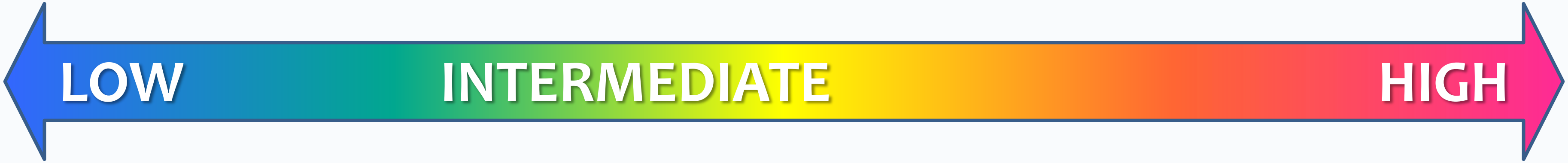

Low grade DCIS

Low cell activity

May remain as it is for many years

High grade DCIS

More active / faster growing

More likely to develop into invasive cancer if not treated

Survival in low grade DCIS without surgery

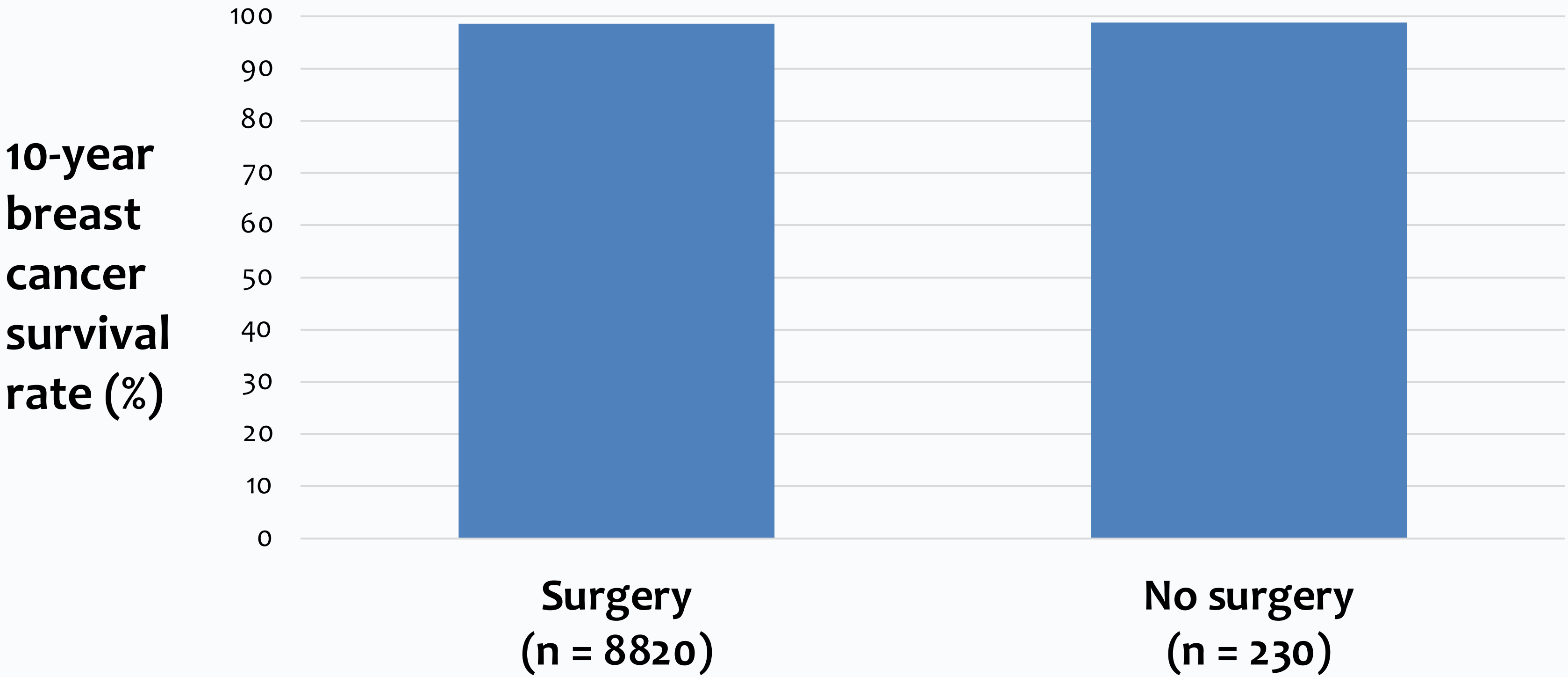

Grade: how fast the DCIS cells may grow

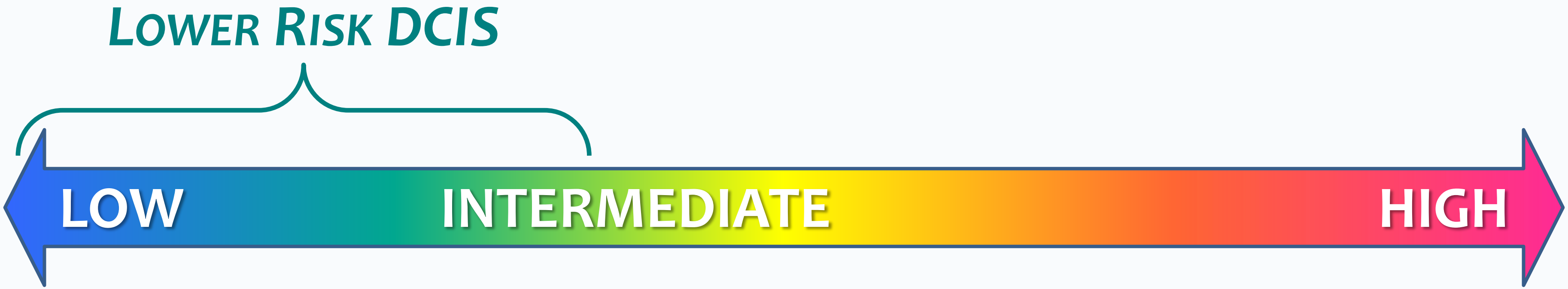

Low grade DCIS

Low cell activity

May remain as it is for many years

High grade DCIS

More active / faster growing

More likely to develop into invasive cancer if not treated

Out of 1000 women who have screening for 25 years (starting when they are 50 years old)

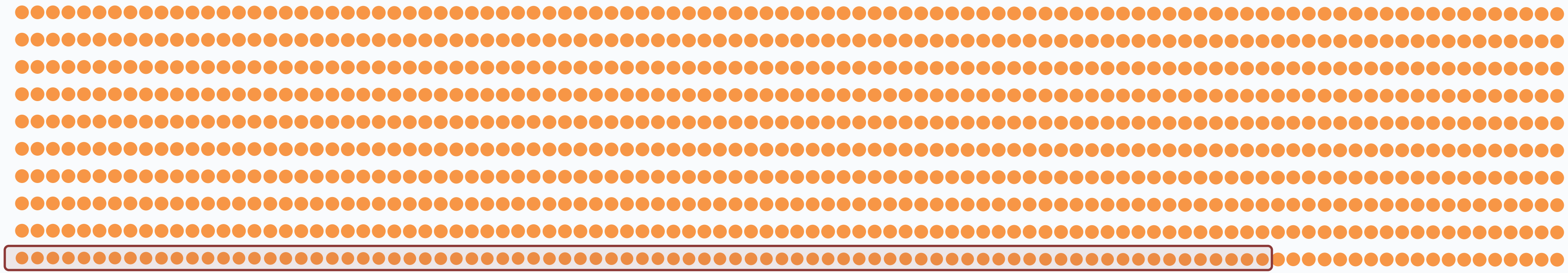

81 are diagnosed through screening.

Of these,

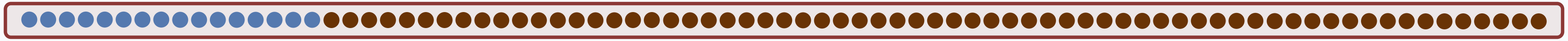

16 are diagnosed with DCIS

and 65 are diagnosed with invasive breast cancer.

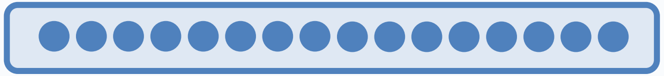

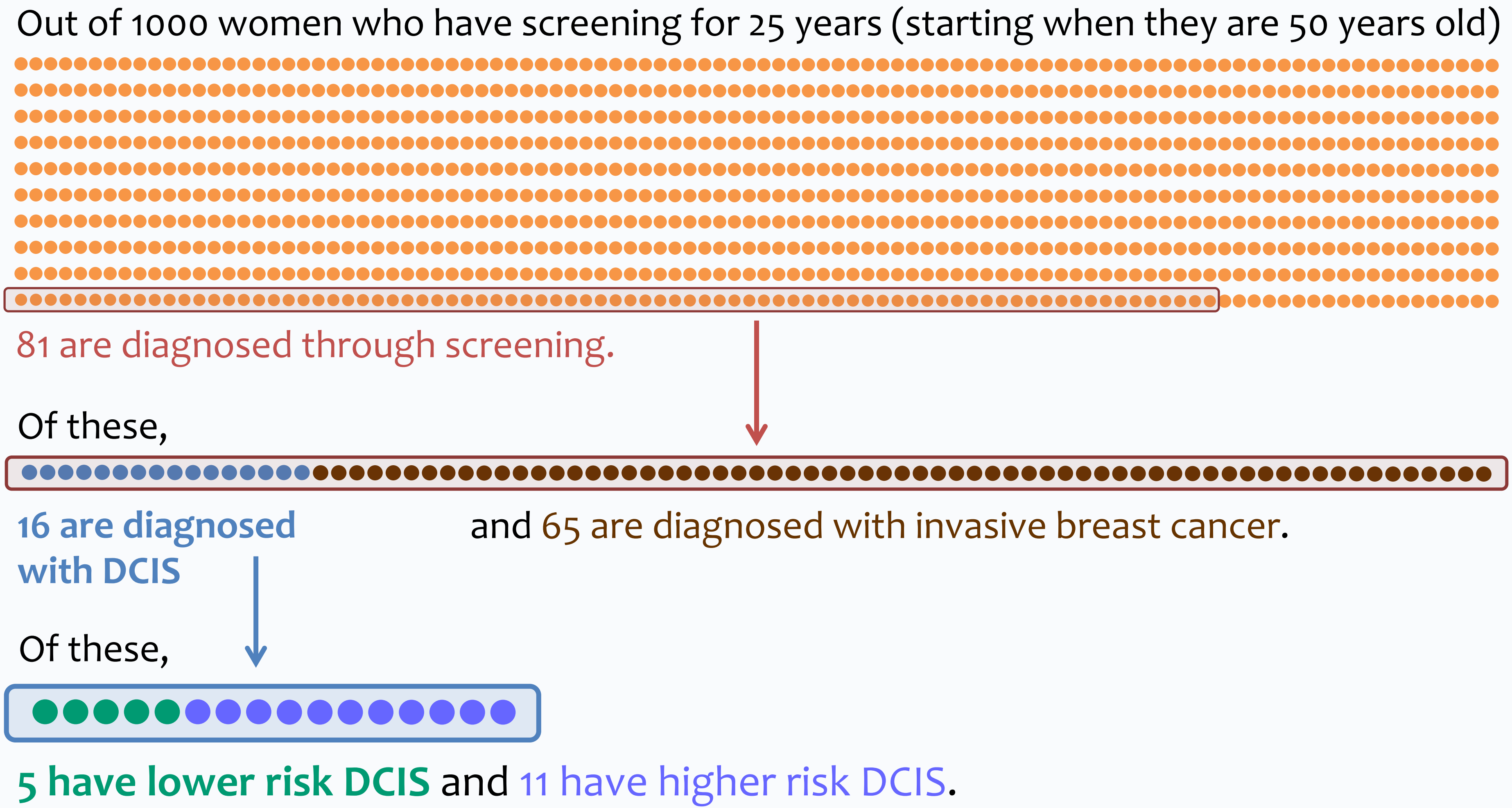
Out of 1000 women who have screening for 25 years (starting when they are 50 years old)
81 are diagnosed through screening.
Of these,
16 are diagnosed with DCIS
and 65 are diagnosed with invasive breast cancer.
Of these,
5 have lower risk DCIS and 11 have higher risk DCIS.

Clinical trials

- Research addressing an important, specific question about a certain health condition
- Often test new ways to manage a condition, by comparing a newer option with current standard care
- Designed to find out whether patient outcomes (health, quality of life) are similar for both options, or whether one option is better than the other
- Provide the highest quality of evidence

Randomisation in clinical trials

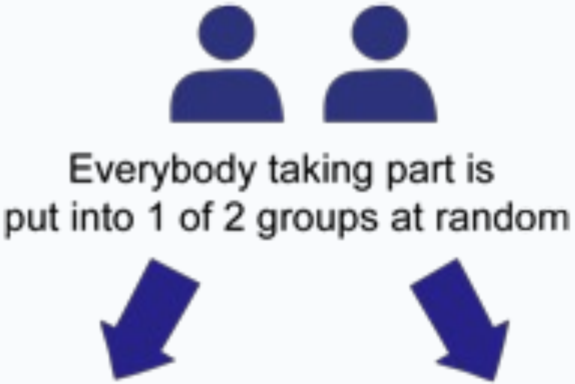

Usual treatment

New treatment

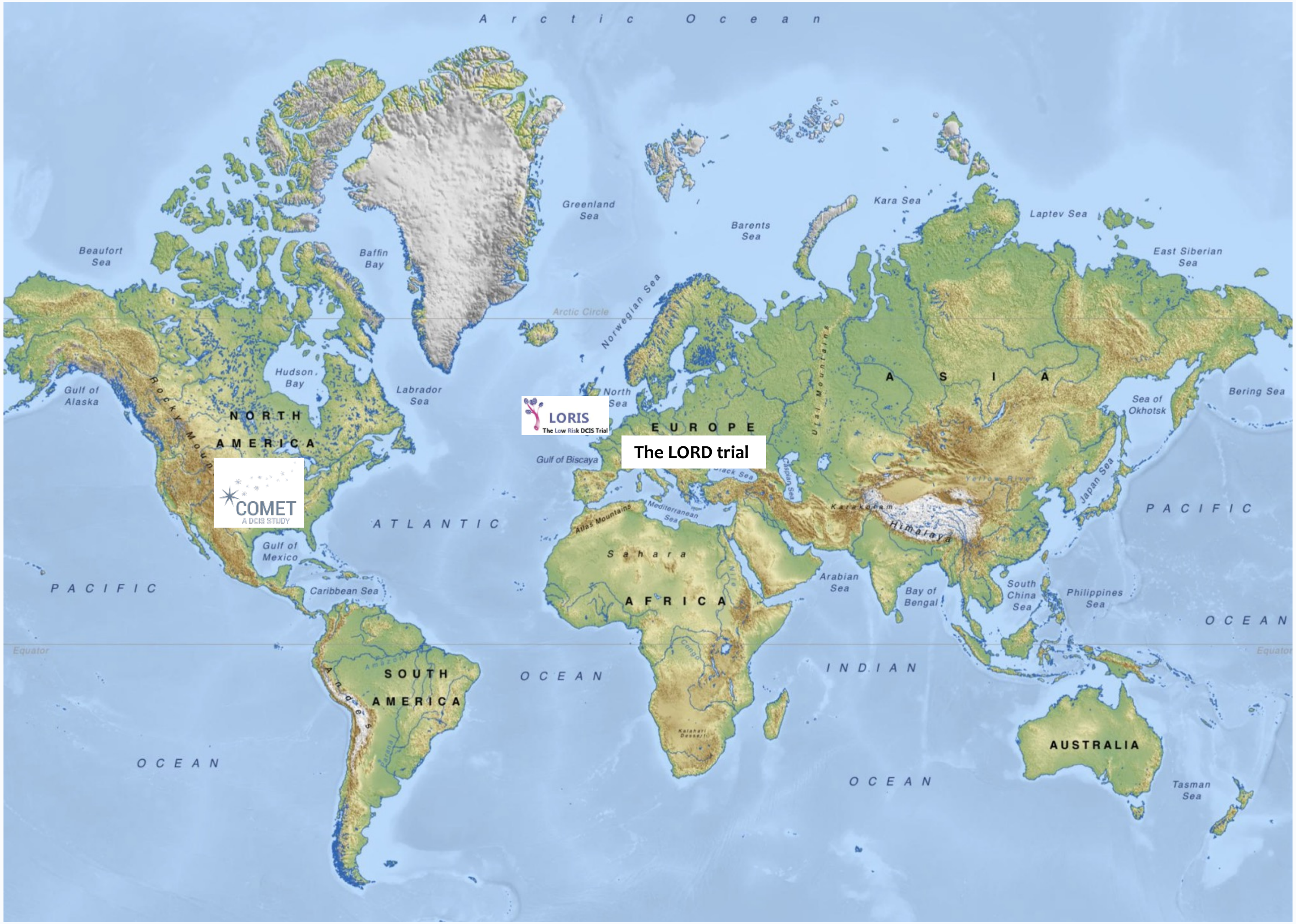
Arctic Ocean
Greenland Sea
Barents Sea
Kara Sea
Laptev Sea
East Siberian Sea
Beaufort Sea
Baffin Bay
Arctic Circle
Norwegian Sea
Hudson Bay
Labrador Sea
Gulf of Alaska
Rocky Mountains
NORTH AMERICA
North Sea
EUROPE
ASIA
Bering Sea
Sea of Okhotsk
Japan Sea
Ural Mountains
Gulf of Biscaya
Black Sea
Caspian Sea
Mediterranean Sea
Atlas Mountains
Karakoram
Himalaya
Gulf of Mexico
Caribbean Sea
ATLANTIC
PACIFIC
Sahara
AFRICA
Arabian Sea
Bay of Bengal
South China Sea
Philippines Sea
OCEAN
Equator
SOUTH AMERICA
Andes
INDIAN
AUSTRALIA
Tasman Sea
LORIS
The Low Risk DCIS Trial
COMET
A DCIS STUDY
The LORD trial

DCIS trials

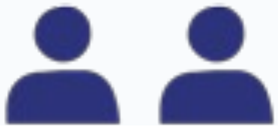

Women with low risk DCIS
(low-intermediate grade, no lump)

<u>Usual treatment</u>

- Surgery
- *+/- Radiotherapy*

More treatment if needed

DCIS trials

Women with low risk DCIS
(low-intermediate grade, no lump)

Usual treatment
- Surgery
- *+/- Radiotherapy*

More treatment if needed

Follow up for 10 years
- Mammogram yearly
- Patient questionnaires

Active monitoring
- No surgery
- No radiotherapy

Treatment if needed later

Follow up for 10 years
- Mammogram yearly
- Patient questionnaires

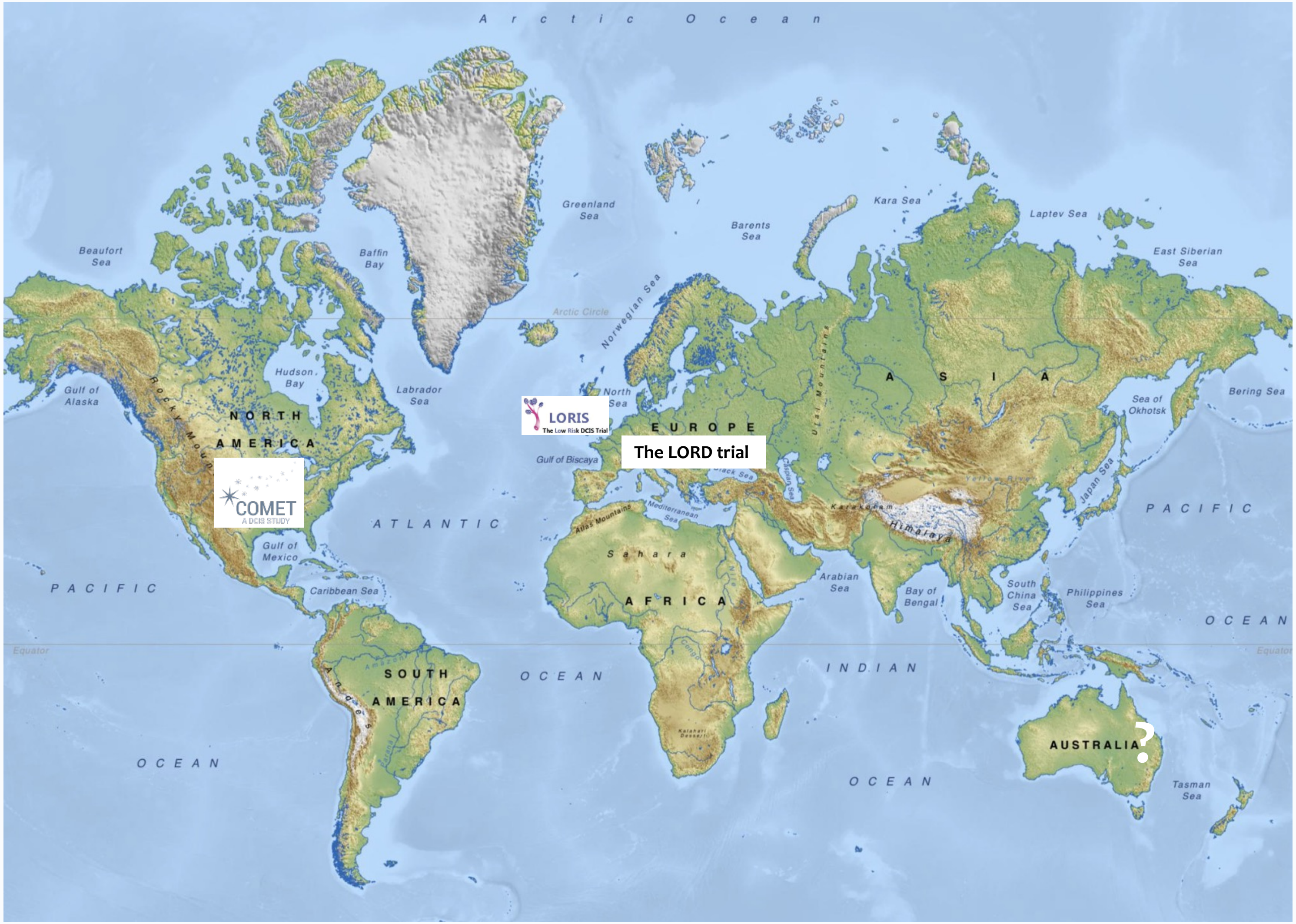
Arctic Ocean
Greenland Sea
Barents Sea
Kara Sea
Laptev Sea
East Siberian Sea
Beaufort Sea
Baffin Bay
Arctic Circle
Norwegian Sea
Hudson Bay
Labrador Sea
Gulf of Alaska
North Sea
NORTH AMERICA
EUROPE
ASIA
Sea of Okhotsk
Bering Sea
LORIS
The Low Risk DCIS Trial
The LORD trial
Gulf of Biscaya
COMET
A DCIS STUDY
ATLANTIC
Gulf of Mexico
Caribbean Sea
Sahara
AFRICA
Arabian Sea
Bay of Bengal
South China Sea
Philippines Sea
PACIFIC
OCEAN
Equator
SOUTH AMERICA
OCEAN
INDIAN
OCEAN
AUSTRALIA
?
Tasman Sea

Time for discussion

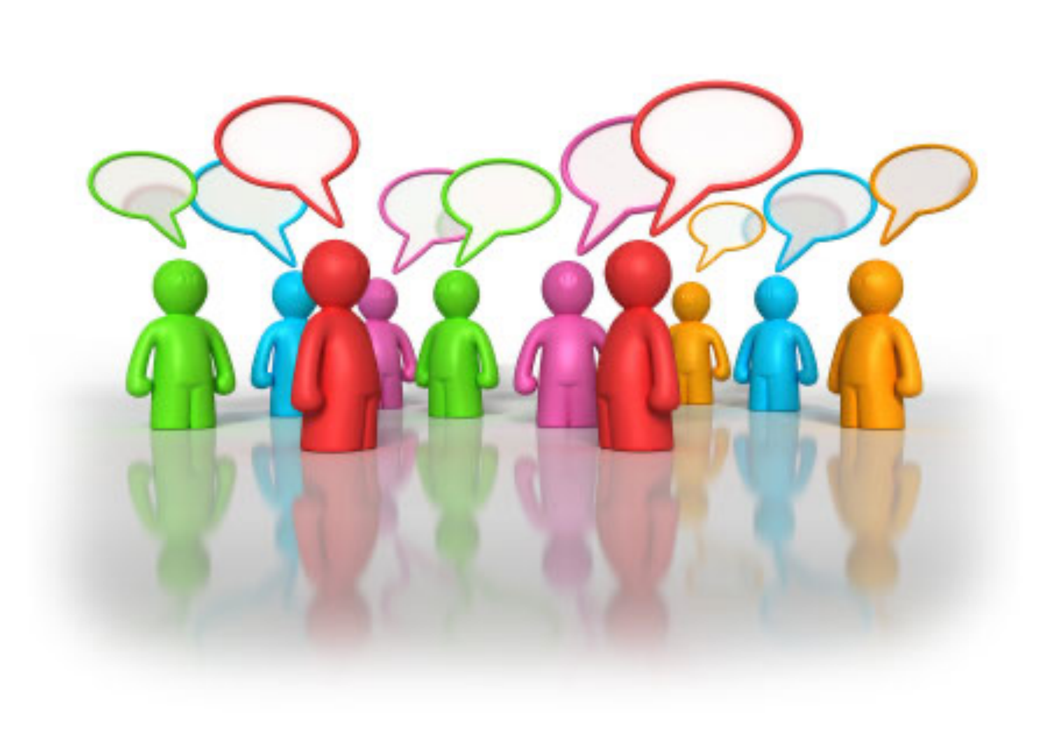

Complete Questionnaire 2

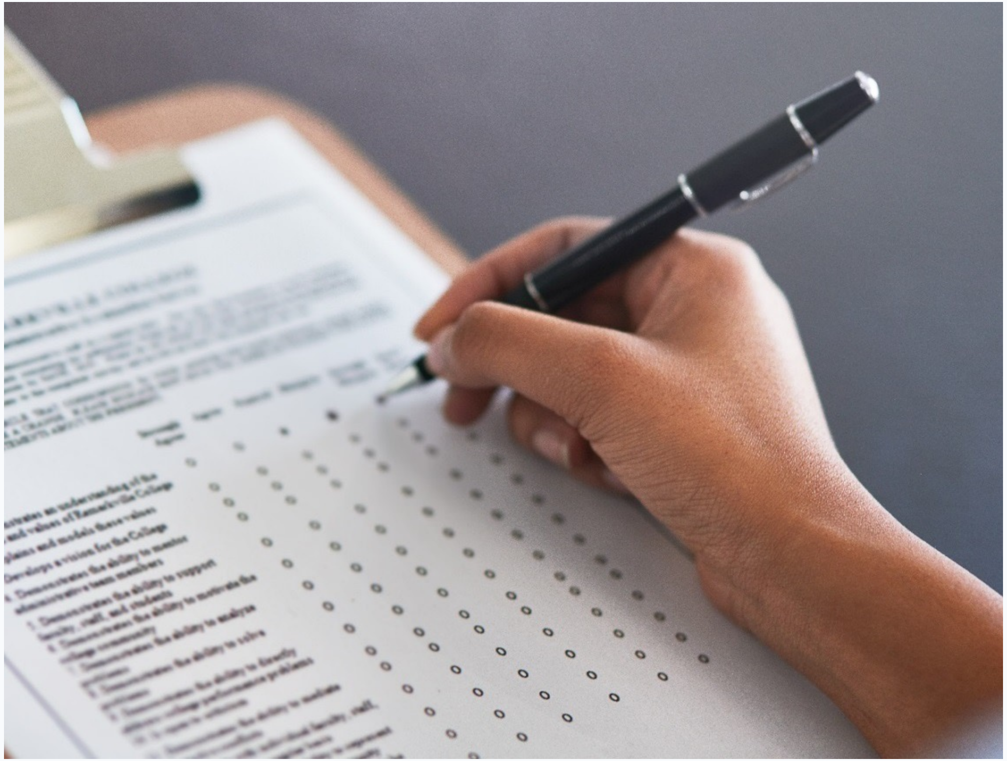

Thank you !

THE UNIVERSITY OF SYDNEY
