# Supplementary material for: Women’s views about current and future management of Ductal Carcinoma in Situ (DCIS): A mixed-methods study
Source: PLoS One. 2023 Jul 21;18(7):e0288972. doi: 10.1371/journal.pone.0288972 (PMC10361483; doi:10.1371/journal.pone.0288972)
Supplement: S3 Appendix — (PDF) [file pone.0288972.s003.pdf]

Interview: DCIS FG1 2018.05.15

Interviewer: **BOLD TEXT**
Interviewee: PLAIN TEXT
Pauses: …
Unclear words: (?)

[TRANSCRIPT BEGINS 00:06:40]

Great. Well, we'll get started. Um, now I know DCIS is probably quite unfamiliar to most of you, so I'm going to start by talking about something that probably is at least a little bit familiar, and is relevant to DCIS, and that's breast cancer screening.

So what is breast cancer screening? In Australia we have a national program that provides screening, free of charge to women, to look for early signs of breast cancer.

It's important to understand that when we talk about screening it's something that's designed for women of a certain age in the general population who are well. So it's not for women who have a breast symptom (like a lump or pain or discharge) that they've noticed that needs investigation – that's a separate process.

So the aim of screening is to find women who have cancer early, so they can be offered treatment early, in the hope that earlier treatment will lead to a better outcome. And the main goal of screening is to reduce the number of women who die from breast cancer.

Now the best available method for doing breast cancer screening is a procedure called a mammogram, which uses x-rays to make images of the breasts. And the whole idea is that these x-ray images can show up cancers or abnormalities that are so small they can't be felt as a lump.

Now if the mammogram images look abnormal or suspicious in some way, the woman gets called back to have some extra tests which may include a biopsy. This means taking, um, a small sample of cells from the breast with a needle to look at them under a microscope, for the purpose of confirming a diagnosis. So from looking at what that small sample of cells looks like under the microscope, the pathologist will either say actually everything's OK and it was a false alarm, or they'll diagnose breast cancer or DCIS.

So ductal carcinoma in situ is a condition that can affect the cells inside the breast.

Typically, DCIS doesn't cause any symptoms like a lump that you can feel, so it's generally only found through screening. Now before we started doing screening in the population, it was hardly ever diagnosed. So in the past, women may have had DCIS but because they had no symptoms and there was no screening, they wouldn't have known about it. Whereas now that we have widespread screening in the population DCIS has become a lot more common than it was before.

Now part of the challenge with this condition is that it's almost like DCIS is a new disease that we've kind of only really discovered through bringing in big population screening programs, that's in Australia and other countries, and that's happened from about the 1990s. And so everything that women are told about DCIS, and everything we're telling you today, is based on the best information we can give from our current scientific knowledge about DCIS. But it's important to understand that that knowledge is still somewhat limited. So there's quite a bit we don't really know yet about DCIS.

Now I'm going to show you this, um, diagram and some numbers now – don't worry too much about the numbers, it's really just to give you a bit of an idea of how many women are affected by DCIS.

So if you imagine that each one of these dots represents one woman. If we take 1000 women in Australia who have breast screening over a period of 25 years, in total about 81 the women will be diagnosed through screening, so some… a diagnosis of one kind or another that they get, as a direct result of the screening mammogram and the further investigations that follow.

If we look more closely at these 81 women, we find that 16 of them have DCIS and the other 65 have what's called invasive breast cancer – and I'm going to explain the difference in a minute. So as you can see, the women with DCIS are a smaller group – about 1/5 of the total group of women who are diagnosed through screening.

So what is DCIS? Let's talk about that in a bit more detail, breaking it down

into each of the words. So the word ductal means it relates to a duct or tube in the breast that carries milk to the nipple. Carcinoma means the cells show abnormal features similar to cancer.

And then the 'in situ' part is very important to defining DCIS. So this illustration shows how DCIS is different from 'invasive' breast cancer, which is really just a more precise term for what we currently think of as… what we commonly understand breast cancer to be. So based on what they can see looking at the cells from the biopsy, the pathologist will diagnose that it's either DCIS (meaning that the abnormal cells are entirely contained within the milk duct) or invasive breast cancer (which means that those cells have moved outside the milk duct into the rest of the breast.

So DCIS and invasive breast cancer are different but they're related. In DCIS, the abnormal cells are contained within the milk duct and have not spread outside the ducts into the other breast tissue. So as long as those cells remain contained like that, DCIS is not a life-threatening condition at all. But DCIS might develop into invasive breast cancer later on, and if that happens the time frame for it to happen is really variable, so it could take many years or decades or it could happen much sooner than that.

In invasive breast cancer, some of the abnormal cells which were previously just inside the ducts have actually taken the next step and spread into the breast tissue around the ducts, and once that's happened, the cancer could then spread to other parts of the body, which is when you really have a problem.

But it's good to remember that although invasive breast cancer has the potential to be a life-threatening disease, these days the vast majority of women diagnosed with breast cancer will be treated successfully and will survive their cancer.

Now DCIS gets described in a lot of different ways. This slide just shows some of the different language that's used about DCIS. And none of these are really wrong but it can be a bit confusing for women because it's described both as a type of cancer and as something that's not quite cancer.

So if you haven't heard of DCIS before, you might have come across some of these other terms like Stage Zero breast cancer or non-invasive breast cancer or pre- invasive breast cancer.

And then what happens after DCIS is diagnosed? Well, as I've said, DCIS itself doesn't affect a woman's health, but it has the potential to turn into invasive breast cancer. Now we have reason to believe that this progression to invasive cancer might not happen for everyone, and I'll explain more about that later. But the bottom line is right now we can't reliably predict which of the women with DCIS will get invasive breast cancer and if they do how long it might take for that to happen. And that's why virtually every woman diagnosed with DCIS is given treatment, to try and prevent that progression from ever happening. And I'll talk shortly in a bit more detail about what that treatment involves.

But firstly, I just wanted to ask you have you ever heard of DCIS before?

No.

No, I haven't.

No.

No? Nobody?

No.

I think I have.

Yep.

But I didn't realise it.

So what… in what context?

Well I, um, a friend… a friend had, like was… so they had it.

Mmm hmm.

But I didn't really realise… but said I've got cancer but it… I've got abnormal cells.

Ok. So do you feel that you understand what I've said so far?

[LOTS OF YES's]

Yep? Any, anything that's unclear or confusing about it? Have you got any questions? Ok. And have you ever heard the idea that some people are diagnosed through screening, and have treatment, for something that might not have ever become life-threatening?

[LOTS OF NO's]

Normally if, um…

It's cancer.

… it's not life-threatening or, um, or you're not sure then you wouldn't get treatment. So this is sort of something new.

Mmm hmm.

They usually tell you to come back after 6 months and check it again, and… and take it from there.

Mmm hmm. Ok. And you were, you were nodding I think?

Yes, yeah, I think, um… just ask the question again for me?

Well, yeah, just whether you've, you've heard that some people can be diagnosed with something and have treatment, even though that something might not have caused them health problems, that might not have been a life-threatening disease.

Er, yeah, I, I probably was thinking someone's, um, being diagnosed but has chosen to have the treatment like, you know, like say diagnosed with some sort of cancer but they say let's just wait and see. But then they so, no, let's just not wait and see but go ahead and do something rather than see if it, you know, it's slow acting or whatever. Does that make sense?

Yeah, yeah. Ok. So what are… for all of you what are your thoughts and feelings about, about this, about DCIS, what I've said so far?

Makes sense.

Yeah.

I think it's a mistake to wait to see whether it's going to turn when you could say, well, I'll prevent it… before it starts.

Mmm hmm.

Well it makes me more determined to have it, keep going to screening.

Yeah, me too.

Mmm. Keep checking, yeah.

Mmm. Me too.

I'm very lazy for screening but, er, now it, yeah… I go regularly for the screening.

Yeah? So why do you say it makes you… why does it make you feel that way?

Er, because, er, it's always, um… it's the, the thought of having breast cancer but now it gives you, you know, more hope that if you have this and you can prevent it and live a normal… it's very important and all that. Er, it's, er, it's not… doesn't have to be cancer but, er, and it can be prevented. So it can stay in the… the… er, where it is within the, the ducts.

Does it always start with, um, that, DC…?

What is…

Yeah.

… the symptoms are only… sorry, sorry.

No, that's ok. Yeah, so before it's invasive does it always start with that first? The DCSI?

It's not always…

DCI… (Laughs)

… DCIS. So DCIS means it's, it started in the duct.

Oh, ok.

Um, breast cancer can also start in…

Somewhere else.

… yeah, what's called the lobe.

Ok.

Um… which can be kind of similar, so you've got lobular carcinoma in situ and then…

Yeah.

Yeah.

And it's usually invasive to start with if it's just in the lobe, not in the duct?

Um, no, it can, it can, so it will start… it can start and be, like one, at the beginning contained somewhere and then… and then move to become invasive breast cancer.

Mmm.

Ok. Um, well we're going to talk a little bit more about treatment for DCIS. So the goal of treating DCIS is to prevent those abnormal cells from turning into invasive cancer. And overall it seems that our current treatments are doing a pretty good job of achieving this goal.

So surgery is really the primary form of treatment for DCIS. So that's doing an operation on the breast, in hospital, under general anaesthetic. Doing surgery also means the pathologists get another chance to look at these cells under the microscope, but this time they've got more to work with because they've hopefully got the whole area that's effected by DCIS, um, rather than just that small bit they looked at in the biopsy. So in most cases this will confirm that it really is just DCIS as the biopsy suggested. But in some cases they find after surgery that the patient actually has invasive breast cancer. So that patient's diagnosis is changed or upgraded at that

point.

Now there are different types of surgery that a patient can have, and surgery may also be combined with another type of treatment. So exactly what treatment each patient has will depend on features of her DCIS (like its size and grade – and I'll talk more about grade later on) as well as characteristics of the woman herself (like her age, overall health, and her preferences).

So I'm going to tell you in a bit more detail about the 2 most common options for treating DCIS. And both of these approaches are considered very effective, and survival rates for women with DCIS are excellent.

So treatment for DCIS most commonly involves breast conserving surgery and then radiotherapy. This means having an operation to remove the part of the breast affected by DCIS and a small area of healthy breast around it.

Um, and after breast conserving surgery, a pathologist will look at the breast tissue that was removed. And sometimes, if they find there's a bit more disease than they originally thought, the woman may need more surgery.

Now everyone responds differently to surgery. Some side effects happen to most people, others happen only occasionally. And some side effects happen straight after surgery, others may take longer to develop. But some of the common side effects include things like pain, numbness, and bruising around the wound.

And after this surgery, the patient will typically have radiotherapy, and that's normally every day for a few weeks. Radiotherapy uses x-rays to destroy any abnormal cells that may be left in the breast.

Now receiving radiotherapy is painless. But women may experience some side effects. So during radiotherapy treatment, the skin of the breast can become red and dry, a bit like sunburn, or the skin can become darker; and women often feel more tired than usual. And these side effects usually settle down after treatment ends and things get back to normal.

Now during breast radiotherapy, it's possible that a small dose of radiation

may reach the heart or lungs, depending exactly where the DCIS is. So some studies have suggested that women who had breast radiotherapy in the past may have a slightly increased chance of having a heart problem later on in life, like a heart attack. So doctors these days are using more modern techniques when giving radiotherapy to try and minimise this problem as much as they can.

Now instead of having breast conserving surgery plus radiotherapy, sometimes a mastectomy may be recommended, for example if the area of DCIS is quite large compared to the size of the breast or for some other reason. So this is a bigger operation which means there's a higher chance of complications and a longer recovery time.

So all the side effects I mentioned for breast conserving surgery are also relevant for mastectomy but because the surgery is more extensive, there may be additional side effects such as stiffness in the arm or shoulder.

Obviously mastectomy affects the body shape and it can affect women's body image or how they feel about their bodies. And many women have the possibility to consider breast reconstruction surgery to try and help with that.

Now just in case you're wondering at this point I want to mention that chemotherapy is not a treatment that's typically used for DCIS. So the main treatments are, as I've outlined: different types of surgery and radiotherapy.

So what are your thoughts and feelings about that information about treatment for DCIS?

It's normal, I guess.

[A FEW MMM's and YES's]

It's, um, easier than, er, the invasive, er, cancer treatments. Um…

In what way?

Er, like you don't have to have chemotherapy, yeah, and have to, um… yeah, have an invasive operation. Yeah.

Ok.

You hear, you hear different people have treatments. You know, that if people say I have breast cancer and then some people might not have chemo, some… some will just have radiation and I've often thought, you know, like you don't ever ask, like why are you have one and not the other really? But now I would imagine that it's because maybe it's, it's been, you know, invasive or not invasive. I mean, so it makes a lot of sense. So I, I didn't really realise that before now (laughs).

Mmm hmm.

You mentioned that, um, radiotherapy is used not chemo, why?

So chemotherapy, um, well radiotherapy treats the breast itself. It's, it's a local treatment. And that's, um… appropriate for DCIS because DCIS is only in the breast.

Mmm hmm.

Whereas chemotherapy is used, um… chemotherapy affects the whole body, so it's used if there's a chance that, um, they, you know, a cancer may have already moved into other parts of the body.

Oh, ok.

Yeah. Ok.

Is there any other treatment for it? No?

Well, those are the, the main treatments. Sometimes, um, another form of treatment called hormonal therapy might be used, although that's not, um, very common. But that's, er… depending on the type of DCIS, for some women, um… hormone therapy might be relevant. And that means taking a medication every day for a number of years that, that effects the hormones in the body that might be, er, relevant to that woman's, um, DCIS. But really… typically it's the surgery and then with or without radiotherapy. So, any other thoughts? Any sort of concerns about…

So that, um, having to have the mastectomy, um, because of, um, DCIS, um, that

would, um, probably be a smaller group of people that, that would need that and that would be, they'd have to be very worried that it was going to turn to, um, to full cancer, yeah, to have that. Yeah, so, um, but for most people it would sort of hopefully just be contained within the, within the duct. Mmm.

Ok. So… just hypothetically: If you had just been diagnosed with DCIS and told about these treatment options, how do you think you might feel?

Be good that it got caught in time for a start (laughs).

[A FEW YEAH's]

(UNCLEAR)… it will turn into, you know, invasive cancer… (UNCLEAR), you have to keep on the treatment…

Can be controlled.

I think you'd still be anxious about it because it's still a form of cancer.

I'd be grateful that it wasn't chemotherapy kind of…

[LOTS OF MMM's and YEAH's]

… like… I'd hate to have to have chemotherapy. Like it just, like you said, it affects your whole body and it kills the good cells, you know?

[A FEW MMM's]

Ok. Anyone feel a bit differently? No? Ok. Um… and so what do you think would be your approach to kind of deciding between those options if you were told you could either have breast conserving surgery and radiotherapy or a mastectomy? What do you think you'd… how would you feel about…

I'd take the first option first.

[A FEW MMM's]

Yeah?

Yeah, the mastectomy would be my last resort.

I think most people would do the same, you know. If you conserve… conservative but I think a lot of the time though it's really left up to the, the surgeon…

Yeah.

… once he gets in and finds out exactly what… type of size and what he's actually dealing with, whether it is or isn't. But yeah, I think it would be pretty devastating to go in thinking you've only got to have conservative surgery, come out, when you come out from it you've had a mastectomy.

Yeah, I'd just be guided by what the specialist, um, said was the, um, the best option for you. You know, even if it wasn't the, the option that you, um, you would prefer to have. Like, you know, I'd be guided by them because they're the experts and they know what's best for you.

Mmm hmm.

I say I agree with the, er, specialist opinion. Yeah… (UNCLEAR).

I, I think I'd probably, um, if I knew it was DCIS, say I want to, um… you know, try the, the lumpectomy first. I think I'd be quite clear that that's what I wanted to do and then, you know, if, if it seemed to be too big like then I, like, you know, if it needs be go back, do other things. But, yeah… I'd think it was, um… yeah, it was, it could be a lot worse, so let's sort of, let's, let's, um, be pleased we've got it early and, and, um, not be too drastic unless we really need to. So let's, you know, take it slowly.

Yeah, the less invasive treatment sounds… sounds better to me.

Ok. Good. Ok, so… one question that is currently occupying DCIS experts at the moment is: Could some women with DCIS actually avoid treatment safely? So current treatment for DCIS is based on our best scientific knowledge and of course wanting to do what's in the best interests of patients.

And as I said before, survival rates for DCIS are excellent. But many patients do experience side effects from treatment, and this includes both physical effects on their bodies, and emotional or psychological effects as well, and they affect women in the short term and in the long term.

So breast cancer experts are now doing research to try and identify women who have DCIS but have a low chance of ever getting invasive breast cancer so that in the future, women like this may be able to avoid surgery and other treatments and therefore avoid all those problems that can come along with treatment, while still having excellent survival rates.

Now as I said before, it's very difficult to reliably tell who these 'lower risk' women are, but we do have some clues. And one of them is something I mentioned earlier called grade.

So after the biopsy is taken, the pathologist looks at the sample under the microscope and gives that case of DCIS a grade based on how the cells look, which gives an idea of how fast the cells are likely to grow.

So low grade indicates that the cells appear to have a lower level of activity, which suggests they may stay like that for quite a long period of time. On the other hand, high grade DCIS means the cells appear more active, so they're likely to grow at a faster rate. So it's considered important to treat high grade DCIS because otherwise it's pretty likely to develop into invasive breast cancer at some point. And then there's an intermediate grade which is somewhere in the middle.

Now you might be wondering, if virtually all women with DCIS are having treatment, so having surgery to take the DCIS out, why am I saying that we think low grade DCIS may stay as it is for many years, even without treatment.

Well, we think that based on a small number of research studies similar to this one.

So as an example I want to tell you a bit about a study that was done in America using a database of routinely collected information about cases of DCIS. So these blue bars represent all the patients in the database who had low grade DCIS. And as you can see, the vast majority of the patients had surgery, um, almost 9000 of them, which is of course the typical thing to do for DCIS, surgery to take it out, so that's what's recommended in all the guidelines for doctors.

But a small number of patients, a couple of hundred, didn't have surgery

for some reason, and we don't really know why not.

And this database kept track of key events that happened to these patients over many years, for example it recorded if any patients died from breast cancer. And what the researchers did was compare the larger group of women who had surgery with the smaller group of women who, after about 10 years. And they found that the women who did have treatment had a very high survival rate, so 99% of them had not died from breast cancer, so they were doing really well. And the research also found that among the women who did not have treatment, 99% of them also had not died of breast cancer.

So this led the researchers to suggest that there may be a subgroup of women with a lower risk kind of DCIS who are likely to have really good health outcomes over time, whether they do or don't have the typical treatment of immediate surgery.

So this idea of a subgroup of DCIS that we could call 'low risk DCIS' is a big focus of current research to investigate whether women in that category could possibly avoid treatment.

And there's a range of factors that are relevant to trying to define this category of DCIS – so grade is an important one but it's not the only thing. There's lots of other, um, factors that, that taken into account as well that I won't go into now.

So coming back to this little diagram I just want to give you a rough idea of how many women with DCIS might be classified into this lower risk category. So of our 16 women with DCIS from before... we think about 5 women have what could be classified as lower risk DCIS based on their biopsy. For the other 11 they are considered to have a higher chance of getting invasive breast cancer so it's still considered more appropriate that those women do have treatment.

And these 'lower risk' women are the focus of current research looking at whether they might be able to avoid treatment. So I'm going to tell you more about that research now, and it's important to remember that we're talking about this small, select group within DCIS patients where

researchers are considering a new, different approach to managing their condition.

And the type of studies I want to talk about now are called clinical trials, so let me explain what they are.

A clinical trial is a research study that asks an important, specific question about a certain health condition. Clinical trials often test new ways to manage a condition, by comparing a newer approach with whatever is the current standard treatment.

Trials are designed to find out whether patient outcomes (so health and quality of life) are similar for both options, or whether one option is better than the other.

Now if you think back to that study I talked about before, with the blue bar graph, it was done by looking at a database containing routinely collected information, basic information about past patients. And although studies like that can be valuable for giving us interesting ideas, it's hard to draw firm conclusions from that kind of research because there's a lot of important information missing, for example details about those patients who unusually did not have surgery and why.

Whereas clinical trials are specially designed to overcome the limitations of other types of research. So that's why trials are considered to give us the highest quality of evidence in science.

So… this illustrates the basic design of a simple clinical trial. Sometimes they can be more complicated, for example with more than 2 groups, but most typically, each patient in the trial is put into 1 of 2 groups. One group of patients gets the standard treatment, while the other group gets a new treatment or a new way of managing their condition. And which group each patient goes into is determined by chance, using a computer. This process is called randomisation and it's a really important part of clinical trials. Because when we compare 2 groups of patients having their condition treated or managed in different ways, we want to make sure the groups are very much the same in every way possible other than the way their condition is treated or managed. If patients and their doctors choose which

treatment to have, then the patients in the different groups are very likely to be different from each other in important ways that are relevant to their health, which can create bias that makes the results less reliable. So patients who volunteer to take part in a clinical trial need to be comfortable with ending up in either of the different groups because they don't get to choose which group they're in. Randomisation makes sure that the study groups are as similar as possible. And that way, we can get the most reliable information from the results of the trial to learn about the effects of those different ways of managing the condition.

Now deciding by chance how a patient will be treated may seem strange, but it's considered an ethical thing to do in situations where we genuinely don't know whether one option is better than the other.

Now there are currently 3 clinical trials going on around the world for women with low risk DCIS: one in the UK, one in Europe based in the Netherlands, and one in the US. They all just started in the last couple of years, and together they will involve over 3000 women. Now there are slight differences between the trials, but they all have the same general design.

The aim of these trials is to learn whether women with low risk DCIS could safely avoid having treatments that may not be necessary for them.

So the women who were invited to take part in these trials have to meet strict criteria to be considered suitable. They have to have low or intermediate grade DCIS (not high grade), they can't have a lump you can feel, plus there's other more technical rules I won't go into. So remember, this is what typically happens to women with low risk DCIS: they have treatment pretty much straight away, and we talked about those typical treatments before, so surgery of some kind with or without radiotherapy.

Of course in the following months and years, if it seems their initial treatment didn't work quite as well as hoped, or their DCIS comes back, or they get DCIS elsewhere or invasive breast cancer, they might end up having some further treatment if needed.

So that's the normal scenario for pretty much all women in places like

Australia where there is no clinical trial. For women who do have a trial available where they live, if they're suitable to offer the trial, they may just say no thank you, I'll stick with the usual treatment. Or, if they decide to join the trial... then they get randomised to either usual treatment or active monitoring. So the usual treatment group of course just has usual treatment. And the other group has a different approach where in the first instance they don't have surgery or radiotherapy. And then patients in both groups are monitored closely, so they all have mammograms at least every year. And importantly, patients in the monitoring group may have treatment later if there's any sign that their DCIS is progressing, and we expect that to happen for some women. So this approach may be called active monitoring or active surveillance and it's actually already an accepted option for men with early prostate cancer because trials similar to this have shown that it's safe. So these trials will test out the idea that this monitoring approach where you only give treatment if it's needed may result in the same very high survival rates as standard immediate treatment, but with fewer side effects overall because some women will avoid treatment altogether, and others will end up having treatment down the track but it's delayed so they may have a period of some years first where they're free of those treatment effects. So those are potential advantages of monitoring.

Then there's also potential disadvantages the trials will examine. For example, patients who don't have immediate treatment may be more likely to get invasive breast cancer – although with the monitoring it's still likely to be picked up at an early stage. As I mentioned before, it's also possible that some women who are diagnosed with DCIS based on the biopsy actually have invasive cancer at the time that the biopsy didn't pick up. And we also don't really know how women having monitoring will feel about not having treatment, so in the trials the patients will do questionnaires to, um, help the researchers understand the psychological effects of this approach, and that's a very important aspect.

Now because there's only a small and select group of women who are eligible for these trials, and because they then need to be followed up for quite a while, it will still be some years before any results from these trials are available.

In the meantime, it's also possible that a similar study could be started in Australia and New Zealand, so women here might one day have the possibility to go into a trial like that.

But otherwise until the results of these trials come out, women with DCIS will continue to have treatment as normal because we don't have strong research evidence yet to prove that any other approach, like monitoring, is safe.

So what are your thoughts and feelings about these clinical trials?

Well they're needed for a start.

[LOTS OF MMM's]

That's how they find things. Um… tell me, men with prostate they usually find out through a blood test, the PCI I think it's called.

PSA?

Yeah, that one (laughs). Um, there isn't a blood test for women that have… can breast cancer?

No.

There isn't. Oh. Haven't got that far yet. Hmmm. Yeah.

But considering the extremely low number, as you said, out of 81, you've got… down to 16 and then from 16 you're down to 5… I think sort of places like Australia the chances of a study would be fairly remote because our population one, is not big and when you get down to such a very tiny number on the overall thing, it would be really hard to (?) a proper sample size to see if a study here would be effective.

Yeah, so that was down to 5 from 1000 to start with. So yeah, you're right Australia has a smaller population and, um… yeah, so a trial here may or may not happen.

Yeah.

Um, otherwise we're, you know, we're relying on the international trials, um… but yeah, um, I am, I am kind of interested in how you might feel if

there were a trial here one day. So…

Yeah, I, I didn't understand about the random, er, trials.

Mmm hmm.

But I understood if it's right, but every group of people who are going through the trial they don't, they don't have to choose which, isn't it? Is that right?

That's right, yeah.

They don't choose. They just go randomly?

Yeah.

Ok.

So for each, um, each person who enters the trial is considered suitable to have either normal treatment or to have monitoring, and so it's, it's kind of like flipping a coin, which group she goes into.

Mmm.

Um, and then, and that means that both groups contain women who are very similar because there's nothing systematic that determine which one they (house?).

Yes, yes.

Yeah, and then they're followed over, um, you know, about 10 years to, to see what happens.

Mmm.

It's like the placebo effect.

Yeah.

When they're testing medication.

That's right.

[A FEW YEAH's]

It's like similar to a drug trial…

That's right.

… where some patients get the actual drug and some get the placebo.

Yeah.

Of course in this case you, you know which one you're in…

Mmm.

… but, um, yeah, the same concept. That's a, a clinical trial.

Yeah, but as you mentioned, they'd be monitoring anyway so if the cancer were to become more invasive they would then step in and say, ok, you're out of that trial, you have to go get… approach that, correct?

So you would have an operation if that happens.

Yes.

You could still be in the trial.

Yes.

Um, but yes, you would be, er… they will, you know, keep a track obviously.

Yeah.

You have… yeah, if you, if your condition changes and you're in the monitoring group and it seems like you should have surgery then you would have the surgery.

Yep.

They have to decide the proper treatment for it then.

Yeah.

Yeah.

Yeah, so basically everyone at the beginning is considered, um, ok to not do

surgery and then if that changes and it looks like it's change… you know, the DCIS is progressing at all, and it seems like surgery is appropriate then they'll just do it at that point. And that might be a few years later.

I think it's good. I would be happy to do that.

Yeah?

Yeah.

I think anything that helps us to understand, um, more and get more and more advanced treatment as time goes on, yeah, is, is good. We need to find out as much as what we can about these things.

[A FEW MMM's]

Yeah, but it's nothing to loose I think. It's something that you… you, you be safe, I think.

Mmm hmm.

Yeah.

Because we're all starting in the low-grade anyway, so it's not like putting in high-grade. So you'll all stay on same level, low-grade, and that's the only way to find out who's going to go quicker and who's not.

Mmm hmm.

As I said, I, I relate it to the prostate because my husband went through it. So the way they found out is the PSA was so much and then within 6 months it went really high. So the surgeon already knew this is a very fast cancer in your… so this is the same thing. Hopefully it'll come up with a blood test (laughs) in the future.

[A FEW MMM's]

Saying, ok, now you've got breast cancer and, um, they could monitor it from the blood test to see how fast it's moving.

Mmm hmm.

Or any form of cancer for that reason, you know?

Ok.

The figure that, that you gave me for about 3000 women who had surgery and the 200 who didn't have, but it doesn't, er, stay that figure. It could happen to other women who maybe decided not to have surgery and had maybe some, er… cancer maybe? Is it… was it that, er… you know what I mean?

That was the America study and that was out of 10,000 people.

10,000?

Yeah, wasn't it?

Yeah.

Yeah, so… so the, the thing about that is, um… it's a bit, you know, it, it suggests that, um… women may not need surgery, um, but we don't really understand why those few women didn't have surgery and, and maybe they were different in some ways to the, to the other women. So maybe they tended to be older, maybe their health wasn't so good, so maybe there's certain reasons why they didn't do surgery. Um, and so we can't really generalise. We can't say whether that applies to other women. Whereas in a clinical trial, like this, um, you have quite a, a big group of women at the beginning who are just randomly treated in one way or another. And that's why you can have more confidence when you compare what happens that the results are, you know, meaningful for, for other women in the future.

Yeah, that means it doesn't matter what they did, but, er… the, the result was, was good, was positive for both, both groups.

Yeah.

Yeah. Yeah.

I suppose it's all about getting more information, isn't it, about breast cancer?

Yeah, it's about early screening and…

Years and years ago you got it and that was it. Now there's other elements that,

that are indicative signs of, of perhaps where it might go. So just anything that enables you to have more information that can bring to the situation I think is worthwhile. So it's, um, people being… putting their hand up to, to say that. I suppose if there were… other risk factors that came into it, you know, people, you know, wouldn't be chose for that random sample, would they?

Yeah, exactly. If they… they have to be considered in the, to be in a low-risk category to even be asked.

Yeah.

And DCIS has, like you said, has no symptoms?

It typically has no symptoms.

No symptoms at all.

So you wouldn't know about it unless you had screening that picked it up.

Mmm.

Ok, so I think a few people have said that they would go into a trial where they were randomised to treatment or monitoring. What about those of you who haven't said anything yet? Do you feel the same way, or do you feel a bit differently… if you were diagnosed with low risk DCIS and you were told that you could go into a trial like this, do you think you would be happy to do that? Or would you…

I would choose active monitoring.

Yeah.

Ok, so just one at a time, sorry. You were saying?

I would choose active monitoring rather than, yeah…

Why do you say that?

I would, um… discuss with the doctor and choose the least, um, traumatic way of dealing with it. Yeah.

Ok.

So I'd rather wait and see what happens than, than go through the… the treatment.

Ok. And what were you going to say?

I would like to go for the treatment first instead of go for the trial because thinking my children, you know, my family so… so, but I think treatment is good option, I think, yeah.

Ok.

I'd be happy to do a trial.

Me too. To help.

I think it's a great…

Help the others.

… great way of education people and, um…

Help…

… (UNCLEAR)…

… something, yeah.

It's a positive, isn't it?

Yeah.

Ok. What about you?

Yeah. If doctor think, you know, I need to do the trial I think I would go for it.

Yeah, ok. Have you said one way or another (laughs)? I can't remember now.

No, I (UNCLEAR)… the chance of a trial I think would be remote because it's such a small, we're such a small number when you get down, as I said, from 1000 to 16, to 5.

Mmm.

But in reality probably would be fine with the trial because you always have that backup under the active monitoring. If something does go astray you've still got treatment as an option.

Mmm hmm.

Um, the beauty, and I don't know that anyone really wants to go and put their body through all that trauma if they don't have to. Um, and who's to say that when we start, that when they start they say 10 years, the advancements that have happened even in the last, you know, so many years, it may be that half way through the trial they come up with a solution. Yes, you pop a couple of pills and away you go. We've found the magic cure.

[A FEW MMM's]

Wouldn't that be nice.

[LOTS OF MMM's]

Last night they were on that they'd found the cure for aging.

[LAUGHTER]

Because they, because they found… they were doing something again on cancer research and whatever cells they were, um, trying to what was it… rewire, they managed to rewire some of the… the, er… what reduce aging or reverse it. So there's always that going on as well.

Ok. So one comment I might make in response to what you said, that you would prefer to have active monitoring. Um, just, just tot point out again that at the moment you might struggle to find a doctor who feels comfortable to, for you to have active monitoring outside of a trial like this. So it's not that it absolutely never happens, but usually… it would only happen if the woman was, for example, in such poor health that she was considered unsuitable for surgery. Whereas somebody who's, you know, suitable for surgery you'd probably find most surgeons, you know, are pretty keen to do that. So… but, you know, it's interesting that you say that preference. But sometimes, you know, it could be that the only way to

really get monitoring rather than treatment is to go into a trial. But that's ok, it's all hypothetical anyway.

It's just, you know, except in the trial you don't have the choice. You don't have an option. You just go into either treatment or active monitoring. Yeah. But you're saying like you don't even have a choice to do active monitoring?

Well, um, at the moment it's not really a choice that's presented because the medical community doesn't feel really confident yet until we have results of trials like this.

Mmm.

Do you know what the statistics are in Australia, like how many women a year are diagnosed with this?

Yeah, it's about… somewhere between 1600 and 2000.

In all of Australia?

In all of Australia each year. But that's DCIS in total. So… you know… probably about half of those are high-grade DCIS and then…

[A FEW MMM's]

… a smaller subset would have…

What's the, sorry, what's the ratio of age?

Well, um…

Like the most effected. Do, do they have a…

Yeah, I mean because it's most typically found through screening the, it's most commonly found in the age group that's targeted for screening, which is 50-74.

Mmm.

So it can happen, er… I guess at any age really, but more typically over 50.

You'd possibly find people who were younger, diagnosed. And the only way it

would be is because they have a history of the breast cancer in their family or something a long the lines, so actually are screened earlier. And that's the only way you'd probably catch somebody below 50.

Mmm, yep. So in these trials they're including women, um, in their 40's and upwards. There's no kind of age limit for going into the trial. Ok. So… I think you've all said now kind of which way you're leaning in terms of whether you'd go into a trial or, or not. Um, can I just ask you to think about, you know, are there any other kind of circumstances where you would feel differently to what you said? If anything was different about you or if anything was different about the research that would make you change your mind either way? Anything that… where you'd feel that you wouldn't want to necessarily have treatment? Anything that would make you Lily, feel like you, you would want to have treatment?

Mmm. Depends on the grade.

Ok. Yep.

Only thing that might be an influencing factor is where you actually live.

Mmm hmm.

Living in, in Sydney or a major metropolitan area, access to all those essential type services are literally at your fingertips. But if you start getting out to the more remote areas, it might change your mind about going and… to say well I'll just opt for the monitoring and actually opt for the treatment because, you know, it's, it's done with, whatever. Not traipsing backwards and forwards at potentially vast distances.

Yep, ok.

And if you had family members who had breast cancer… I mean, would, would that make you ineligible for the trial? Would you think?

Um, not just having family members who had breast cancer, but I think if you were one of those women who has, um, an identified high risk gene, like one of those special genetic mutations that puts you at really high risk, then I think you would be ineligible. But, um… just having, you know, an

Aunt or something with breast cancer, it'd be ok.

Yeah.

'Cause that's quite common.

What about any kind of cancer? Does that effect the trial, if, if a member of your family has had say bowel cancer or… that wouldn't effect the trial at all?

No. So they, they would probably collect lots of information from you about all those kinds of things and, and, you know, check whether it made any difference to outcomes, but no, we don't, we don't believe there's a link between breast cancer and bowel cancer. The only other cancer that is associated is, um, ovarian cancer. And that's in those special, um, genetic mutation cases, where they talk about the Brca 1 and Brca 2 genes. Um, like if you think of Angelina Jolie or certain people who have, um, is very strong genes in their family. But that's, that's quite a small separate population.

Ok. Um, so is there anything else that you would want to know if you were thinking about going in a, a trial like this?

I'd be interested in how you, how you would be monitored.

Yeah.

You know, so that if things… um… you know, if yours ended up being, you know, something that developed into more like, um, you know, how thorough that was, I suppose. I mean, interested in how the other studies had gone and I suppose interested in those, those two blue graphs, like what were the, um… you know, the ages, like the conditions of the people.

Yeah.

Like what were the factors, you know, that, um, in that group. Were they mostly old people or were they, you know, mostly young people or, you know, some of those things?

Knowing what they go through, what the side effects of the treatment and… yeah.

I suppose what's your chance of having…

[LOTS OF MMM's]

Yeah, that's what I was going to say, I'd want to know sort of what chance there is that, um, maybe you made the wrong decision and you should have just opted for the surgery?

Mmm, yeah.

Like what risk is there with going down that path?

Yeah.

Mmm hmm.

I don't see a big risk myself because… if you're going in a trial it's only if you're low-grade, so you're already low…

Mmm.

…risk. And then the way the computer will take, I think, from what I gather, in consideration all your history, your health, I assume?

Well no. So basically if… based on your health and your case of DCIS if you're eligible to go into the trial then it's just completely by chance…

Ok.

… whether you have treatment or monitoring.

Or monitoring, ok. Well there's the monitoring. That's like a safety net, the way I see it. Yeah, so maybe, as you said, a mammogram every year.

Yep.

The doctor feels you're at risk maybe earlier… (laughs) but… I would say that's like a safety net, isn't it?

Yeah, so the, the typical approach would be that every year you have a mammogram.

Mmm hmm.

Um, and if that mammogram comes up as suspicious in anyway then you would have further investigation, of course. And as you go along, of course, if you notice any symptoms at any time you would be encouraged to see your doctor to, to see what's going on.

Right.

So does that, does that feel like a…

Yeah.

That does sound like a good safety net.

Yeah.

Besides, besides the fact if you're in a trial your GP, who you'll probably see pretty often, would be aware you're in, being monitoring… probably in his own way just do a general health to see if you're not slipping or sliding.

[LOTS OF MMM's]

But is the, is the surgery, does it take long time or… the patient has to be for a long time in the hospital?

So the surgery itself, um… could take like typically an hour or two, and you could be in hospital for anything between like a day and a week.

Yeah.

So depending on, um… you know, whether it's a lumpectomy or a mastectomy, whether you're having a, um, breast reconstruction that people sometimes have together with mastectomy. So… yeah, it's, um, it's sort of variable. But… that's sort of the time frame for being in hospital. And then it would usually be, you know, it can be quite some weeks recovering from the surgery, where you might not be able to work, um, and do all your normal activities.

Mmm.

Way I see it the odds are in your favour. Because if you can avoid the surgery, which not everybody can, can recuperate that well. Like I know from my experience

I'm a diabetic and I know I wouldn't recuperate that well from surgery. It would take me a very long time.

Mmm.

I say the risk outweighs itself. You know, it's great. I could avoid the surgery, all better for me.

Mmm hmm.

Yeah, and, um, in the meantime you're helping science to come up with an easier cure for our kids. That's what we have to think of. I mean, I sort of… the other side of life, you know, (laughs) we've got kids and grandkids. You know, we want them to grow up in an environment where there might be a cure or, you know, some miracle. Because cancer's just… overtaking.

Mmm.

It's bad. I know because my mother died of cancer 35 years ago, same age as what I am now. And, um, it's because she never had a, you know, a test. They didn't test for those things those days. You know, that was 35 years ago. I think if she did, maybe they knew it was some kind of cancer, so it came all of a sudden. So that was 35 years ago. Now people know straightaway and hopefully 10 years time, you know, it'll be much easier when somebody, you know, gets diagnosed with, um, DCIS or whatever. Um, they'll say, hey, no problem, you know, (UNCLEAR), you know, pop this pill and you're away, you know? (Laughs) That's what we hope for at least for our kids and grandkids.

Mmm.

Yeah.

Or a vaccine like, er…

(UNCLEAR)

… cervix. Yeah.

Cervix cancer.

Find a vaccine, yeah.

That's right. It's all in the future.

Ok, great well that's all very, um, helpful discussion. So we're getting close to the end so anymore kind of, er, final thoughts that you want to… or, or anything that we haven't covered to do with the topic that you want to say at this point? Um, well then the last thing that we'd like you to do today is one more questionnaire, this one is questionnaire two. Um, before you start, I just want to say thank you so much for coming along, we really appreciate it! Um, so after you've done the questionnaire, you're free to go. Um, and on your way out, we will give you your gift card as a little thank you for come.

Thank you.

And we just need you to sign our sheet to say that you've received it. So just pop up to the girl at the back when you're, when you're ready.

I think this has been interesting but, I mean, I had no idea about any of this.

No, I didn't either.

So, you know, as women, um… it's so important to know more about this.

Mmm hmm.

I head about trials, you know, like skin problems they go to trials. But, er, I haven't heard of, er, this cancer trials.

Mmm.

[TRANSCRIPT ENDS 01:23:48]

END

Interview: DCIS FG2 2018.05.18

Interviewer: **BOLD TEXT**
Interviewee: PLAIN TEXT
Pauses: …
Unclear words: (?)

[TRANSCRIPT BEGINS 00:14:33]

So as I said, obviously, um, DCIS is probably quite unfamiliar to most of you, and I'm going to start by talking about something that probably is at least a little bit more familiar, and is relevant to DCIS, which is breast cancer screening.

So what is breast cancer screening? Well in Australia we have a national program that provides screening, free of charge to women, to look for early signs of breast cancer.

Now it's important to understand that when we talk about screening it's something that's designed for women of a certain age in the general population who are well. Um, it's not for women who have a breast symptom (like a lump or pain or discharge) that they've noticed that needs investigation – that's a separate process.

So the aim of screening is to find women who have cancer early, so they can be offered treatment early, in the hope that earlier treatment will lead to a better outcome. And the main goal of screening ultimately is to reduce the number of women who die from breast cancer.

Now the best available method for breast cancer screening the general population is a procedure called a mammogram, which uses x-rays to make images of the breasts. And the whole idea is that these x-ray images can show up cancers or abnormalities may be so small they can't be felt as a lump.

So if the mammogram images look abnormal or suspicious in any way, the woman gets called back to have extra tests which may include a biopsy. Biopsy means taking, um, using needle to take a small sample of cells from the breast to look at under a microscope, for the purpose of confirming what's going on. So from looking at the small sample of cells under the microscope, a pathologist will either say actually everything's fine, it's a false alarm, or they'll diagnose breast cancer or DCIS.

So DCIS is a condition that can affect the cells inside the breast.

Typically, it doesn't cause any symptoms like a lump that you can feel, so it's generally found through screening. So before we started doing screening in the population, DCIS was hardly ever diagnosed. So in the past, women may have had DCIS but it caused them no symptoms and they didn't have screening, they wouldn't have known about it. Whereas nowadays, now that we have widespread screening the population, um, DCIS has been diagnosed a lot more commonly than it ever was before.

And part of the challenge with this condition is that it's almost like DCIS is a new disease that we've kind of only really discovered through bringing in big population based screening programs, both in Australia and other countries, and that's happened from about the 1990s. And so everything that women are told when they're diagnosed with DCIS, and everything we're telling you today, is the best information we can give based on current scientific understanding about DCIS. But it's important to understand that that knowledge is currently limited. So there's still some things we don't really fully understand about it.

So I'm going to show you a diagram with some numbers now – don't worry too much about the numbers, it's just to really give you an idea of how many women are affected by DCIS.

So if you imagine that each one of these dots represents one woman. If we take 1000 women in the Australian population who have breast screening over a period of 25 years, in total about 81of the women will receive a diagnosis of one kind or another, as a direct result of the screening mammogram and the further investigations that follow.

And if we look more closely at the 81 women, we find that 16 of them have DCIS and the other 65 have what's called invasive breast cancer – and I'm going to explain the difference in a minute. But basically, as you can see, the women with DCIS are a smaller group – so about 1/5 of the total number of women diagnosed through screening.

So let's talk about what is DCIS actually is in a bit more detail, breaking it down word by word. So ductal means it relates to a duct or tube in the

breast that carries milk to the nipple. Carcinoma means the cells show abnormal features similar to cancer.

And 'in situ' means contained in the original place. So the 'in situ' part is very important to defining DCIS. So this illustration shows how DCIS is different from 'invasive' breast cancer, which is really just a more precise term for what we commonly understand breast cancer to be. So in DCIS you have some abnormal cells contained within the duct, whereas in invasive breast cancer some of those cells have started to move outside of the duct into other parts of the breast. So based on what they can see looking at the cells from the biopsy under the microscope the pathologist will diagnose that it's either DCIS or invasive breast cancer.

So DCIS and invasive breast cancer are, um, different but they're related. In DCIS, the abnormal cells are contained within the milk duct and have not spread outside the ducts into the other breast tissue. And as long as those cells remain contained like that, DCIS is not a life-threatening condition at all. But DCIS might develop into invasive breast cancer later on, and if that happens the time frame for it to happen is really variable, so it could take many years or even decades or it could happen more quickly.

In invasive breast cancer, some of the abnormal cells which were previously just inside the ducts have actually taken the next step and spread out into the breast tissue around the ducts, and once that's happened, the cancer could then spread to other parts of the body, which is when you really have a problem.

But it's good to remember that although invasive breast cancer has the potential to be a life-threatening disease, these days the vast majority of women diagnosed with breast cancer will be treated successfully and will survive their cancer.

This slide just shows some of the different phrases that are used to describe DCIS. And none of these are really wrong but it can be a bit confusing for women because you can see DCIS is described both as a type of cancer and as something that's not quite cancer.

So if you haven't heard the term DCIS before, you might have come across

some of these other terms like Stage Zero breast cancer or non-invasive breast cancer or pre- invasive breast cancer.

So what happens after DCIS is diagnosed? Well, as I've said, DCIS itself doesn't affect a woman's health, but it has the potential to turn into invasive breast cancer. Now we have reason to believe that this progression to invasive cancer might not happen for everyone, and I'll explain more about that later. But the bottom line is that right now we can't reliably predict which women with DCIS will get invasive breast cancer or how long it may take before that happens. And that's why virtually every woman diagnosed with DCIS is given treatment, to try and prevent that progression from ever happening. And I'll talk shortly in a more detail about what that treatment involves.

Firstly, um, after that little explanation I just wanted to ask have you ever heard of DCIS before?

[LOTS OF NO's]

I've heard of it but I didn't quite understand exactly what it was.

I've heard the term.

And was that someone you know effected or just…?

No, no. But I work in pathology so…

(Laughs) Oh, that's cheating.

[LAUGHTER]

I, I don't do tests for this though. But I've heard it come, 'cause, um, I work in hematology and we do stuff with, um, close, quite closely to pentalogy and oncology clinics. So I've heard the term but I didn't actually… really know exactly what it was, so…

Ok.

Is it too early for questions?

No.

What's the percentage of… so that, the diagram was good, 16 women gave a good idea what kind of, er, you know, scale we're looking at. But what percentage of Australian women do get, er, DCIS?

Well, um, in terms of numbers, um… it's about between 1600 and 2000 women each year in, in Australia.

[A FEW MMM's]

Right.

That's a lot, isn't it?

Um… but yeah, the percentage, in terms of the diagnosis that comes through screening, it's about 20-25% of those.

Mmm hmm.

Oh.

So I haven't exactly got the percentage you asked for but, um…

Yep, 25% of that?

Yeah, it's… so it's, um…

Well, it's 25% of…

It's about a quarter… sorry. It's about a quarter of the 81, the 81 people?

Yeah, yeah.

Which is about right.

So it's, it's… a few percent.

Mmm hmm.

It's not a… it's, it's less common than, you know, normal breast cancer. Um, yeah.

Ok, thank you.

Ok. So do you feel you understand what I've said so far?

[LOTS OF YES's]

Anything unclear or confusing about it?

[A FEW NO's]

Ok. Um, so have you ever heard the idea that some people are diagnosed through screening, and have treatment, for something that may not have ever become life-threatening?

I wasn't… have I heard of someone's who's kind of done it and there was nothing wrong with them, so it was all good. Is that what you mean?

Well, it's not that there's nothing wrong, but there's, there's… what they have is not actually going to be dangerous to them.

Oh, ok.

[TALKING TOGETHER]

Preventative, yeah.

It's kind of benign.

I've had a friend that to have this.

It's preventative, yes.

I had a cyst removed under my arm not long ago.

Mmm hmm.

I had a major operation to get it removed so the… but it wasn't life threatening to me. It was just annoying. So they did that sort of thing, so… but, yes, it was huge sort of thing so…

Mmm hmm.

… yeah, so sort of like it wasn't life-threatening but they still took it out sort of thing. So it wasn't cancer but it was… yeah, so that's sort of, that's not invasive.

You know it's not going to kill you sort of thing so…

Yep, sure.

Yeah.

Ok.

So it's sort of preventative?

Yeah, that's, that's a way of thinking about it.

Yeah, I've had one friend who actually was, er, felt lumps. Well she… (UNCEALR)… felt lumps and she went to have it checked and so they did. And then they went, well, came back abnormal. So then they did the biopsy and they said well, it seems we have to get it out but she actually had like a sponge, like, it was like a sponge almost in her breast. You could actually feel it and see it, and it was actually starting to spread, the sponge, this thing. It was like it was giving birth to little bits.

Mmm.

And they took her, I took her in and brought her back. And we were lucky that it was actually all benign. And she healed up within a matter of a couple of weeks. And she's had no problems with her breasts since; no lumps, no pain. 'Cause she had pain and everything as well.

[A FEW MMM's]

Yeah, so it was, yeah, it was good that she got it done.

Mmm hmm.

Yeah, I've had quite a few lumps. I've had one biopsy. But that was all through me finding myself, not through screening.

Mmm hmm.

And I've been lucky that there's never, that everything's been benign. I've had cysts and lypoma. Um, I had fibroid adenoma. But yeah, so… fingers crossed it stays that way (laughs).

Yeah.

Ok. So what are your, what are your thoughts and feelings about just what you've learned so far about DCIS?

So you can have DCIS but it, it might not turn into cancer. So it's just floating around in there sort of thing but it mightn't turn cancerous for you. Is that right?

That's… yeah.

Yeah. Mmm.

Unless it moves out of your duct.

Yep.

Yes.

Ok.

So I think it's good that screening will actually find it. You said you can't feel it like a lump or anything like that, so there is potential for it to become life threatening.

Mmm.

So if you do, can be screened for it and find it and act upon it before it gets that way…

Mmm.

… then the screening's a really good process.

Do other people agree with that?

[LOTS OF YEAH's and MMM's]

I think there's a level of, um, with more screening and tests you have for a lot of health issues, more things are known. Um, and so there is all this, oh, I have that question of to… you know, does, does it always develop into something or not? You know, but, um… yeah, how you've explained it and so, yeah, it's the right thing to (laughs) get tested and have some, you know, treatment.

But scary. It's a scary thought.

I have never had a breast screen. But I'd had, um, it done… ultrasounds.

Mmm hmm.

Because I had problems, but it ended up my breasts were too big for my body size apparently (laughs). So I had to go on a diet so my breasts could go smaller.

[LAUGHTER]

Can it, can it be screened with ultrasound? 'Cause like most of my things were found, found out having ultrasound first because I've got thick breast tissue. And then I've had mammograms.

Mmm, 'cause they're not easy to get mammogram. That's my issue, is that mammograms aren't easy to get.

They're not easy to get? Huh? That you find them not easy to get?

I often don't find them easy to get 'cause I've got to find, you know, where the Breast Screen, Breast Screen…

Oh.

… place is and then I've got to find, make my way there. But there's the access to it's quite difficult, I feel.

Mmm.

And I feel there should be far better access 'cause the doctor says, oh yeah, go, just ring up the Breast Screen. Go online and ring up, you know? Google Breast Screen online, sort it out with them. It's like, you know… go to them 'cause we don't care, we're not interested. And they're not all that accessible, I'm… they are not accessible. 'Cause even if you go there, they're only available at certain times. You've got to have appointments. So the rigmarole, um, involved just to get to have one… 'cause I was living rural, so they were like well, you know, we don't have any free services here so unless you want to pay a couple of hundred we're going to… but we can do an ultrasound for free. So that's my issue on them.

Ok. Yeah. I mean in, in theory the Breast Screen program is meant to be

accessible to everyone but obviously…

It's not.

… people in rural, er, locations always have a bit of a harder time. Um, in answer to your question, um, about ultrasound, um… we know that for the, for the general population women 50 plus, um, mammography is, is the best screening tool. But ultrasound is often used in conjunction. So if, um, a woman is recalled after her mammogram, um, some of the, you know, the extra tests they'll do will include ultrasound. And then there are some women who for various reasons ultrasound works better for them, um, and particularly if younger women, er… are, you know, need to have breast investigation, breast imaging, um, and that's to do with the density of the breast tissue and some, some things like that. But yeah, basically, um, both mammograms and ultrasound can potentially pick up DCIS.

Mmm.

Ok. Well I'm going to tell you a little bit more about how DCIS is, is treated. So the goal of treating DCIS is to prevent the abnormal cells from turning into invasive cancer. And overall it seems that our current treatments are doing a pretty good job of achieving this goal.

Now surgery is really the primary form of treatment for DCIS. So that's doing an operation on the breast, in hospital, under general anaesthetic. And doing surgery also means the pathologists get another chance to look at these cells under the microscope, but this time they've got more to work with because they're looking at what's hopefully the whole abnormal area of the breast, not just that small sample they got from the biopsy. And in most cases this will confirm that it really is just DCIS as the biopsy suggested. But in some cases they find after surgery that the patient actually has invasive breast cancer, um, that the biopsy didn't catch. So that patient's diagnosis will be changed or upgraded at that point.

Now there are different types of surgery that, um, a patient can have, and surgery may also be combined with another type of treatment. And exactly what each patient has will depend on features of her DCIS (such as size and grade – which I'll talk more about grade later on) as well as characteristics

of the woman herself (like her age, her overall health, and her preferences).

So I'm going to tell you in a bit more detail about the 2 most common options for treating DCIS. And both of these approaches are considered very effective, um, and survival rates for DCIS are excellent.

So treatment for DCIS most commonly involves breast conserving surgery and then radiotherapy. This means having an operation to remove the part of the breast affected by DCIS and the small area of healthy breast tissue surrounding it. And this may also be called a lumpectomy or wide local excision.

And after breast conserving surgery, um, a pathologist will look at the breast tissue that was removed. And sometimes, if they find there's a bit more disease than they originally thought, the woman may need more surgery.

Now everyone responds differently to surgery. Some side effects happen to most people, others happen only occasionally. And some side effects happen straight after surgery, whereas others may take longer to develop. But some of the common side effects include things like pain, numbness, and bruising around the wound.

And after this surgery, the patient has radiotherapy, normally every day for a few weeks. Radiotherapy uses x-rays to destroy any abnormal cells that may be left in the breast.

Now receiving radiotherapy is painless. But women may experience some side effects. So during the radiotherapy treatment, the skin of the breast can become red and dry, a bit like sunburn, or the skin can become darker; and women often feel more tired than usual. And these side effects usually settle down after treatment finishes and things get back to normal.

During breast radiotherapy, it's possible that a small dose of radiation may reach the heart or lungs, depending exactly where the DCIS is located. And some studies have suggested that women who had breast radiotherapy in the past may have a slightly increased chance of having a heart problem, like a heart attack, later on in life. So doctors these days are using more

modern techniques when giving radiotherapy to try and minimise this problem as much as they can.

Now instead of having breast conserving surgery plus radiotherapy, sometimes a mastectomy may be recommended, for example if the area of DCIS is quite large compared to the total size of the breast or for some other reason. This is a bigger operation which means there's a higher chance of complications and a longer recovery time.

All the side effects I mentioned for breast conserving surgery are also relevant for mastectomy but because this surgery is more extensive, there may be additional side effects such as stiffness in the arm or shoulder.

Now obviously mastectomy affects the body shape and it can affect women's body image or how they feel about their body. And many women have the possibility to consider breast reconstruction surgery to try and help address that.

Now just in case you're wondering at this point I want to mention that chemotherapy is not a treatment that's generally, um, used for DCIS. So the main treatments are, as I've outlined: the different types of surgery and radiotherapy.

So what are your thoughts and feelings about that information about treatment for DCIS?

Well my issue is the fact that having had a number of, er, female friends all over 50 who've all had the, um, problems with breast cancer. They've gone through all this and ended up needing, um, single or double mastectomies. The hard part for them is being, they've been part of the various cancer and breast cancer clinics and the downside has been A) that, um, unless you can afford $9000-$12000, um, these poor women actually sit and rot, their breasts literally rot, with, er, cancer on a waiting list.

Mmm, mmm.

And the agony they go through, the suffering they go through, so to say, oh, you can get reconstructive surgery, the problem is that the reconstructive surgery is, um, not a given unless you can come up for the money or you don't mind waiting

and suffering with, um, and watching more and more, um, er, chunks coming up through your… I've actually seen it. I've seen people going in, and a number of years I've been a carer, so I've seen a lot of all this. And watching these poor ladies who have got covered in rashes. They're so embarrassed. They have problems, they can't leave the car, they can't stand up and they're literally rotting. Their whole chest is rotting.

Yes, so the…

And that's an issue which I still haven't, there's nothing been said about, um, instead of saying yeah, we've got this, this is what's available, however you need to have the money for it. Because, um, because main thing they seem, er, for my friends, experience wise, they've all been told yeah, well when you get the money you can have it done, you'll be right, you'll get over it. That's still been quite a common, um… comment made to women over 50 because the fact that we're older, so who cares?

Mmm.

Do you know what I mean? So…

Yeah, so the, the…

… sorry, I don't mean to be a downer. But that's…

No, an important, um, consequence of what we're talking about, consequences of a diagnosis…

… especially being over 50. Mmm…

… and treatment, there's, you know, there's the physical side, there's the emotional side…

Yes. And the self esteem.

… of course there's the financial side… (UNCLEAR)

And the self esteem. And the pain and the agony.

Ok.

So is that part of the treatment where you were saying… where the radiation can change the colour of the skin, is that, is the skin deteriorating… of the breast?

Well the… it's, I guess it's basically a, it's kind of a burning effect.

Oh.

Yeah, Mum had it. Mum had radiation down here and it burnt it. It looked like she'd put her hand, put her tummy on an element (UNCLEAR)…

Mmm.

… it was… left a hole.

Did it go away?

She passed.

Oh.

She didn't make it through so.

Oh.

Yeah, so…

Yeah, so, um… it can look quite dramatic at the time and in, in most cases it will then recover, although…

Oh, it does recover.

… um… maybe in the occasional case there would be kind of lasting visual, um… changes. Um…

Like, um, the lady downstairs has just had radiotherapy 'cause she got cervical cancer. She's 60 odd sort of thing, and she's had to go back into hospital for, um… what do they call it? Um… not rehabilitation but, you know, like… care sort of thing 'cause she was so sick after it sort of thing. It just sort of just made her really sick, and she just couldn't cope on her own, couldn't eat, couldn't do anything and she said she, her sight was going a little bit and, you know, whatnot sort of thing. So they are, they are side effects of radiotherapy, aren't they?

Yeah, I mean I think with all of these things there's always, um, quite a range of possible side effects.

Side effects.

And people are effected differently. But yeah, that, um…

I mean the treatment's quite the same as if it is breast cancer, isn't it?

Yeah.

You know, your treatment options except for not having the chemo, um, I suppose that that's, the piece is your still making those same decisions about, um, yeah, which, which type of surgery or whatever. Um…

I guess if you catch it earlier it's likely to be, it's like to be smaller so that you… a lumpectomy probably…

Suffice.

… suffice with the radiation, rather than having the full mastectomy?

Um, yeah, I mean, er, if it's DCIS it's sort of by definition always early.

[A FEW MMM's]

Um… so whether the DCIS is in one duct or multiple ducts, um…

Right, ok.

… it's, you know, it's not that it necessarily… progresses as DCIS into more places…

Yeah, yeah, yeah.

… but, but yeah, the, um… yeah, so it's more, it's more kind of the, um, well there's various features of it that can, that can sort of make a clinician lean towards suggesting the mastectomy rather than lumpectomy. Um, and sometimes that's sort of patient factors as well.

Ok.

You said that, um, most cases of DCIS they go through this whole treatment process. Is there any data on, on people, the numbers that do elect to do that and those that wait and see if it gets any, if it becomes invasive?

Ok, that's a good question. Um…

[A FEW YEAH's]

… we're going to talk a bit about that.

[LAUGHTER]

But the short answer is, um, at the moment it's, it's very rare to not have any treatment. Um, so unless the woman is considered, you know, not fit for surgery, like if she's perhaps quite elderly and quite unwell, um, the, the standard recommendation is to have some kind of surgery and other treatment.

Mmm.

What are some of the, um, you know, what are some of the common reoccurring features of, um, the background of the women who do get DCIS? Um, are they, er, women who have, um, you know, have two or three children? I mean some, sometimes like cervical cancer, that's a case of a certain age, um…

Yeah, so, um, there are some risk factors that we know are associated with breast cancer and DCIS is the same. So, um… so for example, I mean the main one is just getting older. So as you get older the risk is higher.

Aging. Mmm hmm.

Um… women who, um, had children at younger ages have a slightly reduced chance of breast cancer.

Mmm hmm.

Um… and other things that can increase your risk, um, are things like gaining a lot of weight in the sort of post-menopausal part of life.

Mmm hmm.

Um… er… drinking a lot of alcohol. So things like maintaining a healthy weight, being physically active, um, not smoking, your kind of standard lifestyle things are thought to be good. Um…

Mmm hmm.

… breast-feeding can reduce your risk as well.

That's, that's the one I was going to ask, yeah.

That's right. See if there was a correlation because of, um, women who breast-fed, because it's…

So women who breast-fed have a, have a lower chance of getting breast cancer. Um, and then of course…

Mmm.

Yeah.

… there's, um…

'Cause there's less chance of (?) 'cause you're breast-feeding. 'Cause I breast fed all four of my children, so it's like, um…

Of course, um…

… still happens though.

… yeah, so, so none of the, you know, these things are kind of making small differences, um, mostly it's just bad luck really.

Oh really?

Yeah…

So it's chance?

Yeah.

So it's not (UNCLEAR)… really where I was going.

Is it dietary? 'Cause you think about obesity, so…

Yeah, so other than, as I said, kind of being a healthy weight and being physically active, we don't really have much, um… evidence of, of strong dietary and nutritional…

Mmm hmm.

What about environment then? You know, like women in the city more susceptible to it than women in the country or… you know?

I don't think so.

I don't think so, no.

So there's no… they can't prove that sort of thing.

Smokers.

Yeah.

I would call smokers, where… (UNCLEAR)

[LAUGHTER]

Yeah, I mean I think smoking is, is one of those, er… again it's not been seen to be a particularly strong association with this type of cancer.

Oh.

Of course there are other certain cancers that are very strongly linked to smoking. Um, whereas breast cancer it's, it's a bit less directly.

What about women in high stressed jobs? Is there any evidence?

No, there's no evidence that stress, um…

Causes breast cancer.

Mmm. Oh, there's not?

It's mostly genetic, isn't it?

Well there is, there is a genetic aspect. Um, so… in, in some family there's a genetic aspect. But most women who get breast cancer actually don't have a family history.

But…

(UNCLEAR)…

… sorry.

Oh, sorry. Er, for the work… did Angelina Jolie, er…

I was thinking of her (laughs).

… do that just because she, she said she got the genes in her… you know, because her mum got that and then she got that, that's why she did that operation?

[A FEW YEAH's]

That's right. So Angelina Jolie is one of a quite small proportion of the population who do have a specific…

Gene. Mmm.

… genetic mutation that puts them at very high risk.

And there are families where you see it on both sides, on (?), those genes. 'Cause I come from an all girl family so that's one thing that's not in our family history of going any way, and we're all most of us large breasted women as well. And with weight gain, etc., but, um, you find sort of like, I've met people where it's in the family and you're actually that almost all of them get it; their daughters get it, you know, even the cousins in the same family tree get it.

Mmm.

So actually our Aunties get it… it's actually quite common, and that would be scary. I would probably consider doing it myself.

Er, what, what year was the first woman diagnosed with breast cancer?

[LAUGHTER]

Oh, that's an interesting one?

Probably a very long time ago. I mean I think they've…

Rembrandt's paintings.

… yeah, you see in some old paintings, um…

That's what they say, yeah, that the breast lumps.

[TALKING TOGETHER]

Renaissance.

The Botticelli's Venus.

Yeah, so it's…

It's been around.

… it's, yeah.

But they actually weren't diagnosed though?

No.

[MUMBLES]

They would have had it and…

Well it's like syphilis diagnosis and…

… so the diagnosis is only when you had the equipment to be able to… or knowledge to be able to.

Yeah, they wouldn't have had a clue.

They wouldn't have known.

(Pour?) money in a doctor.

[LAUGHTER]

Yeah, so in terms of the, the detection through the imagining like

mammography, the first mammography was done in around… the 19…

Yeah, in the 20th century.

… 50's, the 60's. That's when they started testing out that kind of, um, procedure for, for screening purposes. Yeah. Once, once they had x-rays.

Yeah.

Ok. Um, good. How is everyone doing?

[MUMBLES]

Is it just me or is it quite warm?

It's hot, yeah.

[LOTS OF YEAH's]

[TALKING TOGETHER]

I'm just right (laughs).

We'll all be red-faced coming out.

Um, no but I'm feeling… (?)…

Oh, but you're… when are you due?

Yeah, I'm due in July.

Oh, ok. Cool.

First? Second?

First, yeah.

First? Oh. Boy or girl? You've found out?

[LAUGHTER and TALKING]

Ok, um… all right, so we'll, we'll move on to an important question that is currently occupying DCIS experts. And that is could some women with DCIS

actually avoid all this treatment?

That's what someone else said, didn't they?

[MUMBLES]

'Cause it's very invasive all of that.

Yeah.

Why go through it if you don't have to? But if you don't have any data on it…

Mmm.

[A FEW MMM's]

Yeah, yeah, 'cause I think… your personality will sort of, um, determine how you react to the, to the news. So if you're, you know, someone might think, oh well, I'll just, I'll just leave it. And other people think, get that thing out! You know, or you know, let's get, get onto that straightaway and suffer the consequences of all of the treatment.

Yes.

To do…

To do or not to do.

… to not have that, yeah. So I think, I don't know, maybe the support you've got, the information you've got…

Yeah.

… um, the, the money you've got, that might effect how you get, what you do with the news.

Mmm, that's right.

It will effect it actually.

Yeah, so I mean the current treatment for DCIS is based on our best scientific knowledge and of course wanting to do what's in the best

interests of patients.

Um, and as I said before, survival rates for DCIS are excellent. But because many patients do experience side effects, um, and this includes both physical effects on their bodies, and emotional or psychological effects as well, um, and they affect women in the short term and in the long term.

So breast cancer experts are now doing research to try and identify women who have DCIS but have a low chance of getting invasive breast cancer so that in the future, women like this may be able to avoid surgery and other treatments…

And have more choices.

… yeah, and therefore avoid all those problems that can come along with treatment, while still having excellent survival rates.

Now as I said before, it's very difficult to reliably tell who these 'lower risk' patients are, but we do have some clues. And one of them is something I mentioned earlier called grade.

So after the biopsy is taken, the pathologist looks at the sample under the microscope and gives that case of DCIS a grade based on how the cells look, which gives an idea of how fast the abnormal cells are likely to grow.

So low grade indicates that the cells appear to have quite a lower level of activity, which suggests they may stay like that for quite a long time. On the other hand, high grade DCIS means the cells appear more active, so they're considered likely to grow at a faster rate. So it's considered important to treat high grade DCIS because otherwise it's pretty likely to develop into invasive breast cancer at some point. And then there's an intermediate grade that's somewhere in the middle.

Now you might be wondering, if virtually all women with DCIS are having treatment, as I said, why am I saying that we think low grade DCIS may stay as it is for many years and not cause problems, even without treatment.

Well, we think that based on a small number of research studies similar to this example.

So I want to tell you a bit about a study that was done in America using a database of routinely collected information about cases of DCIS. So these blue bars represent all the patients in the database who had low grade DCIS. And as you can see, the vast majority of these patients, almost 9000, were treated with surgery, which is the typical thing to do for DCIS, that's recommended in all the guidelines for doctors.

But a small number of patients, a couple of hundred, didn't have surgery for some reason, and we don't really know why not.

So this database kept track of key events that happened to these women over many years, for example it recorded if any patients died from breast cancer. And what the researchers did was compare the larger group of women who had surgery and the smaller group of women who, after about 10 years. And they found that the women who did have treatment had a very high survival rate, so 99% of them had not died of breast cancer, so they were doing really well. And the research also found that among the women who did not have treatment, 99% of them also had not died of breast cancer.

Oh.

Or needed surgery.

Mmm.

Yeah, so they hadn't had surgery and they still hadn't died from breast cancer. So this led the researchers to suggest that there may be a subgroup of women with DCIS who are likely to have good health outcomes over time, whether they do or don't have the typical treatment of immediate surgery.

[A FEW MMM's]

Do they do like retesting and more frequent testing? Like is, is… you know, 'cause it sounds like you make your decision and then it's done and dusted (laughs) versus, you know, you, you… are they monitoring any patients who opt for the like no, no surgery and…?

So this, that study was really, um, something that they did just looking back…

Yeah.

… at what patients had done in the real world in this database. So it wasn't designed in the beginning to, you know, make this particular comparison, or to…

Yeah, probably I'm meaning more, um, is there another option that patients are given, or not at the moment, the options are just the surgery that was talked about originally?

So basically it's…

Sort of like come back in 6 months and (laughs) test again and see whether it's changed.

Yeah.

Not really.

Ok.

It's not really an option that, um, clinicians feel comfortable to offer at this point in time.

Oh.

Mmm.

So this idea of a subgroup of DCIS that we could call 'low risk DCIS' is now a big focus of new research that is designed to try and investigate this properly, so whether women in that category could possibly avoid treatment.

And there's a range of factors that, that are relevant to trying to define this category of DCIS – so grade is an important one but it's not the only thing. There's lots of other factors that are taken into account that I'm not going into.

So did, sorry, so just from that diagram, the, they pick up that it's more active and faster growing, they can just pick that up from the cells that they test?

Yeah, so it's, it's, er, an indication of, um… you know, they pick up the grade, the grade is based on how it looks visually. And… it's considered to indicate what the activity will, is likely to do over time.

Ok.

So coming back to this little diagram I just want to give you a rough idea of how many women with DCIS might be classified into this sort of lower risk category. So of our 16 women with DCIS from before… we think around about 5 women have what could be classified as lower risk DCIS based on their biopsy.

[A FEW MMM's]

So for the other 11 they're considered to have a higher chance of getting invasive breast cancer at some point so it's still considered more appropriate that those women do have treatment.

So these 'lower risk' women are, as I said, the focus of current research to try and look at whether they might be able to avoid treatment. And I'm going to tell you more about that research now, and so it's just important to remember that we're talking about this quite small and select group within DCIS patient population where researchers are considering a new, different approach to managing their condition.

Mmm.

Any burning questions right now? Ok.

So the type of studies I want to talk about now are called clinical trials, so let me explain what they are.

A clinical trial is a research study that asks an important, specific question about a certain health condition. And clinical trials often test new ways to manage a condition, by comparing a newer approach with whatever is the current standard treatment.

And trials are designed to find out whether patient outcomes (such as health and quality of life) are similar for both options, or whether one option is better than the other.

Now if you think back to that study we talked about before, with the blue bar graph, it was done by looking at a database containing routinely collected basic information about patients from the past. And although studies like that can be valuable for giving us interesting ideas, it's hard to draw firm conclusions from that kind of research because there's a lot of important information missing, for example details about those patients who unusually did not have surgery and exactly why.

Whereas clinical trials are specially designed to overcome the limitations of other types of studies. So that's why trials are considered to give us the highest quality of evidence.

So… this illustrates the basic design of a simple clinical trial. Sometimes they can be more complicated, for example with more than 2 groups, but most typically, each patient in the trial is put into 1 of 2 groups. One group of patients gets the standard treatment, while the other group gets a new treatment or a new way of managing their condition. Now which group each patient goes into is determined by chance, using a computer. This process is called randomisation and it's a very important part of clinical trials. When we compare 2 groups of patients having their condition treated or managed in different ways, we want to make sure the groups are very much the same except for the way their condition is treated or managed. If patients and their doctors choose which treatment to have, then the patients in the different groups are very likely to be different from each other in important ways that are relevant to their health, which can create a bias that makes the results less reliable. So patients who volunteer to take part in the trial need to be comfortable with ending up in either of the groups because they don't get to choose which one they're in. Randomisation makes sure that the study groups are as similar as possible. And that way, we can get the most reliable information from the results of the trial to learn about the effects of the different ways of managing the condition.

Now deciding by chance how a patient will be managed may seem strange, but it's considered an ethical thing to do in situations where we genuinely don't know whether one option is better than the other.

So there are currently 3 clinical trials going on around the world for women with low risk DCIS: one in the UK, one in Europe based in the Netherlands, and one in the US. They all just started in the last couple of years, and together they will involve over 3000 women. Now there are slight differences between the trials, but they all have the same general design.

And they're to learn whether women with low risk DCIS could safely avoid having treatments that may not be necessary for them.

So the women invited to take part in these trials have to meet strict criteria to be considered suitable. They have to have low or intermediate grade DCIS (not high grade), they can't have any lump you can feel, plus there's a lot of other more technical rules I won't go into. So remember, this is what typically happens to women with low risk DCIS: they have treatment pretty much straight away, and we talked about those treatments before.

Um, of course in the following months and years, if it seems their initial treatment didn't work as well as hoped, or their DCIS comes back, or they get DCIS again elsewhere or invasive breast cancer, they might, um, also end up having some further treatment if needed.

So that's the normal scenario for pretty much all women in places like Australia where there is no clinical trial. For women who do have a trial available where they live, if they're suitable to offer the trial, they may just say no thank you, I'll stick with usual treatment. Or, if they decide to join the trial... they get randomised to either usual treatment or the other group called active monitoring. So the usual treatment group of course has usual treatment. And the other group has a different approach where in the first instance they don't have surgery or radiotherapy. And then patients in both groups are monitored closely, so they all have mammograms at least every year. But importantly, patients in the monitoring group may have treatment later on if there's any sign that their DCIS is progressing, and treatment seems necessary. So this approach may be called active

monitoring or active surveillance and it's actually already an accepted option for men with early prostate cancer because trials similar to this have shown that it's a safe option. So these trials will test out the idea that this monitoring approach where you only give treatment if it's needed may result in the same very high survival rates as standard immediate treatment, but with fewer side effects overall because some women will avoid treatment altogether, and others will end up having treatment down the track but it's delayed so they may have a period of some years where they're free of those treatment effects. So those are potential advantages of monitoring.

There's also potential disadvantages the trials will examine. For example, patients who don't have immediate treatment may be more likely to get invasive breast cancer – although with the monitoring of course it's still likely to be picked up at an early stage. As I mentioned before, it's also possible that some women who are diagnosed with DCIS based on their biopsy actually have invasive cancer at the time that the biopsy didn't pick up. We also don't really know how women having monitoring will feel about not having treatment, so in the trials the patients will be doing questionnaires to help the researchers understand the psychological effects of this approach, and that's a very important aspect.

Now because there's only that small and select group of women who are eligible for these trials, and because they then need to be followed up for quite a few years, it will still be some years before we get any results from these trials.

In the meantime, it's also possible that a similar study could be started in Australia and New Zealand, so women here might one day have the possibility to join such a trial.

But otherwise until the results of these trials come out, women with DCIS will continue to have treatment as normal because we don't have strong research evidence yet to show that any other approach, like monitoring, is really safe.

So what are your thoughts and feelings about these clinical trials?

What are the time frames for them? When are they expecting… to present their findings?

Well, um… they all started between 2014 and 2017.

Ok.

And they typically involve about 10 years of follow up, so they may publish some, you know, earlier findings in, in a few years. But then it would still be probably a decade before the definitive results.

We might be coming back to the next market research on it.

So the monitoring, just the monitoring and the blah blah blah blah blah, you know, does it, when you say monitor it do they constantly go to the clinic and, and get tested and dah dah dah dah dah dah, but just no treatment sort of thing? But you constantly monitor them like once a year or twice a year or…?

Yeah, so typically once a year they'll have a mammogram. Um, I think in the American study it may be twice a year. And of course those women are encouraged, you know, if they notice any symptoms at any time they should go and get them checked out.

Yeah, yeah.

But, yeah, basically the protocol is, um, typically once a year to have a mammogram. And if that mammogram shows up anything then they'll have further investigations and as we said they'll have treatment if it, er, if it seems like there's a need for it.

So the women, when they I guess give their consent to go in the trial, they know what the two pathways are?

Yeah.

Yes.

So how's that any different from just having a group of women that nominate not to have treatment and monitor them. I guess because you're almost, you've got a group of women who are prepared to… you, you only go in it if you're prepared

to not have any treatment?

Yep.

So how, how's that different then through a clinical trial than just allowing it as an option and putting a monitoring program in place?

Well, um… it's different because… if you, um… yeah, so as you said, in the trial all the women at the beginning are, have said they're willing to have either. So that means that after you randomize them, um… that the two groups are really similar in not only ways that we can measure and know to look for but also in ways that we might not even realise are important. Um, whereas if we were comparing women who felt comfortable to have monitoring and women who prefer to have treatment, um, we can… we might be able to say oh, they were similar ages and similar type of diagnosis, but there's going to be something about them probably that means that, er, the effects you find in them are not directly comparable.

So they're false. It'd be a negative…

It's a, it's a bias, yes.

It'd be a negative, yeah. Mmm.

I suppose when you're comparing the two groups but I mean you wouldn't end up in the trial and have the treatment though, you could, well you could well say that if you compare them to a group that, another group that opted for the treatment, they would be different is what you're saying. Like they're mental approach to their, to their… their illness could be quite different.

Yeah.

Um, yeah, ok.

Yeah.

Like the people who think they are in the control room, they, they think that they're ok and all this stuff, but if they like, you know, the drug trial and the one they at, they thought they are in the trial group and they see that they've got a lot of side effects, that's a psychological…

Well a drug trial you don't know, because they're…

[TALKING TOGETHER]

How you going to know? Yeah.

But unless you're going to know which group you're in.

Yeah, that's why I find it a little bit, I'm just curious, it's a little bit different.

But it'd have to be a personal decision, wouldn't it? It's like, nah, I'm not going to go through the radiotherapy, I'm not going to do that to myself. So it'd just be a personal decision for just…

Well for me, I was diagnosed as being actually low-risk. Then I, I… well it's more my age, so I would actually look it and say, ok, low-risk. So I, I would want to be able to say, well can I, like the pap smears, if nothing was wrong you have them done every 6 months or every year. But if you had, um, if they were finding stuff then they'd do them more regularly. But it's all changed again now. But the thing is I'd want the same on that, where ok, if I'm low then I really, you know, and you've taken…

Yeah, you'd think about it.

… yeah, I'd want to think about it too.

Yeah.

And if it progressed, then I'd want to have it looked at. But…

The interesting thing is if you opted…

Yes, to not have any treatment.

… yes, you actually in that trial could get put and have to have the treatment.

And I don't, I don't like that.

Which is interesting.

That's like saying, well we've picked you for this section…

So only certain people are going to go… yes, so…

Yes, yes. People who have nothing to loose probably.

Well not necessarily.

No.

People have different, um…

Yeah.

… thoughts and approaches and…

Yeah.

… it's like what you said, when a personality…

Yeah, like natural childbirth, um, versus, um, my specialist will do a caesarian 'cause it's easier (laughs).

So this group's basically saying that they're happy to do either and they have to be happy to do that.

Yeah, that's right, yeah.

And then they just get allocated what they…

[TALKING TOGETHER]

Is there a reason why Australia doesn't have trials?

Yeah.

It seems so strange that, you know, Australia's the first to put money in to trials and clinics and is there are reason why Australia's not involved this time around, with America and particularly they UK and…?

They have more money for research. We, we do our research is more on genetics and stuff, isn't it here?

Well, um…

Like Melbourne.

… so… I can tell you that the, the three trials that are happening in different countries, um, you know, the researchers have designed those trials and they've applied for funding from their kind of local bodies and they've been successful. Um… I know that there is a group of people in Australia who have also designed a trial and have been trying to get funding and have, as yet, not…

Oh.

Not successful?

… so, um…

No money.

From a numbers perspective… you know, you're, in Australia you're dealing with much smaller numbers.

Mmm.

It's true. So it would be Australia and New Zealand, but it'd combine…

But still.

Yeah, it's a small population that you'd be targeting and so, as we've said, you've got, um, a smallish population who would be eligible. Then you've got to consider how many…

You've got to be prepared to participate.

… will participate. So that, that comes into deciding whether a trial is feasible.

[LOTS OF MMM's]

Ok, so… so you've kind of, er, said that it would depend on people's personalities, you know, whether they would be willing to go into a trial that was random, where they were going to be randomised.

I think it's more than that. Er, sometimes it's their, their caregivers, it's their clinicians…

Yeah.

… that, who have a real bias or a real…

[LOTS OF MMM's]

Yes.

… definitive view and can…

I agree with that one.

… very strongly influence…

Persuade (laughs).

… a decision one way or the other.

It's like we, we're the ones looking after you so therefore we, we don't want you to have that, or we want you to have that because if you don't then, you know, we're going to watch you get sick and we're going to feel awful. And you could have had this operation and it might have saved your life. So…

It's like with your family saying things like that to you. But, um, your clinician, a lot of them don't even give you alternative options.

No. That's correct.

They say this is what we're going…

Yeah, yeah.

… we'll book you in, you know, off you go. So…

Otherwise die! (Laughs)

Yeah, once again, you're thinking, you know, you're thinking…

[TALKING TOGETHER]

Yes, but then you have to be a fairly confident individual to seek second opinions as well and…

[A LOT OF YEAH's]

Yeah, as well as…

… you've got to get referrals and…

… yeah, as well as how proactive you are about your own health in general.

… all sorts of things, so…

You don't want to go to your doctor and go I don't want… it's not like I'm not listening to you (laughs).

Just one at a time. Sorry, just for the audio recording.

I think in a lot of trials a lot of time people are… very ill and they will go just, yeah, whatever 'cause I'm looking at maybe death anyway. But when you've got something like this where it's really quite unknown I think it's a bit harder option, like a harder decision to make. Um…

I think it might make it easier.

Yeah?

Because the risk is, is really quite low, so the decision to be part of a clinical trial I think for me personally would probably be easier to be part of the clinical trial. Because then, you know, the risk… you know, my life's not a risk… a very huge risk, you know?

Yeah.

I just think it might be easier.

Yeah, I tend to agree.

[LOTS OF YEAH's]

I think the bit that I was struggling with is if, if that's the path I wanted to go then that decision's been taken away from me and forcing me to have actually invasive treatment…

Yeah.

… um, is probably the little bit in this style of trial that I'm…

Well I think…

… I think I would struggle with.

Yeah, so you're not…

But it's not, it's not making the decision, you know… if I chose to make the decision to not… just be actively monitored but then you're forced to have the treatment, I think that is where it's a bit more difficult than, um…

Well I like what, what's your name?

Liz.

Liz, oh, like Liz said, 12 years ago I was actually very, very sick and hospitalized, um… and as a, and loosing weight and they couldn't find what was wrong with me. Took them 3 months, 3, 4 months to find out exactly what was wrong with me. And in that time when they did find out, um, they then approached me in the clinic and said, listen, um… your medical is such and your survival is that low, and I was on 35% survival…

Mmm.

… is what they gave me, and we're wondering because the fact that you're still here sitting in front of that with all that wrong with you, would you like to join, we would like someone like you to join our, um, our, um… um, group, research group. And, um, so I had to have, I had to do questionnaires, blood tests and then they've kept it all the way through. And the great thing is that same particular research group they now, 12 years later, I actually just completed last year their, um, their newest findings and I was cured.

Oh.

So as a consequence of me, but I was that desperate. And the thing was because I was so sick and they were having, Prince of Wales were having problems finding out what was wrong with me, and do extensive tests, I was like, you know, pin cushion all the time. And as a consequence of being part of that I ended up then being a recipient and being cured.

Mmm.

From the original trials I agreed to. They did my DNA test, they did DNA testing in order to find out what was wrong with me.

Mmm.

So yes, that's why I like all this kind of stuff.

[LAUGHTER]

I'm still here (laughs).

Yeah.

So what about some, some people who haven't said much yet? Um… Marie, what do you… what's kind of going through your head about all this?

You keep shaking your head (laughs).

Um… I'd get a, a second opinion.

Mmm hmm.

And, um… I'd weigh up the pros and cons and I'd probably not go into a trial.

Mmm hmm.

Mmm. Really?

Yeah.

But you'd still have to be, um, medically checked constantly.

Oh yeah.

But you wouldn't do a trial?

No, no. 'Cause, because in case you ended up having to have surgery.

Now what would you do?

Yeah, I wouldn't want to be a guinea pig.

See, I love being a guinea pig.

So that, I'd feel like I was a guinea pig in that situation.

Ok.

'Cause you, you might not be getting any treatment at all. And I, I tend to lean towards, you know… I like to be proactive.

So you'd rather have the standard treatment than…

Yeah.

… go into a trial.

Yeah.

Ok. Um… what about you (?)?

Well, I… I think I prefer the conservative treatment. So, er, I can, I don't mind a, a trial, but yeah, if it's low-risk, um, I would go for the trial.

Mmm hmm. Ok.

Um, I'm scared of the knife, under the knife.

[LAUGHTER]

And radiotherapy.

Yeah, radiotherapy's…

Yeah, I would do the same. If it was low-risk I'd get a second opinion and I wouldn't want to risk myself having to go under the knife and radiotherapy, um, unnecessarily.

Ok.

Today though, a second, even a second opinion, would you get any doctor actually saying you don't need treatment? Or what you said earlier on is, with DCIS it is going to be treatment in Australia today pretty much that would be the recommended pathway?

That would be the typical recommendation.

Yep, yep.

So you might find yourself kind of struggling to find a clinician who felt happy to say, ok...

Don't do it.

Let's just watch it.

... we won't... yeah.

Why so conservative though?

Yeah, because someone will come back and sue them for (laughs)... you, you did tell me not to do it.

Yeah, but you've also got, you've got the conservative meaning the older, um, medical people that've been going for X amount of years. I have an older one and I have a young one, and I always have the younger doctor that, er, she goes, so both my doctors I see both go to England regularly, um, to update, um, new findings on medical stuff and so as a consequence I get to have the old, old school conservative with a wealth of knowledge and then the young ones who have got, learnt that and then are actually up to date with the more recent. And, um, because they're from, they do other countries for medical research themselves, so you, yeah...

Yep. Ok.

What would you do?

Um, I like the name, monitoring, active monitoring, I like the name. I think a hands on approach is good and a second opinion is good. I think kind of all active monitoring is good. It's got a nice ring about it for a woman who, you know, need help at this point in time, no matter, you know, what their circumstances are and, um, for myself, you know, I invest everything. But, er, active monitoring sounds good.

Yeah, I think it sounds really good.

Mmm. I mean the, the (known?) view doesn't hurt if you can afford it. I mean, I'm sorry, did I miss but can you get, er, breast cancer fully blown, er, without DCIS?

Um, so…

That's a good question.

… it can, it could either have started as DCIS or something called LCIS, which the L stands for lobular.

Oh.

Which just means it started in the lobe rather than the duct.

[A FEW AH's]

But basically the, the idea is that all invasive breast cancer had some early stage first, when it was in situ but how long that stage lasts could be hugely variable. So in some cases it can progress really quickly and, you know, there's almost no chance to catch it. Um… whereas DCIS that's found through screening is, is kind of representing the other end of the spectrum, where it might be in that early phase for years.

Would you still use the word, you as a professional, would you still use the terminology, er, dormant and benign? Do you still use those terms, in terms of when you…

So yeah, I would use the term… benign, um, if the… pathologist, um, sees that it's… that it's benign, you know, that it's not harmful at all.

See you would understand that more, wouldn't you? Pathology?

Whereas, um…

Do you guys use that… term?

Well malignant or benign.

Yeah, there you go.

Yeah, yeah, so technically DCIS is malignant. Um, it, because it appears,

um… in a way that's similar to cancer.

Mmm hmm.

Um, so whereas it could be dormant, so the word dormant indicates that it might just… um, stay the same for a long time.

Mmm.

Smoldering (laughs).

But the, the whole idea is that it's, um… as apposed to something that's benign, it's something that has the potential to be dangerous.

[A FEW MMM's]

And that's why it's treated as it is.

But then again with that study where they compared the two groups of women and the one who had the treatment, um, they didn't develop cancer, but equally there was also the women who didn't have the treatment…

It was the same…

… and again, they didn't develop. So I guess there's something strong in that, that, you know, you could be really going under the knife, and I guess… I don't know… are we just looking at certainties all along or are we looking, I mean, will we have this evidence that when we compare those two groups that that second group of women, they didn't develop it either?

Mmm.

They didn't develop full-blown cancer.

Hence why they're doing the research I guess.

[A FEW YEAH's]

Which is good for us in the future, for our children, our daughters.

So that's a really good, um, I guess, you know, women having… well I'm not going to say unnecessary because probably a medico would rip my throat if I said that

(laughs). But, you know, maybe not necessarily having to have that immediately, or if there was that monitoring then you've got a better idea of you can make a decision when you need to make a decision. But not necessarily down this… you know, right now. So I don't know.

Mmm.

I'm reminded of the prostate…

Yeah, yeah.

… whole scenario, which you had mentioned. You know, originally it was.. quite invasive, you'd have it removed and all sorts of consequences as a result of that, and now the studies are showing that that's probably not the way to go depending on your age group and all of that.

It's the age group, yeah.

If you're better to monitor it and wait and see, and even the monitoring has, has changed over the years from, you know, going for regular biopsies and how the biopsies are done versus other forms of monitoring. So, um, I don't know. It's hard to know until you're in the situation what you would really do.

[LOTS OF MMM's]

And so much of it does depend on where you are in your life.

Yeah.

I still have a fairly young child.

Yes.

So I would do anything to prolong my life for his sake.

Mmm.

But, you know, if I didn't have a child or if I had older children… maybe my decisions would be different.

[LOTS OF MMM's and YES's]

Well the other thing would be also…

I agree.

… if you were a single parent with a, you know, young children…

Mmm, no family.

… that would be radically… difference in terms of…

Yeah, that's right.

… your decision-making.

That's right. Yeah.

So with the groups, the three studies that are going, are they all a particular age group? Are they all like women over 50?

They're all over at least 40. Um, sort of 40, 45, 46.

Yeah.

And up to whatever, they don't have any…

Ok.

You could wait 10, 15 years for the results of those studies and they'd be totally inconclusive.

They could be (laughs).

[LAUGHTER]

So, yeah.

What about misdiagnosis? That wouldn't happen with trials though?

Well the trials all have quite strict protocols for confirming the diagnosis and confirming the eligibility at the beginning.

Mmm. Mmm hmm.

Um, so, you know, more kind of double checking than is standard…

Mmm.

… to, to confirm that people who are going to be offered the trial are really the right people to be in there.

Mmm. I only ask because of the time factor, the time line between the time that you, you notice something through to, er, getting it diagnosed, through to… um.. you know, um, getting a second opinion and then seeing how, if it, if there's any, you know, vast difference in between what's, what's going on, what's… in the beginning through to, you know, has it spread rapidly or is there a long kind of incubation period?

So these, um, the women with low-risk DCIS, um, in the trials will have their DCIS found on a mammogram. So either in a screening program or, um, you know, kind of an incidental finding 'cause they've had a, a, you know, the… that they've had some kind of scan for some reason.

Mmm hmm.

Um, generally I think if they have any actual symptoms they wouldn't be, er, included in the trial.

Mmm hmm.

So it's only for cases where the DCIS is, um, you know, found on an image and the woman is otherwise well.

There's no lump or anything.

Um… yeah, whereas…

What's, what's the numbers in the trials, do you know how many people are participating in the trials?

So they're all, each of them is aiming to recruit somewhere roughly around 1000 women. Um, and so that will take some time to recruit.

Yep.

Yep.

From the, you know, the low-risk to the high-risk, when they determine do they have a average speed of those growing? Or you just by the look, by the doctor saying they grade it? Yeah, but do they have arbitrary something to say how they grade it?

So they have, um… kind of guidelines for how to, um… grade it, um, that pathologists, kind of, worldwide agree on. But of course, as with all these things, there is some variability between individuals.

Yeah.

So it's not a, a perfect system. It's, um, you know, they're kind of looking at as many things as they can to try and put together and define this group but it's, um… it's all kind of, you know, based on estimates here and there but it's the best they can do.

The, the reason I was asking because, you know, the growth rate, normally is growing (UNCLEAR)… and the, you know, is depends on whether they are still growing like that or they are (?) here or spread there. You know, depends on that as well.

Yes, so I mean part of why this research is needed is because we don't have much information about that. Because the, the kind of, um, response of the medical community to discovering that there's all this DCIS is… well, it might turn into breast cancer we should take it out.

Yes, mmm.

Um, because all of the, you know, all of it's taken out quite quickly…

Yes, no one knows.

… that's why there's little understanding of what it would do…

Yes, yes.

… otherwise.

Mmm, yes. I think the trial is good way of dealing with it, 'cause…

I think so too. I agree.

Those, um, three trials, they've got fairly similar criteria? Like that they're assessing?

Yeah, there's…

So is anybody looking at anything else? Alternative treatment regimes or… not, not alternative medicine…

(LAUGHS)

… like I'm not talking about pick your herbs in the garden and mush it up and plaster it on your forehead. Um, you know…

Like other treatments besides…

… yeah, is, is radiotherapy alone an option rather than surgery and radiotherapy?

Yeah, so there are some different, um… different treatments kind of trials. One of them, one of the tings they look at is whether women could have a lumpectomy and leave out the radiotherapy, for example. So the radiotherapy has been shown to help but, um, you know, can we identify women who don't really need it. So that's kind of one avenue that's also a topic of research. Um, another type of treatment that's being researched for DCIS is referred to as hormonal therapy, so endocrine therapies. And they are used in breast cancer. So for both breast cancer and DCIS it depends, it's only a certain subgroup of cases that, where that's relevant, where the hormones are particularly relevant.

Right.

But that is, that's kind of another treatment avenue and that involves taking pills every day for about 5 years that effects hormones. Um, so it's being investigated whether that helps reduce the risk of invasive breast cancer. Um… and those, those kind of treatments, um, often women find them quite difficult in terms of side effects.

Mmm.

And they tend to have side effects along the lines of kind of menopausal type symptoms that some women find difficult to, to live with.

[LOTS OF MMM's]

But that's, that's kind of the main other I guess treatment approach that's in the mix.

What about…

It's not used much in DCIS at the moment.

What about stem cell, stem cell research like towards it sort of thing? Or is that just for certain types of cancer sort of thing?

Um… I'm not aware of stem cell research in, in DCIS as such.

No.

[A FEW MMM's]

Um, but there could be. I mean there's bound to be things going on that I'm not aware of.

Mmm.

Ok, can I just ask, um, I think so it was Karen when you were saying before that because of you have a young child you would be wanting to do anything to prolong your life?

Yep.

So would that lead you to want to have treatment if you had DCIS?

I think it, I think it probably would. Yes. Yeah, I think it probably would. Just… yeah, um, because it's not, it's not my decision alone. You know, it has impact on your family. So, yeah, I think I probably would just to remove that element of doubt.

Mmm.

Yeah.

But I think if, if I didn't have that I probably wouldn't. I probably would wait and see, active monitoring.

Mmm.

Yeah.

Ok. So I think we've had a couple of people say they would rather have treatment? And quite a few say that they would want to go on a trial.

No (laughs).

So I, I, yeah, I think mine would be, I would actually, and I don't think, until you got diagnosed it's really hard to say definitively what, what you would do. But in what I've heard today at the moment I think I would be more than comfortable doing active monitoring.

Me too.

But I would probably be a little bit annoyed if I got in a trial, chose that path and then got pushed into having surgery.

Yeah. I agree.

That's the bit I, that's, you know, that's the difficulty.

Yeah.

Rather than just…

[TALKING TOGETHER]

… but, but having just to opt to go down that pathway, um…

Well because effectively that decision is taken away from you.

Yeah.

It's a chance, you know, it's a probability which group you're going to end up in.

'Cause there's no option now. There's no option now to have an active monitoring.

Yeah.

So…

The thing is just like what Karen said and even, um, Anne said, in that my children are grown up and I've got grandchildren and I've only gotten better because I've got grandchildren, 'cause it's given me a reason. But for me now, if I were to be diagnosed with something but they're not sure yet, I myself would actually sit and wait and actively monitor. And, um, I would take a bigger… to me it's like if you don't have any, having been finally cured of something that was killing me, I've actually been down that route and decided that well now I'm older, I've got, the children are grown up and the grandchildren, um, if I, I would have to think about it. I wouldn't be rushing off and having an operation because I don't see that, 'cause I'm 58, I don't really see the need to be worrying about it, um, as much because I'm, er, I don't have to worry about it. I live alone, so no one's going to suffer in my house, there's only me (laughs). So yeah, (?).

I'm quite indecisive so maybe somebody making the decision…

[LAUGHTER and TALKING TOGETHER]

… so I'll probably go yeah, put me in the trial and then lets see…

[LAUGHTER and TALKING TOGETHER]

… proactive about having, keeping, having a look…

Oh yeah, proactive.

… so you'll probably…

You'll just go for the ride, whatever…

[LAUGHTER]

… wherever it takes you.

Well because the, the facts sort of say that it's, it doesn't, either way, because it's got the same percentage…

That's right.

… so you sort of think…

So you might as well go…

… oh well, you know, like, if you're given the two options and well it's the same ratio of people who are, who are sort of…

Yeah, there appears to be, there appears to be. It may not be proven science, but there appears to be a bit of science around being in the low-risk sort of grading and the information they've got to date, um, that would give you some confidence going down a certain pathway. It would give me some confidence going down it, choosing the pathway.

I think if there was a big difference between, um, between the success rate of having active, having active treatment and, and just having the wait and see and active monitoring, I think if there was a big discrepancy between those two things, the two…

That's right.

… the facts we know so far then obviously that would sway you, that would sway me more one way or the other. But at the moment it's sitting quite the same, so… um, I think I'd be more than happy to go into a trial.

Either way?

Either way. Um, no…

[LAUGHTER]

… no, no that's right.

You'd be happy not to go into the trial.

[TALKING TOGETHER]

Yeah, because… but that's what, that option you don't have.

[TALKING TOGETHER]

… you want the pathway of a medical professional monitoring but…

Well why can't we have the option? Why can't that be something that we, um, that we, we opt for (laughs)?

Well you can.

Let's have just active monitoring.

Nobody can tell you what to do with your body, you know.

You can say no.

Oh, you can say no?

But they won't be recommending that.

Mmm.

But you won't, if you say no then you won't be going in the trial.

Yes.

If you say all I want to do is I want to be actively monitored then you can't be participating in the trial because there's the two groups and…

Yeah, well my sister had a growth on her thyroid. And, er, one doctor said, oh, we'll get that out straight, bang bang bang. The other, she got a second opinion, and the other specialist said, no, no, let's just actively monitor. And, um, after 6 months, after a year, the growth, the growth sort of got much smaller and it's still getting smaller. It's still there but it's small. It's getting smaller. So, um, yeah, she opted for the active monitoring more than the surgery.

Mmm. I know people who've gone into trials but it's always been like, er, traditional, um… the traditional medication, etc., um, with a new one coming up. it's not been ever more not having something. So most people I know that have ever been on trials get some sort of treatment. But not the active monitoring kind of thing. But I think if it's, they see some evidence of the low-grade may not develop for some, like may not develop into anything worse for some time, I think it's a good option to… it would be a good option to monitor it. But, as you say, with a trial you're going to get all that information that you really need from two, um, like from one group of people undergoing the two different things.

Ok, um, well thank you that's all been a really interesting discussion. Um, so the last thing I'm going to ask you to do is that one little, um, document two. Um, before you do that is there anything else you want to say that's, you know, we haven't covered that you wanted to just get in?

(UNCLEAR)… is for the lady who died of, um, breast cancer.

Mmm.

Do you know what is her, I know she decide not to go on the knife, er, so she died afterwards but what her condition, do you know? Does anyone know?

Well I mean she had breast cancer. I'm pretty sure she didn't have DCIS.

No.

I'm not aware of what treatment she did or didn't have.

Oh, ok.

But she was, she was one of these relatively unusual young women who had…

Very young, yeah.

… who had aggressive breast cancer.

Oh. Ok. Ok.

So that's, that's a…

Thank you.

… sad case. Um, but it's not, it's not the same as…

Ok, ok. It's not… ok, that's already cancer.

Yeah.

Yeah, they already knew what she had.

She was very young. She was in her 30's, wasn't she?

[MUMBLING]

Ok, um, so I'll just say before we start thank you so much for coming along.

Thank you.

Um, because after you've done the questionnaire, you're free to go. Um, and on your way out, we will give you your gift card as a little thank you for coming. So if you just go and see, um, I think Alanna at the back. We just need you to sign our sheet to say that you've received that. So thank you so much for coming today.

[LOTS OF THANK YOU's]

Very informative. I'm going to get a mammogram (laughs).

[TRANSCRIPT ENDS 01:46:40]

END

Interview: DCIS FG3 2018.05.22

Interviewer: **BOLD TEXT**
Interviewee: PLAIN TEXT
Pauses: …
Unclear words: (?)

[TRANSCRIPT BEGINS 00:14:00]

Um, now as we've, many of you have said, DCIS is something quite familiar, er, unfamiliar at this stage, um, so I'm going to start by talking about something that probably is at least a little bit more familiar, and is relevant to DCIS, and that's breast cancer screening.

So what is breast cancer screening? In Australia we have a national program that provides screening, free of charge to women, to look for early signs of breast cancer.

Now it's important to understand that when we talk about screening it's something that's designed for women of a certain age in the general population who are well. So it's not for women who have noticed a breast symptom (like a lump or pain or discharge) that they've noticed that needs investigation – that's a separate process.

The aim of screening is to find women who have cancer early, so that they can be offered treatment early, in the hope that earlier treatment will lead to a better outcome. And the main goal of screening is to reduce the number of women who die from breast cancer.

So the best available method for breast cancer screening is a procedure called a mammogram, which uses x-rays to make images of the breasts. And the whole idea is that these x-ray images can show up cancers or abnormalities that may be so small they can't be felt as a lump.

So if the mammogram images look abnormal or suspicious in some way, the woman gets called back to have further tests which may include a biopsy. A biopsy means taking using needle to take a small sample of cells from the breast to look at under a microscope, for the purpose of confirming the diagnosis. So from looking at what that small sample of cells looks like under the microscope, a pathologist will then either say actually everything's fine and it was a false alarm, or they'll diagnose breast cancer or DCIS.

So DCIS, ductal carcinoma in situ, is a condition that can affect the cells inside the breast.

Typically, DCIS doesn't cause any symptoms like a lump that you can feel, so it's found through screening. And before we started doing screening in the population, it was hardly ever diagnosed. So in the past, women may have had DCIS but because it didn't cause any symptoms and because there was no screening, they wouldn't have known about it. Whereas nowadays we have quite widespread screening and DCIS is getting diagnosed much more often than it ever was in the past.

So part of the challenge with this condition is that it's almost like DCIS is kind of a new disease that we've only really discovered through bringing in big population based screening programs, in Australia and other countries, and that's happened from about the 1990s. And so everything that women are told when they're diagnosed with DCIS, and everything I'm telling you today, is the best information we can give based on our current scientific understanding of DCIS. But it's important to understand that our scientific knowledge about DCIS currently is somewhat limited. So there's still some things we don't really fully understand yet.

I'm going to show you a diagram with some numbers now – don't worry too much about the numbers, it's just to give you a bit of an idea of how many women are affected by DCIS.

So if you imagine that each one of these dots represents one woman. So if we take 1000 women in the Australian population who have breast screening over a period of 25 years, in total about 81 of them will receive a diagnosis of one kind or another, as a direct result of a screening mammogram and the further investigations that follow.

And if we look in more detail at these 81 women, we find that 16 of them have DCIS and the other 65 have what's called invasive breast cancer – and I'm going to explain the difference in a minute. So as you can see, the women with DCIS are a smaller group – so they're about 1/5 of the total women who are diagnosed through screening.

So let's talk about what is DCIS is in a bit more detail. We'll break it down

word by word. Ductal means it relates to a duct or tube that carries milk to the nipple. Carcinoma means that the cells show abnormal features similar to cancer.

And 'in situ' means contained in the original place. So the 'in situ' part is a really important in defining DCIS. So this illustration shows how DCIS is different from 'invasive' breast cancer, which is really just a more precise term for what we commonly understand breast cancer to be. So based on what they can see looking at the cells from the biopsy, the pathologist will diagnose that it's either DCIS, meaning that the abnormal cells appear to be fully contained inside the duct, or invasive breast cancer, meaning that some of those cells have moved outside the duct into the remaining, into the rest of the breast tissue.

So DCIS and invasive breast cancer are different but they're related. In DCIS, the abnormal cells are contained within the milk ducts and have not spread outside the ducts into the other breast tissue. And as long as those cells remain contained like that, DCIS is not a life-threatening condition at all. But DCIS might develop into invasive breast cancer, and if that happens the time frame for it to happen is really variable, so it could take many years or even decades or it could happen much more quickly.

In invasive breast cancer, some of those abnormal cells which were previously just inside the ducts have actually taken the next step and moved out into the breast tissue around the ducts, and once that's happened, the cancer could then spread to other parts of the body, which is when you really have a problem.

But it's good to remember that although invasive breast cancer has the potential to be a life-threatening disease, these days the vast majority of women diagnosed with breast cancer will be treated successfully and will survive their cancer.

This slide just shows some of the different phrases that are used to describe DCIS. And none of these are wrong but it can be a bit confusing for women because DCIS is described both as a type of cancer and as something that's not quite cancer.

So if you haven't heard the term DCIS before, you might have come across some of these other terms like Stage Zero breast cancer or non-invasive breast cancer or pre-invasive breast cancer.

So what happens after DCIS is diagnosed? Well, as I've said, DCIS itself doesn't affect a woman's health, but it has the potential to turn into invasive breast cancer. Now we have reason to believe that this progression to invasive cancer might not happen for everyone, and I'll explain more about that later. But the bottom line is that right now we can't reliably predict which of the women with DCIS will get invasive breast cancer or how long it may take before that happens. So that's why virtually every woman diagnosed with DCIS is given treatment, to try and prevent that progression from ever happening. And I'll talk shortly in more detail about what that treatment involves.

But firstly I just wanted to, um, ask all of you, so given that explanation have you ever heard of DCIS before?

[LOTS OF NO's]

No? Do you feel you understand what I've said so far?

[LOTS OF YES's]

Anything unclear or confusing about it?

[LOTS OF NO's]

Ok. So have you ever heard the idea that some people are diagnosed with a disease through screening, and they have treatment, even though that condition may never have become life-threatening?

I've heard of it.

Yeah?

Yeah, preventative.

Ok. So what, um, what have you, in what context have you heard about that? Like in terms of, in terms of breast cancer screening?

I do a lot of reading and I've read sort of articles and things. I mean my own personal thing is I don't believe in it. Um, I think there's too much…

Too much what?

… intervention without a confirmed diagnosis.

Ok.

That's my personal opinion.

Mmm hmm. Ok, yep.

I have a friend who has the brca gene in the family and her daughter's just had a double mastectomy as a preventative measure.

Ok.

There's no cancer but she has the brca gene as well.

Mmm hmm.

So…

Is that what Angelina Jolie had.

[LOTS OF YEAH's]

Yeah, same thing.

She's the only one that I've really heard of that's had preventive…

Mmm, I've never heard of…

… treatment, but I did think it was for DCIS, I don't remember hearing those, that term with her.

Ok, yeah, so the people who have the brca gene is a different, um, category.

Right, ok.

A small category of the population who have that gene that makes them really at very high risk of getting breast cancer in the future. But what

about, um, the kind of ordinary people go for screening and then, um, are diagnosed, um… anyway, that's, that's what we're talking about here, DCIS.

Is there new, um, medications or something on the market now that people who are, um, have this DCIS, er, can take to kill off the cancerous cells?

So there are some, um, medications that are currently being researched for use in DCIS.

Yes.

Yes, as a way of, as, as another, um, type of treatment.

Type of treatment before it gets to the stage it's gone into the breast tissue.

Yeah.

Yeah.

Yeah.

Is that still at laboratory stage?

Well, er, no, it's not at laboratory stage. It's, it's being used, er, in some cases, in some places.

Mmm.

Um, in Australia it's not very commonly used in DCIS, but, um…

Why is that?

Well, basically they're still doing research to see whether, um, it helps, um, whether it really helps those women and, um… kind of weighing that up against the side effects that, that those medications can cause. So that's a… yeah.

So if DCIS, um, gets into the… do they just wait and see if it gets into the breast tissue and then you have to have a mastectomy? Or…

So, as I said, no, generally they don't wait. They'll do surgery to take out the DCIS.

Oh, they take it out?

So I'm going to, um, yeah, I'll tell you a bit more about the treatments that women typically have. So, so basically the goal of treating DCIS is to prevent the abnormal cells from turning into invasive cancer. And overall it seems that our current treatments are doing a pretty good job of that.

So surgery is really the primary form of treatment for DCIS. And that means doing an operation, in hospital, under general anaesthetic.

Does that mean removing the breast or removing the cell, or… the duct I mean?

I'll explain more in a moment. There's different types of surgery that women may have, um, and that surgery may also be combined with another form of treatment. Um, just another thing with doing surgery it means the pathologist also gets another chance to look at these cells under the microscope, um, but this time they've got more to work with so, um, they're looking at what's hopefully the whole abnormal area of the breast, rather than just the small sample they got from the biopsy. And in most cases this will confirm that it really is just DCIS as the biopsy suggested. But in some cases they find after surgery that the patient actually has invasive breast cancer that the biopsy missed. So that patient's diagnosis will be changed or upgraded at that point.

So as I said, there's different types of surgery a patient can have, and exactly what each patient has in terms of treatment will depend on features of her DCIS (like the size and grade – and I'll talk more about grade later on) as well as characteristics of the woman herself (like her age, her overall health, and her preferences).

So I'm going to tell you in a bit more detail about the 2 most common options for treating DCIS. And both of these approaches are considered very effective and survival rates for DCIS are excellent.

So treatment for DCIS most commonly involves breast conserving surgery and then radiotherapy. So this means having an operation to remove the part of the breast affected by DCIS and a small area of healthy breast tissue surrounding it. So this may also be called a lumpectomy or wide local excision.

And after breast conserving surgery a pathologist will look at the breast tissue that was removed. And sometimes, if they find that there's a bit more disease than they originally thought, then the woman may need more surgery.

Now everyone responds differently to surgery. Some side effects happen to most people, some happen only occasionally. And some will happen straight after surgery, whereas others can take longer to develop. But some of the common side effects include things like pain, numbness, and bruising around the wound.

And then after this surgery, the patient has radiotherapy, which is normally every day for a few weeks. Radiotherapy uses x-rays to destroy any abnormal cells that may be left in the breast.

Now receiving radiotherapy is painless. But women may experience some side effects. So during the radiotherapy treatment, the skin of the breast can become red and dry, a bit like sunburn, or the skin can become darker; and women often feel more tired than usual. And these side effects usually settle down after treatment finishes and things get back to normal.

Now during breast radiotherapy, it's possible that a small dose of radiation may reach the heart or lungs, depending exactly where the DCIS is. And some studies have suggested that women who had breast radiotherapy in the past may have a slightly increased chance of having a heart problem, like a heart attack, later in life. So doctors these days are using more modern techniques when giving radiotherapy to try and minimise this problem as much as they can.

So instead of having breast conserving surgery plus radiotherapy, sometimes a mastectomy may be recommended, for example if the area of DCIS is quite large compared to the total size of the breast or for some other reason. So this is a bigger operation which means there's a higher chance of complications and a longer recovery time.

So all the side effects I mentioned for breast conserving surgery are also relevant for mastectomy but because this surgery is more extensive, there may be additional side effects such as stiffness in the arm or shoulder.

Now obviously mastectomy affects the body shape and it can affect women's body image or how they feel about their bodies. And many women have the possibility to consider breast reconstruction surgery to try and help with that.

And just in case you're wondering at this point I want to mention that chemotherapy is not a treatment that's currently, that's typically used for DCIS. Um, so the main treatments are, as I've outlined: different types of surgery and radiotherapy. And as we mentioned before, in some cases, um, the hormonal medications might come into it.

So what are your thoughts and feelings about that information about treatment for DCIS?

Mmm. Seems pretty straightforward.

Mmm. Well I think it depends on the age of the woman for a start. Um, I think as you're older, if they're going to pick it up in the screening wouldn't you be screened, er, more regularly to check, make sure that you don't, it doesn't spread?

So you're saying as you get older you might…

Might not go for the, um, surgery option…

Mmm hmm.

… because, as you say, it can take a lot longer to develop. So if they can pick it up in the screening, wouldn't it be best to just keep going for the screening and the ultrasounds?

Ok, what do other people think?

I think that, that's true, yes. I mean if, if it's, it takes, it could take years to develop and if you have regular screening rather than rushing into surgery…

Yes.

… I think that would be a good option.

Is there a genetic link… um, in women that get it? Like, you know, how there's the genetic link with women that have breast cancer, is there a genetic link to getting

DCIS?

So for, for both DCIS and breast cancer there is sometimes a genetic, um, link. But for most women who get it there's no family history or it's kind of a bit more random.

Ok.

Yeah.

Personally, and this is my personal opinion, if I found I had DCIS I would want to be rid of it immediately because I wouldn't want to live with that worry. Is it spreading, you know?

Yeah. I agree with that. That's what I did with my bowel cancer.

[A FEW YEAH's]

I didn't want to sit there and wait for months to see how far it was going to spread. Yeah, just get it off. But then I'm widowed, so I think that makes a big difference too.

How does that make a difference?

Oh, don't have to worry about your sex life do you, if you're a widow?

[LAUGHTER]

At my age, I'm nearly 73, so it doesn't really effect me.

Well my, my oldest sister was diagnosed at 72 with breast cancer and, um, she went into surgery and she said, take them both off. She said, I'm widowed so…

Yeah.

… she said, I hate wearing bras anyway.

[LAUGHTER]

So… she's still going strong at 78.

That's very much personal.

Yeah.

What about younger women when it comes to breast-feeding? Is there a problem with the activity of, er, the manufacture of milk? Does that make any difference to what's going on in the milk ducts?

Um, yeah, so, so breastfeeding is, um, something that can reduce the risk of getting, er, breast cancer or DCIS. Um, and, but in terms of if women have DCIS and would want to breastfeed later, um, it could, it could well be a problem with treatments. But, um, remember also DCIS is generally found through screening, um, and that's generally done with women over 50.

Older.

Or at least over 40.

Mmm.

So the, the number of women who are young enough to, to breastfeed and, you know, who would get DCIS is very small.

But if you're not screening the younger women how can you be sure of that?

Well, um, we know that the, the risk of breast cancer goes up as women get older, so we do have of course occasional cases in younger women, but, um, in general it's a condition that, that arises more as women get older.

So what's the age where it's classified as older?

Well I, I mean it's just... well, it's sort of just...

[TALKING TOGETHER AND LAUGHTER]

Would it though, I'm just saying, would it be 50 when the free breast screening starts?

Around 50. So around the kind of menopausal time.

Right.

Is when, um, the risk gets higher, yeah.

Why does breastfeeding reduce the chance of you getting it?

Good question.

Um, that's a good question. I think t's just the, sort of the activity of…

The flow.

… the moving things through. I mean it's also, um, just having children… well it's also because of the… um… hormones, the hormone activity involved. So just, um… having children, because that provides an interruption to the constant up and down of the hormones…

Oh yeah.

… and the menstrual cycle…

Oh.

… that actually is, um… can reduce the risk of breast cancer.

Like the body's ticking along as it should be… and it helps in someway?

Yeah, so, so even though, um, you know, its normal to have, um, monthly menstrual cycle…

Mmm.

… um, the, the hormones are going up and down a lot and that, for some cases of breast cancer, can sort of contribute to development of breast cancer. So, um, that's also why women who, um, start their periods earlier as young girls and enter menopause later, so they have a longer time when they're having periods, they have a slightly higher risk.

Mmm.

And having children especially earlier in life, that interrupts that…

(Laughs) There goes me.

… and slightly lowers the risk.

I've got some fors and some againsts.

[LAUGHTER and TALKING TOGETHER]

Um, ok, so… ok, so we had a couple of people saying that you think you'd rather, you know, have treatment and get it out straightaway.

Mmm.

And a couple of people saying you might prefer to, um…

Frequent screening.

… screen and see how it goes. Um, what about people who haven't said anything on that yet?

I think I'd prefer to listen to the advice of the doctor that I trust…

Mmm hmm.

… and then make a decision from that.

[A FEW MMM's]

I'd probably lean towards having them both off at my age. Um, if I was younger I wouldn't, but at 71 it's… yeah, we're talking about sex life as being not too existent really.

[LAUGHTER AND TALKING TOGETHER]

I beg your pardon.

[LAUGHTER]

Ok.

I think I'd probably have it, the operation.

Mmm hmm.

I'm afraid I'm an ostrich. I'd bury my head and think well if it's, what will be will be, it's what God's done for me and I would continue my life, and put it to the back of my mind.

Ok.

That's my philosophy.

Having the whole breast off like that's an extreme treatment.

Yeah, that's drastic.

[LOTS OF YEAH's]

Yes, so isn't there a…

Yeah, there's a lumpectomy if…

Mmm. Lumpectomy.

Yeah, so you more commonly would have the, a smaller part of the breast removed and then typically the radiotherapy after that too. You know, they sort of compliment each other.

So how far into the breast are the ducts actually?

Well they're… I guess from that diagram…

Yes.

… they're sort of half…

They look like they're spread out.

Yeah, I mean…

[TALKING TOGETHER]

… presumably mainly ducts and tissue.

Yeah, basically they, you know, they cover quite a… large area of the breast, so…

Mmm.

… that also will depend, um, you know, will influence what kind of surgery is recommended. Sort of how easy it is to get at… that particular area…

Yeah.

… that's involved.

Mmm.

Er, yes, well (laughs) as we get older we have other things going wrong.

(Laughs)

Um, I was diagnosed with (laughs) Type I diabetes at 60, just over 60, that's the one that used to be juvenile, and (laughs) I got that out of, out of nowhere, no family history, nothing. Um, er, just over 60. Um… and, um, I'm on all sorts of medications for complications from diabetes. And, um, yeah, so, um… to take on something else like that, um, you know, I think I've got enough on my plate (laughs). I'm swallowing 14 tablets a day and having 4 injections a day and, um, yeah, so I think I'd probably listen to a trusted doctor, and, um… go on his advice, er, and I'm a bit nervous about something as drastic as a mastectomy. So lumpectomy sounds a bit more like the way I'd go with lots of, with regular, um, screening.

Mmm hmm.

But yeah, I mean, and I say, you know, we're getting arthritis and all sorts of things and, um… (laughs), um, it's, it's hard to know how you would deal with extra on top of if you've got other things already.

Mmm.

It'd be overwhelming.

[LOTS OF MMM's]

Yes. Yeah.

Oh, I should note. I'd hate to have a one off because with these little puppies a good size. I think I'd swim in circles.

[LAUGHTER]

Yeah, I think one would be very awkward.

Yes. I think I'd have to do what my sister did and have them both off, especially for breaststroke.

Yes (laughs).

My, my husband says you'll never sink. You've got built in water wings.

[LAUGHTER]

I had a friend at 35 that actually got breast cancer and had a ma… one breast off. And later on had a reconstruction. Um…

I think you can only get that if you're in a private health… can't you? Because if you're in the public, you know…

She actually showed us when she had it removed and it was really, it wasn't, it didn't look…

Pretty (laughs)

... no, it didn't look that bad.

Oh. Ok.

I mean, she was really…

Well it was a pretty good job. You know?

… yeah, and she was a very thin person and her breasts were reasonably small. And she actually, there's a group of us that she showed, and I wasn't disgusted by it at all. Um… later on she got a new partner and felt that she needed to…

Yeah, well there's a lady in our swimming group who, er, has had a double mastectomy and she's had the reconstruction and… they look great (laughs).

Yeah.

I'm very jealous.

[LAUGHTER]

I'd take off my bra and swim a lap.

[LAUGHTER]

Oranges in socks.

Yeah, something like that.

[LAUGHTER]

Ok. Um…

[LAUGHTER]

Good, well I'll…

She doesn't quite know how to respond.

[LAUGHTER]

No, that's… that's all things, I'm just interested in what goes through your minds.

[LAUGHTER]

All very… interesting. Um… ok, so, so having said that, you know, we're thinking about, a lot about downsides of having treatment and, you know, possibility that you might prefer not to, um… so it is still the case that, er, the medical community at the moment views that DCIS should be treated, um, and in your age group as well. So the screening program is now encouraging women up to the age of 74 to have screening and, you know, so they may be diagnosed with DCIS and the current standard is to offer these kind of treatments. But there is a question, um, that's occupying DCIS experts quite a lot at the moment, which is could some women with DCIS quite safely avoid treatment? So, as I said, the current treatment for DCIS is based on our best scientific knowledge and of course wanting to do what's in the best interest of patients.

And as I said before, survival rates for DCIS are excellent. But of course many patients do experience side effects from treatment and this includes both the physical effects on women's bodies, and emotional or psychological effects as well, and they can affect women in the short term

and the long term.

And so… just want us to talk a bit more about DCIS particularly as women get older. Because, um, as you've mentioned, as, you know, the older women get, the more likely they are to be living with various other health problems. And as I said also, DCIS is a condition that may stay just the same or progress very slowly over many years (or even decades). So for women who are already older when their DCIS is diagnosed, it may not actually affect their health in their remaining lifetime because of those other health problems that are more important for them.

And there's another issue here is that older women may be more affected by the side effects from treatment, such as complications from surgery or heart problems linked to radiotherapy for example.

So do you feel you understand that information?

[LOTS OF MMM's and YES's]

Any questions about that?

[A COUPLE OF NO's]

So, um, any more sort of thoughts and feelings about DCIS in older age groups in particular?

Only for the fact that why are you sort of more or less cutting off the mammogram thing at like 74?

Yeah.

I know a lot of people that have had been diagnosed with breast cancer and most of them I will admit kept their screening up, but they were older than that, in their early 80's.

Yes.

As if, well you get to 74, we care about you up until then (laughs)…

[LAUGHTER]

… after 74, well there you go. And…

Yeah, but you can always have a screen.

Oh yeah, but they don't send you reminders any more and it's up to you and I sort of figure the older you get…

The more forgetful you are (laughs).

… yeah. Well, yeah, I mean I'm…

During these studies what sort of…

[TALKING TOGETHER]

Sorry…

During the studies what length of time have you established from detecting DCIS to it becoming active from dormant?

Well it can be highly variable.

Yeah.

It can be very short, it can be… it can be long but…

But on a ratio of average?

Well, um… I mean the problem is we don't really know because what we tend to do when we find it is take it out.

Right.

Then we can no longer answer that question. So I'm going to show you in a minute a little bit of like one example of a study that, that sort of addresses that.

Mmm. Would that be similar to like when I was breastfeeding… blocked ducts when you're breastfeeding?

Um, no, it's not.

No.

It's not the same thing. Because it's, it's… the cells having these cancer like abnormalities.

Yeah.

It's…

Yep, yep.

Is this sort of a mirror to men's prostate cancer? You know how like they say that most men will die with having a slow growing and… but then some of it turns to cancer. Like it's…

And they die from something else before it…

That's right. They'll die from something else rather than the prostate cancer.

Yeah, but it some of it goes to, you know, the next step, doesn't it? And then they have it removed.

Mmm.

My cousin's husband was diagnosed at 47…

Oh.

… with prostate cancer and it was already in the bones.

[LOTS OF OH's]

So it had spread.

[LOTS OF YEAH's]

So it is possible.

Yeah, and in answer to your question there is, there is a certain similarity there that it can be something that, um… yeah, as you get older it's more, it's quite likely to be there but not effecting you, and may…

You may die of something else first.

Yeah, yeah, that's the thing.

The way I look at treatment too, um… the way science is progressing, if I was diagnosed with DCIS I would tend to watch it, you know, with the doctors advice because you never know what science will come up with in the future.

Yeah.

And it might be a much better way to treat it.

Yeah.

And less invasive.

[A FEW MMM's]

So that's what I would do.

That's why I asked about is it still at the lab stage? The research.

[A FEW MMM's]

Well, um, I mean basically we, there is lab research also going on all the time to…

Mmm.

… but, um, yeah, a… so currently we have the sort of current treatment paradigm of, of pretty much surgery for everyone. Um, and, and so.. yeah, but I'm going to tell you a bit more about the, the current research that, you know, that's quite interesting about this.

So ok, how's everyone doing?

Yeah.

Really good.

Do you need a little 5 minute break to stretch your legs or…

No.

… or are you happy to…

No, we're not old.

[LAUGHTER]

Just checking. Ok. So…

[TALKING ABOUT PASSING WATER]

I'd like to just ask, um, if you've been diagnosed with dense breasts with cysts and, er, calcifications, does that effect this?

So, um, calcifications is, can be a way that DCIS appears on a mammogram.

Mmm, yeah.

It's basically like, um, little white specks that are…

Yes, well I've got that.

Mmm hmm.

I've just been for a screen in November last year and they never came up with anything like this.

Mmm.

So…

Yeah, so… and in terms of cysts, um, you know, a lot of women have cysts in their breast that are benign, they're just kind of little collections of fluid.

Yeah.

Um, but so things, you know, the thing with screening is that if you're having regular screening they'll keep, um, all the pictures so that they can see if there are any changes and…

So they do look out for this.

Yeah, they'll be looking for that.

Yes. Right.

So they'll know what's normal for you, including those cysts and calcifications…

Right.

… and they'll be looking to see whether there's any indication of something new coming up in future screens.

Mmm, ok.

Ok, so, um… so breast cancer experts now are doing research to try and identify women who have DCIS but have a low chance of getting invasive breast cancer so that in the future, women like this may be able to avoid surgery and other treatments and therefore avoid all those problems that can come along with treatment, while still having excellent survival rates.

And of course this kind of development may be of particular interest to older women, um, because of all the things that we've been talking about. And as I said before, it's very difficult to reliably tell who these 'lower risk' women are, but we do have a few clues. And one of them is something I mentioned earlier called grade.

So after the biopsy is taken, the pathologist looks at the sample under the microscope and gives each case of DCIS a grade based on how the cells look, and that gives an idea of how fast the abnormal cells may grow.

So low grade indicates that the cells appear to have a low level of activity, which suggests they may stay like that for quite a long time. On the other hand, high grade DCIS means the cells appear more active, so they're likely to grow at a faster rate. So it's considered important to treat high grade DCIS because otherwise it's pretty likely to develop into invasive breast cancer at some point. And then there's also an intermediate grade that's somewhere in the middle.

Now you might be wondering, if virtually all women with DCIS are having treatment, as I said, um, so having surgery to take the DCIS out, why am I saying that we think low grade DCIS may stay as it is for many years and not cause problems, even without treatment.

Well, we think that based on a small number of research studies similar to this one.

So just as an example I want to tell you a bit about this study that was done in America using a database of routinely collected information about cases of DCIS. So these blue bars represent all the patients in that database who had low grade DCIS. So as you can see, the vast majority of these patients, almost 9000, were treated with surgery, because that's the typical thing to do for DCIS, that's recommended in all the guidelines for doctors.

But a small number of patients, just a couple of hundred, didn't have surgery for some reason, and we don't really know why not.

So this database kept track of key events that happened to all these patients over many years, for example it recorded if any patients died from breast cancer. And what the researchers did in this study was compare the larger group of women who had surgery and the smaller group of women who didn't, after about 10 years. And they found that the women who did have treatment had a very high survival rate, so 99% of them had not died from breast cancer, so they were doing really well. And the research also found that among the women who did not have treatment, 99% of them also had not died of breast cancer.

So this led the researchers to suggest that there may be a subgroup of women with DCIS who are likely to have really good health outcomes over time, whether they do or don't have the typical treatment of immediate surgery for their DCIS.

So this idea of a subgroup of DCIS that we could call 'lower risk DCIS' is a big focus of new research that is designed to try and investigate whether women in that category could possibly avoid treatment.

And there's a range of factors that are relevant to trying to define this category of DCIS – grade is an important one but it's not the only thing. There's lots of other factors that are taken into account that I won't go into now.

And so coming back to this little diagram we looked at before I just want to give you a rough idea of how many women with DCIS might be classified into this lower risk category. So of our 16 women with DCIS from before... we think that about 5 women have what could be classified as lower risk

DCIS based on their biopsy.

For the other 11 they have, um, they're considered to have a higher chance of getting invasive breast cancer at some point so it's still considered more appropriate that those women do have treatment.

But these 'lower risk' women are the focus of current research looking at whether they might be able to avoid treatment. And I'm going to tell you a bit more about that research now. So it's just important to remember that we're talking about this quite small and select group with DCIS where researchers are considering a new, different approach to managing their condition.

So the type of studies I want to talk about now are called clinical trials, so let me explain what they are.

A clinical trial is a research study that asks an important, specific question about a certain health condition. Clinical trials often test new ways to manage a condition, by comparing a newer approach with whatever is the current standard treatment.

And trials are designed to find out whether patient outcomes (like health and quality of life) are similar for both options, or whether one option is better than the other.

Now if you think back to that study I talked about before, with the blue bar graph, it was done by looking at a database of, um, routinely collected basic information about patients from the past. And although studies like that can be valuable for giving us some interesting ideas, it's hard to draw firm conclusions from that kind of research because there's a lot of important information missing, for example details about those patients who unusually did not have surgery and why.

Whereas clinical trials are specially designed to overcome the limitations of other types of research. So that's why trials are considered to give us the highest quality of evidence.

So… this illustrates the basic design of a simple clinical trial. Sometimes they can be more complicated, for example having more than 2 groups, but

most typically, each patient in the trial is put into 1 of 2 groups. One group of patients gets the standard treatment, while the other group gets a new treatment or a new way of managing their condition. And which group each patient goes into is determined by chance, using a computer. This process is called randomisation and it's a very important part of clinical trials. When we compare 2 groups of patients having their condition treated or managed in different ways, we want to make sure the groups are very much the same in all ways other than the way their condition is treated or managed. If patients and their doctors choose which treatment to have, then the patients in the different groups will end up being different from each other in important ways that are relevant to their health, which can create a bias that makes the results less reliable. So patients who volunteer to take part in a clinical trial need to be comfortable with ending up in either of the groups because they don't get to choose which group they're in. Randomisation makes sure that the study groups are as similar as possible. And that way, we can get the most reliable information from the results of the trial to learn about the effects of those different ways of managing the condition.

And deciding by chance how a patient will be treated may seem strange, but it's considered an ethical thing to do in situations where we genuinely don't know which option is better than the other.

So there are currently 3 clinical trials going on around the world for women with low risk DCIS: one in the UK, one in Europe based in the Netherlands, and one in the US. They all just started in the last couple of years, and together they will involve over 3000 women. Now there are slight differences between the trials, but they all have the same general design.

And there aim is to learn whether women with low risk DCIS could safely avoid having treatments that may not be necessary for them.

So the women who are invited to take part in these trials have to meet strict criteria to be considered suitable. They have to have low or intermediate grade DCIS (not high grade), they can't have any lump you can feel, plus there's lot of other more technical rules that I won't go into. So

remember, this is what typically happens to these women with low risk DCIS: um, they have treatment pretty much straight away, um, and we talked about those treatments before of course, so surgery of some king with or without radiotherapy..

And of course in the following months and years, if it seems their initial treatment didn't work as well as hoped, or their DCIS comes back, or they get DCIS elsewhere or invasive breast cancer, they might end up having some further treatment if needed.

So that's the normal scenario for pretty much all the women in places like Australia where there is no clinical trial. But for women who do have a trial available where they live, if they're suitable to offer the trial, they may just say no thank you, I don't want to go in the trial, I'll just have the usual treatment. Or, if they decide to join the trial… then they get randomised to either usual treatment or the other group which is called active monitoring. So the usual treatment group has usual treatment, of course. Whereas the other group has a different approach where in the first instance they don't have surgery or radiotherapy. And then the patients in both groups are monitored closely, so they all have mammograms at least every year. And importantly, patients in the monitoring group may have treatment later if there's any sign that their DCIS is progressing, and we expect that to happen for some of those women. So this approach may be called active monitoring or active surveillance and it's actually already an accepted option for men with early prostate cancer because trials similar to this have shown that it can be safe. So these trials will test out the idea that this monitoring approach where you only give treatment if it's needed may result in the same very high survival rates as standard immediate treatment, but with fewer side effects overall because some women will avoid treatment altogether, and others will end up having treatment down the track but it's delayed so they may have a period of time where they're free of those treatment effects. So those are potential advantages of monitoring.

There's also potential disadvantages that the trials will examine. For example, patients who don't have immediate treatment may be more likely to end up getting invasive breast cancer – although with the monitoring of

course it's still likely to be picked up at an early stage. As I mentioned before, it's also possible that some women who are diagnosed with DCIS based on their biopsy actually have invasive cancer at the time that the biopsy didn't pick up. We also don't really know how women having monitoring will feel about not having treatment, so in the trials the patients will do questionnaires to help the researchers understand the psychological effects of this new approach, and that's a very important aspect of the studies.

Now because there's only quite a small and select group of women who are eligible for these trials, and because they then need to be followed up for quite a few years, it will still be some years before we have any results from these trials.

In the meantime, it's possible that a similar study could be started in Australia and New Zealand, so women here might one day have the possibility to go into a trial.

But otherwise until the results of these trials come out, women with DCIS will continue to have treatment as normal because we don't have strong research evidence to show that any other approach, like active monitoring, is safe.

So what are your thoughts and feelings about these clinical trials?

I think they're a very good idea… if I was in one, god forbid.

Yeah, I would hope I went into the monitoring side (laughs).

[LAUGHTER]

Yeah, my son's in one at the moment for myeloma and I think that's wonderful because there was no other choice like for him. He's not, he's terminal not…

Mmm.

… not diagnostic stage. But I think clinical trials are absolutely fabulous. We need more in Australia.

Ok.

So after they're randomly put into the two, one of the two groups, are they told which one, one they're in?

Yeah, so…

They are told?

Yeah.

They'd have to be if one's having surgery and one's not.

[LOTS OF YEAH's]

Yeah, that's right.

No, I didn't mean for the surgery against the…

The monitoring.

… yeah, back in that section, um, there was the, the current treatment and the trial treatment.

Yeah.

Do they know whether they're staying on what they've all, what they've been on currently? Or if they're in the one with the, the new trial treatment?

So you might be thinking of, um, for example the drug trials, where they often do something called blinding or masking where one group, um, might have a placebo or they might, basically they might not know what they're taking, 'cause they're just taking a pill and they don't know which group they're in. Um, but that's, in this type of trial, a trial of surgery, that's not really possible, so… in these, um, in these studies women would be told straightaway that they're either in the group that's going to have treatment or they're in the monitoring group.

Right.

I'm in a group like that, taking Vitamin D. And you don't know whether you're actually taking it or taking the placebo.

Mmm hmm.

[LOTS OF OH's]

If women have psychological problems about ending up in the…

I haven't got it.

… um, in the group that receives no treatment, would they, um, be taken off, out?

Well, um…

Like if they asked all these questionnaires to see their psychological state and they might worry that, um… because they're not receiving treatment that their chances of survival are reduced and that could stress them out.

Mmm, yeah.

And so, um, so if they see this happening do they take the people off that, um… er, that…

Out of that group.

… that avenue?

Yeah, so if the woman was, um, becoming distressed and really wanted to have treatment then of course she can have that.

I guess she's supposed to decide before you start, but, um, you could sort of get into it and develop a few more…

Yeah.

… stresses about it (laughs).

Yeah, absolutely.

And clinical trials you can terminate at any time on your own choice.

Oh, ok.

Yeah, so you… in that situation you might either kind of withdraw from the study completely if you, if you just didn't want anything to do with it any

more, or you might have your treatment but still stay in the study and do your, you know, questionnaires and so on, so that the researchers can still keep track of how you're going. So, um, as I said, some women in the, in the monitoring group will end up having treatment and that could be because something changes biologically and/or because something changes for them emotionally. And so the researchers will be keeping track on how many women, um, that applies to, um, and, and taking all that into account when they're comparing the initial strategy of initially having treatment versus initially not having treatment and seeing what happens.

And all those women, they're all low-grade?

They're, they're all low or intermediate grade.

Oh, low or intermediate grade.

Yeah. Ok, um… so yeah, I just wanted to explore a little bit more the, you know, if you can all try to imagine if you had been diagnosed with low-risk DCIS and if you're invited to join a trial like this… how do you think you would feel? How would you decide?

I think I would go with the trial, yeah.

I think I would, once again because of my age. Maybe not when I was younger, um, when you have dependent children on you and all the rest of it, um, I wouldn't be taking any chances at all. But at my age I think that would be fine.

Ok, so you're saying if you were younger you would be more likely to just have treatment?

Yes. I think so.

Ok.

After listening to this, yes, I'd go into the trial. But you need to go into it knowing and being accepting of the fact well, what if I… want surgery and I don't get that group or whatever, I don't want the surgery… yeah…

[LAUGHTER and TALKING TOGETHER]

Yeah, that's right.

So you really need to go into it accepting of what group you get into, to me I would say, to start off with.

Mmm hmm.

But then hopefully that questionnaire has sorted your mind out (laughs) to a degree because they say a lot of things are helped along by stress, so I could understand where if you started to stress about it you'd really need to take yourself out of the situation that you, you found quite stressful, whether it's the your name on the (trial?) or the surgery group. Yeah.

Ok.

And again I think your age and your circumstances have a lot to do with it.

[LOTS OF YEAH's]

I think your age has a lot to do with it, 'cause something's got to get you in the end.

Yeah, yeah.

[LAUGHTER]

I need to think about it seriously before I… yeah, I need to read as much about it as I could before I sort of put my toe in the water I think.

Yeah, I think if I was diagnosed with low-grade… I wouldn't go in the, go in for it because, um, into the group, because I'd rather be monitored independently.

[A FEW MMM's]

I agree.

Yeah, because you might be in the other group.

Because otherwise you'd get into the group that had the surgery and if you're low-grade you wouldn't want to have surgery.

That's it, I wouldn't want to have sur… yeah.

Because I'd rather be monitored.

[LOTS OF MMM's]

I'd prefer…

So, I don't think I would go into…

… yeah, that's a good, good point.

… I'd love to help but… (laughs).

Yeah. Personally I wouldn't go in.

If I was high-grade, different story.

Oh yeah, you'd be a shot out…

Yeah (laughs).

Yeah, ok, well I think that's pretty convincing actually, I might change my mind to yours (laughs).

[LAUGHTER]

How can they be sure whether it's high or low grade? I mean it doesn't come up in blue or red like you had on the… (laughs)… screen.

Maybe it does.

Oh, I'd go for a blue one then.

[LAUGHTER]

No, that's a good question. So they, they, they can't really know for sure. They, um, so the pathologists, um, do their best based on looking at that biopsy. And of course, um, in the context of these trials they would have more than one pathologist looking at it. And you know, they'd try and get a consensus as best they can. But, um, it is, it's an imperfect tool. Um, because the biopsy is only a small sample from the breast as well.

Yeah.

So there is, um, always a chance that it's, the diagnosis is wrong.

But why, why couldn't, um… its probably wouldn't work, but if you wanted, if you had the two groups of people that are actually choosing your low-grade and you're choosing not to have surgery… I, I can understand that you need to be… then the person's making that choice and they're more comfortable with what the choice they're making.

[LOTS OF MMM's]

Um, rather than… and then the people that want to have surgery, you've got that other group that want to and the other group doesn't want to.

Doesn't want to, yeah, yeah.

Yeah, it seems… because you're going to cut down on stress. I mean, if you choose to do something you're, you've got control of it. You're, you're the one that's making the choices.

That's right.

[A FEW MMM's]

So that, um, if you're actually making the choice, I've been diagnosed as low-grade and I prefer to go the, the non-invasive, in the non-invasive group…

Monitoring.

… monitoring group, um…

Yeah, but that doesn't make it random then, does it?

Yeah, that's right.

[TALKING TOGETHER]

… it's got to be random, doesn't it?

That'd, that'd be random though, because I don't know you and you don't know me.

Yeah.

And…

But they've the same psychological… um, outlook I would imagine.

Oh, yeah.

One group chooses one things and the other…

Yeah, that…

… chooses another.

… that could be… yeah. Yeah.

Yeah.

It's interesting.

So… yeah, I mean you, that's, that's… that's one way you could look at it. But I guess there's a few challenges with… there. Firstly, as you said, the… the people in those different groups will end up being different, um, and they can be different in ways that we can measure and know to look at, um, but they can also be different in ways that we don't even realise are important. And so we can't get a, a really unbiased comparison of the, of the management strategies.

Mmm.

Um, and the other thing is that at the moment, um… because we don't have a lot of good stud… you know, we don't have this, results of these studies yet, most clinicians at the moment will be quite reluctant to not treat women with DCIS.

[LOTS OF MMM's]

So… I'm not saying it never happens, but in, in women who are considered physically fit to have surgery you will find that most, um, clinicians whom are seeing these patients would be encouraging them to have treatment, 'cause that's what's in all their guidelines based on the evidence that we have to…

So they would feel like, they would feel like they were taking a chance if they didn't do that and the woman died, um, then they'd be responsible for her death by not treating her, wouldn't they?

Or feel responsible.

But they're surgeons aren't they?

Yeah.

They're surgeons aren't they?

And you've made a choice.

Surgeons, like that's what they do. That's what they do.

That's exactly right.

[LOTS OF YEAH's]

But getting back to the, the low-grade, now say I, I go for mammogram every, every year. Um, if they picked it up and said, right, we're going to investigate and do the needle biopsy and they said well it's low grade, and I choose not to do anything about it, um, if I go back for my mammogram the following year and it's still the same, wouldn't that be a good follow-up, rather than surgery? It's up to the individual I gather.

I think that's what we're trying to establish here. Now.

Yeah. I mean…

But you're saying it could progress. What I'm saying is if I go every 12 months, can they tell if it's progressed?

Yes.

Yeah.

Good.

So they can tell but, um… I guess as you say, the, the…

The risk.

… the surgeon will be thinking well what if it progresses sooner than that?

Yeah.

So they can progress quite considerably in 12 months?

Yeah, I mean it's quite unpredictable and so…

Does it depend on your age though? The older you are the slower it is?

[LAUGHTER]

That's just the rest of us.

[TALKING TOGETHER]

What are the chances that it's slow, slow growing?

Yeah, I mean once it's…

Because it's contained?

Yeah, there, there is a suggestion that it may be that it's slower as… for older women but again it's, we don't really know.

Just slows down with us generally.

[LAUGHTER]

Yeah, men's prostate does that as they get older as well, so it's the same.

Yeah.

[TALKING TOGETHER]

My husband was diagnosed with a lump and they did 20 little biopsies and, and it turned out to be benign.

[LOTS OF MMM's]

So…

Yeah.

… you don't know.

No, no.

But if there's some traumatic event in your life doesn't that… in some cases make cancer spread more quickly?

There's actually no evidence that stress…

[LOTS OF MMM's]

Really?

… causes cancer.

If that was the case I would have been dead years ago.

[LAUGHTER]

Most people would.

Yeah, so research has shown that, you know, stressful life events and so on, there's actually no link to cancer.

In some people.

Previously it was thought that stress did make it worse.

Yeah, so it can certainly affect, um… you know, how you, if you, if you do have caner then, um, how you kind of cope with treatment can, can depend a lot about, on, um, you know, how you're doing psychologically and the kind of social support you have and… you know, things like if you're stressed you might feel more pain, for example, things like that.

Right.

But in terms of actually causing cancer to start or to spread…

You'd probably, probably get run down too, that, that wouldn't help matters.

[A FEW MMM's]

Physically, health wise, you get run down.

Yeah, so there are certainly other, you know, physical health conditions that are, that are more related to stress and, um…

Yes.

… but cancer itself is not…

I think that's a fairly widely held belief that stress trigger…

[LOT SOF YES's]

… the growth of cancer.

Absolutely.

Yeah, I've heard, I've heard…

Yes.

But that was back in the 80's wasn't it (laughs).

[LAUGHTER]

When stress was for everything.

Well if I had a choice I'd go into the chop it off group. Even if I was low. 'Cause I wouldn't want something percolating, like it's a precursor to invasive breast cancer, and I wouldn't want it percolating around. I'd have it off.

It's an individual choice.

Choice, yeah.

[LOTS OF YEAH's]

But then, as I say, I don't have a husband.

Or a good one.

[LAUGHTER]

You can have, um, the reconstruction.

Yeah, but you could have that the first step one, the long-term thing…

[LOTS OF YEAH's]

… first before you actually loose the breast.

But this is the, the ducts goes right through.

Yeah.

I think in some cases…

I don't know what it looks like (laughs)…

… it would depend on the support you get, um, from your family, um, or husband if you've still got one, whether they're encouraging you to do what you want to do, not pushing their point and sort of saying, well no, I don't want you to have it because you may not look good, or… and then if you're there thinking well, I'd rather get rid of, don't care how I look, just want to live…

Mmm, so Liz was saying here reconstruction too.

Yeah, yeah.

I know two, two ladies who had the…

And I think it's a lot to do with your own mental strength as well.

… full mastectomy and reconstruction and they're, I think, very happy, yeah.

Everyone's different.

[LOTS OF YEAH's]

Depends on your circumstances so much.

Yeah.

[LOTS OF MMM's]

Ok, um, and so when you were saying that you would want to, um… read up

a lot and think about it a lot before you decided, what would you, what kind of information would you be looking for… to help you?

Um, like to go on, into the, the, um… trial?

If you had to decide whether to go into the trial or not?

Yeah, um… yeah, I'd sort of like to… well, they haven't got statistic on it, I love statistics.

[LAUGHTER]

About how many, um, women that have had low-grade, been diagnosed with low-grade and just let it go with the flow compared to, well, and the people who've actually had the lump… ectomy, is it lumpectomy?

Yeah.

Um… or and, and what their prognosis was? But there's no… if these trials are still going on obviously there's not…

The statistics.

… the statistics to go with, yeah.

Yeah, and that's the problem. There's only three trials worldwide.

Yeah. Which is…

How long have those trials been going on?

Well the American one was 10 years, wasn't it?

Well their… the first one started in 2014. And the other two started last year.

Oh, right, so…

[TALKING TOGETHER]

… statistics.

No, we don't.

10 years.

Yeah, exactly.

That's a long time.

It's early days.

[A FEW MMM's]

From my point of view, if, if you go into this randomised trial, um… if you're in the surgery group, too late to change your mind (laughs) once it's done.

[LAUGHTER]

Whereas the other group you, you still have the…

Yeah, the recourse.

… the choice. So…

That's why I said you've really got to go into it being accepting of which group you would end up in.

Yeah, that's right.

[LOTS OF MMM's]

And knowing well if it's the surgery one, yes, I will have the surgery.

Are you willing to have it? Do you really want to have it?

Yeah, have the surgery. Accepting of both, you know…

I mean, I…

… both outcomes.

Yeah…

Ok.

So maybe we wouldn't make a good (laughs)…

[LAUGHTER]

And as much as I'd like to help, um, other women, um…

Yeah, you have to make the decision for yourself.

… yeah, of course.

First and foremost.

Look after number one.

And I think now I sort of, I think I came in, or started out with a particular, um, I would do this. But in listening to all of this I'm… I sort of think perhaps… that mightn't be what I would do, because at the present I don't have it.

[LOTS OF YEAH's and MMM's]

Yeah, well that's right.

And that, I think changes. We're saying if we have it this is how we feel now. But then once you get something I think your attitude to it…

Yeah, your opinion can change.

[LOTS OF YEAH's and MMM's]

... must change.

Absolutely, yeah.

Um, because then you've got family that are at you. Er, what you going to do, you know? I've got three children and they'd more than likely all have a different opinion. And then the husband and… yeah, I sort of think it's, it's very hard to… well in my opinion now to say in all honestly I would do A or B.

Mmm.

Um…

[LOTS OF MMM's]

But you've got to sort of… I guess, um, they just want to give an idea of which way

you're leaning…

Yeah.

… I suppose.

Yeah, yeah.

Mmm. Ok. And on this side of the table, what… what do you think you would do if you were invited to do a trial?

I'd have to listen to my GP and, and I'm assuming he would send me to a specialist of some sort, um… and take their advice on board and definitely discuss it with my husband and family, so yes, I'm agreeing with…

Leanne.

… Leanne.

The two Leanne's.

[LAUGHTER]

Well I was invited to go on the clinical trials through the Westmead Hospital for a condition that I had. I didn't think I would go, but I did. But I was actually rejected because… um, there was a problem with my lung.

Mmm hmm.

So I couldn't do the trial. But, um… I didn't think I would go on the trial but when it came to the nitty gritty I was prepared to do it. And I was quite depressed when I didn't make the trial.

[LAUGHTER]

So (laughs)… um…

Ok, so what about a trial like this?

... this one's a bit different. This is a low-grade and it's not terminal, I don't think I would go for it. I think I would prefer to just go and get monitored individually through a GP.

Mmm hmm.

And take their opinion, um… I've been involved at the moment in too many clinical trials and chemos and radiotherapy and I don't like where it goes.

Mmm hmm.

I'm horrified to be honest.

Mmm.

At what can happen. I've seen too much and that's made me… very much that I would want to sit back, wait. While it's low-grade there's no need for it. Chemo's quite horrific when it's high dose.

[LOTS OF MMM's]

Really…

Weren't you saying radiography?

Oh, yes, it was.

What's the difference then?

Radiotherapy… oh, sorry.

Thank you, um…

[LAUGHTER]

What's the difference?

Between?

Radiotherapy and chemo.

Yeah, radiotherapy and…

Between radiotherapy and chemotherapy?

Yeah.

So radiotherapy is a local treatment. So it's directed at the breast itself. Um, and it's using x-rays to, so it's using the, the radiation to, um, kill, try and kill cancer cells.

Yeah.

Um, chemotherapy uses drugs or medication to try and kill cancer cells and it's what we call a systemic treatment.

Yeah.

So it goes through the whole body, um, and it's designed to try and kill any cancer cells that have already moved, um, out of the breast to other parts of the body.

Oh.

So that's why it's not used in DCIS. Um, it can be used in invasive breast cancer. But in DCIS the cells are all entirely within the breast. So that's why we use the local treatments of surgery and radiotherapy.

So have they got, um, statistics for people that have had the DCIS, um, they've had the operation and is there any long-term, this person's lived for 10 years and… there's been no more cancers? 15, 20… you know, is there anything that, any… statistics on that?

Yeah, so there are statistics, um, following up those women in the long-term and generally they have a very, really good prognosis. So, um… um, you know, many of them will… generally, you know, their life expectancy is the same as other women. So they'll be, um… some proportion of them who will, er, later on have breast cancer and be treated for that, which in most cases will be successful as well. And then there's some of them who don't get breast cancer. Um, but generally, you know, the, the overall outlook for those, the prognosis for women with DCIS is very good. Um… but some of them are then living in the long-term with some side effects from the treatments they've had.

Such as heart problems or…

Yeah, possibly, um, er, you know, scars from their surgery or, um… you

know, in some cases kind of pain that might have (?) behind. It's, it's quite variable between people.

Ok, so I think we had maybe two people who said they would rather have treatment. And was it everyone else was kind of leaning towards maybe not having treatment?

Yeah

[LOTS OF YEAH's]

I suppose it would depend on what else is wrong with you at our age?

Yeah.

Mmm hmm.

Sorry, I have a question, um, that might change my outlook.

Mmm.

So the, with DCIS it's actually contained in the ducts and if it becomes aggressive… and it's still monitored as far as regular mammograms maybe even twice a year or… whatever, and that it turns into breast cancer, you then still have that option of the treatment at a very early stage of… of breast cancer, don't you?

Yeah. Um, so in most cases, so in the trials for example, they're… the women in the trials will typically be having a mammogram every year. Um… so, yeah, if they stick to that and they go for the mammogram every year any… invasive breast cancer that does arise…

Will be detected early.

… would like be still early.

[LOTS OF MMM's]

Yeah, exactly.

Then I've changed my mind.

[LAUGHTER]

A woman's privilege.

[LAUGHTER]

So you feel like having a mammogram once a year would be… you'd feel comfortable with that as…

Well if, if… if that's considered to be enough with, um, getting breast cancer early.

Or you could go twice a year.

But you could have it twice a year, couldn't you?

Um, you could potentially…

If, if you're at high risk of…

Mmm.

But my, er, it's, it's two years, every two years.

Yes.

But if you feel, um, that there's something wrong like I did, I went and had another one. And I mean, you know, you can have as many as you like, so I think…

Yes. Did you pay for your second one?

No.

No.

No, I didn't. No, I didn't.

I just thought the way… somebody once said to me when I did it that it was every two years but you could have them whenever, but the extra ones you…

No, no.

… had to pay for. But then to me it would be worth it.

If something's detected… exactly but yeah, no, I didn't. I didn't have to pay and I had an ultrasound as well, so… and I go every 12 months because of the family history. My mother, my older sister and her, her daughter.

Well that's, that's right. It's family history isn't it.

They coached me to go every 12 months.

[A FEW MMM's]

Ok, so, um… I think a lot of you have got, you know, quite strong leanings to which group you would rather be in. Do, do any of you feel like you would be quite happy to go into a trial where it was…

Random.

Yeah.

… designed by chance, random?

No.

No.

No.

You think you would?

Yeah.

Didn't you say before that you would rather have the treatment?

Yes, I'd rather have the treatment.

Yeah, but don't forget it's random.

Yeah, it's random, yeah, yeah.

So you might go into the other group.

No, I'd still… I wouldn't mind a trial.

Oh, ok.

So how would you feel if you were allocated to the non-treatment group?

All right. At, at least you're aware of it. You know you can go to your doctor. You know you can go and get other X-rays and what have you.

Mmm hmm.

Do these trials run over a set period of time?

10 years.

Yeah, um, and so initially they all have a, um, a goal for the number of people getting into the trial and then they all have a protocol for how long they're follow-up. And it's, it's generally about 10 years. So they want to, you know, because DCIS can take a long time to…

[TALKING TOGETHER and LAUGHTER]

Are we all going to be here?

[LAUGHTER]

It's a long time if you change your mind that way, it's a long time to ride it out (laughs).

Yeah, um, and you can always change your mind and just withdraw.

Oh you can, ok.

Yeah.

But otherwise, yeah, it is, it's a… it's about 10 years where you're having your visits for your mammograms and your, you know, they're sending you questionnaires and things maybe following you up and… so yeah, it's a long process.

I mean they're keeping, they're really keeping an eye on it. That, that's the thing.

Yeah.

Mmm.

And if you are diagnosed with the low, like would you want to go to… I mean for me personally, would I want to go for the surgery if it was low?

Yeah.

[A FEW MMM's]

Probably not.

No.

So if you went into the, if you went into this, er, clinical trial and your, like in my case I was put into the group where the surgery was, and I didn't want to do it (laughs), so you pull out? No problems? No questions asked?

Yep.

Good.

Yeah, nobody can force you to have surgery.

Surgery, yeah.

So I mean I'd be happy to put my name down, hoping that I got into the…

[LAUGHTER]

Yeah, I'd be like that too.

Yeah, I think we can all do that one, if you could pull out of the option you got that you didn't want (laughs).

Mmm.

I just thought when I had that diagnosis of the bowel cancer, I just thought I don't want this gruesome thing with me in my body.

[A FEW MMM's]

And I, I said to the specialist, you know, just out. And then I said to him, what do you think? And he said, yeah, that's his, that's exactly what he would have advised me. But they go out of their way not to advise you, it's your choice.

[A FEW MMM's]

And I was so grateful to get rid of the blasted thing.

[MMM's]

I just thought it was percolating away there, growing bigger, urgh!

[LAUGHTER]

Well naturally you'd want that out.

Yeah.

A cancer like that you'd want out.

Yes.

[LOTS OF YEAH's]

My mother-in-law had bowel cancer and had it out and I think it was like 25 years ago, and she's still going… she's 91 now, so… (laughs).

Oh, that's pretty good.

Yeah.

[LOTS OF YEAH's]

But they say it goes in families too.

Yeah.

But I didn't want it…

My family had, had bowel cancer. My father died of it. It's very unusual apparently to have both parents with it.

Both parents?

Both parents had it, yeah.

Do you get checked?

Oh yes.

[LAUGHTER and TALKING TOGETHER]

... and he's in the army….

And DCIS (laughs).

… and the medicos there found out. my husband was just under 50 when he was diagnosed, he's got to have a colonoscopy every couple of years and he… not real happy with that.

It's not that bad.

Oh no, but he just hates the preparation. He reckons that the surgery and the anaesthetic, that's fine. But he says the preparations a shit day.

[LAUGHTER]

And I say, I know, I do it every couple of years (laughs).

Yeah, you've, you've got to sit on the toilet the whole day, well several days.

My husband's going in for his tomorrow and it's not as bad as that.

[LOTS OF NO's]

It's horrible stuff.

Tonight's the, tonight's the horrible bit.

Yeah. Enough about that.

Sorry.

[LAUGHTER AND TALKING TOGETHER]

We lost the plot.

Well this is a little bit off track but occasionally there are men who have breast cancer.

[LOTS OF MMM's]

Are there, have they come across any men with this? Or they're not being screened though, are they? So…

Yeah, so very, very rarely it can be diagnosed in men.

[LOTS OF MMM's]

And it's in the milk ducts, which they would not very likely have.

Yes, yes. I realise that.

Yeah, so they do have milk ducts that are kind of…

Dormant (laughs).

Like the rest of them.

[LAUGHTER and TALKING TOGETHER]

Well, um, on that note…

(Laughs)

… just as we're sort of getting towards the end of time, um… I would like to ask you to do one more questionnaire on that document two. Um, and, and that'll be the last thing. So is there anything else that you really want to say before we… kind of wrap up, that we haven't covered?

So what degree are you doing this study? Are you studying DCIS at Uni? Or are you just doing the questionnaires to see if more work can be done? Just curious.

So at the moment I'm doing, um, a few related studies where I'm basically talking, getting people's opinions about some… er, but this study which is with women in the general community. I've, I've also been doing interviews with clinicians who treat women, about their opinions about all these things.

All on DCIS?

Yeah. And also women who've had DCIS themselves.

Mmm, yeah.

So that's… that's kind of the current research program which is all about what we can learn about the communication and the decision-making.

Yeah.

Yep.

Thank you.

[TRANSCRIPT ENDS 01:45:26]

END

Interview: DCIS FG4 2018.05.23

Interviewer: **BOLD TEXT**
Interviewee: PLAIN TEXT
Pauses: …
Unclear words: (?)

[TRANSCRIPT BEGINS 00:12:01]

Um, well let's get started. So DCIS it sounds like it's a bit familiar to some of you and, and not others. But, um, I'm going to start by talking about something that probably is at least a little bit familiar to most of you, and is relevant to DCIS, and that's breast cancer screening.

So what is breast cancer screening? Well in Australia we have a national program that provides screening, free of charge to women, to look for early signs of breast cancer.

Now it's important to understand that when we talk about screening it's something that's designed for women of a certain age in the general population who are well. It's not for women who have a breast symptom (like a lump or pain or discharge) that needs investigation – that's a separate process.

The aim of screening is to find women who have cancer early, so they can be offered treatment early, in the hope that earlier treatment will lead to a better outcome. And the main goal of screening is to reduce the number of women who die from breast cancer.

So the best available method for breast cancer is a procedure called a mammogram, which uses x-rays to make images of the breasts. And the whole idea is that these x-ray images can show up cancers or abnormalities may be so small they can't be felt as a lump.

So if the mammogram images look abnormal or suspicious in some way, the woman gets called back to have extra tests which may include a biopsy. That means using needle to take a small sample of cells from the breast to look at under a microscope, for the purpose of confirming the diagnosis. So from looking at what that small sample of cells under the microscope, a pathologist will either say actually everything's ok, and it was a false alarm, or they'll diagnose breast cancer or DCIS.

So DCIS or ductal carcinoma in situ is a condition that can affect the cells inside the breast.

Typically, DCIS doesn't cause any symptoms like a lump that you can feel, so it's found through screening. So before we started doing screening in the population, it was hardly ever diagnosed. So in the past, women may have had DCIS but because they had no symptoms and there was no screening, they wouldn't have known about it. Whereas nowadays we have widespread screening and so DCIS has been diagnosed a lot more commonly than it used to be.

And part of the challenge with this condition is that it's almost like DCIS is a new disease that we've kind of only really discovered through introducing these population screening programs, in Australia and other countries, and that's happened from about the 1990s. And so everything that women are told when they're diagnosed with DCIS, and everything that I'm telling you today, is the best information we can give based on current scientific knowledge about DCIS. But it's important to understand that that knowledge is currently limited. And there are still some things we don't really fully understand yet about this condition.

Now I'm going to show you a diagram with some numbers now – don't worry too much about the numbers, it's just to give you an idea of how many women are affected by DCIS.

So if you imagine that each one of these dots represents one woman. If we take 1000 women in Australian who have breast screening over a period of 25 years, in total about 81 of them will receive a diagnosis of one kind or another, as a direct result of the screening mammogram and the further investigations that follow.

And if we look in more detail at these 81 women, we find that 16 of them have DCIS and the other 65 have what's called invasive breast cancer – and I'm going to explain the difference in a minute. So, as you can see, the women with DCIS are a smaller group – so they're about 1/5 of the total number of women diagnosed through screening.

So let's talk about what is DCIS is in a bit more detail, breaking it down

word by word. So ductal means it relates to a duct or tube that carries milk to the nipple. Carcinoma means the cells show abnormal features similar to cancer.

'In situ' means contained in the original place. And the 'in situ' part is very important to defining DCIS. So this illustration shows how DCIS is different from 'invasive' breast cancer, which is really just a more precise term for what we commonly understand breast cancer to be. So based on what they can see looking at the cells from the biopsy under the microscope the pathologist will diagnose that it's either DCIS, which means the abnormal cells appear to be entirely contained within the milk duct, or invasive breast cancer, which means those cells have moved outside the duct into the surrounding breast tissue. Um, and by the way I forgot to say before, if anyone sort of has trouble seeing the slides from where you are we've got a printed copy if anyone would prefer to follow on there.

So DCIS and invasive breast cancer are different but they're related. In DCIS, the abnormal cells are contained within the milk ducts and have not spread outside the ducts into the breast tissue. And as long as those cells remain contained like that, DCIS is not a life-threatening condition at all. But DCIS might develop into invasive breast cancer later on, and if that happens the time frame for it to happen is really variable, so it could take many years or it could happen more quickly.

In invasive breast cancer, some of the abnormal cells which were previously just inside the duct have actually taken the next step and spread into the breast tissue around the ducts, and once that's happened, the cancer could then spread to other parts of the body, which is when you really have a problem.

But it's good to remember that although invasive breast cancer has the potential to be a life-threatening disease, these days the majority of women diagnosed with breast cancer will be treated successfully and will survive their cancer.

Now this slide just shows some different phrases that are used to describe DCIS. And none of these are wrong but it can be a bit confusing for women because DCIS is described both as a type of cancer and as something that's

not quite cancer.

So if you haven't heard the term DCIS before, you might have come across some of these, um, phrases like Stage Zero breast cancer or non-invasive breast cancer or pre-invasive breast cancer.

So what happens after DCIS is diagnosed? Well, as I've said, DCIS itself doesn't affect a woman's health, but it has the potential to turn into invasive breast cancer. Now we have reason to believe that this progression to invasive breast cancer might not happen for everyone, and I'll explain more about that later. But the bottom line is that right now we can't reliably predict which of the women with DCIS will get invasive breast cancer or how long it may take before that happens. And that's why virtually every woman diagnosed with DCIS is given treatment, to try and prevent that progression from ever happening. And I'll talk shortly in a more detail about what that treatment involves.

But firstly, I just wanted to, um, come back to asking about whether and what you've heard about DCIS before? So a couple of you mentioned it already. Um, has, has anyone else realised that they've heard of, of DCIS before?

No, I haven't heard the term before.

[A FEW NO's]

I wasn't familiar with the term. I know breast cancer but not, not that.

Ok. And was it you that said that your friend…

Yeah.

… so this all kind of matching up with…

Yes. Mmm.

Ok. Um, for those who, who hadn't heard of it, do you feel you understand what I've said so far?

[LOTS OF MMM's and YEP's]

Anything unclear or confusing?

[A FEW NO's]

Any questions going through your head right now?

Er, what's the age… is it over, anyone over 50's recommended to have it every… screen, er, every 2 years? To a certain age or… from what I understood only up to about 50-75? Or…

Yes, so…

… should they do for the rest of their life?

Well the, the current recommendation, um, is from 50-75 as you said, every 2 years, yeah.

I have a friend, she is 89 and she's just always kept up the every 2 years and her last screen, 6 months ago, they picked up a… and I don't know if it's a DCIS, but it was a very small lump from the screen. They've actually taken it out.

Mmm hmm.

And she's 88.

Yeah.

So if she hadn't have kept her up her screens it may have became… um, invasive into her body.

Yes. So…

It could have been a life saver.

… women over 75 can still access the screening program, they're just not, um, really actively invited. So they, they stop receiving reminder letters but if they choose to they can still have screening.

Yeah, I got mine last month.

So…

So I'm doing it.

Yep.

'Cause I didn't do it last year, but I'm really making an effort to do it.

Mmm hmm.

'Cause at 66 you start, you know, thinking about things.

Because the register with the, um, breast screening they, they will remind you every two years, which I think is great because otherwise I, I would forget and I'd never…

I got my letter last…

If they don't do it they'll send you another letter.

Oh right.

Yes.

Well I got my letter so…

Which is good, it's good.

[TALKING TOGETHER]

… and I changed jobs and I sort of…

It's like pap smears.

Oh, I know…

So I'm booked in for next week. Luckily it's near where I am.

… but the second time my mother had the breast cancer it was a grain of rice.

Mmm.

So she'd already had a mastectomy and then she had the other, it was a grain of rice.

So you could pick that up.

Yeah.

Were you going to say anything?

Yes, just, just to mention that there are other types of breast cancer like lobular cancer, so just to… make it a bit more comprehensive, maybe you could say a few words about that… when we deal with breast cancer it is not the only type of breast cancer that we deal with.

Yeah, so, so other than invasive cancer there are other types of in situ cancers, so in the case of lobular carcinoma in situ, that means that instead of being in the duct it's in the lobe. So a slightly different part. Um, but it's also a, a… a carcinoma in situ, so it's contained and hasn't spread. But, um, DCIS is a bit more common than LCIS.

So I just wanted to come back to, you know, from, from what I've said so far, what you've learned about DCIS, what are your kind of thoughts and feeling about it?

So my understanding is it can be picked up with the breast screening?

Yeah.

I think a little bit more discussion in a medical… um, topics on, you know, TV should be more frequently.

Mmm hmm.

Because apart from the breast-screening letter I never see anything around, you know, reminding people to go and get a check.

Ok.

And when you have the screen is it more precise to check, to see that, that it is the other type of cancers, breast cancers? Like, do they sort of really have to look into the, er, the, um… you know, the film to, to find it? Or does it show up easily?

Well it, it certainly shows up, um, you know, they pick it up with their routine images and their routine reading process. So it's not, um, like a

special kind of extra examination or anything. But, um, both invasive cancers and DCIS are, are shown up, um, quite, you know…

With, with the current machinery they use.

Yeah, so it's not that it will always pick up 100% of everything by any means, but, um, yeah, it is, it's part of the routine process that they're looking for DCIS as well as invasive cancers.

Ok. Um, I'm going to talk a little bit more about how DCIS is treated. So the goal of treating DCIS is to prevent the abnormal cells from turning into invasive cancer. And overall it seems that our current treatments are doing a pretty good job of achieving this goal.

Surgery is really the primary form of treatment for DCIS. So that's doing an operation on the breast, in hospital, under general anaesthetic. And doing surgery also means the pathologists get another chance to look at these cells under the microscope, but this time they've got more to work with because they've got what's hopefully the whole abnormal area to look at, um, rather than just that small sample they got from the biopsy. And in most cases this will confirm that it really is just DCIS as the biopsy suggested. But in some cases they find after surgery that the patient actually has invasive breast cancer that the biopsy didn't catch. So that patient's diagnosis will is changed or upgraded at that point.

Now there are different types of surgery that a patient can have, and surgery may also be combined with another form of treatment. And exactly what each patient has will depend on features of her DCIS (such as its size and grade – which I'll talk more about grade later) as well as characteristics of the woman herself (like her age, her overall health, and her preferences).

So I'm going to tell you in a bit more detail about the 2 most common options for treating DCIS. And both of these approaches are considered very effective and survival rates for women with DCIS are excellent.

So treatment for DCIS most commonly involves breast conserving surgery and then radiotherapy. This means having an operation to remove the part of the breast that's affected by DCIS and a small area of healthy breast

tissue surrounding it. So this may also be called a lumpectomy or wide local excision.

And after breast conserving surgery a pathologist will look at the breast tissue that was removed. And sometimes, if they find there's a bit more disease than they originally thought, the woman may need more surgery.

Now everyone responds differently to surgery. Some side effects happen to most people, others happen only occasionally. And some side effects happen straight after surgery, and others may take longer to develop. But some of the common side effects include things like pain, numbness, and bruising around the wound.

So that, is that done on the same day or does the person come back for that extra surgery?

If they, if they need extra surgery that will be done later.

Oh right. Ok.

Yeah. So after this surgery, the patient has radiotherapy, normally every day for a few weeks. Radiotherapy uses x-rays to destroy any abnormal cells that may be left in the breast.

Receiving radiotherapy is painless. But women may experience some side effects. So during the radiotherapy treatment, the skin of the breast may become red and dry, a bit like sunburn, or the skin can become darker; and women often feel more tired than usual. And these side effects usually settle down after treatment finishes and things get back to normal.

Now during breast radiotherapy, it's possible that a small dose of radiation may reach the heart or lungs, depending exactly where the DCIS is. And some studies have suggested that women who had breast radiotherapy in the past may have a slightly increased chance of having heart problems, like a heart attack, later on in life. So doctors these days are using more modern techniques when giving radiotherapy to try and minimise this problem as much as they can.

Now instead of having breast conserving surgery plus radiotherapy,

sometimes a mastectomy may be recommended, for example if the area of DCIS is quite large compared to the total size of the breast or for some other reason. Now this is a bigger operation which means there's a higher chance of complications and a longer recovery time.

So all the side effects I mentioned for breast conserving surgery are also relevant for mastectomy but because the surgery is more extensive, there may be additional side effects such as stiffness in the arm or shoulder.

And obviously mastectomy affects the body shape and it can affect women's body image or how they feel about their bodies. Many women have the possibility to consider breast reconstruction surgery to try and help with that.

And just in case you're wondering at this point I want to mention that chemotherapy is not a treatment that's generally used in DCIS. So the main treatments are, as I've outlined: those different types of surgery and radiotherapy.

So what are your thoughts and feelings about, um, that information about treatment for DCIS?

What I know from my mother (who had?) breast cancer, she had, having just the one breast and even though she had a prosthesis, um, because she felt uncomfortable. You know, you feel quite lopsided and the rest of it. Um, that was, it was a bit problematic, you know. Well, it was not comfortable. Also, she had, um, the nodes taken out from under the arm. And then she developed lymphedema and she's had to wear a stocking on her arm there forever more.

Mmm hmm.

And then 10 years after that the other breast got the cancer as well and that was the one that just had a, a grain of rice. And she had a mastectomy then. Um, and basically she said, look, she said to me look, have them both done and make sure you tell the doctor to research the, um, the lymph nodes very carefully so that they take as minimal if they have to as possible. Because it's awful having this, er, you know… and you've got to be so careful. It gets, um, it doesn't get, um, cellulitis as soon as you… you know, if you scratch your arm or anything like that you get cellulitis. And I mean, that can, if it gets bad you have to get your arm

amputated. It’s terrible. You’ve got to get antibiotics straightaway. So that’s just something that, you know, that’s just something that my mother (?). And looking at all the problems and everything she’s still having, you know.

Mmm hmm.

At what age did she die?

She’s not dead.

Oh, right.

She’s a miracle patient because she had also ovarian cancer that developed, er, to 10 pounds in 10 days.

[GASPS]

And the reason, they suspect the reason she got the, the breast cancer was because she had the ovarian cancer. ‘Cause it’s often linked.

Mmm.

Mmm, same genes.

When did they decide to take the lymph nodes out?

Yeah.

‘Cause I had a friend, she had hers out 15 years ago and then just recently she, cancer was in the other breast and had a mastectomy.

Mmm.

But on the other arm it was the, the lymph nodes only. So because of DCIS is going from contained into… further tissue?

So that would… yeah, that would be more relevant for, um… invasive breast cancer, um, that you would be more likely taking the lymph nodes, which is some, the nodes under the armpit and that’s what can often lead to the problems in your arms.

Yes, she’s had that.

So, um, yeah, it… that may or may not be involved in a mastectomy for DCIS. Um, but certainly the, if you're having the breast conserving surgery then the lymph nodes are not affected, so you don't have, um, those, those really, um, quite major side effects.

Ok, what do, what do other people think in response to, um, learning about DCIS treatment?

Oh, it can be quite devastating. It changes your quality of life.

Mmm hmm.

Well…

What do you mean?

Well all the other things, you know, the… being detected, having an operation, all the possibilities that could go wrong. Thank God my mother lived to 95. Mind you, the last 4 years were in a nursing home and I wish she had've died when she had the heart attack when she was 91 (laughs).

[LAUGHTER]

So I either have, well possibly cancer on my father's side, but that's… a few people separating, or (?) on my mother's.

Mmm, I think it's also… I'm got a young daughter and, um, I think a good relationship with a GP who keeps these discussions ongoing…

Mmm.

… um, so that by the time my daughter reaches 50 it's not I've got to rush and get a, you know, it's, it's something that's just talked about naturally and, er, be prepared and, and, um, have checks regularly. I think it's, I think your GP possibly is, for a lot of people, first port of call.

Mmm.

I think if they can familiarize patients as time goes on, um, people might be a bit more prepared. I think sometimes the shock, I mean it's still a shock when it happens.

Especially if you know it's in the family.

Well that's right.

Can I just comment as an ex-GP that I started sending women for screening at 40.

Yeah.

Not 50.

No, that's…

So from 50 you get the recall, but you can start at 40.

Yeah. I've been having mine since I was 36.

Probably because of your mother. But it's in your family.

But I had, I had a lump there, was like a little pea on the breast and, and because of my mother's history they let me have a scan. But it was, it was nothing. It was just a cycle thing.

Mmm.

And it… there was nothing there. And when I came back to have another check, it was gone. But from then on I still kept going. Yeah.

But thankfully with all this research in medicines today we can, can save our lives.

[LOTS OF MMM's]

But also, but also a GP can, I had a, um, malignant melanoma picked up in 1983 by a GP in the city and she just took one look at it and rang a surgeon up and I saw him the next day. And, that was just a GP that picked that up. I mean I noticed it was new.

Mmm.

It hadn't been there last summer sort of thing. Um, but I, you know, I really do think that GP's are…

Was it itchy or…?

It was just, it was my husband actually, he said I don't remember that being there last summer. 'Cause all through winter you've got pants on and I suppose I hadn't noticed it much. But she just looked at it and said, oh, I don't like the look of that at all.

Mmm.

So that was a long time ago.

So was it a dark colour or…?

Yeah, it was quite dark.

Oh.

So could I just ask if sort of hypothetically if you can imagine that you've just been diagnosed with DCIS and then you were told about these standard treatment options, how do you think you would be feeling?

You're in shock I think.

Yeah. In shock.

I think the next step is how you're approached by the medical profession.

[A FEW MMM's]

Um… you know, you hear some horror stories I'm afraid, um…

Mmm.

… very curse… cursory comments and… it's a bit well this will do, this will happen now, now, now, now. I think there needs to, yeah, I think people need to be treated very carefully and very gently through the decisions they have to make.

Or they don't tell you and unless you don't get the information unless you ask the right questions.

That's right, but…

That's the other thing. But this type of thing is creating the…

… but that's going to be hard if you're in shock with no medical…

… yeah, but I mean once the shock wears off…

Yeah.

… I mean, and this type of thing…

Oh, I agree, yeah.

… should be, this sort of thing is like to be creating more awareness of DCIS.

Yeah, yeah.

Which I think is, is the main, is, you know, the main thing. And I also think you have, also, as you said, happens if you have a really good GP, which I do. I have a fantastic GP. And, um… I think that, you know, that a lot of people these days don't have a, don't have a regular GP. They just go to a Medical Center if they've got a sore throat or something like that. So you don't have a relationship…

It's such a shame.

… yeah, you don't have a relationship with a, you know…

Unfortunately they don't keep doing it forever.

Oh that, but that… yeah, but that…

I saw my GP for 25 years and she retired last Friday.

… (UNCLEAR)…

[TALKING TOGETHER]

I've been through the same thing. And I've also had since that happened I, I've gone through 3 GP's until I found this one now.

Well I've heard of that sort of thing too.

But it's taken me quite a while. It's taken me quite a while.

To go from one to the next.

You shop around.

But it's difficult.

Yeah, it is difficult.

I've got nothing wrong with me so I don't have a relationship with a GP.

Oh, but we've, we had a husband/wife. We're lucky enough when we moved into the area where we live. So we've been going to the same people for 30 years. Um, and they're about my age, um…

That's the problem.

… you know, each time I see Catherine I say, now, you going to be here for the next few years?

But all my family have been to those two people and it's sort of been first name basis and they know our history.

Ok…

But… I think it's really important to create, to start to create public awareness about this.

Mmm hmm.

Because most, most women I know they don't have a clue. They don't know. They just think it's breast cancer. Or they either have it or they don't have it. They don't know about this. And because it's undetected, because it's… there's no effects basically and it's detected only through…

An X-ray.

… and x-ray, if they don't go and have, you know, a mammogram regularly…

Or actually go every year.

… yeah, it's (UNCLEAR)…

Or even start doing it before you're 50.

… create a public awareness about it I think is really important.

Mmm.

Ok. And so what about if… if you could imagine once you're diagnosed, if you were sort of, um, given… er, some options, say between those two options for treatment, 'cause sometimes, um, the woman's preferences come into it…

Mmm hmm.

… and how, how would you go about deciding which kind of treatment you'd want to have or…

I guess it depends on the age.

I would start by getting as much information as possible.

[LOTS OF AGREEMENT]

You can't start by saying, I mean once you get over the shock, you then, and I'm sure it's a terrible shock, um, you then have to start… so your, your GP or whoever gives you the results will have to write down for you what you have and, and the various possibilities. Go away, think about it and… um, I, I was in the situation where I had an issue with my eyes that had to be dealt with very fast, and my, my specialist said to me, go away, think about it, don't wait too long. But I went home and then I Googled it and I found out what I needed to know and then I made the appointment the next day, once I'd read it up. So it would be the same. You would have to…

The best informed decision.

Yeah.

You make your best decision I think would be different for everybody really, wouldn't it?

And, and if you're not happy with the doctor you're sent too…

Get a second opinion.

… ask for a second opinion.

Yeah.

And I, I think that's a problem that a lot of young people have, that they're scared to ask for second opinions.

Mmm.

Because they're not used to it.

Mmm hmm.

The decision might be based on whether it, it's in the family, whether it's hereditary.

Mmm.

Could… you know, make… help make an informed decision as well.

And that would surely be asked of by the medical profession, the person… I mean, if you're diagnosed that would be one of the questions surely? Do you have any…

Well one would hope so, because…

You'd hope so.

… once upon a time you were just told this is what you've got, this is what you're going to have. Now that's in the past. It shouldn't be happening today.

But quite often you were told your grandmother died of cancer, but you didn't… I mean, I didn't necessarily know. It could have been anything.

Yeah, well even when my mother had the ovarian cancer and they were going to (UNCLEAR)… they, the doctor, she had the best doctor in Australia apparently, but he sent her stuff, her DNA and that, over to America. And it came back and it was negative as far as, um, um… hereditary. And we went through, like she had a very large family, her mother had 11 sisters and brothers, and on, you know, right through the family. And they had lots of children and nobody had any of this. And we went, you know, they asked us particular questions and, and anyway… but we think now that my mother worked in a perfume factory when

she was younger…

Oh.

… and even though she used to wear a white coat, she used to come home and her white underwear was pink. And all this business with the…

Dyes.

… talcum powder stuff.

Oh.

And they think that that could have been the trigger for…

Oh.

… for, for that.

Mmm, yeah, especially if all the rest never picked up.

You know, that could have been the trigger for that. And apparently a lot of people who have worked in cosmetics that's, they've come from all (UNCLEAR)… and they, you know, the face powders and all the different things, like I say, her white underwear when she came home (?) they were pink. And that sort of, sort of came to light when that sort of started. So whether or not the effect that she's had, still had the ovarian cancer and somehow or other it's still came… once you get that it's still a risk, well, you know… we just assume (?). But often they say once you get one you've got a, you know, quite a possibility you'll get the other.

Yeah, and just coming back to, um… Julie, when you said before that it would depend on age, kind of what treatment, what, what did…

Well, I mean, you know, I don't know, when you're in your… say 70's, you might decide to have both breasts taken out 'cause you're just going to feel more comfortable. Where if you were younger you would possibly want to keep more of your body shape and, and put a bra on with the, you know, stuffing in it just to make sure, so that physically it, it still looks like you've got two breasts.

Mmm hmm. Ok.

Yeah.

But there's a lot of options to do with reconstruction, you know. I mean I've got a couple of friends who had their surgery, their mastectomy and had the reconstruction at the same time.

Yeah, sort of save the pain and do it all together.

'Cause it was a very positive outcome. And that was 15 years ago. So…

It must be even better now.

So if a young person, I'm thinking of my daughter, um, was diagnosed with DCIS, now they wouldn't necessarily do a mastectomy unless they could see that it was invasive, would they?

Um, no they may do a mastectomy even if it's not invasive.

Right, so if she said I would like to go on and have children and breastfeed, that could be a consideration for her, that she would… have one breast. But they say that they wouldn't advise that or…?

So, um… basically the, whether a mastectomy is recommended will depend on things that could include, you know, how extensive the DCIS is. So if it's in just one small part of the breast versus, you know, a bigger part, even though it's still not invasive, then they might recommend the mastectomy. Or, um… but if it's a, even if it's in a small… you know, but then the other thing that comes into it is the person's individual circumstances.

that's right.

Yeah, so what she… what age she is, what she might want to do in the future, yeah.

Would it be difficult for a young person to actually get diagnosed because of requiring the, the mammograms?

Yeah, so…

I mean, how old would you be when you went for your first mammogram?

Yeah, so typically you are 50 plus.

50, 50.

Yeah, but I mean…

Unless you had a family history.

… yeah… but I understand it's very difficult to diagnose younger because younger people have lumpy breasts anyway.

That's true.

Yeah.

Yeah, it is.

That's right. So it is, it's more commonly… you know, most people with DCIS would not be in the position where they're considering breast screening in the future. Ok. And any other, um, sort of concerns, um, things you might be, be worried about facing those kind of treatments?

No? Ok. So there's, um, a question that is sort of occupying breast cancer and DCIS experts at the moment. And that is, um, the question of whether some women with DCIS could actually be spared treatment?

So the experts are now doing research to try and see if they can identify women who have DCIS but have a low chance of getting invasive breast cancer in the future, um, so that in future years women like this may be able to avoid surgery and other treatment, and therefore avoid all those problems that can come along with treatment, while still having excellent survival rates.

So as I said before, it's very difficult to reliably tell who these 'lower risk' patients might be, but we do have some clues. And one of them is something I mentioned earlier called grade.

So after the biopsy is taken, the pathologist looks at the sample under the microscope and gives each case of DCIS a grade based on how the cells look, and this gives an idea of how fast the abnormal cells may grow.

So low grade indicates that the cells appear to have a lower level of activity, which suggests they may stay like that for quite a long period of time. On the other hand, high grade DCIS means the cells appear more active, so they're likely to grow at a faster rate. So it's considered important to treat high grade DCIS because otherwise those women are, are pretty likely to develop invasive breast cancer at some point. And then there's an intermediate grade which is somewhere in the middle.

Now you might be wondering, if virtually all women with DCIS are having treatment, so having surgery to take the DCIS out, why am I saying we think low grade DCIS may stay as it is for many years and not cause problems, even without treatment.

Well, we think that based on a small number of research studies similar to this one.

So as an example I want to tell you a bit about a study that was done in America using a database of routinely collected information about cases of DCIS. So these blue bars represent all the patients in the database who had low grade DCIS. So as you can see, the vast majority of these patients, almost 9000, were treated with surgery, so that's the typical thing to do for DCIS, that's recommended in all the guidelines for doctors.

But a small number of patients, a couple of hundred, didn't have surgery for some reason, and we don't really know why not.

So this database kept track of key events that happened to these patients over many years, for example it recorded if any patients died from breast cancer. And what the researchers in this study did was compare the large group of women who did have treatment and the smaller group who did not have treatment, after 10 years. And they found that the women who did have treatment had a very high survival rate, so 99% of them had not died from breast cancer, so they were doing really well. And the research also found that among the women who did not have treatment, 99% of them also had not died of breast cancer.

Mmm.

So this led the researchers to suggest that there may be a subgroup of

women with DCIS who are likely to have really good health outcomes over time, whether they do or don't have the typical treatment of surgery immediately.

Mmm, and that seems to be related to grade, does it?

Yeah, so this is only women with low-grade.

Mmm.

Whereas, um, they also looked at women with high-grade and that difference was much bigger.

So what I want to ask, have they perfected a blood test for these things?

To determine the…

The DCIS or even the… other cancers, breast cancers?

Blood test?

No.

No.

So it's based on, um…

'Cause you can have a blood test for ovarian cancer if you think you're going to get it.

Yeah, so there's no blood test.

CA125. I have one every year.

So this is all based on, er, the diagnosis is based on the biopsy and if the woman has surgery then, as I said, they'll also look at that as to confirm the diagnosis based on what the tissue, breast tissue looks like.

Do you know if these survivors without surgery and the survivors with surgery, did they have any, any cross-referenced, um, health issues or… DNA or any kind of thing that would have, that would… um, you know, set them apart or not set them apart from each other?

So, no, from this study, from this kind of, um… it's called a retrospective study. So they just looked at this database, which has some quite basic information.

Ok.

So there's a lot of information we might like to know that's missing.

Mmm hmm.

So, yeah, this kind of research it sort of suggests ideas to us, but it's hard to draw firm conclusions about what we want to know.

Ok.

The one percent… that weren't alive at the end of that time, are they only including people who died of, of breast cancer?

Yeah, in this particular statistic that's…

Ok.

… kind of whether they died of breast cancer. So that's, yeah, breast cancer survival.

Ok, so, so basically from kind of a, a small number of studies similar to this, um, we've got this idea of a subgroup of DCIS that we could call 'low risk DCIS'. And that's a big focus of current research, to investigate if women in that category could possibly avoid treatment.

And there's a range of factors that are relevant to trying to define this category of DCIS – grade is an important one but it's not the only thing. But there's lots of other things to take into account that I'm not going into.

So just coming back to this diagram that I showed before, I just want to give you a rough idea of how many women with DCIS might be classified into this of lower risk category. So of our, um, 16 women with DCIS from before… we think that about 5 women have what could be classified as lower risk DCIS based on their biopsy.

For the other 11 they're considered to have a higher chance of getting

invasive breast cancer, so it's still considered more appropriate that those women do have treatment.

But these 'lower risk' women are the focus of current research, looking at whether they might be able to avoid treatment. And I'm going to tell you a bit more about that research shortly, and it's just important to remember that we're talking about this quite small and select group of women within DCIS patient population where researchers are considering a new, different approach to managing their condition.

Any burning questions right now?

Would, would you assume that if you had DCIS even low-risk that you might be asked to do screening a bit more frequently?

So at the moment if you have, er, DCIS that's low-risk you'll typically have treatment.

Ok.

That's the, that's the current situation.

But you're probably going to go into probables later (laughs).

Yeah, so I'll, I'll go on, um…

[LAUGHTER]

So there are certain studies, um, there's a type of studies I want to talk about now called clinical trials, so I just want to explain what these are.

Now a clinical trial is a research study that asks an important, specific question about a certain health condition. And clinical trials often test out new ways of managing a condition, by comparing a newer approach with whatever is the current standard care.

And trials are designed to find out whether patient outcomes (like health and quality of life) are similar for both options, or whether one option is better than the other.

So if you think back to that study we talked about before, with the blue bar

graph, as I said, it was done by looking at a database containing routinely collected basic information about past patients. Um, and as I said, it's, it's hard to draw firm conclusions from that kind of research because there's a lot of important information missing, for example details about those patients who unusually didn't have surgery and exactly why.

Whereas clinical trials are specially designed to overcome limitations of other types of research. And that's why trials are considered to give us the highest quality of evidence.

So… this illustrates the basic design of a simple clinical trial. Sometimes they can be more complicated, for example more than 2 groups, but most typically, each patient in the trial is put into 1 of 2 groups. One group of patients gets the standard treatment, while the other group gets a new treatment or a new way of managing their condition. And which group each patient goes into is determined by chance, using a computer. This process is called randomisation and it's a very important part of clinical trials. When we compare 2 groups of patients who are having their condition treated or managed in different ways, we want to make sure the groups are very much the same in all ways other than how their condition is being treated or managed. If patients and their doctors choose which treatment to have, then the patients in the different groups are very likely to be different from each other in important ways relating to their health, and that can create a bias that makes the results less reliable. So patients who volunteer to take part in a clinical trial need to be comfortable with ending up in either of the groups because they don't get to choose which one they'll be in. Randomisation makes sure that the study groups are as similar as possible. And that way, we can get the most reliable information from the results of the trial to learn about the effects of the different ways of managing the condition.

Now deciding by chance how a patient will be treated may seem strange, but it's considered an ethical thing to do in situations where we genuinely don't know whether one option is better than the other.

So there are currently 3 clinical trials going on around the world for women with low risk DCIS: one in the UK, one in Europe based in the

Netherlands, and one in the US. They all just started in the last couple of years, and together they will involve over 3000 women. Now there are slight differences between the trials, but they all have the same general design.

And the aim of these trials is to learn whether women with low risk DCIS could safely avoid having treatments that may not be necessary for them.

So the women invited to take part in these trials have to meet strict criteria to be considered suitable. They have to have low or intermediate grade DCIS (not high grade), they can't have any lump that you can feel, plus there's a lot of other more technical rules I won't go into. So remembering that this is what typically happens to women with low risk DCIS: they have treatment pretty much straight away, and we talked about those typical treatments before, so surgery of some kind with or without radiotherapy.

Of course in the following months and years, if it seems their initial treatment hasn't worked as well as hoped, or their DCIS comes back, or they get DCIS again elsewhere or invasive breast cancer, they might end up having some further treatment if needed.

So that's the normal scenario for pretty much all the women in places like Australia where there is no clinical trial. And for women who do have a trial available where they live, if they're suitable to offer the trial, they may just say no thank you, I don't want to go in the trial, I'll stick with usual treatment. Or, if they decide to join the trial... then they get randomised to either usual treatment or the other group called active monitoring. So the usual treatment group of course has the usual treatment. And the other group has a different approach where in the first instance they don't have surgery or radiotherapy. And then the patients in both groups are monitored closely, so they all have mammograms at least every year. And importantly, patients in the monitoring group may have treatment later if there's any sign that their DCIS is progressing, and we expect that to happen for some women. So this approach may be called active monitoring or active surveillance and it's actually already an accepted option for men with early prostate cancer because trials similar to this have shown that it can be a safe option for them. So these trials will test out the idea that this

monitoring approach where you only give treatment if it's needed may result in the same very high survival rates as standard immediate treatment, but with fewer side effects overall because some women will avoid treatment altogether, and others will end up having treatment down the track but it's delayed so they may have a period of some years where they're able to avoid those side effects. So those are potential advantages of monitoring.

There's also potential disadvantages that the trials will examine. For example, patients who don't have immediate treatment may be more likely to get invasive cancer – although with the monitoring of course it's still likely to be picked up at an early stage. As I mentioned before, it's also possible that some women who are diagnosed with DCIS based on the biopsy actually have invasive cancer at the time that the biopsy didn't pick up. And we also don't really know how women having monitoring will feel about not having treatment, so in the trials all the patients will do questionnaires to help the researchers understand the psychological effects of, of living with that approach, and that's a very important aspect of the studies.

Now because there's only a small and select group of women who are eligible for these trials, and because they then need to be followed up for quite a few years, it will still be some years before we have the results from these trials.

In the meantime, it's also possible that a similar study could be started in Australia and New Zealand, so women here might one day have the possibility to go into a trial like this.

But otherwise until the results of these trials come out, women with DCIS will continue to have normal treatment because we don't have strong research evidence to show that any other approach, like active monitoring, is really safe.

So what are your thoughts and feelings about these clinical trials?

Well regardless you're still going to have your mammograms.

Mmm hmm.

So, you know, if any changes will, will have to show up.

Ok, so if you didn't have treatment you'd still be having mammograms that would be looking for changes.

[LOTS OF MMM's]

Yeah?

And if, you know, you were aware of any sort of symptoms as well, like you know pain in your breast or maybe a tender nipple or something, or, or problem under the armpit, 'cause that often triggers something, and then, you know, your immune system's going, working in overtime to sort of get that going.

Yeah, that's right. So the women in the trials would be told that, you know, if they do notice any symptoms they should go get them checked out straightaway, yep.

So if they have a, if they've got themselves a pretty good doctor, they just, you know, head along to the doctor and just tell the doctor the concerns that they have, which is good because sometimes you just get load of your chest (laughs), you don't have to think about it yourself.

That's right.

You know? And that can be all part of the treatment as well, sort of thing. Because if you're fretting about this it's not helping.

Mmm hmm.

If you're in one of these trials though it wouldn't be your actual doctor, your GP, that would be running your active surveillance, it would be you guys that are running the trial, wouldn't it?

Yeah, so the actually, um, mammograms, er, would be provided by some kind of service that was, yeah, had signed up for the trial.

'Cause I've done clinical trials for other things and, yes, my doctor might know that I'm doing it but I'm going in and having regular things done with that clinical trial bunch of people, be it whatever kind of body test it is or whatever.

Mmm hmm. Yep.

But the big issue that I've noticed in some of those trials was, um, how diligent the organisers, the people that were running things, as to how diligent they were in making sure that we came to our actual appointments.

Mmm, yeah, to follow-up. You've got to keep the statistics coming in, 'cause…

I knew people that started and they dropped out 'cause they got lazy. And I often, I often wondered, you know, well how much nagging did the (laughs), did the researcher people do to actually get them to come?

You've got to be committed.

Yeah, exactly. And those, you know, those trials were finding out important stuff and if it's for a surveillance reason it's really important to come for that check-up (laughs).

Well that would be a point of discussion.

Yeah.

At the beginning, to make sure that people understand it was a long-term decision not a short-term.

You can't just say oh well I don't feel like going this year.

They have to be, they have to feel that they're available, you know, to go, like not be wishy-washy.

Mmm.

Yeah, you've got to be… (UNCLEAR)… if they've got a lot of things going on in their life, well they've got to work out, you know, do I do this or do I not do this in, in the beginning.

Mmm hmm.

Not change in the right in the middle and say no, I'm not doing it anymore. 'Cause that, that could be very… your, you know, you particular survey could be quite relevant part of it, you know?

Mmm.

You know?

Would there be any feedback to the person that's in the trial from the medical, just like a, a catch up? How's it been going? Um, not, not the only contact being don't forget next Monday. Just a general… just a courtesy contact, just to say look, you know, I don't know how long, how… long between visits there is…

Mmm.

… whether it's 6 months, 2 months or but whether, you know, the person feels that they're the… you know, the people doing the trial are conscious of them at all times so that they're, you know, quite often they're just a contact to say how are you? Are things going ok?

A support.

Support, that's the word (laughs).

Yeah, so, um, a lot of the trials do sort of try and build that in.

Yeah.

Yeah, I think that'd be good.

This would be different than a lot of trials, wouldn't it? Because it's not like taking a drug or a placebo?

No.

You would know whether or not you had surgery. No one can hide that from you.

Yes, that's right.

So that already sets you apart from a lot of clinical trials and I don't know whether there could be psychological effects on that.

Well that's why I said, the contact, the support would be required, yeah.

With the overseas, the three sets over there, did they have a, a big percentage of people who balked at going into the no treatment? Did they say look, I'm not, I

just want to have treatment? Did they find it easy to get people?

Well that's a good question.

[LOTS OF YEAH's]

(Laughs)

And really the answer is, I don't know.

[LAUGHTER]

Um, they will, they will tell us in the end.

It's still going on.

Yeah.

But that could be a personality trait.

Mmm hmm.

That sort of person to look maybe a bit more adventurous than the woman next door who thinks, oh, no I'm just not going to take… I just want…

I think there'd be other reasons too. I think there'd be religious reasons. There'd be age reasons. You know, if you were getting really quite on you might say, oh, I'm not having surgery, I'm going to die soon anyway, or whatever, which people do. Um, I had a friend with, with lung cancer who said not having any more chemo, didn't work the first time, that's it, I'd rather die. And I think… but she was already getting into her late 70's so I think…

Yeah, that, that…

… people, people will have their reasons for not wanting to, to have surgery and it could be that they're in a more high risk group anyway, even if they don't appear to be… from what ever reason that they don't want to have the surgery.

Mmm hmm.

But I don't know. You'd have to… get the scientific…

And you don't know if, you know, they're in denial.

Where is those too.

Ok, so… if I, can I ask you to try and imagine yourselves kind of in this situation, if you've been diagnosed with low-risk DCIS and invited to be in a trial like this, how do you think you would feel about whether to join the research or not?

I'd probably want to know if I was in the really low, low, low risk.

[LAUGHTER]

Am I right down here, or am I up here? And that would, that would be the information I'd make the decision on.

Mmm hmm.

I think

Show me a picture.

Mmm.

Where is it?

[LOTS OF YEAH's]

Ok, so if you had, if you had a picture and you, and you felt like you had a really good idea of where it was and it was, it was low-risk, then…

Could the location in the breast affect the decision to have a lumpectomy or a mastectomy?

Yeah.

Because if I had low-risk and the alternative was going to be a mastectomy by the location, I'd be saying I'd prefer no treatment. Wouldn't want to loose a breast if I was in the low-risk group just because the location, not, not the extent, but the location was such that it was going to require a bigger operation.

Mmm hmm.

I mean, I don't know if that's a factor.

Mmm.

But if it is I would, um, I would take that into account.

Ok.

The treatment could be, um, is it always a mastectomy or is it just a lumpectomy?

It could be a lumpectomy.

[LOTS OF AGREEMENT]

I just know a few people who've had the lumpectomy and whatever and in the end they've had to have the mastectomy anyway.

My friend did.

Yeah, but they might have had invasive cancer though.

[LOTS OF MMM's]

Ok.

I think it's being provided with as much information as possible.

And what kind of information might you want other than…?

Well an honest approach to whether it's a, you know, you are genuinely in this area of the graph which, that we feel is low, low risk.

Mmm hmm.

Um, we feel you are a good candidate for the program because surely they're not going to be recommending… into that, into that program for no treatment if the medical profession has any… I mean if they're further up the scale you would, just wouldn't take the risk, would you?

Yeah, so if you were told you were a good candidate for this trial, where you'd be randomised to either treatment or monitoring…

That would be… it'd be… they would help you make an informed decision.

Decision, yeah.

So do you think you would say yes to the trial?

Um, again, depending on age…

Mmm hmm.

… it could determine… and if I was in generally good health otherwise.

Yeah.

Ok, so if you were in good health would that make you…

It might, yeah.

… want to go in the trial?

Yeah.

Ok.

I'd want to make sure that I, if I went into the trial I was going to be just followed or monitored, the support was really there. Because I think… you'd always be wondering, well I would anyway, oh, there's a change, oh, I've got a pain, oh, I wonder if anything's happening. Um, who can I, you know, who can I see or ring up or…

You'd want a mammogram every month.

Yeah, that's right.

[LAUGHTER]

And the radiation will kill you (laughs).

[LAUGHTER]

Exactly.

So there's a point here that only surgery or radiotherapy. Because I personally

would have surgery, but probably not radiotherapy. So you would need then more groups not just…

One or the other, yeah.

… yeah… (UNCLEAR).

But radiotherapy's not that… not that great either. My mother had, had it and…

Yeah, so what they'll be doing is…

… she's had other side effects from it.

… yeah, they'll be comparing, um, you know, the usual treatment group will encompass whatever specific, um, treatment options women are having. So some would have just surgery, some would have surgery and radiotherapy but it'll represent whatever's, um, typically happening, you know, in a group in that location.

But if it's…

And they'd compare that against…

… in a small area it's not quite as bad. See my mother had it in big areas and it raises a more (?). It's only a small area then it's probably not quite as bad.

Well if we're, if we're not in the situation, which we're not at the moment, um… it's very hard to know what to say because we don't know what we're going to be offered in 10 years time or 5 years time or whenever it might happen. And the, the treatments might be, as you say, radiotherapy, so we've discovered that you can get away without having surgery but, but you would need radiotherapy and maybe you would prefer surgery to radiotherapy.

And maybe in 10 years time they might have pills. Take a course of tablets.

Well…

You know? You know?

Ok.

But might not want them either depending on the side effects (laughs).

Yeah, were you about to say something there?

No.

Ok?

No, I was just thinking it's really hard to answer that right here, right now.

[LOTS OF YEAH's]

And I'll, you know, definitely go away and research and maybe Google and see, you know, how I was feeling about… I don't know. It's… and we're all different.

Mmm.

We're all different.

And they've got the support groups to give advice to women which sites they should look at because lots of, lot of sites on, on the Internet are, are misleading.

[LOTS OF YEAH's]

So you have to know where to…

Yes.

… look.

Yes, that's true too. Mmm.

That's why doctors hate Dr. Google.

No, not necessarily. If it is true, it does the job for me…

[TALKING TOGETHER]

… to go home in their own environment and read through all that information. But as a GP you would be saying look, if you're going to look at it these are the sites to go to. Don't go to…

Yes, exactly. Exactly.

So yeah, I mean I think… I appreciate I'm asking you a difficult question to kind of put you on the spot in a hypothetical. Is anyone feeling…

It's being informed. I think that's the key.

Yeah.

Feeling that you're informed, that you're going to be supported, that if there's problem that you have a first port of call.

Yes.

Um, whether they say well just, you know, initially go back to your GP but otherwise no, come into us and we'll just have a little check. Er, I think that's, if you feel more inclusive, the whole thing's inclusive of you, I think that, that… I think we wouldn't, I think I wouldn't make the decision if I didn't feel that.

Mmm hmm. Ok, so just to check that I'm understanding, if you feel like, if you felt like you would be well supported as a participant in the trial and you would know who to go to with questions…

Yes, that's right.

Yes, yes.

… then you…

And my general good health, my general health was good.

Mmm hmm.

And all strict check-ups. You know, you've got to be really diligent with that an d have the support. 'Cause it's a mental thing too, isn't it?

Well it is, very much.

That's right…

You have to be positive. You have to have a…

Positive thinking, mmm.

I'm a great believer that negativity is not…

That it's going to work (laughs).

… (UNCLEAR)…

Yes.

Ok, does anyone feeling… no, there's no way I would go into a trial like that?

(UNCLEAR)

You always have the option of dropping out.

Yeah.

Always.

Ok.

And I think that's something that at the beginning, if they're really, really clued in to everything that's happening, as to this is where you fit and this is why we would like you to consider this, but there's still the option of dropping out.

And their personal circumstances may change. You know, if you're talking about a 10 year trial, you don't know what's going to happen in the meantime.

Mmm.

I suppose that's with all trials, I guess?

And of course the other thing is in Australia the distance, I mean, you can do a trial in Sydney or in Melbourne, but if they are somewhere in nowhere you can't (?) people (?).

Mmm hmm. So if people were in more remote locations?

Yes.

Do you think, um…

To driver to Melbourne for the day at the University to do it, that sometimes can

be hard. If the husband dies and got no one to drive them, that does cause problems. I've read articles about that.

Mmm hmm. So do you think women in that situation might be more likely to… say… I just want to have treatment now and not…

They might.

Possibly.

In country areas I think there's a consideration because it can be so difficult to get to…

And expensive.

… yeah, and expensive. Difficult to get to for regular check-ups. I mean, I'm lucky, I can catch a bus into town.

[A FEW MMM's]

No problem for me. But if I lived a 4 hour drive from the nearest town, let alone nearest hospital, that's…

Mmm.

… that, you know, could be a determining factor.

Mmm, yeah, right, people in remote areas.

But then they probably, possibly wouldn't be part of the… trial anyway.

Well that, that's what I'm saying.

Yeah.

They probably would say no to it. Possibly because of the remote…

And so other than that, um, issue of, er, distance, when you kind of raised the idea of well how many women just balk at the idea of a trial and…

Yeah.

… so if, if, um, the researchers were finding that women were, you know,

were just refusing to go into a trial, what is it that, that makes you all feel more comfortable with the idea? What sort of, what… what have I said that sort of convinces you that it's… could be a worthwhile thing to do?

Well we all want to get an outcome and it's going to only be done through statistics and looking into individual people. And I think you always want to have, you know, human race have a better life. And living in Strathfield I could quite easily get to a hospital, you know, to have tests done on me.

As I say, I think if you were genuinely presented with the case that you were very low risk, I think the idea further down the track that there's some other areas that… er… there's a wait and see. I've got a friend who's on a wait and see at the moment, um, lymphoma, and, um, and that… they don't know, they've just taken initial tests but it could, the, the doctors are saying it could be something we, we better leave you alone for a while and see what happens. Or it could be very aggressive and obviously we have to do something. So I think if people can feel confident that there is a, a period where you don't have to rush into something, um… then that, that could be good for them and their circumstances.

One of the things that sets us apart is that you have given us information today that most people don't have.

They don't.

Which points to education being one of the big factors…

[LOTS OF MMM's]

… that is going to be required to get people to participate in these trials.

[A FEW MMM's]

I agree.

So presumably a woman goes for a mammogram, DCIS is picked up, they do grading, ok, so who, who then comes on board and says to the woman you're very low grade, you… we would like you to consider being part of a trial… who comes on board then? Is that her specialist or is it a University program, or…?

Yeah, well…

Who proposes that question to the woman?

It's a team of people involved…

Oh.

… in determining whether she's eligible. And in terms of exactly who actually talks to her about it, um, it could vary. It could be, um, that the surgeon does or something there's like a research nurse or a, a research assistant who…

Well that's where the information's important.

Yep.

'Cause you, you've got the shock of being diagnosed with something and then somebody else is proposing a secondary question, decision; surgery or go… go for no treatment.

Mmm.

So there's a, I think that's very important, as you say, information is just absolutely important. And the person has to feel that it's just, yeah, they've been given as much information as possible.

And unbiased. Unbiased.

Unbiased.

So you're not being pushed into a trial because they need a certain number of people to do it.

Mmm hmm.

Yeah, I think who talks to, I think you're right, is going to be very important in this. And, you know, you know from talking to us in the beginning how few of us had even heard of DCIS. So it's not going to be any different with the general population. Could be worse.

People just think breast cancer.

Mmm. Yeah.

That's what I said before about getting public information out.

Well I had no idea.

No.

And so who, who would you want it to be talking to you about it?

Well isn't that the government?

The government, shouldn't the government be doing that?

No, you mean talking about the initial…

Oh, you mean about going into the…

Yeah.

… when you go the, oh, ok.

Talking to your local doctor and then he might know about a trial and says I suggest you do it.

The doctors do know about the trials.

Yeah.

They do yeah. Lots of times they do.

Well I haven't had breast cancer so what is the procedure now?

Usually, usually if, if, if a cancer is picked up, er, by the screening process then the people who are doing the screening process call the patient back for another one and they recommend biopsy, and if it comes back positive they, they, they are referred to a team. And they, somebody in the team, because it's a surgeon, oncologist, radiotherapist, someone in the team may raise the (question?). So it's not usually one person…

No, no.

… who decides that… (UNCLEAR)… but it's a team.

But I suppose it's, it's… then that person, who ever it is, has all the information so that when they go into that discussion with that patient they're, they are confident that they are given them as much information as possible.

Ok, and so having been given that information, um… what would be, what would you do to help decide which way to go? Are there other people you would be talking to or other, um…

Well, it would be your family.

Yep.

In my case I'd have to get on the phone and ring my sister in Perth. You know, I'm not going to have a lot of support in that area. But I mean most people are married and got brothers and sisters. But certainly it would be your family.

[A FEW MMM's]

Ok, and some of you mentioned before that, um, like your health might come into it. So it was, you know, if you felt like you were in good health you would be comfortable going in a trial.

Possibly, yeah.

So what about if you, if you were not in such good health?

And also the age.

And yeah, if you were older, would that make you more like or less likely to go in a trial?

I would be less likely. If you're not really very well you're dragging yourself off to somewhere you don't want to be when you're not well.

Mmm hmm.

And you think, oh…

I think that might be something you have to look at in your trial.

Yeah.

You might want to say I'm only going to do women between 50 and 60…

Mmm hmm.

… compared to women between 50 and 75.

And it's at a hospital within half an hour on a bus. Or, you know…

Or we're only going to look at city living women.

Yep.

Do you know the age group in those trials overseas? Was there an age group?

Yeah, so it's, um, they're… the lower cut off is either 40 or 45 or 46, but there's no upper limit.

Oh, ok.

So they can potentially include quite old…

So when you are saying would you part of a trial, are you saying would you take a risk of not having treatment? So that is what you are saying?

Mmm.

Yeah, so basically…

So that is the question, would you take that… going in the trial means taking the risk of not having therapy.

But knowing that if something happens…

Yes.

… in the meantime, you'll be, it'll be acted on.

Yes. It was basically if you're not in the trial but if you have surgery, then the follow-up is the same. You still have yearly mammography.

[A FEW YEAH's]

So, er, in those groups plus if you're not a trial you just do the same thing.

[A FEW MMM’s]

I would want more than that though.

Mmm hmm.

I’d want to know that I’ll be able to ring them if I’m worried, or go in and see them. Which would be more than if I had a… had surgery or something, ‘cause that’s over. You know what I mean? Like…

No, you are attached to similar to the thing for many years until they hand you over to the GP.

But I, I think that not having anything… makes me a bit scary. But I’d try it…

Yeah, but…

… but I’d want to know that I had lots of support.

Yeah, I think…

Yeah.

… in most cases you would have basically, I think. And maybe who, who doesn’t have a treatment would be more support.

More support, that’s right.

Er, yeah, well what’s that on earlier about, um, was it radiotherapy and they had it again and it could effect the heart later on?

Mmm.

So with me, because I have a heart condition, it might dissuade me not to have the treatment ‘cause of that, rather than having radiotherapy because of the heart.

Ok.

So I think health for me definitely would be a factor…

And if you did have a condition, surely you would… expect to be allowed to speak to your other specialists and see, and see what they think.

Oh, I mean a lot of, lot of specialists perhaps.

Yeah.

Mmm. Yeah.

Yeah, ok. Um… ok, and you also mentioned that, you know, people's personality could, could come into it. Do you have people in your lives who come to mind who you just know would think totally differently to you? Would…

Oh yeah.

(Laughs)

… tell me about how they would respond if they were here?

Oh, people who might be skeptical. Um…

Negative thinking all the time.

Negative thinking people.

Think of the worst all the time.

[A FEW YEAH's]

The just, they just want me as a guinea pig.

Well some people do think that.

Yeah, it's true.

[LOTS OF MMM's and LAUGHTER]

So yeah, I think the person's, the person's, the person's personality. but that might be determined by hem saying I just want whatever treatment is necessary. So they've already made that decision.

Or the alternative. Or I'm just, I've had enough, I'm not going to do it.

Yes.

You, you'd get the extremes with them.

But if you'd had enough and you weren't going to do anything…

Well then, yes, you would.

… you'd do treatment, er, no treatment.

Well, you wouldn't get the choice again, if it's randomised.

Oh, if it's randomised, yeah, yeah.

And, and that could be an issue too, that people who, who don't want to have it… and there could be more than you expect I… I don't know. Um, but if, if you really don't want to have treatment and if you go in the trial, there's a 50% chance that you're going to get it. You might refuse to go into it.

And have the treatment.

Well you, you might refuse to go into the, the…

The random…

… the randomised trial.

Yes, of course.

And all right, you can pull out at any time, but then that's going to skew the numbers then. Why would you bother anyway? Just say no, I'm just not going to do it. And that sounds weird but a girl I went to school with in her 30's refused cancer treatment, died, left two little kids.

[A FEW MMM's]

People do, they just refuse.

[A FEW MMM's]

They don't want to go through for whatever reasons.

Mmm.

Mmm.

Human nature would have to come into it.

Well that's the personality again, isn't it?

But here we are not talking about chemotherapy. Often think about cancer treatment as chemotherapy when they loose their hair and really can feel unwell. This one is, is not.

It's not.

It's not chemo, it's radiotherapy.

Ok. Um, well thank you for all that discussion. That, that's really interesting. Um, so just before we wrap up the session I would like to ask you to fill in that second questionnaire, questionnaire number two. So before we do that is there anything else you want to say that you haven't had a chance to… express yet, about this?

No? Ok, so just once you have done that questionnaire number two you'll be done. So when you're done, um, Brooke will collect those up and she'll give you a gift card as a thanks for coming along and she'll just ask you to sign a page to say you've received that. So thanks very much for coming.

Thank you.

[LOTS OF THANK YOU's]

[TRANSCRIPT ENDS 01:37:23]

END

Interview: DCIS FG5 2018.05.25

Interviewer: **BOLD TEXT**
Interviewee: PLAIN TEXT
Pauses: …
Unclear words: (?)

[TRANSCRIPT BEGINS 00:17:12]

So as a number of you have said, that DCIS is quite an unfamiliar thing right now, um, I'm going to start by talking about something that probably is at least a little bit, um, more familiar, and is relevant to DCIS, which is breast cancer screening.

So what is breast cancer screening? Well in Australia we have a national program that provides screening, free of charge to women, to look for early signs of breast cancer.

Now it's important to understand that when we talk about screening it's something that's designed for women of a certain age in the general population who are well. So it's not for women who have a breast symptom (like a lump or pain or discharge) that needs investigation – that's a separate process.

The aim of screening is to find women who have cancer early, so that they can be offered treatment early, in the hope that earlier treatment will lead to a better outcome. And the main goal of screening is to reduce the number of women who die of breast cancer.

So the best available method for breast cancer screening is a procedure called a mammogram, which uses x-rays to make images of the breasts. And the whole idea is that these x-ray images can show up cancers or abnormalities that may be so small they can't be felt as a lump.

So if the mammogram images look abnormal or suspicious in some way, the woman gets called back to have some extra tests which may include a biopsy. Biopsy means using needle to take a small sample of cells from the breast to look at under a microscope, for the purpose of confirming a diagnosis. So from looking at what that small sample of cells under the microscope, the pathologist will either say actually everything's ok, it was a false alarm, or they'll diagnose breast cancer or DCIS.

DCIS or ductal carcinoma in situ is a condition that can affect the cells inside the breast.

Typically, DCIS doesn't cause any symptoms like a lump you can feel, so it's generally found through screening. So before we started doing screening in the population, it was hardly ever diagnosed. So in the past, women may have had DCIS but because it didn't cause them any symptoms and because there was no screening, they wouldn't have known about it. Whereas now, nowadays we have widespread screening in the population and so DCIS has been diagnosed a lot more commonly than it used to be.

Can I just ask, is that, um, when you say DCIS does it show up as a shadow or does it show up as a, er, a lump of cells or does it show as, um, a different sort of, er, in the, in the, er, x-ray thing?

Yeah, it typically shows up as, um, what they call calcifications, so little white specks.

Yeah.

Um, that's how it typically looks.

Ok.

But part of the challenge with this condition is it's almost like DCIS is a new disease that we've kind of only really discovered through introducing population screening programs, in Australia and other countries, and that's happened from about the 1990s. So everything that women are told when they're diagnosed with DCIS, and everything that I'm telling you today, is the best information we can give based on current scientific knowledge about DCIS. But it's important to understand that that knowledge is still kind of limited. So there's still quite a bit that we don't really fully understand yet about DCIS.

Now I'm going to show you a diagram with some numbers now – don't worry too much about the numbers, it's just to give you, um, an idea of how many women are affected by DCIS.

So if you imagine that each one of these dots represents one woman. If we

take 1000 women in Australian who have breast screening for a period of 25 years, in total about 81 of them will receive a diagnosis of one kind or another, as a direct result of the screening mammogram and the further investigations that follow.

And if we look in more detail at these 81 women, we find that about 16 of them have DCIS and the other 65 have what's called invasive breast cancer…

Excuse me.

… and I'm going to explain the difference in a minute.

Oh.

So this is just to illustrate the women with DCIS are a smaller group – so they're about 1/5 of the total number of women who are diagnosed through screening.

So let's just talk about what is DCIS is in a bit more detail, breaking it down word by word. So the word ductal means it relates to a duct or tube in the breast that carries milk to the nipple. Carcinoma means the cells show abnormal features similar to cancer.

And 'in situ' means contained in the original place. The 'in situ' part is very important to defining DCIS. So this illustration shows how DCIS is different from 'invasive' breast cancer, which is really just a more precise term for what we commonly understand breast cancer to be. So based on what they can see looking at the cells from the biopsy the pathologist will diagnose that it's either DCIS, which means the abnormal cells appear to be fully contained within the duct, or invasive breast cancer, which means those cells or some of those cells have moved outside the duct into the breast tissue around the duct.

So DCIS and invasive breast cancer are different but they're related. In DCIS, the abnormal cells are contained within the milk duct and have not spread outside the ducts to the other breast tissue. So as long as those cells remain contained like that, DCIS is not a life-threatening condition at all. But DCIS might develop into invasive breast cancer later on, and if that

happens the time frame for it to happen is really variable, so it could take many years or it could happen much more quickly.

So in invasive breast cancer in means some of the abnormal cells which were previously just inside the ducts have spread into the breast tissue around the ducts. So it still can be entirely within the breast, it doesn't, hasn't necessarily spread anywhere else in the body. It's just the fact that it's gone outside the ducts that, that makes it invasive breast cancer. But once that's happened it means the cancer could then spread to other parts of the body, and that's when you really have a problem.

That's when invasive breast cancer has the potential to be a life-threatening disease, but it's good to remember that these days the majority of women diagnosed with breast cancer will be treated successfully and will survive their cancer.

Now these, um, this slide shows some different phrases that are used to describe DCIS. So none of these are wrong but you can imagine it can be a bit confusing for women because DCIS is described both as a type of cancer and as something that's not quite cancer.

So even if you haven't heard the term DCIS before, you might have come across some of these other terms like Stage Zero breast cancer or non-invasive breast cancer or pre-invasive breast cancer.

Excuse me, does (swollen?) tissue cause breast, er, cancer?

Sorry, what was that?

Swollen tissue in the breast.

Swollen tissue?

Yeah.

Um… not, so it's, it's a change that's, um, at the level of the…

Hormones?

… little cells inside the, the ducts or the lobes of the breast.

Oh.

And yeah, it can be to do with hormones, um… er… yeah, but it's not, um, you know, there are other symptoms that you can get in your breast. Swelling, or like cysts or sometimes lumps that are benign, that are not necessarily related to this.

No, but it'd still be worthwhile getting them checked (laughs).

Yeah, absolutely.

If I had swelling I'd do something about it.

Yeah. Ok, so, um, what happens after DCIS is diagnosed? Well, as I've said, DCIS itself doesn't affect a woman's health, but it has the potential to turn into invasive breast cancer. Now we have reason to believe that this progression to invasive breast cancer might not happen for everyone, and I'll talk more a bit later about why I say that. But the bottom line is that right now we can't reliably predict which of the women with DCIS will go on to develop invasive cancer or how long it may take before that happens. And that's why virtually every woman diagnosed with DCIS is given treatment, to try and prevent that progression from ever happening. And I'll talk shortly in a more detail about what treatment involves.

But firstly, I just wanted to come back to, um, asking you all again if you've ever heard of DCIS before, now that I've explained a bit more about it?

Never.

Not ever. Not in those four letters.

[A FEW NO's]

Yeah, not in that term.

No.

I've heard of people who've had, er, some kind of cancer that's treatable, it's not… cancerous or they, they… but that's as far as it goes. But not the actual term. I've never heard of it.

[A FEW NO's]

Yeah, I've never heard of the term before.

Yeah.

My sister had difficulty breast-feeding. Her daughter's now 31, um, and just about when her daughter was about 18 months old she had a lump in her breast which ended up being like a cyst in the duct. Um, they investigated it, as I said, you know, 30 odd years ago, well 29 years ago, and then nothing was ever… but the term, that term was never bandied around.

Mmm hmm. Ok. Um, and do you feel you understand what I've said so far?

[LOTS OF MMM's and YEAH's]

Is there anything unclear or confusing?

No.

No. Can I ask just something, just ordinary cysts for example, is that a part of… would they need to be investigated if they're outside the ducts?

So, um, I mean any…

Is there calcification cysts?

… any symptoms that you notice should be investigated, but if they determine that it's a benign cyst it, it means that it's basically like a little lump of, of fluid, like water essentially and it's not going to cause any problems. So with various investigations they can determine that. Um…

And they would mention in their diagnosis if there is an investigation needed, would they?

Yeah, so if, if you… receive a diagnosis of breast cancer or of DCIS that suggests that its, er, something that does need treatment…

And when did they start diagnosing these, um, the DCIS?

Well basically when they started doing, um, population based

mammography screening. So the, um, program in Australia started around 1990 and…

'Cause I've never seen written anywhere this DCIS.

Yeah.

So I wonder if it was a new thing.

(UNCLEAR)… this name, but, er, breast cancer I heard it for a long time. And that my niece, she's about in her 40's, she took her breast couple of years ago. But what happened to her, she has 3 boys, the youngest she didn't breast feed and when she had breast feed it's very painful for her and too much, er, you know, must be infection and then it grew something, that must be cause of breast cancer. But no one in the family thank god has this problem. Only she is. I don't know, must be through her feeding the baby or infection in the nipple. I don't know what caused the breast cancer.

Yeah, most of the time we don't really know what causes it. So, um…

So presumably when they started doing, keeping the numbers of women who were being screened they noticed this anomaly of the women that had the, the, um, the duct…

Cancer.

… dot, rather than anywhere else so I'm assuming it developed it's own special name as a result of an anomaly picked up in, in the mass screening?

Yeah, so basically it is something that we've only really, we've only known it's there because we now have this imaging technique that can show up these…

Yeah.

… you know, these calcifications.

You've now got 20 years of data.

Yeah, which makes a difference.

Yeah.

Which you can look back on.

Yeah.

So does ultrasound show up the, the, um, DCIS the way the mammogram does?

Yeah, it, it also does show it up.

I wonder why we don't go to ultrasound as apposed to mammographs?

Yeah, well, um, basically the…

In the screening, I mean.

… the research study evidence shows that for the general population mammograms are the best tool, are the most accurate tool for screening.

I cynically would say if it was testicular cancer they'd be doing something very different than squishing it.

[LAUGHTER]

Definitely.

There wouldn't be an expense…

[TALKING TOGETHER]

Sorry. No, I mean it's just I know many women who will not have a bar of mammography because they don't want to…

It's true and also but if you're a young woman, like my sister was only 30, then the breast is too dense. I mean certainly I notice now that it's not dense at all, it's just sort of flopping around there.

[LAUGHTER]

And it would be much easier to, um, to squish it. But I, I just think, you know, that ultrasounds have been around for quite a long time. I don't know why they haven't swapped over. I think it's expense and I cynically say exactly what I said.

If it was testicular cancer they would have been ultra-sounded.

Yeah, they would (laughs).

But to me I've done it two, two times the mammogram. First time it's bit painful. Second time I couldn't… let her do my second side.

Yeah.

It's very, very painful.

Mmm.

But, er, but last year they sent me another one to go. I didn't go.

No.

[LOTS OF MMM's]

I didn't (?) because as I say whatever happens to me, you know, when God wills, happens, happens. But… you could still, from the machine they put you… if…

It, it is quite invasive.

… the machine damaging you.

Mmm, well I found the last time I had a mammogram, which was probably 15/18 months ago, I think they had a different machine, and you weren't, you weren't as pancaked…

Yeah.

… as, as previous years. 'Cause I've been having them for about 20 years.

Yeah.

And, yes, it's quite a shock the first time you go.

Oh yes.

And you are really flattened. But, and I commented to the operator last time, but this didn't seem to have quite the same pressure, and she said no, different machine.

Different machine, yeah.

I, I agree. I only went a few weeks ago for my latest one…

Yeah, some sort of advancement.

… and it was, it was so quick and it was just over and done with.

[LAUGHTER AND TALKING TOGETHER]

It's not, it's not a comfortable experience…

And then they say to you, don't breathe and, and when you're in pain they tell you to breathe, to forget about… they say don't breathe and I'm like…

[LAUGHTER]

… and your eye-balls are popping out (laughs).

[LOTS OF YEAH's]

That's why they say you can focus on, on not breathing (laughs), supposed to forget the pain.

[TALKING TOGETHER]

But for a few minutes it's an uncomfortable feeling, it's well worth it whatever they're going to find.

It is. I've, I've always gone to them.

Yeah.

But they've not been enjoyable, no (laughs).

Yeah, but you wouldn't, you wouldn't choose to do it as a pass time but…

… (UNCLEAR)… enjoyable.

Bu, yeah, lots of other things are worse so…

[LOTS OF AGREEMENT]

Oh, well I've had a few surgeries and I've been through…

Yeah. Let's not go there (laughs).

… don't go there because…

No, it's not a pleasant experience no…

… I've had worse (laughs).

So if, if you go for a mammogram now and they call you back, is it now you have DCIS or you have invasive? Is, is… are they the two terms? Is there no, um… if you have DCIS you have a certain… path to go down? Or if you've got invasive you go down a different path? Or is it, is everyone now just diagnosed with DCIS and then they go… um…?

Um, no, so after the, the mammogram and then the additional investigations like biopsy, each woman will get a diagnosis that could be either invasive breast cancer or DCIS. Um, and there's a, there's a few other things that can be diagnosed as well that are a bit less common, or, um, you know, or they'll be told they're actually… that everything's fine. And then after that I'm going to tell you a bit more about what, you know, what the pathway is.

Ok.

Yeah.

Could I just, er, make a comment, not my last mammogram, the one before, I got called back to go to Westmead and they said you could be here for hours or could be here for… and they retested and they did extra testing. Then they said to me, oh, it was nothing. It was some fluid or something. Nothing that they said to me should be concerned about. I wonder if it was this that they were talking about.

No, I don't think so, because if it's DCIS then…

Then they have to do some kind of treatment?

Yeah.

Ok, yeah, 'cause I, I actually was called in and I was there for a while doing all this

sort of testing…

Yeah.

… and then they said to me it's all clear. And it was something else.

Yeah. So that, that…

So that could… so that could be anything, couldn't it (laughs)?

Yeah, so that was something that they consider benign and not dangerous at all.

Ok.

I'm sorry, can I just clarify, I, I assume from the information you've given us before that the mammogram was enough to diagnose the DCIS. Do they, do they, um, then send you for a biopsy before they'll actually say it's DCIS?

Yes.

Right, ok, thank you.

So they never diagnose anything just from a mammogram.

Ok, thank you.

The mammogram will only show, um, everything looks normal or something's a bit abnormal and we need to do, um, extra tests.

Mmm.

Is it a clue that they need to, to look for…

To investigate.

Exactly. So those extra tests that you might have had, you know, sometimes they…

Yeah, that's probably what it was, yeah.

… (UNCLEAR)… having another mammogram, having ultrasound, having a clinical breast examination with the doctor and the biopsy is often, er… the

key to confirming a diagnosis or, or not.

Yeah, ok, thanks. Are the cells different in DCIS than they are in invasive breast cancer?

Well it's…

Is it a different type of cell?

Well it's the difference between whether the abnormality is, whether those cells are only inside the duct or whether those abnormal cells are outside the duct.

Ok.

So let's talk a bit about, um, about what happens if you are diagnosed with DCIS. Um, as I said before, you will be recommended to have treatment. And the goal of giving you treatment for DCIS is to prevent the abnormal cells from turning into invasive breast cancer. And overall it seems that our current treatments are doing a pretty good job of achieving this goal.

So surgery is really the primary form of treatment for DCIS. So that's doing an operation on the breast, in hospital, under general anaesthetic. And doing surgery also means the pathologists get another chance to look at these cells under the microscope, but this time they've got more to work with because they've got hopefully the entire area that's affected by DCIS rather than just the small bit that was taken for the biopsy before.

Excuse me, do they surgically remove the breast, or just the milk duct?

So I'm going to tell you in a…

Oh.

… in a moment about, um… yeah, what can happen. But…

(Laughs)

… whatever they have removed they'll, they'll then look at it, um, again under the microscope and, um, in most cases it will confirm the original diagnosis, DCIS. Um, but in some cases they find after surgery that the

patient actually has invasive breast cancer, um, that the biopsy didn't catch. So that patient's diagnosis is changed or upgraded at that point.

Now there are different types of surgery a patient can have, which I'll explain in a minute and surgery may also be combined with another form of treatment. And exactly what treatment each patient has will depend on features of her DCIS (like its size and something called grade – which I'll talk a bit more about later) as well as characteristics of the woman herself (like her age, her overall health, and her preferences).

So I'm going to tell you in a bit more detail about the 2 most common approached for treating DCIS. And both of these are considered very effective and survival rates for women with DCIS are excellent.

So treatment for DCIS most commonly involves breast conserving surgery and then radiotherapy. So this means having an operation to remove the part of the breast that's affected by DCIS and a small area of healthy breast tissue around it. And this may also be called a lumpectomy or wide local excision.

And after breast conserving surgery, as I said, a pathologist will look at the breast tissue that was removed. And sometimes, if they find there's a bit more disease than they originally thought, then the woman may need more surgery.

Now everyone responds differently to surgery. Some side effects happen to most people, some happen only occasionally. And some side effects happen straight after surgery, and others may take longer to develop. But some of the common side effects include things like pain, numbness, and bruising around the wound.

And after this surgery, the patient will typically have radiotherapy, and that's normally every day for a few weeks. Radiotherapy uses x-rays to destroy any abnormal cells that may be left behind in the breast.

Now receiving radiotherapy is painless. But women may experience some side effects. So during the radiotherapy treatment, the skin of the breast..

Oh my God.

… may become red and dry, a bit like sunburn…

That would freak you out slightly, wouldn't it? (Laughs)

… or the skin can become darker; and women often feel more tired than usual. And these side effects usually settle down after treatment finishes and things get back to normal.

Now during breast radiotherapy, it's possible that a small dose of radiation may reach the heart or lungs, depending exactly where the DCIS is. And some studies have suggested that women who had breast radiotherapy in the past may have a slightly increased chance of having a heart problem, like a heart attack, later on in life. So doctors these days are using more modern techniques when giving radiotherapy to try and minimise this problem as much as they can.

Now instead of having breast conserving surgery plus radiotherapy, sometimes a mastectomy may be recommended, for example if the area of DCIS is quite large compared to the total size of the breast or for some other reason. So this is a bigger operation which means there's a higher chance of complications and a longer recovery time.

So all the side effects I mentioned for breast conserving surgery are also relevant for mastectomy but because the surgery is more extensive, there may be additional side effects such as stiffness in the arm or shoulder.

And obviously mastectomy affects the body shape and it can affect women's body image or how they feel about their body. And many women have the possibility to consider breast reconstruction surgery to try and help with that.

Now just in case you're wondering at this point I want to mention that chemotherapy is not a treatment that's used in DCIS. So the main treatments are, as I've outlined: those different types of surgery and radiotherapy.

So what are your thoughts and feelings about that information about treatment for DCIS?

I suppose the first thing that comes to mind is that if DCIS, there was only a certain number of the women that went on to develop invasive breast cancer, there would be a real need to toss up and say, well do I just leave it and hope for the best, or do I just…

I was just thinking the same way, yeah.

… go ahead with something else, yeah. You sort of think, oh, will I bother? Not bother, that's the wrong term.

Yeah.

But, you know, well given that I've got a hmm hmm chance of, of it just staying here and it not developing into anything, would I actually do anything right now?

But treatment for DCIS is only carried out once it has left the duct and has entered the breast…

But DCIS doesn't leave the duct.

No, it doesn't leave the duct. It's still inside the duct.

It doesn't leave the ducts.

Still inside the ducts.

That's why I'm thinking, is surgery, is it really necessary?

Mmm.

Ok, so just to clarify…

Yes.

… they will treat DCIS, um, when it's still inside the duct.

Right, Ok.

Yeah.

That's the typical, er…

Because once it's moved outside the duct it stops being DCIS, doesn't it then

become invasive cancer.

Invasive breast cancer, yes.

[A FEW YEAH's]

But it would be pretty rare that they'd remove an entire breast because of DCIS though, wouldn't it? Isn't that more an invasive cancer?

Well, um, not necessarily. So, um… it is… it's more common to have the smaller surgery, lumpectomy, but mastectomy is actually more common than you might think and it, it could be that the DCIS is in lots of ducts throughout the breast.

Yeah.

So it's still DCIS.

Mmm.

Yeah.

It's not invasive, but it might be affecting quite a large area of the breast.

Yeah.

Oh.

And depending, you know, particularly for women who have smaller breasts, you know, if there's, if it's hard to remove all the DCIS without, er, you know, and, and have a good cosmetic outcome they might suggest just removing the whole breast.

But, but that's, but they're working on the basis that it will be invasive.

That it might develop. Yes, so that could possibly develop into… yeah.

That's, that's what I'm thinking. I mean… so it may or may not, but isn't there, er, more tests then, before a decision is made?

I'm with you. I'd want… regular screening please and if it moved to something else…

I, I'd want more testing. I would not want surgery because, oh, you've got DCIS that may or may not... it'd be life-threatening, but isn't there other treatments? Other, um, some other things they can do to, to make sure that it is, that it will become invasive? That's, that's…

Could they do radiation therapy in situ? Like, couldn't they just use some focused radiation…

Yeah. That's right. Like they do on the kidneys with stones and that. That's what I'm thinking.

… yeah. Sorry.

I suggest, you know, when just disease hit the things…

Laser? Something like laser?

… better to remove everythings because whatever they took it's more, too much more work for the doctors, more for the hospital, more for the things and later on they going to appear… or sometimes persons lucky if sometime you have little wound and you body, you quickly recover it. Some other people took more infection, more infection, more infection until they get it again, you know? Each persons different. Each persons different have, why they have operations. Some people very quick recover, some people… they won't. So… with Gods help, I don't know, each persons different.

And they're the sorts of thing people always need to decide to…

Well that's right.

… to consider before surgery.

That should be a decision.

Yes, because people are all individuals and they all have different, different healing paths, different ways of coping. Some people you know…

Yeah, that's right.

… can cry at the…

That's why with the people, you know, better to try little bit. The persons be able to recover quick. It's helped the little treatment. But if their body not taken the things better to remove the whole, er, infections.

Mmm. Ok, so a couple of you have said, um, you might feel, er, quite reluctant to undergo surgery for…

Well I wouldn't jump right into it. I would not take that… or say, look, I recently had surgery a few months ago. It took me a year and a half to make the decision because they say it may or may not be, until I went to a few doctors. Now some doctors I went to Norwest, that I'll never recommend to anyone…

(Laughs) I know what you're saying.

… believe me, um, there were some other doctors who were not bad, but then I ended up with a particular doctor who actually explained it to me, who actually went in detail and said, look, these are the options and it's your decision. Not this particular doctor said to me like you've got this, you've got to do surgery straightaway. And I, you know, she kept on ringing me and ringing me to say, aren't you coming? I said to her if you don't stop bothering me I'm going to call the registration board about you.

Mmm.

And this is the sort of thing, when they say straight into surgery from my experience I'll be reluctant.

[A FEW YES's]

Because there are doctors who will push, you know? Oh, you've got this, you go straight in.

Yeah, yeah.

It's important.

Yes.

Whereas other doctors will actually say to you, well this is the disease, we can do further tests, we can do this, we can do… and they make you feel more comfortable

about it and that's why, again, I will have second and third, and maybe fifth thought about doing this sort of thing, from my experience with the…

Yeah.

… medical practitioners.

Ok, what about other people that haven't said much.

Well I think I'd jump the complete other way to that.

(LAUGHS)

If someone told me I had anything like that I'd be there in a flash.

Yeah, yeah, yeah.

Do it tomorrow.

Yeah, I'm just talking from what I've been through.

I've, I've put up other surgeries and gone through a couple of years of torture with trying all these other non-invasive things.

Yeah, yeah.

And I look back and I go, why didn't I just go back and have the operation on the first day and get that couple of years of my life back.

Mmm.

So if it was me and they said I had that and I needed to have it, whatever it was, remove, I'd be there tomorrow.

And I mean that's the difference isn't it?

Mmm.

Everything is such very much a personal…

Based on experience.

… thing and based on experience.

Yep.

And, you know, if one of those alternative therapies had have worked for you, you would probably be saying fabulous… (UNCLEAR)…

Thank god I did it.

… yeah, exactly.

That's right.

So we're all, we're all, um, the summation of our many experiences.

Yeah. That's how I see it.

But certainly the medical person with whom you interact that gives you the first information can make or break where you go from there.

Well it did, 'cause I was very cynical for about a few months. I wasn't approachable at all.

No, no.

She really made my life miserable.

(UNCLEAR)… 'cause you're denying that, that medical professional, yeah.

Yeah, she actually made my life miserable.

I think I agree with (Rayleen?) that if… 'cause I've had a couple of surgeries that they've said, you know, this is a problem and you need surgery, and I've done it. Um… yeah, if somebody said to me, you know, these are the test results and you've got this breast issue… I think I'd say well talk to me about the alternatives, talk to me about the procedure and I'd make my mind up within a very short period of time.

Yeah.

And you think you'd… which way would you go? I mean…

Oh, if they said surgery I would. And I… I think back to the last women that I worked with who was diagnosed with breast cancer, she went to the doctor on the

Tuesday and she came into work on the Wednesday and she just said to the boss, it's breast cancer and I'm finishing up today…

Yep.

… and I'm on leave, and… I'll be back…

Whenever.

… whenever, and she actually had the same care team that woman who, um, had been diagnosed 18 months before, had. But she just came in and went, bang.

Yeah.

That's it.

I worked with a woman, I worked with a woman who did exactly the same thing.

Yep.

And she's fine now. And, and one of the things that, that the whole thing with breast cancer that they've always pushed is the earlier you do something, find it and do something about it, your better chances of survival. And, and that would be enough for me to say, if you've got anything just go and deal with it.

But then it's such an… I mean look at the ad that just started on television now where the women are, you know, my sister has dark hair, I have blond hair?

[A FEW YEAH's]

And you know there was that thing about the gene for breast cancer in high profile people like, oh what's her face…

Oh yeah. Angelina, yeah.

… yeah, Angelina Jolie just went off and had a mastectomy without any symptoms or anything.

In her 30's.

Yeah.

Yeah, that's crazy to me.

Yeah, but you know, she said well I've got the gene and the research tells us at this point in time that I've got a high, high chance of it. Then of course you go on a few years and the research is now saying well no, there's not necessarily a genetic predisposition.

Exactly.

I mean, that the gene might still be there but most breast cancers are not the result of this genetic predisposition. So I think sometimes too if you, if you're, um, aware of research in a particular area, knowing how it changes over time, sometimes you know you'll zap in and do exactly what… you can only go with best practice at the time. None of us have crystal balls.

That's right.

But it's still… I don't know. I don't know, having done research myself I know (laughs)…

It's like any surgery, it's like any surgery you have, you have to hope that you've chosen the best practitioner…

Yes, yes, exactly.

… for you, who is going to do the best job for you… and you're going to have the best outcome. And that applies to anything that you've done.

Yeah.

But sometimes…

And sometimes, sometimes things are still going to go wrong.

I've been taken through emergency and as a private patient I've just taken the doctor that they said, yeah, this doctor can do it. Well, I really had no choice, you know? I was in a, a bad place and I needed the surgery.

[A FEW MMM's]

Now I'd never heard of this doctor.

Yep.

Didn't have the opportunity to speak to anybody about it. But he did a great job.

Yep.

So… yeah…

I suppose it goes both ways.

… it's the luck of the draw.

Yeah, I think so, yeah.

So your referring doctor…

I didn't have a referring doctor. I went…

So how did you… you went straight to the hospital…

I went…

[TALKING TOGETHER]

… I was ambo-ed in…

Yeah.

… and I, I paid a surcharge to go to Norwest Private…

Yep.

… and I was in there at about 11 o'clock at night. 8 o'clock in the morning I was operated on.

Wow.

Yeah, and I didn't have time to go, oh well, look I might hang around over the weekend, you know, see if I can find somebody else to do this.

Yeah.

[TALKING TOGETHER]

There's a difference whether it's life-threatening or not. If it's immediately life-threatening…

That's right, that's different.

… and the only thing, you know, you're semi-comatose or something or other, you might make a different decisions…

Exactly.

… than if you walk into your specialists rooms where you've made an appointment and you say, look, I've got a concern and we find… you know, it would very much depend on the emergency of the situation.

And that's, that's what I've done with other surgeries.

[A FEW YEAH's]

Just coming back to, um, the, you know, imagining that it's, you've been told it's DCIS, um… just wanted to give a chance to some quieter people…

Yeah (laughs).

… to allow you to… what, what do you think your initial response would be?

Um, if I had that I think I will, er, because if you keep it and it, er, may, the possibility become, er… oh, what you call it, bit worse…

Becomes cancerous…

… yeah, invasive, er, maybe I take it out, yeah.

You'd take it out. Yep. What do you think… is it Laura?

Cathy.

Cathy, sorry?

I'd, I'd, um, make sure it's fixed up before it got worse.

Mmm hmm. Ok.

What about you Barbara?

Barbara?

You think you'd just jump in?

Oh, well I, um… once again I think you're guided by what, what the, the doctor says. If you're, if you're really not happy with, with… that diagnosis I guess you can go to a… get a second opinion as they say.

Yep, yep.

Um… but I… I mean if, if it's only in one site you might say yes, ok. If, if it's going to be a form of mastectomy you might sort of have to think, oh, do I really want to go down that path?

That's right.

Or do I… um…

I guess the advantage of the DCIS diagnosis though, you have got time to actually…

Mmm. Consider.

… explore a couple of other, um…

Alternatives.

… specialists or whatever, and get a few opinions before you went straight into surgery. You know, you, you could make up your mind that I'm going to have something done but you'd… it's not like a life-threatening thing tomorrow. So you've got a little bit of time to actually investigate who you want to do your surgery.

Mmm. But depends how the cancer's slow. Some cancers very quick spread.

Yeah. But this one…

Some cancers still…

We're talking about DCIS.

[TALKING TOEGTHER]

But DCIS supposedly isn't.

But is it? It's a choice.

I would like to seek a second opinion.

Yeah.

And also, it's very important the relationship you have with the specialist.

Yes.

With the doctor.

Yes, yes.

And some doctors will feel comfortable if you… I wouldn't like to ahead and, er, and have that done if I don't feel comfortable with the doctor. Then you go to another one, he explains things better and you feel comfortable. You, you can trust that person and then you can make up your mind.

Yeah.

I have a problem, er, with my knee, I'm talking about a few years ago…

[TALKING TOGETHER]

Please, one person at a time. So, yeah, sorry, you were telling us about your knee problem that you had?

Your knee? What happened to your knee?

The meniscus broken, painful, especially… (UNCLEAR)… gave me even the, er… um… the price of how much the surgery will cost…

Oh yes (laughs).

… the booking and everything, and then…

They don't give you a chance to answer.

… straightaway, the cost, the quote and everything for the cost, book the room and everything. I said, no, I want to think about it. I went to see my chiropractor,

he gave me some exercise to do, I never had the operation and now the pain is gone.

Couldn't have been a torn meniscus then, I'm sorry (laughs).

Sorry?

I'm sorry, it couldn't have been a torn meniscus. It must have been... (laughs)...

It is, it was, it was because I had an MRI done.

All right, ok.

It was, it was.

Oh.

Because he exercise the muscle and the muscle, er, sustained it, the, er...

I had a knee since 1980 and two years ago I had a total replacement after so many reconstructions (laughs).

But the meniscus you cannot compare to, to the breast cancer, you know?

No, but we're talking about doctors.

Yeah.

Ok.

We're talking about doctors, how they behave.

Yeah, I definitely would seek a second or third opinion.

Yeah. But certainly in an emergency situations...

That's different. No, but that's different. You sort of just go along. But where you have time perhaps to make decisions I think it also very much depends on whether you have, and I mean even if you have top private health cover and you've belonged to it for years and you've been paying your Medicare surcharge and all the rest of it, I mean it costs you $270 just to walk into the specialists door.

Oh, my Gosh.

And you get what, $70 back on Medicare?

If you're lucky.

If you're lucky. So I mean, again, the choices that you make in terms of having a diagnosis like that are very much [COUGHS] impacted by your financial situation.

Yes.

And there are also many women around, women more so than men, who you know, doctor's an authority figure, if he said, she said, do this some people will just do it because…

Yeah. 'Cause they give them the authority.

… they're influenced by the authority so…

Well, again, yeah, back to the doctor who did my surgery last time, I wanted to go to Le Sands, because I've heard a lot about it. He just said, he listened to me and then afterwards he said to me, I'm, I'm a specialist, I can cost you a lot of money, but if my wife was doing this surgery I'd only go to a public hospital because if something goes wrong the amenities are available, you don't… (UNCLEAR). And when the doctor, a specialist tells you that, you're like well I should trust him because he's not out for the money.

Mmm, mmm.

And when he told me that and I'm like, you know what…

[PHONE RINGS]

Sorry.

… (UNCLEAR)…

No, I'm saying that all specialists are out for the money…

No, no, no, no, no.

Yeah.

I'm only saying that when a doctor, the way he reacts, you know, you interact with the doctor, does have a lot of, er, impact on…

Yes.

… the… how you feel or how you behave and what you want done.

Yep.

Yeah, because I was ready to go. He wouldn't let me go.

Mmm.

Ok, so I just want to, um, move along then to… come to an important question that is occupying DCIS experts at the moment. Which is could some women with DCIS actually avoid treatment?

Mmm hmm.

So current treatment for DCIS is based on our best scientific knowledge and of course wanting to do what's in the best interest of patients. Um, and as I said before, survival rates are excellent. But as we're discussed many patients do experience side effects from treatment and that includes both physical effects on women's bodies and emotional or psychological effects as well. And they can have an impact on women in the short term and in the long term.

So experts are now doing research to try and see if they can identify women who have DCIS but who have a low chance of getting invasive breast cancer, so that in future years women like this may be able to avoid surgery and other treatments, and therefore avoid all those problems that can come along with treatment, while still having excellent survival rates.

So as I said before, it's very difficult to reliably tell who these 'lower risk' patients are, but we do have some clues. And one of them is something I mentioned earlier called grade.

So after the biopsy is taken, the pathologist looks at the sample under the microscope and gives each case of DCIS a grade based on how the cells look, which gives an idea of how fast the abnormal cells are likely to grow.

So low grade indicates that the cells appear to have a lower level of activity, um, which suggests they may stay like that for a long period of time. On the other hand, high grade DCIS means the cells appear more active, and so they're likely to grow at a faster rate. So it's considered important to treat high grade DCIS because otherwise it's pretty likely to develop invasive breast cancer at some point.

I'm sorry, this grading happens for all cancer cells, doesn't it? Not just DCIS.

That's right.

Yeah.

Yep. Um, and then there's also an intermediate grade which is somewhere in the middle.

Now you might be wondering, if virtually all women with DCIS are having treatment, so having surgery to take DCIS out, why am I saying that we think low grade DCIS may stay as it is for many years and not cause problems, even without treatment.

Well, we think that maybe the case based on a small number of research, um, including studies similar to this one.

So just as one example I want to tell you a bit about a study that was done in America using a database of routinely collected information about cases of DCIS. Now these blue bars represent all the patients in the database who had low grade DCIS. So the vast majority of these patients, um, about 9000 people who are represented by this bar, were treated by surgery, which is the typical thing to do for DCIS, that's recommended in all the guidelines for doctors.

And then a small number of patients, just a couple of hundred, didn't have surgery for some reason, and we don't really know why not.

So this database kept track of key events that happened to these patients over a number of years, for example it recorded if any patients died from breast cancer. And what the researchers did in this study was compare the large group of women who did have treatment and the smaller group of

women who didn't, after 10 years. And they found that the women who did have treatment, um, after about 10 years, 99% of them had not died from breast cancer, so that's a very high survival rate, they were doing really well. And they found that among the women who did not have treatment, 99% of them also had not died from breast cancer.

Mmm.

And so this led the researchers to suggest that there may be a subgroup of women with DCIS who are likely to have really good health outcomes over time, whether they do or don't have the typical treatment of immediate surgery.

So this idea of a subgroup of DCIS that we could call 'low risk DCIS' is a big focus of current research, to investigate whether women in that category could possibly avoid treatment.

And there's a range of factors that are relevant to trying to define this category of DCIS – so grade is an important one but there's lots of other things they take into account as well that I won't go into now.

So coming back to this little diagram that we looked at earlier, I just want to give you a rough idea of how many women with DCIS might be classified into this of lower risk category. So of our 16 women with DCIS that we had before... we think about 5 women have what could be classified as lower risk DCIS based on their biopsy.

For the other 11 they're considered to have a higher chance of getting invasive breast cancer, so it's still considered more appropriate that these women do have treatment.

But the 'lower risk' women are the focus of this current research, looking at whether they might be able to avoid treatment. And I'm going to tell you more about that research shortly, so it's just important to remember that we're talking about this quite small and select subgroup of women with DCIS where researchers are considering a new, different approach to managing their condition.

So the type of studies I want to talk about now are called clinical trials, so

let me explain what they are.

A clinical trial is a research study that asks an important, specific question about a certain health condition. Clinical trials often test out new ways of managing a condition, by comparing a newer approach with whatever is the current standard care.

And trials are designed to find out whether patient outcomes (such as health and quality of life) are similar for both options, or whether one option is better than the other.

Now if you think back to that study we talked about before, with the blue bar graph, it was done by looking at a database containing routinely collected basic information about past patients. And although studies like that can be valuable for giving us some interesting ideas, it's hard to draw firm conclusions from that kind of research because there's a lot of important information missing, for example details about those patients who unusually didn't have surgery and why.

Whereas clinical trials are specially designed to overcome limitations of other kinds of research. And that's why trials are considered to give us the highest quality of evidence.

So… this illustrates the basic design of a simple clinical trial. Sometimes they can be more complicated, for example more than 2 groups, but most typically, each patient in the trial is put into 1 of 2 groups. One group of patients gets the standard treatment, while the other group gets a new treatment or a new way of managing their condition. And which group each patient goes into is determined by chance. This process is called randomisation and it's a very important part of clinical trials. Because when we compare 2 groups of patients who are having their condition treated or managed in different ways, we want to make sure the groups are very much the same as each other except for the way their condition is being managed. 'Cause if patients and their doctors choose which treatment to have, then the patients in the different groups are very likely to be different from each other in important ways related to their health, and that can create a bias that makes the results less reliable. So patients who volunteer to take part in clinical trials need to be comfortable with

ending up in either of the groups because they don't get to choose which group they're in. So this process of randomisation makes sure that the study groups are as similar as possible. And that way, we can get the most reliable information from the results of the trial to learn about the effects of those different ways of managing the condition.

And deciding by chance how a patient will be treated might seem strange, but it's considered an ethical thing to do in situations where we genuinely don't know whether one option is really better than the other.

Mmm.

So there are currently 3 clinical trials going on around the world for women with low risk DCIS: one in the UK, one in Europe based in the Netherlands, and one in the US. They all just started in the last couple of years, and together they will involve over 3000 women. Now there are slight differences between the trials, but they all have the same general design.

And the aim of these trials is to learn whether women with low risk DCIS could safely avoid having treatments that may not be necessary for them.

So the women who are invited to take part in these trials have to meet strict criteria to be considered suitable. They have to have low or intermediate grade DCIS (not high grade), they can't have any lump that you can feel in the breast, um, plus there's a lot of other more technical rules I won't get into. So remembering that this is what typically happens to women with low risk DCIS: they have treatment pretty much straight away, and we talked about those treatments before of course, the different types of surgery and radiotherapy.

Now of course in the following months and years, if it seems their initial treatment hasn't worked quite as well as they hoped, or their DCIS comes back, or they get DCIS elsewhere or invasive breast cancer, they might end up also having some further treatment if needed.

So that's the normal scenario for pretty much all the women with DCIS, um, in places like Australia where there is no clinical trial. For women who do have a trial available where they live, if they're suitable to offer the trial,

they may just say no thank you, I don't want to go in the trial, I just want to have the normal, standard treatment. Or, if they decide to join the trial... then they get randomised to either usual treatment or the other group which is called active monitoring. So the usual treatment group of course has usual treatment. And the other group has a different approach where in the first instance they don't have surgery or radiotherapy. And then the patients in both groups are monitored closely, so they all have mammograms at least every year. And importantly, patients in the monitoring group may have treatment later if there's any sign that their DCIS is progressing, and we expect that to happen for some women. So this approach may be called active monitoring or active surveillance and it's actually already an accepted option for men with early prostate cancer because trials similar like this in the past have shown that it can be a safe. So these trials will test out the idea that this monitoring approach where you only give treatment if it's needed may result in the same very high survival rates as standard immediate treatment, but with fewer side effects overall because some women will avoid treatment altogether, and others will end up having treatment down the track but it's delayed so they may have a period of some years where they're free of any treatment side effects. So those are potential advantages of monitoring.

There's also potential disadvantages that the trials will investigate. For example, patients who don't have immediate treatment may be more likely to get invasive breast cancer – although with the monitoring it's still likely to be picked up at an early stage. As I mentioned before, it's also possible that some of these women who are diagnosed with DCIS based on the biopsy actually have invasive cancer at the time that the biopsy didn't pick up. And we also don't really know how women would feel about not having treatment, so in the trials the patients will do questionnaires to help the researchers understand the psychological effects of not having treatment for DCIS, and that's a very important aspect of this research as well.

Now because there's only a small and select group of women who are eligible for these trials, and because they then need to be followed up for quite a few years, it will still be some years before we know any results of these trials.

Um, in the meantime, it's possible that a similar study could be started in Australia and New Zealand, so women here might have the possibility to go into a trial like this.

But otherwise until the results of these other trials are published, women with DCIS will continue to have treatment as normal because at this point we don't have strong research evidence to show that any other approach, like active monitoring, is really safe.

So what are your thoughts and feelings about these clinical trials?

It really comes down to how much risk there is that there is invasive cancer as well as the DCIS, isn't it? Because… they're the only ones, it's those people who've got DCIS and got invasive that nobody knows about until they actually do the surgery and then they find it. They're the, they're the really trouble spot aren't they?

Mmm hmm.

And if everyone only had DCIS… and said no, I'll just monitor it, then there's not really any more risk to them.

No.

'Cause is they're been having really regular mammograms and things as soon as it goes outside those ducts it'll get picked up and they'll do something then. It's really those ones who've got invasive cancer that they don't know.

Mmm hmm. Ok. What do other people think?

Um, I've had a family member who went on a trial for leukemia and, um, it's something I will never do. Er, the experience and then when it was too late… the people who were running the trial washed their hands of him. And that was very, it was… it was a very sad thing.

I mean the ethics of clinical trials, I mean most, most legitimate ones…

Yeah, yeah, yeah, I understand…

… have a very strong ethical component.

Yeah, yeah.

The have to be. But you will get the odd one where that happens to you depending on who's running it.

Well, well it did happen and that person did pass away 'cause when it was too late they just said, take him to the nearest hospital. That was it.

But new, new treatments can't be…

That's right.

Yeah.

You take…

… developed or, or researched…

… *yeah, yeah, yeah… but this is about…*

[TALKING TOGETHER]

And when you sign up for those that's explained to you.

Yeah, yeah, but that was a very bad experience.

[A FEW YEAH's]

Yep, and that's always going to impact on your decision making…

That's right because if I didn't, if we didn't have a bad experience…

… (UNCLEAR)… yes.

… I'd probably be all for it.

But you know there are clinical trials and there are clinical trials.

But that's right. I'm not saying all clinical trials.

Yes, yes.

I'm just saying a particular one, that dealt with leukemia, and it was a group of

people from Germany, doctors, up in North Shore Hospital. But, yes, the experience wasn't, um… as we'd hoped for.

Mmm, ok. So, um… so basically as, as we said before, the typical experience of women diagnosed with DCIS is they're recommended to have treatment.

That's right.

They're recommended to have surgery. And some women now might be given an option to go into a trial where they would be randomised to either the normal pathway or this new, this alternative of monitoring. So it's, it's a bit hypothetically but how…

It depends which pathway you went into.

That's right.

If you went down and had surgery you'd know.

You'd know you were on that pathway (laughs).

[LAUGHTER]

If you went down the, the monitoring, you know, oh, I'm in a clinical trial, so…

Yeah, yeah.

No, so you, you would agree upfront to either go in the clinical trial or not…

Or not, and then it would be a matter of which ballot came out, yes.

But then you're randomised.

Yeah.

But then you're randomised. So if you have surgery, then you…

Yeah, but you would know that but it's not necessarily going to impact on the results of the study (laughs).

But would you…

But randomisation…

[TALKING TOGETHER]

And that's really just to make it more scientific because randomised samples have more credibility than…

Yeah, yeah, I know, but…

… than we'll pick you and you because you've both got brown eyes and I'm wanting to find out what happens to brown eyes…

Well, that, that leukemia research was randomised as well.

Right, yeah.

Yeah…

[TALKING TOGETHER]

… before, before the people are assigned randomly, the group, everyone would have to agree to, you know, that I might be in the group that doesn't get treatment, are you willing to still be part of the study? Which of course in that instance you're going to get people who might have been fabulous candidates who say, well no, I don't want to be part of this study.

Yep.

Or you get, get people who choose to be. So they… you know the, the people…

But what, what… the problem that we experienced was, um, you don't know what the outcome's going to be.

Yeah, but you would know that going in.

Yeah. No, no, no, but…

Presumably.

… what you are promised or what you ended up with was two different things.

Right, ok.

You were promised something and then when it was too late [CLAPS HANDS].

Presumably though, before you participated in the trial, if it was ethical…

Yeah, it would have been…

Yeah, but if it was an ethical study…

[TALKING TOGETHER]

No, no… I wasn't heavily involved in it, um, but in the end when they said… when they said that, yeah, but I wasn't involved in it.

Yeah, so that sounds like…

Um, this is just a general knowledge…

So the person involved could have actually been told that that could be a possible outcome and they chose not to hear that.

Well yes, that's right.

If you were diagnosed with a low-grade DCIS, you could then make your choice, because I'm sure that your treating doctor would follow those sort of guidelines and do regular testing to determine whether the grade of your cancer increased or not and then you could make the decision based on that information. If you have a low-grade DCIS and it was suggested to you that perhaps you don't need immediate surgery… most people would probably follow that…

Yeah.

… information but would have… um, follow-up tests and whatever over, you know, the next period of time. And make that, make that decision without necessarily going into a trial, whereas Barbara said, where you'd know whether you were going to be the treatment that's currently there or whether they're going to hold off. I think it's more that you would have to make that personal decision that, no, I'm not going to have invasive treatment. I'm going to sit back and wait and see.

In fact that's one of the things to be glad about. If you were diagnosed with low-grade DCIS…

That's right.

… then you actually do have the…

You have the choice.

… you have time to make decisions. Whereas a lot of people who get a cancer diagnosis, they know they don't have time.

Well that's right.

So in a way, if you have to have a cancer diagnosis it's probably one of the nicer ones to have.

So, so I don't see how a clinical trial set up like that would work.

Mmm, yeah.

'Cause you would know…

Yeah, but…

... your outcome.

So you would know, um, you know which…

You know what, what team you're on.

Yep, but then what happens is they follow-up closely what happens to you over time and that's how they, um, learn… what the, um…

Ok, so…

... what the outcome of...

So, so if they could say, well people with this, this grade, if they choose not to have surgery and then they could get numbers for how many would perhaps need surgery in 3 years, 6 years, 9 years…

Yes, yes.

… and put up all their statistics in that way.

How many with the DCIS actually do progress to something…

Yes. To something worse.

… yeah.

That's right.

So that's the information that you get from the trial, yeah.

So it's good, it's good from their point of view.

Yes.

It doesn't really do much for you.

Well it does because as the testing progresses if your condition deteriorates then you put your hand up and you say sorry, I'm walking away, I'm going to go and have surgery.

By the same token, if your condition didn't become something else…

That's right. You go…

… you can then save from having unnecessary surgery in the first instance.

Yes.

Yeah, so, um… if you're in the monitoring group and your condition progresses and you're recommended to have surgery, then it doesn't mean that you have to leave the trial. You would stay in the trial…

Ok, then you would join that other group where you become…

Well, um…

You're just another…

… a statistic.

… you're just another variation in the…

Yeah.

That's right.

Yeah.

Because the, the active monitoring approach means…

Yeah, ok. I follow.

… monitoring from the begin, with treatment later if you need it.

Mmm.

So they'll still be comparing that group with the group that had treatment.

But doesn't that mean that's another extra group in the middle? That…

No.

Well no that's…

[TALKING TOGETHER]

… their path is different.

Your outcome would be of the monitoring, of the active monitoring group…

Yeah, but what I'm saying is…

[TALKING TOGETHER]

… down the track 80% didn't, that might be a result.

Yeah, so basically they'll use different, um, statistics to make lots of…

The progression. Yeah.

Yeah, and take everything into account.

Right.

But, um… yeah, so, so important, it's important to realise that at the moment, um… in the absence of results from these trials clinicians will, will tell you, you know, what we do and what's recommended to us in our guidelines is to give you surgery. So, um, I'm not saying that it, it never happens that women, you know, obviously no one can force you to have the

treatment, but you're not going to really be offered, well, you could have surgery or you could not have surgery. Um… 'cause at the moment, um… clinicians may feel comfortable to offer you to go into a research study if there's a trial like this, you know, if there is one and you're suitable, but otherwise they will tend to say that, you know, the, the right thing to do is have surgery. So, I'm just trying to convey that, you know, you're kind of options that I'm presenting you hypothetically could be either to go into a trial where you may end up having surgery or not having surgery, versus having the treatment.

Ok, so as yet you don't have any figures on how many people decide not to, not to have surgery but then who progressively through that trial do require surgery. So you don't have…

That's right.

… an attrition rate for those who've fallen… or by necessity have come out of the decision that they're not going to have surgery because their condition worsens. So you can't say, well we had 500 women who followed the no surgical path and of that 500, over a period of 10 years, we had 280 who needed to go and have surgery. You don't have those statistics, yet?

No.

Ok.

I mean the researchers presumably would have some formative data, um, but they might not be releasing it (laughs). So you wouldn't have that…

And it's not clinical data so, like it's not… hasn't been randomised and everything else, so the people, the people with the numbers could have had other conditions as well that you don't know about.

Presumably they would have taken that into account in terms of…

[TALKING TOGETHER]

… well they'd probably do all the tests then and make sure you're virtually on the same health…

… with how many, with how many, it was just survival rate you called it, um, out of the, I've forgotten the numbers, but it was a small number who didn't have the surgery and they didn't die of breast cancer. They might have been hit by a bus the next day, you know, like…

Exactly.

… but the numbers… the fact that they didn't die of breast cancer is really wishy washy because…

But presumably your…

[TALKING TOGETHER]

… your suitability to be selected for a trial, they try and, um…

Oh yes, I…

… work for those variables.

Oh yes.

So if you had another form of cancer, well they possibly wouldn't consider you.

But the numbers that were presented before…

Well we don't know.

… that wasn't actually a clinical trial.

No.

They were just random numbers of a thousand.

Yeah.

And that's what I'm saying, that's why they need to do clinical trials to come up with that data.

Mmm. I've got you. Yeah, I've got you.

'Cause those, those numbers were just… you know, out of a thousand, you know…

I think it's very good idea.

You think it's a good idea to do the trials?

Yeah.

Yeah.

Or to get more information.

To get more information so that people can make, um, a choice when they're diagnosed…

They can have an informed…

Yeah.

… yeah, they'll have information…

Yes.

… on which to base their decision to either go with the surgery or not go for the surgery.

Yeah.

They could…

Like, er, when I was 25, 26 year old I have… I have baby, new baby, and maybe through the… pushing things, have something here, little lump on my neck. So when the baby 3 months old doctor advised me to took, they do biopsy and just fluid there, so I have big operations, not big, not very small, but I stayed there 10 days in Westmead Hospital. So they found the bananas, not cancer, just… they called banana? In the thyroid. So I have half my thyroid gone out. And another few years later I saw my friend. She has same problem. And she said you do drugs (?). I said, why? She said, why you took out? Now the medical have medicine for it, you shouldn't do your operations, it's true, you know?

That's right.

That's true, yes.

That's right, that's progress though.

Every year, in January, I did it in January, it's very, very good doctor who did it for me, but every year on the same time I have the operation I feel like a knife cut in me. Every now, three years I never feel it, but since I was 25 year old, I feel 22nd January and I…

[LAUGHTER]

… just happen for three days, I feel this feeling and then been three years I never feel nothing.

Well that's a good thing.

Mmm, so yeah, so are you saying, um, the trial would be a good opportunity to maybe avoid surgery?

To make a decision.

[A FEW YEAH's]

To make a decision.

[TALKING TOGETHER]

The quickly advise me you have to be in hospital, they send me a letter, they ring me…

That's right, you didn't get a choice.

No choice. I just…

And that's what we're talking about, when you don't get a choice.

… left… four young children, the oldest was 6 and they youngest was 3 months old. And I did the operations. So it was too much for me that time.

Yeah.

So progress in medicine tends to happen…

[A FEW YEAH's]

… when they have more information…

[A FEW YEAH's]

… which they tend to get from clinical trials….

[TALKING TOGETHER]

I would see more people and more people advise me to do it or not to do it, I think it's better to have this, er, trial and know people talking about it.

[A FEW YES's]

But you were quoting before the, um, prostate cancer. I knew lots of me who like the first diagnosed at all… like… they'd make a choice, they, they had a choice but they were, they were advised to have full surgery. And in hindsight now most of them probably didn't need it.

Yeah, yeah… always get opinions, always get opinions.

And, but you know, if that's the result of a clinical trial that's eventually come up with who should have surgery and who shouldn't, then that's great.

But you should have to work on the information that you have at the time.

Well, it takes time, that's the problem.

So if you would… if you receive that diagnosis and your specialist could say to you, we have this information and based on this information that would be my advice to you, then you're getting, there's something concrete to base this information on.

Mmm.

He's not saying, oh well, you know, a lot of women or some women or… you can get the actual data. So I, I think a trial like that would be great.

Mmm hmm. Ok, and… so it'll, it'll be great when we have the results of the trials.

Mmm.

Yeah.

Um, what about in the meantime, if you can try and imagine if you were diagnosed, you know, soon and, um, if you were offered to go into a trial, do you think you'd say yes, I'll go in the trial?

I would if it was a low-grade.

Yeah. I would as well, because you're still being monitored so you're not sort of just saying I'll be ignoring the diagnosis.

I wouldn't go into the trial because they might put me in the surgery group and I wouldn't want the surgery group.

No, no, no, no, no. They…

That's right, yeah.

… if they put me in, in to monitor, to, to…

To monitor, yes.

… to track the development…

But not surgery, no.

… if any, because it might be what it is and it's not going to get any worse.

That's right. That's what I…

You'd really, really…

[TALKING TOGETHER]

… whether you just wanted to be monitored…

Or surgery, yeah.

… or have surgery. You wouldn't want to…

Yeah, if you can choose that, then it's good.

[LAUGHTER]

Yes, but then it wouldn't be a randomised trial (laughs).

It won't be a randomised trial. That's right.

Well like there's no reason why your data, you couldn't sort of say well as a side bar you can, if you're quite happy, I'm quite happy to give you my results as I, as I…

As I… yeah, I wouldn't mind doing that, yeah.

Yeah.

Yeah, but I wouldn't want to be put in a position where I don't have a choice. I like to have a choice

Ok.

That's the only reason I, um… based on experience I would have a choice.

So we think they're a good idea but personally I wouldn't be…

Yeah, yeah.

It is, it is pretty invasive surgery, yeah.

Yeah, it is. It's major.

I, I… I've got a friend at the gym who 10 years ago had to have total mastectomy, and she's had a tough time…

[A FEW MMM's]

… in that time.

Mmm.

Yeah, and she's only just now, you know, feeling well enough and motivated enough…

To move on.

… to get herself to the gym. She had to finish up work, yeah… it's been an absolute nightmare of a path for her, with a total mastectomy and reconstruction…

Yeah.

… that wasn't necessarily done well the first time.

Oh… yeah?

And the stories that she's been telling me over the last couple of months (laughs)… yeah, it, it's… pretty bad…

Makes you wonder.

… but she had no choice.

Yeah.

And she accepted the information she was given and she said, I had no choice.

Mmm. Wow.

And she's alive to tell the story.

True.

She's alive to tell the story.

With a lot of suffering.

It's not even the surgery, I mean you might not wake up from the anesthetic (laughs).

[LAUGHTER]

There's a risk every time you have a general anesthetic for heavens sake (laughs). Even for minor, minor surgery.

I know.

That's what I'm saying.

Yeah.

Things can go wrong with the littlest surgery.

Yeah, yeah.

Yeah, exactly.

Yeah, but we're talking on the basis of that, not…

Yes.

… everything is on the basis of that particular…

I know, I'm just being…

We're just presuming that everything goes well…

Yes, that's right.

… and all you've got to go through is…

We might not trust some doctors but we believe in the sanctity of anesthesia (laughs).

[LAUGHTER]

Yes.

Ok. (?), what do you think? I'm putting you on the spot.

Would you go into a trial?

Um, yes, I would go into a trial if it helped.

Ok.

Mmm.

So then you would be comfortable with the… the decision would not be yours which way you, you might have treatment or you might not have treatment, but you would be contributing to… the…

Greater good.

… learning, yeah.

Just want to… I'd just want to fix it all up and make sure that I was getting better.

Ok.

Mmm.

So does that mean you would rather have treatment…

Yeah.

… then maybe you wouldn't go in the trial 'cause in the trial you might end up not having treatment.

Mmm, yeah, well I just want to get better.

Mmm.

Mmm.

I'd like to be randomised to not have treatment if it was low-grade.

Yeah.

[LAUGHTER]

You want your cake and to eat it too.

[LAUGHTER AND TALKING TOGETHER]

It still, it still comes back to that whole point though that you could be low-grade and still have invasive cancer and not know.

Yeah.

Yes.

Yes.

Unless you had the surgery.

Yes.

That's true.

Yes, but eventually if you were monitoring you would eventually pick it up.

Yeah, eventually, but… you know, how invasive does it get before it's… you know, there's none of that data either really is there? So…

No.

Well that's a valid point Rayleen.

Yeah. If you actually went in…

I, I think I'd still go… so that's the best thing that we've got today and I got diagnosed today, I'd just go and have the surgery.

So you wouldn't go in the trial either.

No, no.

'Cause you might be in the monitoring group.

Yeah.

Um, could I just, this is probably something…

But you've got the psychological worry the whole time.

… but that's right, yeah.

That if you, ok, I'm just going to monitor it, but at the back of your mind there's this little person on your shoulder going, you've got cancer cells in there.

Yeah, yeah.

Your days are numbered.

That's right.

Whether there's a lot in the ducts, a big number or a little number, you've got cancer cells.

Yeah, yeah, yeah. A while back there was a, a program about a guy in his 70's, was diagnosed with cancer and the doctor said to him, you'll be lucky if you have 6 months, 12 months to live. He sold his house…

(Laughs)

… packed up, decided to travel around Australia.

I've heard about this.

On his way he picked up a hitchhiker, a young lady from Germany. Five years later he's in his 80's, they've got a 2 year old son and there's nothing wrong with him.

(Laughs) Yep.

See what I mean? He was told you've got maximum of 12 months to live.

I have a friend who was given three years, five years ago. And she's still (laughs)… she's on oxygen and all the rest of it, but she's still… she's not in a great state, but she's still with us.

Yep. Yeah, but I'm talking about this man who sold everything and decided to travel Australia, in a caravan.

Yeah.

And then…

And he now has a toddler.

He's got a toddler. She's in her 30's, he's in his 80's.

[TALKING TOGETHER and LAUGHTER]

Not a good thing…

Give me back the cancer (laughs).

What I'm trying to say is if he's dying, if his cancer was so invasive…

[LAUGHTER]

(UNCLEAR)

… that's what I'm talking about.

There are also people, I mean you're talking about the little brain, the voice in the

back of your head saying that you've got cancer, but there are equally, there are some people who's little voice just buries it's head in the sand and says, well she'll be right mate.

Yeah. Well this is what…

[TALKING TOGETHER]

… your personality…

Exactly.

But that's, that's the point of clinical trials as well, to, to actually try and capture that stress level as well, isn't it?

Yeah. Definitely. What were you going to say?

In my opinion it's person hasn't got fever or pain or nothing's wrong, if you see anything in yourself and doesn't worry you, don't go surgery. Because sometime surgery's good, helps, sometime effect more.

Doesn't help, no.

Mmm, ok. Margaret, what are you thinking?

Me?

Me?

Oh, sorry, I can't actually read anyone's name.

Um…

Yeah.

… well I, as I said before I, I agree with clinical trials, you need clinical trials…

Yes.

… in order to progress medicine.

That's right.

Um, but in this particular instance you know which group you're in and, um… I mean, if you could be told yes, it's only in, you know, one or two ducts and that's low-grade, I'd probably wait and sort of, um, see how it went. But if it's in, you know, three-quarters of your breast and, and you know…

But that's different, yeah.

… and all these cells are going to start…

But that would be high-grade, wouldn't it?

… multiplying more, but, you know… yeah.

But that would make you high-grade and that would make you…

[LOTS OF YEAH's]

So you wouldn't be eligible for the trial, yeah.

[TALKING TOGETHER]

Yeah, um…

Yeah, so, so basically… you know, you'll, you'll either be told, um, that you're a candidate for a trial like this because we don't really know whether it's better to treat you or not treat you. Or you'll be told you're not suitable for this trial, actually your condition needs treatment. So, um, I'm… yeah, if, if you are told that, you know, if you're invited to go in a trial it means that the doctors really don't, honestly don't know…

That's right.

… whether you should have treatment or not.

Yes. Mmm.

So then I'm, I'm asking you if you're in that situation would you be comfortable to go in a trial where that then is decided by chance…

Um, yes probably…

… or would you feel…

Yes, probably. But I mean…

Yeah.

If you don't like the choice you're given you can always walk away.

Yeah, that's right.

Can you actually walk away?

Yeah, they can't…

[LOTS OF YEAH's]

… they can't put you in manacles (laughs).

[TALKING TOGETHER]

But some people go into a contract where…

Yeah, so you can always withdraw from a trial.

Yeah.

Um…

Well if that option was given then I'd probably go into it, yes (laughs)… 100% (laughs).

Yep.

Yeah.

So knowing that you could then…

That you could walk away any time.

Mmm hmm.

Yeah.

Yeah, but you might have had surgery before that.

(Laughs)

I mean if you go, if you're saying you're going into the trial…

And you're put into the surgery group, traumatic surgery.

… but seeing as you're going into the trial you might have surgery.

So you actually go straightaway and they give you surgery? That's what I'm talking about.

But you discuss it first.

Half… half, half the people.

Yeah.

Yeah, but if you're told well you've been put in the surgery arm you could still withdraw.

You can still have that choice.

[LOTS OF YES's]

That's what I'm talking about.

Oh, ok. All right.

If you are in the surgery and you don't want it, you can walk away.

Yeah.

That's what I'm talking about. If you can then that's a different option.

Yeah, yeah.

Not just you're (?) surgery, you've got to have surgery.

What would you do?

Oh, I, yeah, I'd try the trial.

Yeah, I'll try on that basis.

'Cause you can withdraw.

That's right. That basis it's alright.

Yeah.

Yep. I think we're all want lots of options, don't we?

Depends on the policy.

Yeah.

And it depends on your condition.

I would go in the trial, I think, yeah.

Yeah?

Yeah, thinking about it now…

Yeah.

… I would like to think about it a little bit, you know, but I would probably, yeah, go for the trial and then, er, should I deteriorate I can always have the surgery later on.

That's right.

Yep, yeah.

That's right, 'cause you're given an option.

Considering, er… the studies that they've done on people who, er, had the, er, the treatment, the surgery or not, I would follow, I would go on the trial, yeah.

Mmm. Ok. Well, um, we're getting towards the end now, so, um, before you go I'm going to ask you to complete that questionnaire number two. So just before we all do that, is there anything else that you want to say that you haven't had a chance to say, um, about this topic, you know, before we stop talking? No?

I, I think the idea of, of a trial to see, to, to compare, um… to look at the people who, who opt not to have surgery and, and to just determine how many end up having surgery, because in the beginning if, if it's diagnosed as low grade all of

your test results are available for ever and you can at least then track, or somebody can do another trial, another research and track the development of a low-grade into a moderate an perhaps to a high-grade, and that gives you more of a basis to be able to advise people as to whether… a low-grade, yeah, what the… what the, um…

Chances…

… chances are of a low-grade developing into a high-grade over your lifespan.

So like an advantage, an advantage of a trial is that it may also have data and come up with results that…

Yes.

… weren't originally the original questions…

Yes, yes.

… and so then you might actually get more information.

Yes.

That's right.

And that, that's what research is all about.

Yep.

Because anybody doing a PhD has always got to do a literature review and come back in and say, well, you know, these people did this result, this research and, um… it, um, it sits with my research in this way. So, yeah…

Mmm.

… research is always a good tool.

But they need to track all the peripheral things.

Yes.

So you've got people in clinical trials, they're tracking everything else that might

change in their whole wellbeing…

Yes.

… in that progression.

Well it will throw up some correlations…

Yeah.

… as well as some causative effects and things like that too, and then that gives someone inspiration to find out perhaps to take this little pathway into more detail.

[TRANSCRIPT ENDS 01:48:05]

END

Interview: DCIS FG6 2018.05.28

Interviewer: **BOLD TEXT**
Interviewee: PLAIN TEXT
Pauses: …
Unclear words: (?)

[TRANSCRIPT BEGINS 00:16:09]

So, um…

Thank you.

… as I said before, I know DCIS may well be quite unfamiliar to you I'm going to start by talking about something that probably is at least a bit more familiar, um, and is relevant to DCIS, and that's breast cancer screening.

So what is breast cancer screening? Well in Australia we have a national program that provides screening, free of charge to women, to look for early signs of breast cancer.

Now it's important to understand that when we talk about screening it's something that's designed for women of a certain age in the general population who are well. So it's not something for women who have a breast symptom (like a lump or pain or discharge) that needs investigation – that's a separate process.

But the aim of screening is to find women who have cancer early, so that they can be offered treatment early, in the hope that earlier treatment will lead to a better outcome. And the main goal of screening is to reduce the number of women who die from breast cancer.

So the best available method for breast cancer screening is a procedure called a mammogram, which uses x-rays to make images of the breasts. And the whole idea is that these x-ray images can show up cancers or abnormalities that may be so small they can't be felt as a lump.

And if the mammogram images look abnormal or suspicious in some way, the woman gets called back to have further tests which may include a biopsy. This means using a needle to take a small sample of cells from the breast to look at under a microscope, for the purpose of confirming the

diagnosis. So from looking at what that small sample of cells looks like under the microscope, a pathologist will then either say actually everything's fine and it was a false alarm, or they'll diagnose breast cancer or DCIS.

So, ductal carcinoma, DCIS, ductal carcinoma in situ, is a condition that can affect the cells inside the breast.

Typically, DCIS doesn't cause any symptoms, so it's found through screening. And before we started doing screening in the population, DCIS was hardly ever diagnosed. So in the past, women may have had DCIS but because it didn't cause them any symptoms and because there was no screening, they wouldn't have known about it. Whereas nowadays, now that we have quite widespread screening, um, DCIS is getting diagnosed a lot more commonly than it was before.

And part of the challenge with this condition is that it's almost like DCIS is kind of a new disease that we've only really discovered through bringing in population screening programs, in Australia and other countries, and that's happened from about the 1990s. And so everything that women are told when they're diagnosed with DCIS, and everything that I'm telling you today, is the best information we can give based on current scientific knowledge of DCIS. But it's important to understand that that knowledge is somewhat limited. So there's still quite a bit we don't really understand about DCIS.

I'm going to show you a diagram now and some numbers – don't worry too much about the numbers, it's just to give you a bit of an idea of how many women are affected by DCIS.

So imagine that each one of these dots represents one woman. If we take 1000 women in the Australian population who have breast screening over a period of 25 years, in total about 81 women will receive a diagnosis of one kind or another, as a direct result of the screening mammogram and the further investigations that follow.

And then if we look in more detail at these 81 women, we find that 16 of them have DCIS and the other 65 have what's called invasive breast cancer

– and I'm going to explain the difference in a minute. But as you can see, the women with DCIS are a smaller group – so they're about 1/5 of the total women diagnosed through screening.

So let's talk about what is DCIS is in a bit more detail, breaking it down word by word. Ductal means it relates to a duct or a tube that carries milk to the nipple. Carcinoma means the cells show abnormal features similar to cancer.

And 'in situ' means contained in the original place. The 'in situ' part is a really important to defining DCIS. So this illustration shows how DCIS is different from 'invasive' breast cancer, which is really just a more precise term for what we commonly understand breast cancer to be. So based on what they can see looking at the cells from the biopsy, the pathologist will diagnose that it's either DCIS, which means the abnormal cells appear to be fully contained within the duct, or invasive breast cancer, meaning that some of those cells have moved outside the duct into the breast tissue surrounding the duct.

So DCIS and invasive breast cancer are different but they're related. In DCIS, the abnormal cells are contained within the milk duct and have not spread outside the ducts into the other breast tissue. So as long as those cells remain contained like that, DCIS is not a life-threatening condition at all. But DCIS might develop into invasive breast cancer later on, and if that happens the time frame for it to happen is really variable, so it could take many years or it could happen much more quickly.

In invasive breast cancer, some of those abnormal cells which were previously just inside the ducts have spread into the breast tissue around the ducts, so it still can be entirely within the breast, it doesn't mean that it has spread to anywhere else in the body, um, it's just the fact that it's gone outside the duct that means we label it as invasive breast cancer. But once that's happened it means that the cancer could then spread to other parts of the body, and that's when you have a problem.

But it's good to remember that although invasive breast cancer has the potential to be a life-threatening disease, these days the majority of women diagnosed with breast cancer will be treated successfully and will survive

their breast cancer.

So on this slide, um, there's just a few of the different phrases that are used to describe DCIS. And none of these are really wrong but it can be a bit confusing for women because DCIS is described both as a type of cancer and as something that's not quite cancer.

So even if you haven't heard the term DCIS before, you might have come across some of these phrases like Stage Zero breast cancer or non-invasive breast cancer or pre-invasive breast cancer.

So what happens after DCIS is diagnosed? Well, as I've said, DCIS itself doesn't affect a woman's health, but it has the potential to turn into invasive breast cancer. Now we have reason to believe that this progression to invasive cancer might not happen for everyone, and I'll explain more about that later. But the bottom line is that right now we can't reliably predict which of the women with DCIS will get invasive cancer or how long it may take before that happens. And that's why virtually every woman diagnosed with DCIS is given treatment, to try and prevent that progression from ever happening. And I'll talk more shortly about what that treatment entails.

But firstly I just wanted ask all of you, so after that little explanation have you ever heard of DCIS before?

[LOTS OF NO's]

Yes (laughs).

Ok, lots of no's and one yes. In what, er, situation have you heard about it?

That was what my daughter was diagnosed with, DCIS.

Ok.

Yes. But it progressed. It had progressed. They didn't realise until they did a full mastectomy.

Ok.

Is, is that, um, does that present as a lump? Would you find that as a lump?

Typically no.

No, you only find it…

So typically you only find it through screening.

A mammogram. Mmm.

A mammogram. Was that the situation for…

No, she did have lumpy breasts.

Ok.

So she was doing a 12-month regular mammogram because they said she was high-risk. That's after feeding four children too, so… (laughs)…

Yeah.

… it was sort of not typical for everything you read. But… nevertheless, um…

Ok.

... if she hadn't been careful…

And so was one of those yearly screens that…

Yes.

... picked up…

It picked it up, yes.

Ok. Um… do you feel that you've understood what I've said so far?

[LOTS OF YES's]

Is there anything that's unclear or confusing? Any questions entering your mind just now?

No.

Ok, um… so could someone just recap for me what, what the difference is between DCIS and invasive breast cancer?

It doesn't…

Well it… sorry.

Well the DCIS is within the milk duct…

Mmm hmm.

… whereas the invasive one can be outside the milk duct.

Exactly. Yep. Ok. So, um, based on learning about the existence of DCIS for the first time, what are your thoughts and feelings about it?

I think we should have mammograms…

Yes.

… as often as we can.

Mmm hmm.

Well I'm wondering about, um, the practice for example followed by my own GP, which was to tell me that she didn't think I needed to have them any more?

Mmm hmm.

Is that right? Or…

So…

(Laughs)

… we, we can talk a bit more about, about that, um… the situation with the screening program is that it used to be targeted to women between age 50-69. And just recently they've decided to extend the target age up to 74. So, um, women in their 70's, which I think is all of you, um, are kind of in that group where there's been a bit of a change in the recommendation.

Yeah, well my feeling is generally I am happy to follow the advice of my doctors

who I think are very competent. But I'm… what I've heard today has made me wonder about whether I should be, um… so willing to allow the decision to be effectively be made by the doctor.

Mmm hmm.

Can I say something?

Mmm hmm.

I know somebody who was diagnosed with breast cancer at 81 only through a routine mammogram, so…

[A LOT OF YEAH's]

You can still get mammograms after 74, they're still free, but you've got to remember yourself.

But I do think I kind of get panicky about it because x-rays are not good for us either (laughs), so…

Mmm.

… regular things are what we need, isn't it? I mean, aren't they?

Mmm hmm.

And is it prevalent in younger women or…?

Well it… like breast cancer generally, it gets, um, the risk gets higher as you get older.

Oh, right.

But also as I said with DCIS in particularly because it's found through screening, it will typically be found in the age group that's having screening.

Right.

So, so typically from about age 50, um, women in their 50's and 60's are…

Right.

… the ones having the most screening. Um, but yeah, you can still get it later.

And what if you're a younger woman like below 50? Do they, should, should still have mammograms often or not?

Well the, the recommendation is to start from aged 50.

Because that's when a lot of… younger people, you know, to have breast cancer… well, not a lot, haven't known one personally but have, have heard about it, you know?

Yeah, so it can happen in younger women.

Yes, yes.

Um, but the… the screening is, is more effective as you get older actually.

Ok.

I had, I had the impression from my GP that when you get to like an age like mine, 74 years old, that your risk is reduced. Is that incorrect?

Um, so your, your natural chance of getting breast cancer is not reduced, um, but there are other reasons that we might talk about…

Ok.

… that you might not, um, necessarily keep having screening. But, yeah, it doesn't, doesn't… the risk of getting cancer doesn't actually go down as such.

What about being told that, um, if you have a history of breast cancer, especially in an (?) family you should start having tests quite early, especially if your grandfather died at 50? Um, and I was astonished to see these new ads on the telly that say 9 out of 10 people it's got nothing to do with your family history. I could be the only one. So that seems to knock on the head everything we've been told for decades and I'd hate to see women who have got a history of breast cancer being put off by that. Oh, it doesn't matter, I'm all right. So what, what do

you think, do you think we should still be telling our grandchildren to be having it done from… odd ones from 35 or 30 or…?

Yeah, so if you talk to your doctor about your family history then that does, um, influence, you may get different recommendations, such as… 'cause if you have a stronger family history, um, you may be recommended to start earlier. Um, or have, you know, more frequent screening, or some different…

You know, now that you hear on these, these programs of young women having double mastectomies because the history is so strong…

Yes, yes.

… now that they will probably get it. That sounds very…

I don't think that's just history though, is it? That's that BCA or whatever… the, the gene.

Yeah, so there's a small proportion of the population who have one of the genes, a mutation in a gene that makes it very, very likely that they'll get breast cancer, um… and that's, as I said, a small number of people. Um, and then there's some people who have a bit of a family history but not necessarily any special genes. And then there's most people, who don't really have a strong, um, history and that's the, the general population, that kind of average…

Can I say something else? Apparently, and correct me if I'm wrong, if there's prostate cancer in the family, that is also a precursor to a possible breast cancer.

Yeah, I've heard that.

I'm not sure, um, yeah, I think I have come across that as well, um, breast and ovarian cancer they tend to link a lot. Um, I'm not quite sure about prostate cancer. But prostate cancer is…

Yeah, I've heard that as well.

I think it is because my, yeah, my brother's been diagnosed with prostate and only because they checked him because of the family history of breast cancer. So

I think there is a strong link.

And the other way around as well?

I'm not sure.

Ok, any other questions or thoughts sort of at this point? Ok. So I'm going to talk a little bit about, um, how DCIS is treated. So the goal of treating DCIS is to prevent the abnormal cells from turning into invasive cancer. And overall it seems that our current treatments are doing a pretty good job of achieving this goal.

Now surgery is really the primary form of treatment for DCIS. So that's doing an operation on the breast, in hospital, under general anaesthetic.

And doing surgery means that the pathologist gets another chance to look at these cells under the microscope, but this time they've got more to work with because they've got what's hopefully the whole area of the breast that's affected by DCIS, rather than just the small bit that was taken from the biopsy. And in most cases this will confirm that it really is just DCIS as the biopsy suggested. But in some cases they find after surgery that the patient actually has invasive breast cancer that the biopsy didn't catch. So that patient's diagnosis is changed or upgraded at that point.

Now there are different types of surgery a patient can have, and surgery may also be combined with another form of treatment. And exactly what each patient has will depend on features of her DCIS (such as it's size and grade – which I'll talk about a bit later) as well as characteristics of the woman herself (like her age, her overall health, and her preferences).

So I'm going to tell you in a bit more detail about the 2 most common options for treating DCIS. And both of these approaches are considered very effective and survival rates for women with DCIS are excellent.

Can I just ask a question? With the DCIS cancer and the invasive cancer, is there a difference in the cells?

So it's… it's a matter of whether the cells are inside the duct…

Inside or outside.

... or outside. Yep.

So treatment for DCIS most commonly involves breast conserving surgery and then radiotherapy. This means having an operation to remove the part of the breast affected by DCIS and a small area of healthy breast tissue surrounding that. And this may also be called a lumpectomy or wide local excision.

And after breast conserving surgery a pathologist will look at the breast tissue that was removed. And sometimes, if they find there's a bit more disease than they originally thought, the woman may need more surgery.

But everyone responds differently to surgery. Some side effects happen to most people, others happen only occasionally. And some will happen straight after surgery, and others can take longer to develop. But some of the common side effects include things like pain, numbness, and bruising around the wound.

And after this surgery, the patient has radiotherapy, normally every day for a few weeks. Radiotherapy uses x-rays to destroy any abnormal cells that may be left in the breast.

Receiving radiotherapy is painless. But women may experience some side effects. During the radiotherapy treatment, the skin of the breast can become red and dry, a bit like sunburn, um, or the skin can become darker; and women often feel more tired than usual. And these side effects usually settle down after treatment finishes and things get back to normal.

During breast radiotherapy, it's possible that a small dose of radiation may reach the heart or lungs, depending exactly where the DCIS is. And some studies have suggested that women who had breast radiotherapy in the past may have a slightly increased chance of having a heart problem, like a heart attack, later in life. So doctors these days are using more modern techniques when giving radiotherapy to try and minimise this problem as much as they can.

Now instead of having breast conserving surgery and radiotherapy, sometimes a mastectomy may be recommended, for example if the area of DCIS is quite large compared to the total size of the breast or for some

other reason. This is a bigger operation, which means there's a higher chance of complications and a longer recovery time.

So all the side effects that I mentioned for breast conserving surgery are also relevant for mastectomy but because this surgery is more extensive, there may be additional side effects such as stiffness in the arm or shoulder.

And obviously mastectomy affects the body shape and it can affect women's body image or how they feel about their body. Many women have the possibility to consider breast reconstruction surgery to try and help with that.

And just in case you're wondering at this point I just want to mention that chemotherapy is not a treatment that's typically used for DCIS. So the main treatments are, as I've outlined: different types of surgery and radiotherapy.

So what are your thoughts and feelings about that information about treatment for DCIS?

Um, could you have it and it never spreads?

Well, that's a good question.

Yes.

Um, basically at this point its, its hard to answer that.

Mmm, yeah, because it's a bit of a… you know, you're going to go through a lot maybe for nothing, you know.

Ok. What are other people's views about that?

Would, would they ever be able to find out for sure whether it's going to spread or not…

Well…

… down the track?

… they're working on it.

Yeah?

At this point, um, as I said, it's, it's hard to say. So I'll talk a little bit more about it but… at this point it's treated on the assumption that it…

It might.

… it might.

And are you going to talk about the causes of it later on, or can I just ask about the causes because they say in Mosman, like in suburbs like Mosman you're more likely to get it because it's a lifestyle disease, breast cancer.

What?

Is that the same for that one?

So the same kind of risk factors are involved with DCIS and when they say, when they talk about lifestyle, um, it's because we know things like, um… um, so having children early in life can reduce your risk of breast cancer. Um, and we know that as time goes on and as women get more educated and more involved in the workforce they're having children later in life. Um, so that's kind of a lifestyle thing. And also things like, um, gaining a lot of weight in your, in middle age, um, can increase your risk. Drinking alcohol can increase your risk.

Smoking?

Smoking, um, it's a bit unclear. It's not as, it's not, er, a clearer link as there is between smoking and many other cancers.

And is it only certain foods because some foods make you larger or is it certain foods like red meat or other things, um, you're more inclined to get it?

I don't think there's certain foods that are linked to breast cancer. It's more…

It's not a dietary thing?

… your, your weight and being physically active more generally, yeah. Ok, what, what do others think about, um, I mean if you're trying to imagine hypothetically if you had just been diagnosed with DCIS and you'd been told about these treatment options, how do you think you'd be feeling?

Hopeful.

[LOTS OF MMM's and YEAH's]

Hopeful.

I'd go for the treatment.

I'd go for the full mastectomy. Only knowing what I know.

[A FEW YEAH's]

Because that's… my daughter was diagnosed and once they did the mastectomy they found a rogue cell.

Mmm.

So there was no radiation. It was full chemo.

Mmm hmm.

How did they know with the, um, biopsy whether it's DCIS or whether it's invasive? Do they have to do two biopsies; one inside where they think it is and one further over? I mean how do they know it's one or the other?

Well, they, so what they take out for the biopsy will include, um, cells that are inside…

Mmm.

… and cells that are outside the duct.

They include both.

Yeah.

Oh right.

So they, they try to, they try and capture both, um, and make a judgment based on that. But as I said, um, sometimes when they do take out more tissue they find that the biopsy wasn't quite capturing everything that was there.

Mmm. And they take the biopsy from the duct itself?

I mean I think they, they try and, um…

Or as close as?

… yeah, so they can see from the mammogram, um… you know, they'll look at where it is. They may well, in the course of that investigation, also do ultrasound and other imagining to try and zero in on the location. Um… and they, yeah, they pick the best spot they can to take the biopsy.

They don't seem to be able to tell fully that it's escaped until they've actually done the surgery. That's my understanding.

Mmm, so sometimes they can see clearly that it has gone outside the duct. Um, and sometimes, as I said, that's… yeah, it becomes more accurate when they have more tissue that's been taken out.

Ok. Um…

Can I just... ask a question, sorry? Someone made, both of you said it made you feel hopeful.

Yeah.

And I just thought maybe you would be able to explain a bit more about what you meant by hopeful.

Well if you have the DCIS there are treatments, you know, and I think that… it, it gives you some hope. It gives you some hope. I have a cousin that passed away 40 years ago and she had a breast cancer. She had, um, she had to have a mastectomy, but then it just… back then they didn't have the technology and the knowhow they've got now. With her it spread everywhere. But I think there's hope. If there's some sort of treatment, there's hope. Yeah.

I agree with that and I find I increasingly know people who have breast cancer, who have surgery or whatever treatment and it never returns.

Yeah, me too.

Ok. What about the… the sort of quiet area around there?

[LAUGHTER]

I think I would have the treatment. It, it depends I think on your age, um… and whether you have another ongoing illness or something like that. But, um, no, I have had a friend who had radiotherapy and she ended up with a spot on the lung from the treatment. Um, which she hadn't been told about earlier, um, but she's still ok. And I've had another friend who's gone 20-odd years and, um, no problems after she had a mastectomy.

Mmm, yeah, well quite often medical treatments can cause, you know, some other illness to happen. For example, in my own case taking this blood thinning medication, which is necessary because I've got peripheral vascular disease and minor cardiac, um, disease, um, but that caused my esophagus to be wrecked and so now I have a lot of trouble eating. It's not related to breast cancer but…

No… yeah.

… it's treatments.

[A FEW MMM's]

So when you were saying it would depend on your age and…

Yes, and whether you had ongoing issues. Yes.

So if you did have other ongoing major health issues how would that potentially effect…

Oh, well I think you could decide that you didn't want the treatment, um, you know, just take your chances and… I've got friends who, you know, have chronic illnesses and they really just wish they could go.

Mmm.

But, you know, they're in their 90's and they've had enough.

[A FEW MMM's]

I think until you walk in their shoes, um, you really don't know what you'd do.

Mmm. How's everyone doing at this point? Do you need a couple of minutes little break to… stretch your legs?

[LOTS OF NO's]

It's very interesting.

Yeah, it's really good.

Ok. All right. So… I want to move on to a, a question that's currently occupying DCIS, um, and that's a question of could some women with DCIS safely avoid treatment? So our current treatment for DCIS is based on our best scientific knowledge and of course wanting to do what's best for patients.

Um, and as I said before, survival rates for DCIS are excellent. But many patients do experience side effects from treatment and this includes both the physical effects on women's bodies, and emotional or psychological effects as well, and they affect women in the short term and the long term.

And so… just coming back to, um, this issue that we've kind of just raised, um, particularly about DCIS as women get older. The older women get, the more likely they are to be living with, um, various other health problems. And DCIS is a condition that may just stay the same or progress very slowly over many years (or even decades). So for women who are already older when their DCIS is diagnosed, it may not actually affect their health in their remaining lifetime because of other health problems that are more important for them.

And another issue here is that older women may be more affected by the side effects from DCIS treatments, such as complications from surgery or heart problems linked to radiotherapy for example.

So do you feel you understand that information?

[LOTS OF MMM's and YES's]

So what are your thoughts and feelings about DCIS in older women in particular?

Well I think if I was diagnosed and I'm in my 80's, maybe I, I don't think I would want to have any treatment. Hopefully it would just stay as it is and not effect me (laughs) for the rest of my life. You don't know, do you?

Mmm.

Yeah, I, I'm with you. I'm with you.

I mean, fancy having radiation or chemotherapy or… not you have that there, but when I'm older…

I'd think I'd try everything to keep and stay alive.

Yeah. I'd want to stay alive but…

I'd try the treatment with the radiation.

As long as it didn't effect your health and that I think the way things are, the, the research is going I think the more, as the years go on it's going to get easier and easier and easier for them to contain all these, the radiation therapy and all the rest of it. And I think you've got more chance of not getting complications by the time, you know, if you get into your 80's, by then hopefully it'll be a breeze (laughs).

And I think it all depends on all your other health…

[LOTS OF YEAH's]

… your health problems. You know, if you want to put yourself through that with all your other health problems. Yeah.

Mmm.

Also statistically because the cell growth slows down so much, doesn't that relate back to the cancer, it doesn't grow as quickly?

We, yeah, we think it may be the case that, um, as you get older the, the rate of progress gets slower.

Yes.

Yeah. Again it's, it's a bit hard to say for sure.

Mmm, so the younger you are and more drastic possibly the treatment to accept, the older you are… it'd be better not to. I don't know. You'd have to weigh it up at the time.

Mmm hmm.

When I go back to, um, prostate cancer, my brother was told when he was about 75 that if he ate right and exercised and ate the super foods all the time, he'd probably have about 5 years and they wouldn't bother doing any treatment. Well that was about 17 years ago. He's 87 now and he's just been on a trip with his granddaughter all around America.

Mmm. And did he have surgery?

Nothing.

Wow.

They said eat right, exercise, and…

Yeah.

… and it's certainly worked so far, so maybe that's the same for breast cancer. Live right and it's a help.

[A FEW MMM's]

Has it anything to do with exercise, did you say that? I think you did.

Yeah, yeah, being, being active is, is considered pretty good for most things and, yeah, breast cancer is no exception.

Ok. So, yeah, a few people, um, feeling like it, you might feel differently based on if you are older and less healthy…

Yeah.

… and some other people saying go for everything you, you could. Yeah?

And I think it would depend on your medical advice as well.

[A FEW YEAH's]

If you trust your physician and they've always been very sensible. If they say oh, you should do this or you shouldn't do that, you're more likely to go with them.

Mmm hmm.

If you were a bit bewildered it's almost like a stab in the dark. And, um… thank god for the medical profession.

Yes.

Yeah, but I think there are doctors and doctors. Some doctors make big mistakes.

Yeah, you'd have to have confidence.

There are doctors and doctors.

You have to have confidence.

And lot of doctors wont put themselves forward to say anything in case they're wrong.

That's true.

[A FEW MMM's]

Also… that's what I…

I think I'd be getting a second opinion…

Yeah.

[A FEW YEAH's]

… because…

You can compare.

You're exactly right. That's exactly right.

And you might have to get a third one (laughs) because they might leave you hanging, yeah.

[LAUGHTER]

Yep.

I don't know. You don't know, you don't know until…

Yeah, I agree with more than one opinion, yeah.

Um, so… as I said, um, breast cancer experts now actually doing research to try and identify women with DCIS who have a low chance of getting invasive breast cancer so that in the future, women like this may be able to avoid surgery and other treatments and therefore avoid all those problems that can come with treatment, while still having excellent survival rates.

And this kind of development may be of particular interest to older women, because of all the things we've just talked about. Now as I said before, it's very difficult to reliably tell who these 'lower risk' women are, but we do have a few clues. And one of them is something I mentioned earlier called grade.

So after the biopsy is taken, the pathologist looks at the sample under the microscope and gives each case of DCIS a grade based on how the cells look, and that gives an idea of how fast the cells are likely to grow.

Low grade indicates that the cells appear to have a low level of activity, which suggests they may stay like that for quite a long time. On the other hand, high grade DCIS means the cells appear more active, so they're likely to grow at a faster rate. So it's considered important to treat high grade DCIS because otherwise it's pretty likely to turn into invasive breast cancer at some point. And then there's also an intermediate grade which is somewhere in the middle.

Now you might be wondering, if virtually all women with DCIS are having

treatment, so having surgery to take the DCIS out, why am I saying that we think low grade DCIS may stay as it is for many years and not cause problems, even without treatment.

Well, we think that may be the case based on a small number of research studies similar to this one.

So as an example I want to tell you a bit about this study that was done in America using a database of routinely collected information about cases of DCIS. These blue bars represent all the patients in that database who had low grade DCIS. So the vast majority of these patients in the database, about 9000 women, had surgery as treatment, um, which is the typical thing to do for DCIS, that's recommended in all the guidelines for doctors.

But a small number of patients, a couple of hundred, didn't have surgery for some reason, and we don't really know why not.

So this database kept track of key events that happened to these patients over many years, for example it recorded if any patients died from breast cancer. And what the researchers did for this study was compare the large group of women who had surgery and the small group of women who didn't, after about 10 years. And they found that the women who did have treatment had a very high survival rate, so 99% of them had not died from breast cancer, so they were doing really well. And the research also found that among the women who didn't have treatment, 99% of them also had not died of breast cancer.

So this led the researchers to suggest that there may be a subgroup of women with DCIS who are likely to have really good health outcomes over time, whether they do or don't have the typical treatment of immediate surgery.

So this idea of a subgroup of DCIS that we could call 'low risk DCIS' is a big focus of new research that is designed to try and investigate whether women in that category could possibly avoid treatment.

And there's a range of factors that are relevant to trying to define this category of DCIS – grade is an important one but there's lots of other factors that are taken into account, which I won't go into now.

But just coming back to this little diagram I showed you before I just want to give you a rough idea of how many women with DCIS might be classified into this lower risk category. So of our 16 women with DCIS from before... we think about 5 women have what could be classified as lower risk DCIS based on their biopsy.

For the other 11 they're considered to have a higher chance of getting invasive breast cancer at some point so it's still considered more appropriate that these women do have treatment.

But these 'lower risk' women are the focus of current research looking at whether they might be able to avoid treatment. And I'm going to tell you a bit more about that research now. And it's just important to remember that we're talking about quite a small and select group with DCIS where researchers are considering a new, different approach to managing their condition.

So the type of studies I want to talk about now are called clinical trials, so let me explain what they are.

A clinical trial is a research study that asks an important, specific question about a certain health condition. Clinical trials often test new ways to manage a condition, by comparing a newer approach with whatever is the current standard treatment.

And trials are designed to find out whether patient outcomes (like health and quality of life) are similar for both options, or whether one option is better than the other.

Now if you think back to that study I talked about before, with the blue bar graph, it was done by looking at a database containing routinely collected basic information about past patients. And although studies like that can be valuable for giving us interesting ideas, it's hard to draw firm conclusions from that kind of research because there's a lot of important information missing, for example details about those patients who unusually didn't have surgery and why.

Whereas clinical trials are specially designed to overcome the limitations of other types of studies. So that's why trials are considered to give us the

highest quality of evidence.

Now this illustrates the basic design of a simple clinical trial. Sometimes they can be more complicated, for example with more than 2 groups, but most typically, each patient in the trial is put into 1 of 2 groups. One group of patients gets the usual standard treatment, and the other group gets a new treatment or a new way of managing their condition. And which group each patient goes into is determined by chance. This process is called randomisation and it's a very important part of clinical trials. When we compare 2 groups of patients having their condition treated or managed in different ways, we want to make sure the groups are very much the same except for the way their condition is treated or managed. If patients and their doctors choose which option to have, then the patients in the different groups are likely to be different from each other in important ways relating to their health, which can create a bias that makes the results less, um, less reliable. So patients who volunteer to take part in a clinical trial need to be comfortable with ending up in either one of the groups because they don't get to choose which group they're in. Randomisation makes sure that the study groups are as similar as possible. And that way, we can get the most reliable information from the results of the trial to learn about the effects of those different ways of managing the condition.

Now deciding by chance how a patient will be treated may seem strange, but it's considered an ethical thing to do in situations where we genuinely don't know whether one option is really better than the other.

So there are currently 3 clinical trials going on around the world for women with low risk DCIS: one in the UK, one in Europe based in the Netherlands, and one in the US. They all just started in the last couple of years, and together they will involve over 3000 women. Now there are slight differences between the trials, but they all have the same general design.

And their aim is to learn whether women with low risk DCIS could safely avoid having treatments that may not be necessary for them.

So the women who are invited to take part in these trials have to meet strict criteria to be considered suitable. They have to have low or

intermediate grade DCIS (not high grade), they can't have a lump you can feel, plus there's lot of other more technical rules that I won't go into. So remember, this is what typically happens for women with low risk DCIS: they have treatment pretty much straight away, um, and we talked about those treatments before, so surgery of some king with or without radiotherapy.

And of course in the following months and years, if it seems their initial treatment hasn't worked quite as well as hoped, or their DCIS comes back, or they get DCIS elsewhere or invasive breast cancer, they might end up having some further treatment down the track if needed.

So that's the normal scenario for pretty much all the women in places like Australia where there is no clinical trial running. For women who do have a trial available where they live, if they're suitable to offer the trial, they may just say no thank you, I don't want to go in the trial, I just want to have the usual treatment. Or, if they decide to join the trial... then they get randomised, so allocated by chance, to either a usual treatment group or the active monitoring group. So the usual treatment group of course has usual treatment. And the other group has a different approach where in the first instance they don't have surgery or radiotherapy. And then patients in both groups are monitored closely, so they all have mammograms at least every year. And importantly, patients in the monitoring group may have treatment later if there's any sign that their DCIS is progressing, and we expect that to happen for some women. So this approach may be called active monitoring or active surveillance and it's actually already an accepted option for men with early prostate cancer because trials similar to this have shown that it can be a safe option for them. So these trials in DCIS will test out the idea that this monitoring approach where you only give treatment if it's needed may result in the same very high survival rates as standard immediate treatment, but with fewer side effects overall because some women will avoid treatment altogether, and others will end up having treatment down the track but it's delayed so they may have a quite a few years without treatment and without any of those side effects. So those are potential advantages of monitoring.

There's also potential disadvantages that the trials will examine. For

example, patients who don't have immediate treatment may be more likely to get invasive breast cancer – although with the monitoring of course it's still likely to be picked up at an early stage. As I mentioned before, it's also possible that some women who are diagnosed with DCIS based on the biopsy actually have invasive cancer at the time that the biopsy didn't pick up. And we also don't really know how women having monitoring will feel about not having treatment, so in the trials the patients will complete questionnaires to help the researchers understand the psychological effects of this new monitoring approach, and that's a very important aspect of the research as well.

Now because there's only quite a small and select group of women who are eligible for these trials, and because they then need to be followed up for quite some time, it will still be some years before we have any results from this research.

In the meantime, it's possible that a similar trial could be started locally in Australia and New Zealand, so women here might one day have the possibility to enter such a trial.

But otherwise until the results of these trials come out, women with DCIS will continue to have treatment as normal because we don't yet have strong research evidence to prove that any other approach, like monitoring, is really safe.

So what are your thoughts and feelings about these trials?

It's gone out of my head.

[LAUGHTER]

What, what age group are these trials conduct… how old are the women that they're doing these trials on?

So they're, they're from about 40 or 45 up.

So they could be in their 80's? They could be… yeah.

Yeah.

So a ten year trial probably?

Yep.

So you've got to have low risk to be eligible?

That's right.

Yeah, well I'd go for the monitoring.

Ok.

Yeah.

So if you…

But I probably wouldn't last 10 years (laughs).

[LAUGHTER]

In going to the trial that means that you'll be, um, allocated by chance…

Yeah.

… to either monitoring or…

Yeah, if it's low-risk I would.

Mmm... (UNCLEAR)…

But while you're still being monitored if, if your condition becomes worse…

Yeah.

… then they know about it. Something can be done.

Yeah.

[A FEW MMM's]

Sounds like the best of all worlds really.

Mmm.

Yeah? Ok, anyone feel differently?

Yeah (laughs).

Yeah?

Not for older women, I think 60, 65 plus, very good idea. I think it's high-risk for younger women.

Yes.

I don't know that 45… mmm, if I was 45 I wouldn't. The age I am now, yes, I'd say monitoring.

Mmm hmm. Ok. What about, what about you Elizabeth?

Well I think monitoring is a good idea, yes. I would certainly… if it was me I would like…

I think if you were in the trial they would be watching you really, really closely, so…

Mmm, so whichever group you get into, really doesn't really matter, because even if you're in the monitoring and your condition becomes worse you can do something about it.

[LOTS OF MMM's and YEAH's]

Mmm hmm.

And the other thing is that it's a 10-year trial. In that 10 years advances are going to be made.

That's right.

[LOTS OF YES's]

So if you're 45 at the time…

Yes.

… and you finish when you're 55, the condition might be more manageable than

when you were 45.

Yeah, that's true.

And your ideas might change if you are actually diagnosed.

[LOT SOF YEAH's]

Yeah, it's a different thing, yes.

[TALKING TOGETHER]

… we can philosophise about it.

Yes, you don't know what you would do. Mmm.

Exactly, mmm.

You don't know, do you?

Depends on the diagnosis, doesn't?

Mmm.

Sorry?

It depends on the diagnosis.

Yes, exactly.

I think it all depends on that.

Yeah, exactly.

I find I hear a lot more younger people being diagnosed with breast cancer these days than…

Yes, me too.

… than older people.

Well, we're getting more and more affluent, aren't we?

Mmm. Maybe.

Young people are getting more affluent…

Yes, maybe.

… and having babies later.

… getting tested but, you know, so many girls and they're in their 30's, 35… 29, and…

It's not only girls. It's men and boys as well.

Mmm, yeah.

Which is…

Yeah, I've just had a male friend had a mastectomy, so… but then he's, he's very into the good life. Big and boozy and football…

[LAUGHTER]

Ok, what about you Gill?

I've, I've just had a friend loose her daughter at 49 last week with breast cancer.

[LOTS OF MMM's]

Um, you just don't think you'll loose your child like that.

Mmm, that's right.

I mean she had had it for a while but, um… yes, I, I just… as I said before it just depends on the circumstances at the time, if you were diagnosed. And the age…

Mmm hmm.

… too, but I think it's a wonderful idea. How long have they been doing the, um, trials?

Well the first one started in 2014.

Oh.

And the other two just started last year.

Right, so they've got a while to…

Yeah.

[LOTS OF MMM's]

It'd be interesting to hear in 5 years time as to what, how they're all travelling.

[LOTS OF MMM's]

And if you did decide to do it here, how would you, how would you select people? You'd get them through the mammogram testing people?

Yeah, that's the main way that, that the diagnosis happens, so… so, um… so… I think the general feeling is that you would be, you would consider going into a trial?

Mmm.

So how do you feel about that, that idea that, um, then what happens to you is determine by kind of flipping a coin sort of out of your hands?

It is ethical, isn't it? It's a very, very good way of doing it.

So you think it's…

I do, mmm.

… it's a good way of doing it?

I do, yeah.

Yeah, especially if you're still being monitored and…

[A FEW MMM's]

… if it progresses then you, you know, you'd be out of that group and receiving treatment, wouldn't you, so, you know.

But wouldn't you know pretty quickly anyway if you have a breast operation you know which group you're in (laughs).

Yeah, yeah, you do know.

It's not a secret.

It's not a secret, that's right.

[A FEW MMM's]

Not always, you have to have the full operation sometimes to know how bad it is.

No, I'm not saying that. You'd know which group you were in. You'd know whether you're in the monitoring group or the surgery group.

[LOTS OF YEAH's]

Oh I see, once you've been selected. Yes, right.

So… if you, um, agreed to go into the trial then yes, you would be randomised to one group or another and you would be told immediately. Do you think, um, you'd be kind of hoping for one versus the other? Or do you think you'd…

Mmm, I would (laughs).

Yeah, what would you be hoping for?

The one that was monitored.

Yes, I'd think I'd rather than, yeah.

[A FEW MMM's]

And do you feel the, so if they're monitored, if their appointments are once a year having mammograms, does that feel like, um…

Enough.

… enough? Do you feel…

I was thinking that. Perhaps every six months?

Yes, me too.

I know, you know, having mammograms you can get problems later on, but, er, yeah, I, I just feel 12 months might be a bit too long.

Mmm hmm.

That's a high exposure to radiation and…

[A FEW MMM's]

… so I think…

So the mammograms are radiation are they?

Oh, absolutely.

I didn't know that.

They say it's low radiation but it's still every 6 months that's…

Yeah, well I didn't know it was radiation.

But x-rays are radiation too. All x-rays.

Yeah.

Even when you have your teeth done the doctor, the dentist whizzes into…

[TALKING TOGETHER AND LAUGHTER]

Yep, we're exposed to it a lot, a lot. And when you're flying in airplanes it's radiation. So…

Really?

Yep.

Ok, um… and so what about, um… can you think of other, I mean… you've had some thoughts about this already but other people you know or other people in other circumstances who might feel differently. Like can you imagine some people who might say, I don't want to go into this research, I just want to have…

Absolutely.

Yes.

[LOTS OF YES's]

What's, what's different about them? Is it just a…

There are just people who are terrified of anything, won't go to the doctor incase they're diagnosed with something. So they wouldn't take part in a trial.

Mmm hmm.

I don't think. They keep away from doctors.

Mmm hmm.

And then a, a lot of people it interferes with their lifestyle.

That's true.

You know, running to doctors and tests and…

Yeah.

… I have a friend who has breast cancer and she's a funny lady. She's a funny lady but, er, you know, you see her, I'm not going back, I'm not going there any more, you know? She'd rather… interferes with her lifestyle.

Mmm hmm.

Yeah.

I, I've known two people with breast cancer who decided they wanted surgery. They, they weren't going to muck around with any other kind of treatment. Just go for it.

[A FEW MMM's]

And they both had good outcomes.

[A FEW MMM's]

And I think that most people you hear of, or a lot I hear of, as soon as they're diagnosed they want to go and have their surgery and…

[A FEW YEAH's]

… you know, and everything done that's out there to do.

'Cause cancer's scary.

Yes, yes. If you're diagnosed, very scary.

[LOTS OF AGREEMENT]

And nobody wants to die.

[LOTS OF NO's]

Can't live forever but…

Life is precious and you do whatever you can…

… exactly.

… to keep going.

And cancer seems a very unpleasant way to die too.

Very unpleasant and breast cancer the, the secondary's are what is really unpleasant.

But the breast cancer is different than the DCIS in that, because if you've got definitely in, have it treated. With the other, if you thought it might never progress, or if the doctor tells you it might never, you'd go for the trial. I would.

Mmm hmm.

But not everybody will. I have a nursing sister friend…

No, of course not. Yes.

… who was diagnosed low-risk DCIS…

Yes.

… one breast and she had a double mastectomy.

Right.

[A FEW MMM's and YEAH's]

No reconstruction and she's happy.

[A FEW MMM's and YEAH's]

And she said if she hadn't had it done…

Yeah.

… she would have been a nervous wreck so…

[A FEW MMM's]

… so it does depend on the individual.

Yes, it does. Yes.

Yes.

What about the, um, problem with going, you know, with radiotherapy going into heart problems? I have, my eldest daughter…

Yeah.

… had (SCAD?)… do you know what SCAD is?

No.

Spontaneous Coronary Artery Dissection, which is a tear in the aorta. Now she was under a top cardio… um, cardiologist and she was the third only ever surviving patient of his, and she was under a trial, er, like, um, a chest case. So she developed something like that… she'd have to weigh up take, having radio 'cause it could effect her heart.

Yeah, yeah.

She's on medication for the rest of her life and she was only 35 at the time when this was diagnosed.

God.

So, something like that, you just don't know. Normally I would have said, yes,

she'd go in for surgery but it might be more invasive because of her heart problem.

Yes, it's all individual.

And it'd really… yeah, that'd be really scary.

Mmm.

Yeah, the heart thing would worry me too.

Yeah.

Ok. Um, yeah, I'm just quite curious about, er, if you kind of imagine that let's say, and we don't really know much about how these trials are going in detail, they're still going, but if for example they found they were inviting women to go in the trial and women were just saying no, you know, I'm, I don't like the idea of that, I just want to have treatment. Um, what is it that, what is it that makes you all feel differently? You all seem quite positive…

Now are we talking about the DCIS or cancer?

Yeah, just with DCIS.

Yeah.

Low-risk DCIS.

No, I'd have the trial.

I think it would be the diagnosis for me.

Mmm hmm.

I'd have to have a second opinion and trust the diagnosis.

[A FEW MMM's]

Mmm hmm.

And if it was low-risk… otherwise (laughs)…

Yes, it'd have to be low-risk, yes.

And do men get this DCIS or do they just get breast cancer?

They can very, very rarely get DCIS.

But if you did have a diagnosis and you had a man with a diagnosis it would be good to include them as well, wouldn't it? Not just women.

Yeah, I, I don't think…

A small subgroup.

Yeah.

There's not enough of them?

I think there's probably just not enough of them.

[LOTS OF MMM's]

Well I don't know how you find out that men have had it 'cause I don't know a lot of men who go for mammograms. 'Cause you don't...

Exactly.

… unless they're very fat, you can't…

Yeah.

… I don't know… I must ask my friend how he was diagnosed.

[LAUGHTER]

Oh yeah, it's true.

I don't think the percentage is all that high though for men.

I don't know. See it's more than you think.

Yes.

[A FEW MMM's]

Apparently.

Ok.

I thought there might be a man here today…

[LAUGHTER]

… you know, because they do get it.

Yeah.

You know, breast cancer.

No, we're, we're women only, um, research…

[LAUGHTER]

… at this point.

It's quite sexist.

They'd probably be too embarrassed (laughs).

[LAUGHTER]

I should imagine.

Well my friend would be in it… (?) he'd be in, especially in… (UNCLEAR)…

Mmm.

Mmm.

Ok, um… and… is there anything else, er, features about the research or, um… er, information that I've said that is particularly important to you in, in kind of feeling comfortable or not about going into a trial versus having standard treatment? Or anything, you know, if you were, if you were asked to go in a trial are there other people you would want to talk to or other information you would want to know before you made that decision?

You'd have to go to your GP at least and have a chat with them, and your family.

Mmm hmm.

I think.

I'd have to make up my mind whether I wanted treatment or not. And if it didn't matter then you could go into a trial. But if I really didn't want treatment, like the surgery and that, I would go for the other and I wouldn't' go into a trial.

Mmm hmm.

Because, you know, you might, you might be looking down one avenue and then you have to turn round and you're on the other side and that I would like to make my own decision first.

Mmm hmm.

Um… if it didn't matter, if it didn't, if I wasn't too sure which way I'd go, I'd probably go into a trial and let them decide. But I'd, I'd have to, I'd have to weigh everything, yeah.

Wouldn't you ask the people in a survey, if you were… if this happened to you and you were diagnosed, dah dah dah dah dah, you were given the choice of this, which would you pick? And if there was 97% of that and 15% of this, wouldn't you let that 15% have theirs and then ask those to change over? Say there's a chance that you can be in either, if there was only a few who said, oh, I've got to have the surgery, you'd just let them do it, wouldn't you? And split the other people up that didn't care.

Well, I mean certainly if people want to, really want to have surgery, you would, they would do that. But the idea with the trial is that you go into it being willing to have either.

[A FEW MMM's AND YEAH's]

Yeah, um, because… if you start comparing people who choose one thing and people who choose another then there's all kinds of factors that might be coming into play. So even though it might be that they have different personalities and that's why they've chosen one thing, it could be that that somehow affects how they do as well. And that's why a trial is designed to get rid of those differences by having the process of randomisation.

And the trial could come up with something better than what's been used at the…

you know, the treatment itself, there's always a chance that the trial will come up with something better.

Well, the trial is testing out the, the approach of, um, monitoring and treating later if needed.

Right.

And seeing whether that approach overall is better than…

Right, yeah.

… the current approach of treating immediately.

So, so it's getting the results of, of one group versus the other and… to get the results.

Yeah.

Mmm.

Yeah.

So it's looking at, um… you know, whether they get invasive breast cancer, you know, whether they die…

Yeah.

… um, but also what kind, their quality of life, um, symptoms that they experience, um, how they're feeling, you know, whether they're anxious or worried or… um… needing to have time off work and all those kinds of things…

Mmm.

… they'll ask those patients a lot of questions and gather as much information as they can to, to give a full picture of, um, their health and quality of life outcomes of those two approaches.

I think you have to be pretty brave to go into a trial, quite honestly (laughs).

[A FEW MMM's]

That's right.

Yeah.

What do you mean?

Well, because there's still a risk. So… and depending on your age if survival was… your biggest criteria, which I think for most of us it is…

Mmm.

… probably want to be treated.

Mmm hmm.

Get rid of the cancer.

Couldn't they still do a trial of not having, and not tossing up a coin to see which area, but still do a trial on the ones that have had, and ones that haven't had the surgery?

Mmm.

I mean you'd still get much the same results?

Yeah, instead of having… so you could still choose…

Yeah.

… what you wanted to do and take people from each group. I think, yes…

[A FEW YEAH's]

… rather than randomly being told…

Yeah.

… well you're going to have this.

[A FEW MMM's]

'Cause there must be a vast majority of people on the low risk end that would choose not to have anything done.

To me that's more sensible to have a trial done where the people have made their own choice.

Yes.

And you would still…

(UNCLEAR)

… yeah, and would still get the, and still be monitoring it. Yeah.

[A FEW YEAH's]

So that's, that's interesting that you'd say that. Basically the situation is that… the medical community, so the, um, doctors that you would be consulting, don't really feel very comfortable with not treating DCIS at this point, because they would want to wait for the results from this kind of research to make them feel confident that they can say, it, you know, you'll probably be ok if you don't have treatment. So, so… for, for the medical community as a whole, if there's trial available they will, um… you know, be willing to offer women the option of going into a trial where they know they're going to be monitored really closely, um, and, you know, everything will be kept a close eye on. Whereas, um… we know that they're not very, um, likely to say to you that you can not have treatment if you don't want.

Mmm.

Of course some people don't have treatment, but it tends to be only if they're really considered unsuitable to have surgery because they're so unwell or so elderly. So…

So isn't that a good thing then? If the medical profession thinks it's better to have treatment…

I think they take it.

… if, if they're evenly split and think it's a brilliant idea. But not just risking people's lives. I don't know how many thousand you'd do. If the medical profession thinks it's better to operate, why would you put the other group at risk? Just for a research…

Well, um, I guess what I'm saying is, yeah, um, that they… might think that, you know, in theory it's very important to have this research and that there will be a group that don't need the treatment, who are better off without. But for an individual doctor with an individual patient…

Yeah.

… that's…

Yeah.

… that's… it's difficult for doctors to do something that's atypical…

Risks the life of that one person…

[A FEW MMM's]

… their patient, yeah.

… other than in a research study.

I suppose they take an oath…

Yeah.

… to fix as many people as they can (laughs)… the Hippocratic oath, isn't it?

Mmm hmm.

What were the numbers of the other countries that are in it, in the, er, research at the moment?

Well they're still in the process of recruiting…

Oh, right.

… the patients into. So they're aiming for, um, each trial is aiming for roughly around 1000 patients.

Oh, that many?

So it takes a while for them to get that many patients in, you know, to start with and then they've got, um, the sort of 5-10 years follow-up.

And they're definitely going to split it 50/50 are they?

Yep.

Well maybe you could give them an incentive to do it, and only the people who accepted the incentive need go and they knew all the risks and everything else, and if you… I don't know what you'd offer them. A trip to the moon or something?

[LAUGHTER]

Then they'd… there's no ethical question, they've made the decision. And it's not their life and death situation, they've looked at both the options and the incentive makes it worthwhile.

Mmm hmm. So I mean all the… a trial won't be able to start unless it has approval from an ethics committee that, that consider the whole situation and determines that it is ethical to do the trial. Um, and of course it's always, er, important that the women are fully informed of the, the pros and cons of going in the trial.

And with doctors you get the old school that will never change.

That's right, yeah.

And the ones that will always be open to new things and, um, new trials and things like that. So it's just weighing up the two different types of doctors.

Mmm hmm.

Is it really difficult to coordinate say all the oncologists in Australia to submit data? 'Cause there must… I mean they do advise if it's low, then you ask the question, you don't have to have the treatment. They must have patients that choose not to, so that you don't have this dilemma of going away, a certain way in the trial that you don't want to go.

Mmm.

You're just getting the data from the doctors. Is that something, is that really a difficult thing to do?

Well the guidelines that guide the doctors at the moment say the standard thing to do is treatment.

At all costs?

So… that's what I'm saying. It's not a, it's, it's kind of an exceptional case if, for women to say, no, I really don't want anything. So it does happen, but there's very small numbers. Kind of so… kind of reflected in that other graph that I showed you…

Oh, I see.

… you, you could find small numbers of women who are not having treatment, but… you may well find that those women are typically older and sicker, and so you can't really make a comparison that you can generalise to the general population.

And that might have been a small, that was obviously a small sample, but it might have been too small. It's lovely to say that that says 99% of them survived, but if it had been the same amount of people there might have been more.

Mmm.

Which is very difficult. You've got a big job ahead of you if you want to go ahead with this.

[LAUGHTER]

Another 30 years before you sort it out.

[LAUGHTER]

I think only because cancer is a very scary disease.

[LOTS OF YEAH's]

Are they, are they graded, you know you said they are graded, are they graded one to four like other basic cancer is? You know how they say you've got a grade four…

So you might be thinking of stage, the term stage.

Oh, stage.

Yeah, we talk about stage one, two, three, four cancers.

It's stage. Mmm.

So DCIS is all, in terms of stage, it's considered like stage zero.

Oh, right.

And, and the grade, so cancer's also get grade, um…

Yeah.

… whereas grade is low, intermediate, high. It's a slightly different, um…

So it's stage, yeah…

Yeah.

That's right.

OK. Um… so… yeah, so we're getting towards the end. I just want to kind of wrap up the conversation. Um… so does, is everyone feeling like if you were in this situation you would be… er, happy to go into a trial potentially? Has anyone kind of shifted what they've said before in the last few minutes of discussion? I know it's a…

I think you have to wait till you're diagnosed.

[A FEW YES's]

'Cause we can all say one thing or the other, but unless you're diagnosed or your daughter's diagnosed then its… all bets are off then.

Mmm

We can all say we'd do it, then… it, it's very difficult.

[A FEW MMM's]

I agree.

Do you want us to make a decision yes or no, that you would or you wouldn't? Or do… are you quite happy with I don't know?

[LAUGHTER]

I don't know is a perfectly valid, um…

[LAUGHTER]

… answer.

But I think by having the trials is the only way that the, the cure for the disease is going to progress. Because if they don't have the trials, you know, how, how are things going to progress.

Yes, but this isn't a cure we're talking about.

Yeah.

It's a statistic, whether to take the treatment or not.

Yeah.

So from a selfish point of view I'd want to make my own choices (laughs).

Yes, same here. I'd rather make my own choice.

Yes, I think I'd like to make my own choice too.

If it was a cure that was involved, but it isn't really, is it?

[A FEW NO's]

I think as you said before it would be good to have two trials.

Yes.

One, one for treatment, one for, um…

Yeah, rather than let the powers that be make the decision.

Mmm.

I would rather, I wouldn't mind going for trial…

Yes.

… which, which ever side I want to.

Mmm.

Not where they put me, yeah.

And you might want to choose the doctor who operates on you.

[LAUGHTER]

And I'd certainly like to see the results of the people who are monitored, you know, just to see what that… is the outcome of that.

So these people going into England and America and Europe are brave people (laughs).

Mmm hmm.

Yeah, it'd be interesting the age groups as well.

Yes.

I think that's a huge factor.

Yes, and it'd be interesting to see whether the three, um, groups get the same sort of answer. Of if the American one gets a totally different one from the English one or the Danish one.

The European, yeah.

[A FEW MMM's]

Probably it would be completely different I'd say (laughs).

Well, you just don't know. It's, er… it's very interesting.

[A FEW YEAH's]

They are all, you know, talking to each other and so that they'll be able to

put their results together potentially and compare. So it's quite a… a global sort of collaboration in that sense.

It'd be nice to be still alive to get the results of that.

[LAUGHTER and YEAH's]

Absolutely.

[TALKING TOGETHER]

Ok, so, um, the final thing I'm going to ask you to do is one more questionnaire that's on the table. It's labeled as questionnaire two. Um, but before you start I just want to say thank you all so much for coming and sharing your views. It's been really valuable for us. Um, and when you've done that questionnaire you're free to go.

[TRANSCRIPT ENDS 01:41:14]

END
