# Supplementary material for: Women’s views about current and future management of Ductal Carcinoma in Situ (DCIS): A mixed-methods study
Source: PLoS One. 2023 Jul 21;18(7):e0288972. doi: 10.1371/journal.pone.0288972 (PMC10361483; doi:10.1371/journal.pone.0288972)
Supplement: S4 Appendix — (PDF) [file pone.0288972.s004.pdf]

ID	AgeGroup	Marital	AusBorn	Education	WorkStatus	GeneralHealth	BCWorry	BCScreening	ScreeningIntent	DCISownwords	DCIScausedeath	DCISreliablypredict	DCISlikelytreated	DCIStreatnotneed	ControlPrefScale	JoinTrial	Why
11	50-59	1	0	5	2	3	1	2	3	cells in the duct. carcinoma is that the cell has a different form which can be cancer sitiu is defining the type of cells	2	1	1	2	2	3	Trial might interfere with my time and family and my work
12	50-59	1	1	5	5	4	1	2	1	This is the milk duct cells which have become abnormal or cancerous and can possibly become worse if it invades other tissues in the breast.	2	2	1	2	1	1	I think this is a positive way in finding solutions to these or any illness/life threatening conditions
13	50-59	1	1	1	1	4	2	4	1	Abnormal cells contained in a milk duct that could stay as they are or could turn into invasive cancer at a later date.	2	1	1	1	1	1	I would be low risk and it is important that all options are considered - no one treatment suits everyone. Advancements in research will help the females in our families in the years to come.
14	50-59	1	0	5	3	2	3	4	1	DCIS to me is like an early warning sign. If caught early you could avoid breast cancer and any further complications.	1	2	3	1	1	1	To me the odds are in my favor and the monitoring is a safety net so I dont have anything to loose
15	50-59	1	0	1	1	5	1	1	2	An early, non invasive, contained cancer cells in the milk duct of the breast	2	2	1	2	2	2	I would like to have the option to have treatment or not as treatment may no even be necessary at this stage
16	50-59	1	0	2	5	4	3	0	1	abnormal (cancer) cells affecting the duct of the breast	1	1	1	1	2	1	I will decide after consulting with a specialist to join or do not join the clinical trial. Apart from I will consider my age, health condition, and my family
17	50-59	1	1	2	1	3	2	2	1	cancerous cells contained within the breast duct that may or may not develop into full breast cancer	2	3	1	1	3	1	I say that because I am in a low risk category and I would be closely monitored to ensure nothing changes for the worst and if it does I would be moved into the correct treatment as quickly as possible
18	50-59	1	1	4	1	4	1	2	1	Cancerous cells which in the milk ducts. Which is confined to this area alone. It may or may not lead to more invasive cancer. It can only be detected by screening as there are generally no symptoms to indicate that it is present	2	2	1	1	2	1	The trial does provide an option which is currently not available. My doctors opinion is of value because they will also add medical info into the process of deciding
19	50-59	1	0	4	2	3	1	4	1		3	2	1	1	4	1	I think if doctor say it good for me to try I will go for it
20	50-59	1	1	1	2	3	1	3	1	Cancerous cells that are in situ - still in the duct (milk duct of the breast)	2	2	1	1	2	2	I prefer after consideration of the data collected that I be actively monitored without surgery
21	50-59	2	0	1	1	3	2	1	1	DCIS is a localized cancer in the breast	2	2	1	1	3	3	Might depend on the situation then
22	50-59	1	1	1	5	4	2	2	1	It is the presence of cancerous cells within the duct/s of the breast	3	2	1	3	2	2	Because of the randomisation aspect of the trial I would want to decide myself whether to have treatment or not, not have that decided for me
23	50-59	3	1	4	5	3	1	0	3	Cancerous cells forming in the breast which may form into breast cancer but also may not	2	2	1	1	1	1	Id rather be monitored and not have such invasive treatment
24	50-59	1	1	4	2	4	2	2	1	Abnormal cells that are found in the milk ducts that may or may not become cancerous	2	3	3	1	2	2	I would prefer the decision on making to be in my hands
25	50-59	2	1	2	1	3	2	0	1	It is a carcinoma found in the milk ducts of the breast and is contained. It has not spread outside the ducts but could possibly do so in the future.	2	2	1	2	2	1	If there is a chance that the surgery and radiotherapyis unnecessary I would possibly prefer not to have it, but as there is no proven studies yet I think this would be a good option to be carefully monitored if you ended up in that group or get the treatment and it would be helpful for people in the future
26	50-59	2	0	2	5	3	2	1	3	traces of DCIS in the women's milk ducts after a mamogram. Only way to find early enough.	3	1	1	1	3	3	Depending on my age and the initial diagnosis. Being older I might not want to have surgery
27	50-59	4	0	5	2	5	1	0	4	It is non invasive breast cancer, it is confined to the ducts, survival rate is excellent. There are 2 forms of treatment; one, the standard treatment surgery and radiotherapy and second,what is called active monitoring. It might develop into breast cancer, can lie dormant for years. Good news is it is picked up by screening. There are no trials in Australia at present	2	1	2	1	3	1	Active monitoring appeals. It appears as if one is in control of ones treatment choices and being monitoring over long period of time (if thats possible in low - midrange DCIS) lens a different result than immediate treatment
28	50-59	1	0	1	1	4	1	4	1	Cancer in the milk duct that is in sitting - it may or may not progress to full blown cancer	2	2	1	1	2	2	Because it may not develop to fullblown cancer and I can actively monitor it and see where we go if it looks like it is changing I can opt to have the surgery then
29	50-59	4	1	1	3	5	1	4	1	Abnormal cells contained within a milk duct in the breast. Can progress to a breast cancer - detection through regular mamograms	2	2	1	3	2	1	Though with a very strong preference to be in the active monitoring group
30	70-74	3	1	1	3	2	2	4	1	Possible precursor to breast cancer	2	2	2	3	1	1	Like to know. Very important to have such a trial.
31	70-74	1	1	1	3	4	2	4	1	DCIS is changed cells wholly contained within the milk ducts	2	2	1	1	1	1	By joining a clincial trial an individual always has the oppurtunity to pull out if you do not feel comfortable with the outcomes. eg Which group you are in
32	70-74	1	0	2	3	5	1	4	2	Pre cancerous cells contained within the milk ducts of the breast.	2	2	1	1	2	2	Because of the randomness of selection for treatment or non treatment with monitoring and the chance of being obliged to have unnecessary surgery
33	70-74	1	1	4	3	4	2	4	1	It is a carcinoma in a milk duct (or ducts) in the breast which is contained but has the potential to spread into the breast and become breast cancer	1	1	2	2	3	3	I do not have this condition so my opinion is not necessarily the same at this time as it would be if/when face with the reality
34	70-74	1	0	2	3	2	1	1	5	It is a carcinoma in the milk gland that is contained	3	2	1	1	1	2	I would not go in the group that required surgery therefore I would be joining the group with the wrong attitude of mind
35	70-74	1	0	5	3	2	1	4	2	Cancerous cells in the milk ducts in women's breasts	2	2	1	1	2	1	I think all the information that can be gathered is important as I have a daughter and 3 granddaughters and I would like to think they are protected as they get older
36	70-74	1	1	2	3	5	1	4	3	Abnormal cells which can develop in the milk glands of women's breasts. They may remain in the milk gland or spread into the breast tissue. If they spread to the tissue then they can also spread to the rest of the body. They therefor must be treated.	2	2	1	1	2	2	Would not want to end up in the group which had the opposite treatment to the treatment wanted.
37	70-74	1	1	5	3	4	1	4	1	A condition diagnosed in milk ducts and may spread into invasive breast cancer	2	2	1	2	2	1	It is a way medical science forms opinions and can move forward with new treatment opinion also how to understand how medical issue develop and hope to treat them
38	70-74	1	1	5	3	4	2	4	1	Abnormal cells in milk ducts that with time might progress to breast cancer	2	2	3	1	2	1	At the very least, I would be closely monitored to see if the DCIS has progressed to the next grade
39	70-74	1	1	5	3	3	1	4	1	Pre cancer contained in the breast	2	2	1	1	1	2	I wouldnt go into a trial that was random, I would rather monitor myself and not have surgery
40	60-69	1	1	1	3	3	2	4	1	Cancer cells contained within the ducts	2	3	1	3	2	3	age, general health could be needed to be considered. Plus knowledge of the "grade" of the DCIS
41	60-69	1	0	1	2	4	2	4	1	formed in breast duct internally as a primary malignancy site non spread	2	1	1	1	2	3	
42	60-69	4	1	1	3	3	1	4	1	Pre cancer with a range of treatment options depending on the grade. A trial is a worthwhile step forward to gain further evidence for treatment options.	2	2	1	1	2	1	I have "a risk" personality providing there is education and lots of support for intervention if removed
43	60-69	4	1	5	3	1	2	4	1	Cancer cells confined / contained in a certain area such as the duct cell in the breast	2	2	1	1	2	1	Based on my health (side effects radiotherapy) etc.
44	60-69	1	0	1	3	4	2	4	1	cancer cells in the ductal wall that has not spread yet	2	2	1	1	3	3	Depends on circumtances at the time of DG
45	60-69	4	1	1	2	4	2	2	1	Localised cancer cells in the duct of the breasts. Have not spread beyond the site.	2	2	1	1	2	3	Depends on circumstances at the time
47	60-69	2	1	2	1	5	2	4	1	Possible small spots that may end up being a problem later on - so should be looked into further by your Dr	1	2	1	1	2	1	need to get more medical knowledge
48	60-69	2	1	2	2	3	2	4	1	Cancer in the breast ducts	2	2	1	2	2	3	Working part time and looking after elderly infermed parents. Father currently in rehab after hospital treatment - Alzheimer- Mother not able to walk due to ill health
49	60-69	4	0	4	2	4	1	3	1	Cancer in the breast milk ducts	3	2	1	2	3	1	If I have DCIS and I have any chance of going into remission I will try anything
50	60-69	1	0	6	5	3	1	2	3	I think its very good!	3	3	1				
51	60-69	1	1	2	3	5	1	4	1	Cancer cells that are detected which are wholly contained within the ducts	2	2	1	1	3	1	Based on the opinion of the doctor that my DCIS was of low risk
52	60-69	4	1	1	3	4	1	0	4	Cancer cells within ducts - not invasive breast cancer - although some women diagnosed with DCIS may go on to develop invasive breast cancer	2	2	1	1	1	2	Whilst I believe in the need to have more information my personal choice would overall the random allocation
53	60-69	1	1	5	3	2	1	0	5	Would go and have surgery	1	1	1	1		2	To get final clinical trial
55	60-69	2	0	2	2	3	1	1	2	Condition that can lead to breast cancer	3	2	1	3	1	1	Clinical trial would give more option to care my DCIS condition
56	60-69	1	0	5	2	3	3	4	1	Carcinoma- means cancer - milk duct not developed to evasive cancer	1	1	1	1		1	because I have a choice to leave clinical trial whenever I want - only basis I would join
57	60-69	3	0	4	3	3	2	4	1	A type of breast condition that could or could not become cancer	2	2	3	2	2	1	That it would be for the best option
58	60-69	1	1	1	2	3	1	4	1	Cancer contained within the milk ducts	2	2	1	1	2	1	research has to be done in order for information to be gathered to further develop treatment plans
59	60-69	1	1	2	3	5	1	4	1	A cancer confined to a milk duct within the breast	2	2	1	1	2	1	need to give researchers an oppurtunity to collect data and develop new drugs and procedures
60	70-74	2	0	2	5	2	2	4	1	DCIS within the milk ducts may remain there escape from milk ducts can become invasive cancer and develop further	2	2	1	1	2	1	Age and expectations at present time
61	70-74	1	1	5	3	5	2	4	1	Abonormal cells in the milk ducts	2	1	2	2	1	2	I would refer if operation needed, to choose my surgeon
63	70-74	1	1	2	3	5	2	4	3	Cancer cells contained in the milk duct	2	2	1	1	3	3	I dont think you would know unless you were diagnosed. I would like to help a clinical trial but dont know exactly what I would decide
64	70-74	1	0	2	2	2	2	4	1	Carcinoma that is completely contained within a milk duct in the breast	2	1	1	1	1	2	I would prefer to choose whether to be treated or not as carcinoma is a scary disease and survival for most people is really important
65	70-74	3	1	5	3	3	2	4	4	something associated with breast cancer	2	2	1	1	4	1	I dont want treatment and radiation unnecessary
66	70-74	1	0	2	3	5	3	4	1	Mutated cells which remain inside the duct	2	2	1	2	3	3	I think it is impossible to be sure until a diagnosis is actually made
67	70-74	1	1	5	3	5	1	1	1	cancer in the duct glands	3	3	2	2		3	until you are diagnosed you will not be able to say which you will decide
68	70-74	1	1	5	3	3	2	4	1	DCIS is a cancer that affects the duct glands it can confine itself just to the ducts or become evasive and spread further	2	2	1	2	3	1	Trials give results without results we cannot help to develop a cure
69	70-74	2	0	1	3	2	1	4	3	Cancerous condition inside the confines of the ducts in the breast that are intended to transport milk to the nipple	2	3	1	3	2	1	I think that would give me the best chacne of acheiveing a good outcome plus I would be indirectly contributing to the possible improvement of medical knowledge and practice
