# Supplementary material for: Women’s views about current and future management of Ductal Carcinoma in Situ (DCIS): A mixed-methods study
Source: PLoS One. 2023 Jul 21;18(7):e0288972. doi: 10.1371/journal.pone.0288972 (PMC10361483; doi:10.1371/journal.pone.0288972)
Supplement: Attachment — Submitted filename: Nickel et al PLOS One response to reviewers.docx [file pone.0288972.s005.docx]

24 April 2023

Editor in Chief, *PLOS One*

Dear Dr Emily Chenette

**Re: Women’s views about current and future management of Ductal Carcinoma in Situ (DCIS): a mixed-methods study**

Thank you for giving us the opportunity to revise and resubmit our manuscript to *PLOS One.*

We have provided a point-by-point response to reviewers below as indicated in the decision correspondence. We have also marked any changes made in the manuscript using tracked changes and uploaded this as a tracked version along with an unmarked version of the manuscript.

Sincerely,

Brooke Nickel, on behalf of all authors

**Journal Requirements:**

**When submitting your revision, we need you to address these additional requirements.**

**1. Please ensure that your manuscript meets PLOS ONE's style requirements, including those for file naming.**

We have ensured that the manuscript meets the PLOS One’s style requirements.

**2. We note that the grant information you provided in the ‘Funding Information’ and ‘Financial Disclosure’ sections do not match. When you resubmit, please ensure that you provide the correct grant numbers for the awards you received for your study in the ‘Funding Information’ section.**

We have ensured that the Funding Information in the manuscript and Financial Disclosures section now align. Please note that the grant through which this study was funded does not have a specific grant number.

**3. We note that you have indicated that data from this study are available upon request. PLOS only allows data to be available upon request if there are legal or ethical restrictions on sharing data publicly. For more information on unacceptable data access restrictions, please see** [**http://journals.plos.org/plosone/s/data-availability#loc-unacceptable-data-access-restrictions**](https://protect-au.mimecast.com/s/GttHCOMKzVTN9joyMf5myvE?domain=journals.plos.org)**.**

**In your revised cover letter, please address the following prompts:**

**a) If there are ethical or legal restrictions on sharing a de-identified data set, please explain them in detail (e.g., data contain potentially sensitive information, data are owned by a third-party organization, etc.) and who has imposed them (e.g., an ethics committee). Please also provide contact information for a data access committee, ethics committee, or other institutional body to which data requests may be sent.**

**b) If there are no restrictions, please upload the minimal anonymized data set necessary to replicate your study findings as either Supporting Information files or to a stable, public repository and provide us with the relevant URLs, DOIs, or accession numbers. For a list of acceptable repositories, please see** [**http://journals.plos.org/plosone/s/data-availability#loc-recommended-repositories**](https://protect-au.mimecast.com/s/R369CP7LAXf0rRqkPfBA80m?domain=journals.plos.org)**.**

**We will update your Data Availability statement on your behalf to reflect the information you provide.**

We have now uploaded the anonymised data set (combined transcript files and questionnaire data spreadsheet) as Supporting Information files (3 and 4).

**4. Your ethics statement should only appear in the Methods section of your manuscript. If your ethics statement is written in any section besides the Methods, please delete it from any other section.**

We have removed the Ethics statement from the information at end of the manuscript ensuring that it is now only included in the Methods section of the manuscript.

**5. Please include captions for your Supporting Information files at the end of your manuscript, and update any in-text citations to match accordingly.**

We have now included captions for our Supporting Information files at the end of the manuscript and ensure in-text citations to these files match accordingly.

**6. Review your reference list to ensure that it is complete and correct.**

We have not made any significant changes to the reference list aside from correcting minor typos and punctuation errors in references as highlighted by Reviewer 2.

**Additional Editor Comments:

Dear Authors,
Management of low-risk ductal carcinoma in situ is controversial and little is known about general community views about its management.
This article is interesting and I think that you can easily address reviewers' suggestions.
Consequently, I suggest a minor revision before accepting the paper.**

Thank you.

**Reviewers' Comments:**

**Reviewer #1: Management of low-risk ductal carcinoma in situ (DCIS) is controversial, with clinical trials currently assessing the safety of active monitoring amidst concern about overtreatment. Little is known about general community views regarding DCIS and its management.
If the participants enrolled are medical staff with breast cancer, it would be more meaningful.**

Thank you for reviewing our manuscript.

**Reviewer #2: This paper assessed Australian women's views regarding the management of ductal carcinoma in situ (DCIS).
1. In the Introduction, 2nd paragraph, 2nd sentence, I suggest: The incidence of DCIS has markedly. In the 3rd sentence, the authors should note that when comparing the treatment of DCIS to the treatment of invasive carcinoma, chemotherapy is not administered in DCIS cases. In the 4th sentence, the authors should provide some examples of physical and psychological harm due to treatment for DCIS.**

Thank you for your thorough review of our manuscript.

We have incorporated your suggested edits into the second paragraph of the Introduction. Please see page 3: “Before widespread breast screening, DCIS was diagnosed rarely. The incidence of DCIS has markedly increased since implementation of organised screening, and DCIS represents approximately 20% of screen-detected malignant lesions [2]. There are now concerns that screening has led to overdiagnosis and overtreatment of low-risk (small volume, low to intermediate grade) DCIS [3, 4], yet the majority of patients with DCIS of all grades are offered treatment, including surgery, radiotherapy and endocrine therapies which are similar to treatment for invasive cancer [5], importantly noting that chemotherapy is not administered in DCIS cases. These treatments may not improve overall survival which is already excellent, particularly for low-grade DCIS, and may cause detriments to health and quality of life including physical (e.g. cardiotoxicity, pain and fatigue) and psychological harm (e.g. negative effect on body image) [6,7][8].”

**2. In Materials and Methods, Study design, 2nd sentence, the authors should provide examples to indicate that the state of the science has moved forward substantially.**

We have now provided more details in relation to the state of science. Please see page 4: “Our study built on qualitative work done in preparation for the LORIS DCIS monitoring trial [21], and was conducted with a larger Australian sample several years later in a context where the state of the science in relation to the management of DCIS had moved forward substantially and three international clinical trials, including the LORIS trial, had commenced [12-14].”

**3. In Materials and Methods, Participant recruitment, 1st paragraph, the authors should indicate how the subjects were recruited. In the 3rd sentence, the authors should discuss why those not fluent in English were excluded. Excluding the non-English speaking women appears to bias the selection process.**

We had previously included details about how women were recruited into the study by an independent research recruitment agency using random digit dialling. We have now included the name of the recruitment agency (Taverner Research) and further expanded this information. We have also addressed why non-English speakers were excluded. Please see page 4: “We recruited a community sample of women across metropolitan Sydney, Australia. Women were aged 50-74 years, the target age range for population-based breast screening in Australia. An independent research recruitment organisation (Taverner Research) used random landline and location known mobile samples from Sydney to contact potential participants by phone. Trained interviewers introduced the study as a ‘focus group about women’s health’ and used a series of questions to assess eligibility, excluding women with a personal history of breast cancer or DCIS and those not fluent in English as interpreting services were unavailable for this study. Interested participants were then mailed/emailed the Participant Information Statement and booked into a focus group time.”

We have also added a sentence into the Limitations section of the Discussion to address this point about non-English speakers. Please see page 16: “We recruited a diverse sample of women varying in educational background. However, it is important to note that excluding non-English speaking women means our findings may not reflect the views of such women.”

**4. In Materials and Methods, Participant recruitment, 2nd paragraph, in the last sentence, to reach thematic data saturation, as indicated by data redundancy is not clear.**

We have now clarified this sentence. Please see page 4: “This number of participants enabled us to reach thematic data saturation, as indicated by data redundancy i.e. when participants no longer raised original themes [22, 23].”

**5. In Materials and Methods, Data collection and analysis, 1st paragraph, 2nd sentence, I suggest: read all of the transcripts. In the 3rd sentence, an inductive approach and at semantic level are not clear. In the 5th sentence, more details should be provided regarding checking a sub-sample.**

We have now made the suggested changes and clarifications. Please see page 6: “Focus group discussions were audio-recorded and transcribed verbatim. Transcripts were thematically analysed to identify recurring themes and data patterns [24], using NVivo software. Two researchers (BN and JH) independently reviewed notes taken during the focus groups and one researcher (BN) read all of the transcripts. Analysis initially took an inductive approach where there were no set or pre-determined hypotheses to ensure the findings were grounded in participant responses, and data were analysed based on the explicit meaning of what participants said (i.e. at semantic level). Using constant comparison [22, 25], the two researchers (BN and JH) discussed similarities and differences in the data and developed the coding framework. Coding was performed by one researcher (BN), with JH checking a sub-sample of the data to ensure coding was consistent with the framework. The final coding was then examined by two researchers (BN and AD) and discussed with JH to identify overarching themes and concepts.”

**6. In Materials and Methods, Data collection and analysis, 2nd paragraph, 1st sentence, breast cancer worry and future intentions should be defined. Clarification is needed in the 3rd sentence, which indicated that free-text responses were scored as 0, 1, or 2.**

We have now clarified the measures used and the scoring of the free-text responses further. Please see page 6: “Questionnaire 1 (start of session) included demographics, breast cancer worry (*not worried at all, a bit worried, quite worried, very worried*), breast screening history *(never been screened, once, twice, three or more times*) and future intentions (*definitely will, likely, unsure, not likely, definitely will not*). Questionnaire 2 (end of session) assessed conceptual understanding of DCIS using a free-text item (defining DCIS) and four true/false questions adapted from previous research [26], hypothetical intentions towards joining a DCIS monitoring trial (multiple choice) together with reasons (free text), and shared decision making preferences [27]. Free-text responses for women’s definitions of DCIS were scored as 0, 1, or 2 according to how many of the following two points they indicated correctly: (i) that DCIS represents a change within cells confined to the breast milk ducts, and (ii) the uncertain potential for DCIS to progress to invasive cancer. Responses regarding interest in joining a DCIS clinical trial were summarised descriptively, and free-text responses about the corresponding reasons were incorporated into the thematic analysis.”

**7. In Table 1, age should be indicated in years. Does university degree mean undergraduate degree? Leaving certificate is not clear.**

We have now clarified the points above and update Table 1 accordingly. Please see pages 6-7.

**8. In Discussion, 1st paragraph, 2nd sentence, the should be added before final.**

This has now been added. Please see page 15.

**9. In Discussion, 3rd paragraph, 2nd sentence, the should be added before importance.**

This has now been added. Please see page 15.

**10. In Discussion, Strengths and limitations, 12th line, the should be added before participants.**

This has now been added. Please see page 16.

**11. In the references, only the first word of the title of the article should be capitalized in references 3, 4, 6, 10, 15, 17, 27, and 34. The journal for reference 3 is JAMA Oncology. The journal for references 7, 14, and 37 is European Journal of Cancer. The journal is reference 8 is BMJ. In reference 11, the last author citation should be Grand Challenge PRECISION Consortium. The journal in reference 11 is Breast Cancer Research and Treatment. In reference 13, the journal is BMJ Open. In references 18 and 29, the journal is Health Expectations. In reference 21, the journal is Lancet Oncology. In reference 24, the reference is Qualitative Research in Psychology. In reference 28, the journal is Patient Education and Counseling. In reference 30, the journal is JAMA. In reference 31, the journal is British Journal of Cancer. In references 32 and 33, the journal is Medical Decision Making. In reference 34, the journal is Thyroid. In reference 35, the journal is Urologic Oncology.**

We have now updated the reference list accordingly. Please see pages 17-19.
