# Supplementary material for: Women’s views about current and future management of Ductal Carcinoma in Situ (DCIS): A mixed-methods study
Source: PLoS One. 2023 Jul 21;18(7):e0288972. doi: 10.1371/journal.pone.0288972 (PMC10361483; doi:10.1371/journal.pone.0288972)
Supplement: Peer review history

# Peer review: Decision Letter 0

**DOI:** 10.1371/journal.pone.0288972.r001

**Authors:** Daniele Ugo Tari

15 Mar 2023

PONE-D-23-00830Women’s views about current and future management of Ductal Carcinoma in Situ (DCIS): a mixed-methods studyPLOS ONE

Dear Dr. Nickel,

Thank you for submitting your manuscript to PLOS ONE. After careful consideration, we feel that it has merit but does not fully meet PLOS ONE’s publication criteria as it currently stands. Therefore, we invite you to submit a revised version of the manuscript that addresses the points raised during the review process. Please, address the suggestions provided by reviewer #2. Please submit your revised manuscript by Apr 29 2023 11:59PM. If you will need more time than this to complete your revisions, please reply to this message or contact the journal office at plosone@plos.org. When you're ready to submit your revision, log on to https://www.editorialmanager.com/pone/ and select the 'Submissions Needing Revision' folder to locate your manuscript file.

Please include the following items when submitting your revised manuscript:

- A rebuttal letter that responds to each point raised by the academic editor and reviewer(s). You should upload this letter as a separate file labeled 'Response to Reviewers'.
- A marked-up copy of your manuscript that highlights changes made to the original version. You should upload this as a separate file labeled 'Revised Manuscript with Track Changes'.
- An unmarked version of your revised paper without tracked changes. You should upload this as a separate file labeled 'Manuscript'.

If you would like to make changes to your financial disclosure, please include your updated statement in your cover letter. Guidelines for resubmitting your figure files are available below the reviewer comments at the end of this letter.

If applicable, we recommend that you deposit your laboratory protocols in protocols.io to enhance the reproducibility of your results. Protocols.io assigns your protocol its own identifier (DOI) so that it can be cited independently in the future. For instructions see: https://journals.plos.org/plosone/s/submission-guidelines#loc-laboratory-protocols. Additionally, PLOS ONE offers an option for publishing peer-reviewed Lab Protocol articles, which describe protocols hosted on protocols.io. Read more information on sharing protocols at https://plos.org/protocols?utm_medium=editorial-email&utm_source=authorletters&utm_campaign=protocols.

We look forward to receiving your revised manuscript.

Kind regards,

Daniele Ugo Tari, M.D.

Academic Editor

PLOS ONE

Journal Requirements:

When submitting your revision, we need you to address these additional requirements.

1. Please ensure that your manuscript meets PLOS ONE's style requirements, including those for file naming. The PLOS ONE style templates can be found at 

https://journals.plos.org/plosone/s/file?id=wjVg/PLOSOne_formatting_sample_main_body.pdf and 

https://journals.plos.org/plosone/s/file?id=ba62/PLOSOne_formatting_sample_title_authors_affiliations.pdf

2. We note that the grant information you provided in the ‘Funding Information’ and ‘Financial Disclosure’ sections do not match. 

When you resubmit, please ensure that you provide the correct grant numbers for the awards you received for your study in the ‘Funding Information’ section.

3. We note that you have indicated that data from this study are available upon request. PLOS only allows data to be available upon request if there are legal or ethical restrictions on sharing data publicly. For more information on unacceptable data access restrictions, please see http://journals.plos.org/plosone/s/data-availability#loc-unacceptable-data-access-restrictions. 

In your revised cover letter, please address the following prompts:

a) If there are ethical or legal restrictions on sharing a de-identified data set, please explain them in detail (e.g., data contain potentially sensitive information, data are owned by a third-party organization, etc.) and who has imposed them (e.g., an ethics committee). Please also provide contact information for a data access committee, ethics committee, or other institutional body to which data requests may be sent.

b) If there are no restrictions, please upload the minimal anonymized data set necessary to replicate your study findings as either Supporting Information files or to a stable, public repository and provide us with the relevant URLs, DOIs, or accession numbers. For a list of acceptable repositories, please see http://journals.plos.org/plosone/s/data-availability#loc-recommended-repositories.

We will update your Data Availability statement on your behalf to reflect the information you provide.

4. Your ethics statement should only appear in the Methods section of your manuscript. If your ethics statement is written in any section besides the Methods, please delete it from any other section. 

5. Please include captions for your Supporting Information files at the end of your manuscript, and update any in-text citations to match accordingly. Please see our Supporting Information guidelines for more information: http://journals.plos.org/plosone/s/supporting-information. 

6. review your reference list to ensure that it is complete and correct. If you have cited papers that have been retracted, please include the rationale for doing so in the manuscript text, or remove these references and replace them with relevant current references. Any changes to the reference list should be mentioned in the rebuttal letter that accompanies your revised manuscript. If you need to cite a retracted article, indicate the article’s retracted status in the References list and also include a citation and full reference for the retraction notice.

Additional Editor Comments:

Dear Authors,

Management of low-risk ductal carcinoma in situ is controversial and little is known about general community views about its management.

This article is interesting and I think that you can easily address reviewers' suggestions.

Consequently, I suggest a minor revision before accepting the paper.

Sincerely,

[Note: HTML markup is below. Please do not edit.]

Reviewers' comments:

Reviewer's Responses to Questions

**Comments to the Author**

1. Is the manuscript technically sound, and do the data support the conclusions?

The manuscript must describe a technically sound piece of scientific research with data that supports the conclusions. Experiments must have been conducted rigorously, with appropriate controls, replication, and sample sizes. The conclusions must be drawn appropriately based on the data presented. 

Reviewer #1: Yes

Reviewer #2: Yes

**********

2. Has the statistical analysis been performed appropriately and rigorously? 

Reviewer #1: N/A

Reviewer #2: Yes

**********

3. Have the authors made all data underlying the findings in their manuscript fully available?

The PLOS Data policy requires authors to make all data underlying the findings described in their manuscript fully available without restriction, with rare exception (please refer to the Data Availability Statement in the manuscript PDF file). The data should be provided as part of the manuscript or its supporting information, or deposited to a public repository. For example, in addition to summary statistics, the data points behind means, medians and variance measures should be available. If there are restrictions on publicly sharing data—e.g. participant privacy or use of data from a third party—those must be specified.

Reviewer #1: Yes

Reviewer #2: Yes

**********

4. Is the manuscript presented in an intelligible fashion and written in standard English?

PLOS ONE does not copyedit accepted manuscripts, so the language in submitted articles must be clear, correct, and unambiguous. Any typographical or grammatical errors should be corrected at revision, so please note any specific errors here.

Reviewer #1: Yes

Reviewer #2: Yes

**********

5. Review Comments to the Author

Please use the space provided to explain your answers to the questions above. You may also include additional comments for the author, including concerns about dual publication, research ethics, or publication ethics. (Please upload your review as an attachment if it exceeds 20,000 characters)

Reviewer #1: Management of low-risk ductal carcinoma in situ (DCIS) is controversial, with clinical trials currently assessing the safety of active monitoring amidst concern about overtreatment.Little is known about general community views regarding DCIS and its management.

If the participants enrolled are medical staff with breast cancer, it would be more meaningful.

Reviewer #2: This paper assessed Australian women's views regarding the management of ductal carcinoma in situ (DCIS).

1. In the Introduction, 2nd paragraph, 2nd sentence, I suggest: The incidence of DCIS has markedly. In the 3rd sentence, the authors should note that when comparing the treatment of DCIS to the treatment of invasive carcinoma, chemotherapy is not administered in DCIS cases. In the 4th sentence, the authors should provide some examples of physical and psychological harm due to treatment for DCIS.

2. In Materials and Methods, Study design, 2nd sentence, the authors should provide examples to indicate that the state of the science has moved forward substantially.

3. In Materials and Methods, Participant recruitment, 1st paragraph, the authors should indicate how the subjects were recruited. In the 3rd sentence, the authors should discuss why those not fluent in English were excluded. Excluding the non-English speaking women appears to bias the selection process.

4. In Materials and Methods, Participant recruitment, 2nd paragraph, in the last sentence, to reach thematic data saturation, as indicated by data redundancy is not clear.

5. In Materials and Methods, Data collection and analysis, 1st paragraph, 2nd sentence, I suggest: read all of the transcripts. In the 3rd sentence, an inductive approach and at semantic level are not clear. In the 5th sentence, more details should be provided regarding checking a sub-sample.

6. In Materials and Methods, Data collection and analysis, 2nd paragraph, 1st sentence, breast cancer worry and future intentions should be defined. Clarification is needed in the 3rd sentence, which indicated that free-text responses were scored as 0, 1, or 2.

7. In Table 1, age should be indicated in years. Does university degree mean undergraduate degree? Leaving certificate is not clear.

8. In Discussion, 1st paragraph, 2nd sentence, the should be added before final.

9. In Discussion, 3rd paragraph, 2nd sentence, the should be added before importance.

10. In Discussion, Strengths and limitations, 12th line, the should be added before participants.

11. In the references, only the first word of the title of the article should be capitalized in references 3, 4, 6, 10, 15, 17, 27, and 34. The journal for reference 3 is JAMA Oncology. The journal for references 7, 14, and 37 is European Journal of Cancer. The journal is reference 8 is BMJ. In reference 11, the last author citation should be Grand Challenge PRECISION Consortium. The journal in reference 11 is Breast Cancer Research and Treatment. In reference 13, the journal is BMJ Open. In references 18 and 29, the journal is Health Expectations. In reference 21, the journal is Lancet Oncology. In reference 24, the reference is Qualitative Research in Psychology. In reference 28, the journal is Patient Education and Counseling. In reference 30, the journal is JAMA. In reference 31, the journal is British Journal of Cancer. In references 32 and 33, the journal is Medical Decision Making. In reference 34, the journal is Thyroid. In reference 35, the journal is Urologic Oncology.

**********

6. PLOS authors have the option to publish the peer review history of their article (what does this mean?). If published, this will include your full peer review and any attached files.

If you choose “no”, your identity will remain anonymous but your review may still be made public.

**Do you want your identity to be public for this peer review?** For information about this choice, including consent withdrawal, please see our Privacy Policy.

Reviewer #1: **Yes: **Jingxian Ding

Reviewer #2: **Yes: **Gary Whitman

**********

[NOTE: If reviewer comments were submitted as an attachment file, they will be attached to this email and accessible via the submission site. Please log into your account, locate the manuscript record, and check for the action link "View Attachments". If this link does not appear, there are no attachment files.]

While revising your submission, please upload your figure files to the Preflight Analysis and Conversion Engine (PACE) digital diagnostic tool, https://pacev2.apexcovantage.com/. PACE helps ensure that figures meet PLOS requirements. To use PACE, you must first register as a user. Registration is free. Then, login and navigate to the UPLOAD tab, where you will find detailed instructions on how to use the tool. If you encounter any issues or have any questions when using PACE, please email PLOS at figures@plos.org. Please note that Supporting Information files do not need this step.

# Author comment: Author response to Decision Letter 0

**DOI:** 10.1371/journal.pone.0288972.r002

26 Apr 2023

24 April 2023

Editor in Chief, PLOS One 

Dear Dr Emily Chenette

Re: Women’s views about current and future management of Ductal Carcinoma in Situ (DCIS): a mixed-methods study 

Thank you for giving us the opportunity to revise and resubmit our manuscript to PLOS One. 

We have provided a point-by-point response to reviewers below as indicated in the decision correspondence. We have also marked any changes made in the manuscript using tracked changes and uploaded this as a tracked version along with an unmarked version of the manuscript. 

Sincerely, 

Brooke Nickel, on behalf of all authors

Journal Requirements:

When submitting your revision, we need you to address these additional requirements.

1. Please ensure that your manuscript meets PLOS ONE's style requirements, including those for file naming. 

We have ensured that the manuscript meets the PLOS One’s style requirements. 

2. We note that the grant information you provided in the ‘Funding Information’ and ‘Financial Disclosure’ sections do not match. When you resubmit, please ensure that you provide the correct grant numbers for the awards you received for your study in the ‘Funding Information’ section.

We have ensured that the Funding Information in the manuscript and Financial Disclosures section now align. Please note that the grant through which this study was funded does not have a specific grant number. 

3. We note that you have indicated that data from this study are available upon request. PLOS only allows data to be available upon request if there are legal or ethical restrictions on sharing data publicly. For more information on unacceptable data access restrictions, please see http://journals.plos.org/plosone/s/data-availability#loc-unacceptable-data-access-restrictions. 

In your revised cover letter, please address the following prompts:

a) If there are ethical or legal restrictions on sharing a de-identified data set, please explain them in detail (e.g., data contain potentially sensitive information, data are owned by a third-party organization, etc.) and who has imposed them (e.g., an ethics committee). Please also provide contact information for a data access committee, ethics committee, or other institutional body to which data requests may be sent.

b) If there are no restrictions, please upload the minimal anonymized data set necessary to replicate your study findings as either Supporting Information files or to a stable, public repository and provide us with the relevant URLs, DOIs, or accession numbers. For a list of acceptable repositories, please see http://journals.plos.org/plosone/s/data-availability#loc-recommended-repositories.

We will update your Data Availability statement on your behalf to reflect the information you provide.

We have now uploaded the anonymised data set (combined transcript files and questionnaire data spreadsheet) as Supporting Information files (3 and 4). 

4. Your ethics statement should only appear in the Methods section of your manuscript. If your ethics statement is written in any section besides the Methods, please delete it from any other section.

We have removed the Ethics statement from the information at end of the manuscript ensuring that it is now only included in the Methods section of the manuscript. 

5. Please include captions for your Supporting Information files at the end of your manuscript, and update any in-text citations to match accordingly. 

We have now included captions for our Supporting Information files at the end of the manuscript and ensure in-text citations to these files match accordingly. 

6. Review your reference list to ensure that it is complete and correct.

We have not made any significant changes to the reference list aside from correcting minor typos and punctuation errors in references as highlighted by Reviewer 2. 

Additional Editor Comments:

Dear Authors,

Management of low-risk ductal carcinoma in situ is controversial and little is known about general community views about its management.

This article is interesting and I think that you can easily address reviewers' suggestions.

Consequently, I suggest a minor revision before accepting the paper.

Thank you. 

Reviewers' Comments:

Reviewer #1: Management of low-risk ductal carcinoma in situ (DCIS) is controversial, with clinical trials currently assessing the safety of active monitoring amidst concern about overtreatment. Little is known about general community views regarding DCIS and its management.

If the participants enrolled are medical staff with breast cancer, it would be more meaningful.

Thank you for reviewing our manuscript. 

Reviewer #2: This paper assessed Australian women's views regarding the management of ductal carcinoma in situ (DCIS).

1. In the Introduction, 2nd paragraph, 2nd sentence, I suggest: The incidence of DCIS has markedly. In the 3rd sentence, the authors should note that when comparing the treatment of DCIS to the treatment of invasive carcinoma, chemotherapy is not administered in DCIS cases. In the 4th sentence, the authors should provide some examples of physical and psychological harm due to treatment for DCIS.

Thank you for your thorough review of our manuscript. 

We have incorporated your suggested edits into the second paragraph of the Introduction. Please see page 3: “Before widespread breast screening, DCIS was diagnosed rarely. The incidence of DCIS has markedly increased since implementation of organised screening, and DCIS represents approximately 20% of screen-detected malignant lesions [2]. There are now concerns that screening has led to overdiagnosis and overtreatment of low-risk (small volume, low to intermediate grade) DCIS [3, 4], yet the majority of patients with DCIS of all grades are offered treatment, including surgery, radiotherapy and endocrine therapies which are similar to treatment for invasive cancer [5], importantly noting that chemotherapy is not administered in DCIS cases. These treatments may not improve overall survival which is already excellent, particularly for low-grade DCIS, and may cause detriments to health and quality of life including physical (e.g. cardiotoxicity, pain and fatigue) and psychological harm (e.g. negative effect on body image) [6,7][8].”

2. In Materials and Methods, Study design, 2nd sentence, the authors should provide examples to indicate that the state of the science has moved forward substantially.

We have now provided more details in relation to the state of science. Please see page 4: “Our study built on qualitative work done in preparation for the LORIS DCIS monitoring trial [21], and was conducted with a larger Australian sample several years later in a context where the state of the science in relation to the management of DCIS had moved forward substantially and three international clinical trials, including the LORIS trial, had commenced [12-14].”

3. In Materials and Methods, Participant recruitment, 1st paragraph, the authors should indicate how the subjects were recruited. In the 3rd sentence, the authors should discuss why those not fluent in English were excluded. Excluding the non-English speaking women appears to bias the selection process. 

We had previously included details about how women were recruited into the study by an independent research recruitment agency using random digit dialling. We have now included the name of the recruitment agency (Taverner Research) and further expanded this information. We have also addressed why non-English speakers were excluded. Please see page 4: “We recruited a community sample of women across metropolitan Sydney, Australia. Women were aged 50-74 years, the target age range for population-based breast screening in Australia. An independent research recruitment organisation (Taverner Research) used random landline and location known mobile samples from Sydney to contact potential participants by phone. Trained interviewers introduced the study as a ‘focus group about women’s health’ and used a series of questions to assess eligibility, excluding women with a personal history of breast cancer or DCIS and those not fluent in English as interpreting services were unavailable for this study. Interested participants were then mailed/emailed the Participant Information Statement and booked into a focus group time.”

We have also added a sentence into the Limitations section of the Discussion to address this point about non-English speakers. Please see page 16: “We recruited a diverse sample of women varying in educational background. However, it is important to note that excluding non-English speaking women means our findings may not reflect the views of such women.”

4. In Materials and Methods, Participant recruitment, 2nd paragraph, in the last sentence, to reach thematic data saturation, as indicated by data redundancy is not clear.

We have now clarified this sentence. Please see page 4: “This number of participants enabled us to reach thematic data saturation, as indicated by data redundancy i.e. when participants no longer raised original themes [22, 23].”

5. In Materials and Methods, Data collection and analysis, 1st paragraph, 2nd sentence, I suggest: read all of the transcripts. In the 3rd sentence, an inductive approach and at semantic level are not clear. In the 5th sentence, more details should be provided regarding checking a sub-sample.

We have now made the suggested changes and clarifications. Please see page 6: “Focus group discussions were audio-recorded and transcribed verbatim. Transcripts were thematically analysed to identify recurring themes and data patterns [24], using NVivo software. Two researchers (BN and JH) independently reviewed notes taken during the focus groups and one researcher (BN) read all of the transcripts. Analysis initially took an inductive approach where there were no set or pre-determined hypotheses to ensure the findings were grounded in participant responses, and data were analysed based on the explicit meaning of what participants said (i.e. at semantic level). Using constant comparison [22, 25], the two researchers (BN and JH) discussed similarities and differences in the data and developed the coding framework. Coding was performed by one researcher (BN), with JH checking a sub-sample of the data to ensure coding was consistent with the framework. The final coding was then examined by two researchers (BN and AD) and discussed with JH to identify overarching themes and concepts.”

6. In Materials and Methods, Data collection and analysis, 2nd paragraph, 1st sentence, breast cancer worry and future intentions should be defined. Clarification is needed in the 3rd sentence, which indicated that free-text responses were scored as 0, 1, or 2.

We have now clarified the measures used and the scoring of the free-text responses further. Please see page 6: “Questionnaire 1 (start of session) included demographics, breast cancer worry (not worried at all, a bit worried, quite worried, very worried), breast screening history (never been screened, once, twice, three or more times) and future intentions (definitely will, likely, unsure, not likely, definitely will not). Questionnaire 2 (end of session) assessed conceptual understanding of DCIS using a free-text item (defining DCIS) and four true/false questions adapted from previous research [26], hypothetical intentions towards joining a DCIS monitoring trial (multiple choice) together with reasons (free text), and shared decision making preferences [27]. Free-text responses for women’s definitions of DCIS were scored as 0, 1, or 2 according to how many of the following two points they indicated correctly: (i) that DCIS represents a change within cells confined to the breast milk ducts, and (ii) the uncertain potential for DCIS to progress to invasive cancer. Responses regarding interest in joining a DCIS clinical trial were summarised descriptively, and free-text responses about the corresponding reasons were incorporated into the thematic analysis.”

7. In Table 1, age should be indicated in years. Does university degree mean undergraduate degree? Leaving certificate is not clear.

We have now clarified the points above and update Table 1 accordingly. Please see pages 6-7.

8. In Discussion, 1st paragraph, 2nd sentence, the should be added before final.

This has now been added. Please see page 15.

9. In Discussion, 3rd paragraph, 2nd sentence, the should be added before importance.

This has now been added. Please see page 15.

10. In Discussion, Strengths and limitations, 12th line, the should be added before participants.

This has now been added. Please see page 16.

11. In the references, only the first word of the title of the article should be capitalized in references 3, 4, 6, 10, 15, 17, 27, and 34. The journal for reference 3 is JAMA Oncology. The journal for references 7, 14, and 37 is European Journal of Cancer. The journal is reference 8 is BMJ. In reference 11, the last author citation should be Grand Challenge PRECISION Consortium. The journal in reference 11 is Breast Cancer Research and Treatment. In reference 13, the journal is BMJ Open. In references 18 and 29, the journal is Health Expectations. In reference 21, the journal is Lancet Oncology. In reference 24, the reference is Qualitative Research in Psychology. In reference 28, the journal is Patient Education and Counseling. In reference 30, the journal is JAMA. In reference 31, the journal is British Journal of Cancer. In references 32 and 33, the journal is Medical Decision Making. In reference 34, the journal is Thyroid. In reference 35, the journal is Urologic Oncology.

We have now updated the reference list accordingly. Please see pages 17-19.

Attachment Submitted filename: Nickel et al PLOS One response to reviewers.docx Click here for additional data file.

# Editor report: Decision Letter 1

**DOI:** 10.1371/journal.pone.0288972.r003

**Authors:** Daniele Ugo Tari

10 Jul 2023

Women’s views about current and future management of Ductal Carcinoma in Situ (DCIS): a mixed-methods study

PONE-D-23-00830R1

Dear Dr. Nickel,

We’re pleased to inform you that your manuscript has been judged scientifically suitable for publication and will be formally accepted for publication once it meets all outstanding technical requirements.

Within one week, you’ll receive an e-mail detailing the required amendments. When these have been addressed, you’ll receive a formal acceptance letter and your manuscript will be scheduled for publication.

An invoice for payment will follow shortly after the formal acceptance. To ensure an efficient process, please log into Editorial Manager at http://www.editorialmanager.com/pone/, click the 'Update My Information' link at the top of the page, and double check that your user information is up-to-date. If you have any billing related questions, please contact our Author Billing department directly at authorbilling@plos.org.

If your institution or institutions have a press office, please notify them about your upcoming paper to help maximize its impact. If they’ll be preparing press materials, please inform our press team as soon as possible -- no later than 48 hours after receiving the formal acceptance. Your manuscript will remain under strict press embargo until 2 pm Eastern Time on the date of publication. For more information, please contact onepress@plos.org.

Kind regards,

Daniele Ugo Tari, M.D.

Academic Editor

PLOS ONE

# Editor report: Acceptance letter

**DOI:** 10.1371/journal.pone.0288972.r004

**Authors:** Daniele Ugo Tari

13 Jul 2023

PONE-D-23-00830R1 

Women’s views about current and future management of Ductal Carcinoma in Situ (DCIS): a mixed-methods study 

Dear Dr. Nickel:

I'm pleased to inform you that your manuscript has been deemed suitable for publication in PLOS ONE. Congratulations! Your manuscript is now with our production department. 

If your institution or institutions have a press office, please let them know about your upcoming paper now to help maximize its impact. If they'll be preparing press materials, please inform our press team within the next 48 hours. Your manuscript will remain under strict press embargo until 2 pm Eastern Time on the date of publication. For more information please contact onepress@plos.org.

If we can help with anything else, please email us at plosone@plos.org. 

Thank you for submitting your work to PLOS ONE and supporting open access. 

Kind regards, 

PLOS ONE Editorial Office Staff

on behalf of

Dr. Daniele Ugo Tari 

Academic Editor

PLOS ONE
